# Comparative population genomics reveals evolutionary potential and conservation priorities in three *Procapra* species

**DOI:** 10.1093/molbev/msag258

**Published:** 2026-10-07

**Authors:** Haoyang Wang, Xiaofan Ma, Xin Huang, Xiaoyu Hu, Chao Zhang, Huayao Gao, Ming Lu, Yalun Wang, Tongzuo Zhang, Zexuan Gao, Yonggang Nie

**Affiliations:** State Key Laboratory of Animal Biodiversity Conservation and Integrated Pest Management, Institute of Zoology, Chinese Academy of Sciences, Beijing 100101, China; University of Chinese Academy of Sciences, Beijing 100049, China; State Key Laboratory of Animal Biodiversity Conservation and Integrated Pest Management, Institute of Zoology, Chinese Academy of Sciences, Beijing 100101, China; University of Chinese Academy of Sciences, Beijing 100049, China; Jiangxi Provincial Key Laboratory of Conservation Biology, Jiangxi Agricultural University, Nanchang 330045, China; State Key Laboratory of Animal Biodiversity Conservation and Integrated Pest Management, Institute of Zoology, Chinese Academy of Sciences, Beijing 100101, China; State Key Laboratory of Animal Biodiversity Conservation and Integrated Pest Management, Institute of Zoology, Chinese Academy of Sciences, Beijing 100101, China; State Key Laboratory of Animal Biodiversity Conservation and Integrated Pest Management, Institute of Zoology, Chinese Academy of Sciences, Beijing 100101, China; University of Chinese Academy of Sciences, Beijing 100049, China; State Key Laboratory of Animal Biodiversity Conservation and Integrated Pest Management, Institute of Zoology, Chinese Academy of Sciences, Beijing 100101, China; University of Chinese Academy of Sciences, Beijing 100049, China; State Key Laboratory of Animal Biodiversity Conservation and Integrated Pest Management, Institute of Zoology, Chinese Academy of Sciences, Beijing 100101, China; Jiangxi Provincial Key Laboratory of Conservation Biology, Jiangxi Agricultural University, Nanchang 330045, China; Qinghai Provincial Key Laboratory of Animal Ecological Genomics, Northwest Institute of Plateau Biology, Chinese Academy of Sciences, Xining 810008, China; State Key Laboratory of Animal Biodiversity Conservation and Integrated Pest Management, Institute of Zoology, Chinese Academy of Sciences, Beijing 100101, China; University of Chinese Academy of Sciences, Beijing 100049, China; State Key Laboratory of Animal Biodiversity Conservation and Integrated Pest Management, Institute of Zoology, Chinese Academy of Sciences, Beijing 100101, China; University of Chinese Academy of Sciences, Beijing 100049, China

**Keywords:** *Procapra*, genetic diversity, inbreeding, demographic history, conservation genomics

## Abstract

The genus *Procapra*, comprising the Mongolian gazelle (MG, *Procapra gutturosa*), Przewalski's gazelle (PG, *Procapra przewalskii*), and Tibetan gazelle (TG, *Procapra picticaudata*), has experienced substantial range contractions and population declines due to anthropogenic disturbance and habitat degradation. Effective conservation of these species requires a clearer understanding of how distinct demographic histories have shaped their current evolutionary potential and genetic risks. Here, we assembled chromosome-level genomes for all three *Procapra* species and conducted population genomic analyses using whole-genome resequencing data from 93 individuals. Our findings show that MG underwent substantial historical population expansion and retained the highest genetic diversity and lowest levels of inbreeding. In contrast, TG currently has a relatively large census size, but its historically small effective population size may have contributed to elevated inbreeding and reduced genetic diversity. Despite having the smallest census size among the three species, PG retained intermediate genetic diversity, consistent with its historically larger effective population size, whereas severe contemporary habitat fragmentation is associated with elevated inbreeding and a pronounced demographic decline. Collectively, our results demonstrate that census size alone does not reliably predict genetic risk in closely related *Procapra* species and highlight the importance of demographic history in shaping evolutionary potential and species-specific conservation needs. Given the escalating anthropogenic fragmentation affecting PG, targeted conservation actions are urgently needed to limit further inbreeding and erosion of genetic diversity and to safeguard its long-term viability.

## Introduction

Climate change, habitat loss, and intensifying anthropogenic pressures pose severe threats to wildlife populations, raising concerns about their long-term viability (Bellard et al. 2012; Powers and Jetz 2019). These pressures often drive population declines and elevate genetic risks by reducing genetic diversity and increasing inbreeding (Saremi et al. 2019; Furni et al. 2025; Teng et al. 2025). However, census size alone is an incomplete indicator of evolutionary potential, as even numerically large populations may retain genomic risks due to historical bottlenecks, sustained demographic declines, or restricted gene flow (Leffler et al. 2012; Westbury et al. 2019; Femerling et al. 2023; Pečnerová et al. 2024). Moreover, anthropogenic habitat fragmentation can rapidly intensify these risks by limiting dispersal and increasing inbreeding within isolated populations (Reed and Frankham 2003; Robinson et al. 2019). Therefore, comprehensive genomic assessments are crucial for accurately evaluating conservation risk and informing management, particularly in species affected by ongoing habitat degradation and fragmentation (Hu et al. 2025).

Within the order Artiodactyla, diverse gazelle lineages of the subfamily Antilopinae (family Bovidae) were once widely distributed across northern China, reflecting the historical presence of extensive suitable habitats in the region (Jiang 2004). However, climatic fluctuations and increasing anthropogenic pressures have reshaped these ecosystems, contributing to the regional disappearance or severe contraction of many gazelle lineages formerly present in the region. Today, three *Procapra* species, together with the goitered gazelle (*Gazella subgutturosa*), retain wild populations in China. These surviving *Procapra* species, the Mongolian gazelle (MG, *Procapra gutturosa*), Przewalski's gazelle (PG, *Procapra przewalskii*), and Tibetan gazelle (TG, *Procapra picticaudata*), differ markedly in census size, distribution, and conservation status. MG, categorized as Least Concern by the International Union for Conservation of Nature (IUCN), has the widest distribution and largest estimated population, with ∼2 million individuals (Buuveibaatar et al. 2024). Historically widespread across northern China and Central Asia, MG has undergone substantial range contraction associated with land-use change and overgrazing and is now largely restricted to Mongolia and adjacent regions of Russia and China (Lhagvasuren and Milner-Gulland 1997; Sokolov and Lushchekina 1997; Mendgen et al. 2023). PG, currently listed as Endangered by the IUCN and as a Class I protected species in China, was historically distributed across Gansu, Inner Mongolia, Ningxia, and Qinghai in China (Jiang et al. 1995). By the 1990s, its population had declined to fewer than 300 individuals and become restricted to fragmented habitats surrounding Qinghai Lake (Jiang et al. 2000). Although conservation initiatives have supported substantial population recovery, the species persists in severely fragmented and isolated habitat patches (Jiang et al. 1996; Ping et al. 2018). Continued human-driven landscape fragmentation likely restricts dispersal and gene flow, posing ongoing challenges to its long-term viability. In contrast, TG occupies a broad range across the Qinghai–Tibet Plateau and has an estimated population of ∼100,000 individuals (Leslie 2010). It is categorized as Near Threatened by the IUCN. Nonetheless, it also faces increasing habitat contraction and has received comparatively limited conservation attention (Lu and Wang 2003; Leslie 2010). Together, the three *Procapra* species represent contrasting demographic and ecological trajectories, providing a valuable system for examining how historical demography and recent human disturbance shape evolutionary potential.

Previous genetic studies based on mitochondrial DNA (mtDNA) and microsatellite markers have provided initial insights into the evolutionary history and genetic diversity of *Procapra*. Mitochondrial cytochrome b (*Cytb*) analyses suggested that TG is the earliest-diverging lineage, followed by MG and PG, and introgression has been reported between sympatric TG and PG (Wang and Fang 2005; Chen 2015). Earlier marker-based studies also suggested that PG had lower genetic diversity than MG and TG (Yang and Jiang 2011; Yu et al. 2017). Severe habitat fragmentation has left many remnant PG groups geographically isolated, potentially restricting dispersal and gene flow and increasing their vulnerability to loss of genetic diversity due to genetic drift and inbreeding (Yu et al. 2017; Ping et al. 2018). Nevertheless, the evolutionary and demographic dynamics of *Procapra* remain insufficiently resolved. mtDNA represents a single maternally inherited, nonrecombining locus and may therefore provide an incomplete view of species evolutionary history, whereas microsatellite markers sample only a limited fraction of the genome. Consequently, these marker systems provide limited resolution for evaluating genome-wide genetic diversity, recent inbreeding, and genetic load, which are critical for assessing genetic risks in declining and fragmented populations (Hedrick and Kalinowski 2000; Schmidt et al. 2024).

To address these gaps, we assembled chromosome-level genomes for all 3 *Procapra* species and resequenced 93 individuals, including 32 MG, 49 PG, and 12 TG. This genomic dataset enabled us to investigate population structure, demographic history, and interspecific gene flow and to quantify genome-wide genetic diversity, inbreeding, and putative genetic load, key parameters for evaluating the vulnerability of small and fragmented populations. By integrating these lines of evidence, we established a comparative population genomic framework for *Procapra* and examined how divergent demographic histories have shaped current evolutionary potential and genetic risks. Our findings provide conservation-relevant insights by characterizing contrasting genetic risks among the three species and informing management strategies for preserving the long-term viability of this gazelle lineage under accelerating environmental change.

## Results

### Chromosome-level genome assembly and annotation

To generate chromosome-level assemblies for the three *Procapra* species, we applied a standardized strategy combining PacBio HiFi long-read sequencing with Hi-C chromatin conformation capture (Tables S1 and S2). HiFi reads were assembled into contigs, yielding contig N50 values of 82.49 Mb for MG, 94.83 Mb for PG, and 91.11 Mb for TG. Hi-C scaffolding anchored contigs onto 29 autosomes and the X and Y chromosomes, resulting in scaffold N50 values of 102.69 Mb for MG, 101.90 Mb for PG, and 117.96 Mb for TG (Fig. 1a; Figures S2 and S3; Table S3). The final assembly sizes were 2.72 Gb for MG, 2.69 Gb for PG, and 2.93 Gb for TG, with unplaced scaffolds accounting for <1% of each assembly: 0.80% in MG, 0.19% in PG, and 0.33% in TG (Table S4). Assembly completeness, assessed using BUSCO on the genome assemblies, was 98.1% for both MG and PG and 98.2% for TG (Table S5). Base-level accuracy was also high, with quality value (QV) scores of 47.99, 48.98, and 48.46 for MG, PG, and TG, respectively (Table S3).

**Figure 1:**
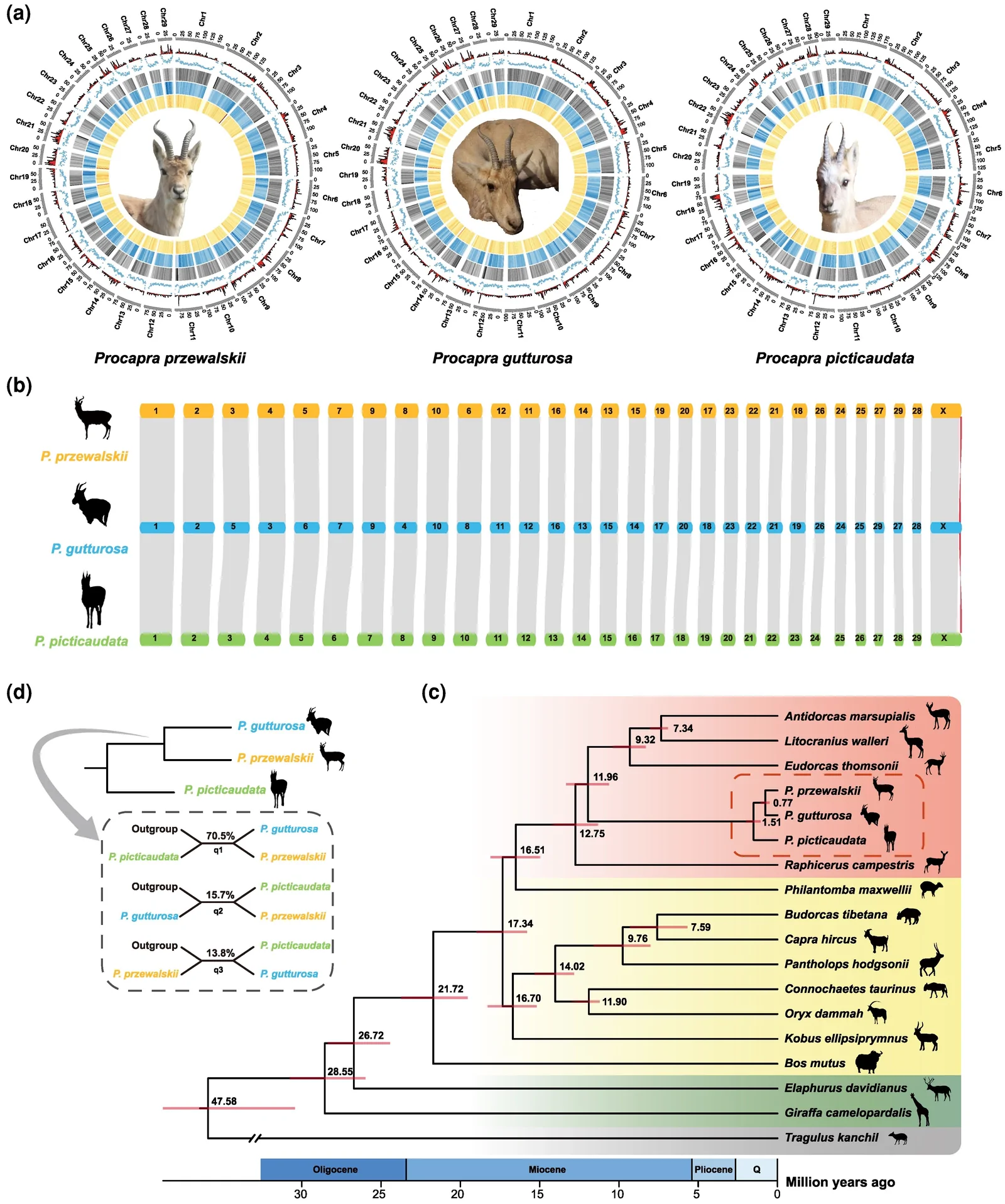
Genomic features and phylogenetic relationships of *Procapra* species. a) Circos plots of the PG, MG, and TG genomes showing chromosome organization and major genomic features, including gene density, guanine-cytosine (GC) content, long interspersed nuclear elements (LINEs), short interspersed nuclear elements (SINEs), and long terminal repeats (LTRs). b) Whole-genome synteny among the three *Procapra* species; the putative X-chromosome inversion is highlighted. c) Time-calibrated phylogeny of *Procapra* and related ruminants, with *T. kanchil* as the outgroup. Node values indicate estimated divergence times (Ma), and bars represent 95% credibility intervals. d) ASTRAL species tree based on 6,675 gene trees, with quartet frequencies shown for the three alternative *Procapra* topologies.

Following genome assembly, we annotated repetitive elements and protein-coding genes. Repetitive sequences accounted for 53.44%, 52.92%, and 56.79% of the MG, PG, and TG assemblies, respectively (Figure S4; Tables S6 and S7). We identified 21,397, 21,532, and 21,455 protein-coding genes in MG, PG, and TG, respectively, of which 20,702, 20,740, and 20,737 were functionally annotated against the NCBI non-redundant protein (NR) and Swiss-Prot databases (Figure S5; Tables S8 and S9). BUSCO analysis of the predicted protein sets showed slightly higher completeness values of 98.3% for MG, 98.8% for PG, and 99.0% for TG, indicating highly complete gene annotations across all three species (Table S10).

Whole-genome synteny analysis revealed highly conserved chromosome organization among the three species. The main structural difference detected was a putative inversion on the X chromosome between MG and both PG and TG (Fig. 1b).

### Phylogenomic reconstruction and divergence time estimation in *Procapra*

We reconstructed a genome-scale phylogeny comprising 18 ruminant species, using the lesser mouse-deer (*Tragulus kanchil*) as the outgroup (Table S11). After alignment processing and quality filtering, 6,675 orthologous alignments of annotated protein-coding sequences (CDSs) were retained, and 4-fold degenerate (4D) sites were used to infer the concatenated phylogeny and estimate divergence times. The resulting phylogeny recovered TG as the earliest-diverging lineage within *Procapra*, splitting from the common ancestor of MG and PG at ∼1.51 Ma. MG and PG subsequently diverged at ∼0.77 Ma (Fig. 1c). The overall topology of the remaining ruminant taxa was broadly consistent with previous studies (Chen et al. 2019).

To account for gene-tree discordance, we further reconstructed a coalescent-based species tree with ASTRAL using the 6,675 individual gene trees. This analysis recovered the same topology, with MG and PG forming a sister group and TG representing the earliest-diverging *Procapra* lineage. The MG–PG sister relationship received strong support, with a local posterior probability of 1.0 and quartet scores of 0.705, 0.157, and 0.138 for the primary and two alternative topologies, respectively (Fig. 1d).

### Population structure analysis

For interspecific population structure analyses, resequencing reads were mapped to the chromosome-level PG reference genome to provide a common genomic coordinate system across species. The average sequencing depths were 13.18× for MG, 23.23× for PG, and 22.49× for TG, with corresponding genome coverages of 99.13%, 99.34%, and 97.68%, respectively (Table S13).

Principal component analysis (PCA) and the autosomal neighbor-joining (NJ) tree consistently separated MG, PG, and TG into three distinct species-level genetic clusters (Figs. 2b and c and S8). PG individuals showed broader dispersion within the PCA space, indicating within-species genetic heterogeneity. ADMIXTURE analyses based on 100 independent replicates for each *K* supported *K* = 3 as the best-fitting species-level clustering pattern, with MG, PG, and TG corresponding to three major ancestry components (Figs. 2d and S9). Pairwise *F*_ST_ estimates were consistent with these patterns, with the strongest differentiation observed between PG and TG (0.605) and the weakest between PG and MG (0.201) (Fig. 2c).

**Figure 2:**
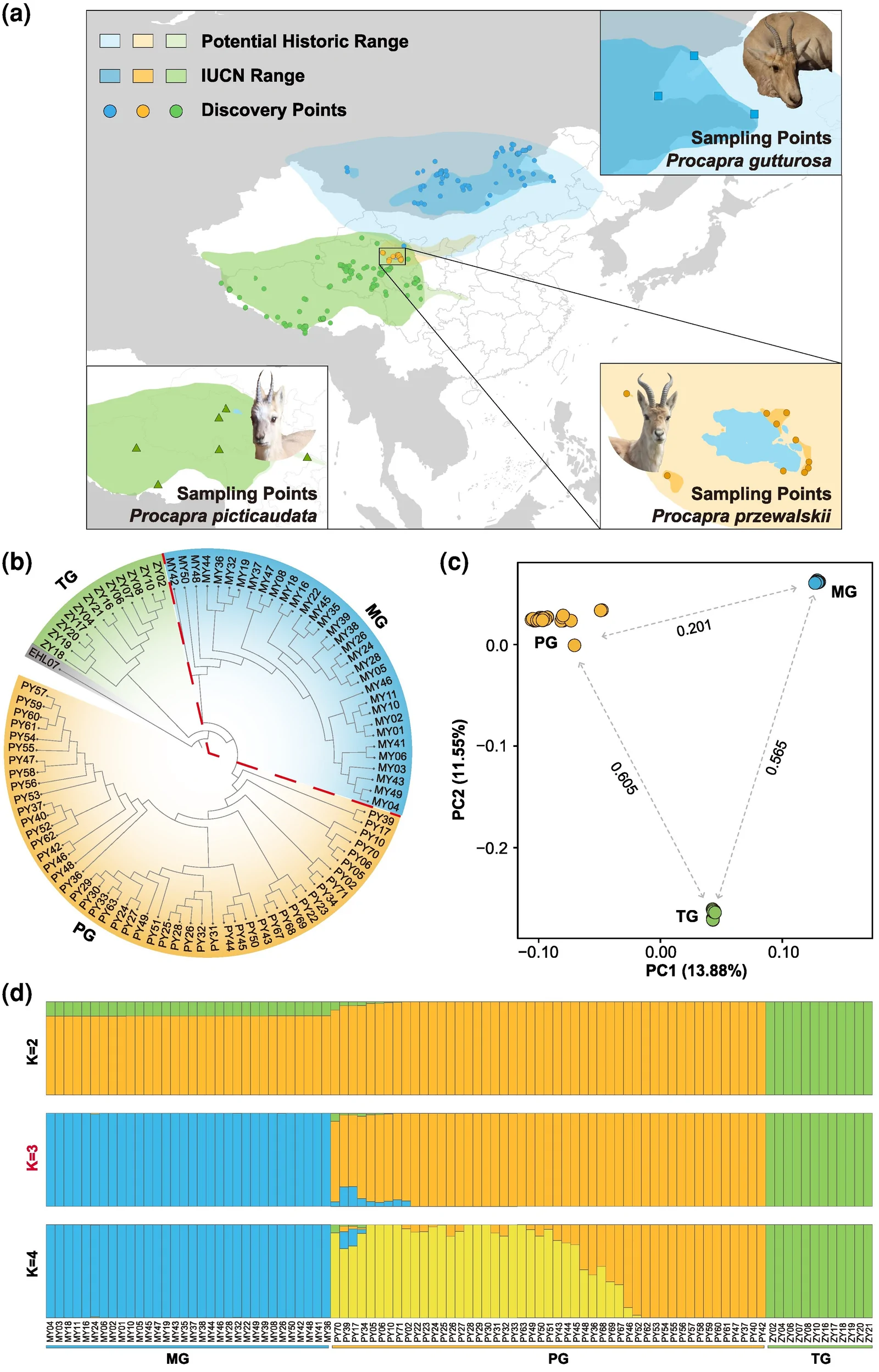
Geographic distribution and population structure of *Procapra* species. a) Potential historical ranges, current IUCN ranges, occurrence records, and sampling locations of MG, PG, and TG. Insets show the sampling locations for each species. b) Autosomal NJ tree of the three *Procapra* species, with *G. subgutturosa* as the outgroup. c) PCA of MG, PG, and TG; dashed lines indicate pairwise *F*_ST_ values. d) ADMIXTURE results for *K* = 2 to 4, with *K* = 3 representing the best-supported species-level clustering.

Species-specific analyses further revealed distinct patterns of within-species genetic variation. Although ADMIXTURE analyses favored *K* = 1 for all three species under the current sampling scheme, species-specific PCA revealed additional within-species variation that was not resolved as discrete ancestry components by ADMIXTURE (Figs. S10 to S13). In MG, PCA showed no consistent geographic subdivision among the sampled individuals. In PG, PCA indicated genetic heterogeneity; however, clearly delineated geographic clusters could not be reliably identified given the uneven representation of its fragmented remnant groups. The TG-specific PCA revealed three geographically associated genetic clusters, corresponding to individuals from Tianjun in Qinghai, Nagqu and Shannan in the Tibet Autonomous Region, and Dulan in Qinghai together with sampled localities in Sichuan. Taken together, the ADMIXTURE results suggest that the sampled TG individuals are characterized by a broadly shared genetic background, together with finer-scale geography-associated differentiation revealed by PCA. Because the primary objective of this study was to compare evolutionary histories and conservation-relevant genomic variation among the three *Procapra* species, subsequent comparative analyses were conducted primarily at the species level.

Mitochondrial genome phylogeny revealed that four PG individuals clustered with TG, a pattern consistent with historical maternal introgression from TG into PG (Fig. S6). In contrast, the Y-chromosome NJ tree recovered species-specific clustering without evident interspecific mixing (Fig. S7).

### Contrasting demographic histories among *Procapra* species

Pairwise Sequentially Markovian Coalescent (PSMC) and SMC++ analyses revealed markedly contrasting demographic trajectories among the three *Procapra* species (Fig. 3a and b). MG and PG both experienced pronounced historical reductions in effective population size (*N*_e_), followed by substantial population expansions, whereas TG maintained a comparatively low *N*_e_ over much of its demographic history. The early declines in MG and PG approximately coincided with the Naynayxungla Glaciation (NG; ∼780 to 500 kya), whereas their subsequent expansions reached maxima around the Penultimate Glaciation (∼300 to 130 kya). In contrast, TG showed no comparable historical expansion and maintained a relatively constrained demographic trajectory.

**Figure 3:**
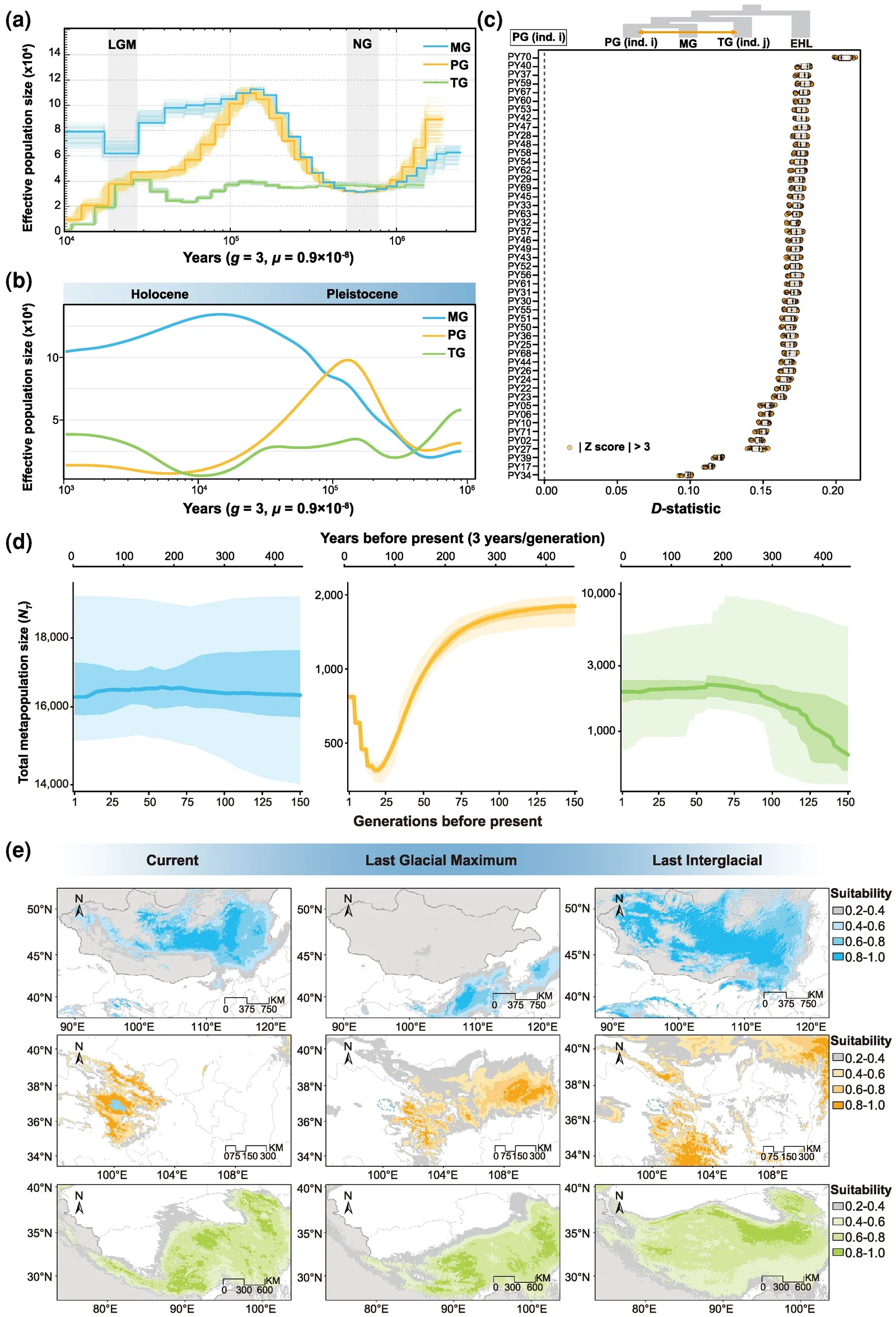
Demographic history, interspecific allele sharing, and habitat suitability of *Procapra* species. a) Historical demographic trajectories inferred using PSMC with 100 bootstrap replicates. Shaded areas indicate the LGM and NG. b) Demographic trajectories inferred using SMC++. c) Individual-level *D*-statistics testing excess allele sharing between PG and TG; points corresponding to tests with |Z| > 3 are highlighted. d) Recent demographic trajectories inferred using GONE2 under the structure-aware metapopulation model. Lines show median estimates of total metapopulation size (*N*_T_) across 30 replicates, with shaded regions representing the 50% and 95% empirical intervals. e) Predicted habitat suitability under current, LGM, and LIG conditions; rows correspond to MG, PG, and TG.

More recent demographic changes also differed among species. Around the Last Glacial Maximum (LGM; ∼21 kya), PG and TG showed pronounced declines in *N*_e_, whereas MG retained a comparatively larger effective population size. Species distribution models revealed species-specific changes in predicted climatic suitability across the three climatic periods (Fig. 3e). Predicted suitable habitat for PG and TG was substantially reduced during the LGM relative to the Last Interglacial (LIG), whereas MG retained a comparatively broad area of suitable habitat despite a southward distributional shift. Following the LGM, SMC++ indicated demographic expansion in MG, while PG and TG remained at relatively low *N*_e_ after more limited recoveries.

Recent demographic inference further highlighted contrasting population dynamics among species. PopSizeABC indicated recent demographic declines of varying magnitude across all three species (Fig. S14). GONE2 showed that MG maintained a relatively large and stable total metapopulation size (*N*_T_) of ∼16,000 over recent generations (Fig. 3d). In contrast, PG declined markedly from ∼1,800 around 150 generations ago to ∼300 around 20 generations ago, followed by a modest recent increase to ∼700. TG showed a different trajectory, with *N*_T_ increasing from ∼600 earlier in the inferred period to ∼2,000 toward the present. Together, these analyses reveal substantially different historical and recent demographic trajectories among MG, PG, and TG.

### Interspecific allele sharing among *Procapra* species

Given the historical range overlap among *Procapra* species, we evaluated patterns of interspecific allele sharing using multiple complementary approaches. Species-level *D*-statistics calculated with qpDstat revealed significant excess allele sharing between TG and PG (Fig. S15; Table S15). Individual-level tests further showed that PG–TG allele sharing was widespread across PG individuals, although its magnitude varied among individuals (Fig. 3c; Table S16). PY70 exhibited the strongest TG-related allele-sharing signal and was sampled from the only region where PG and TG currently remain sympatric. Several PG individuals from northeastern localities, including PY40 and PY59, also showed relatively elevated TG-related allele sharing (Fig. S17). Patterns in the PCA and ADMIXTURE analyses also suggested possible MG-associated ancestry in a subset of PG individuals, motivating additional individual-level tests of MG–PG allele sharing. These tests showed that PY17 and PY39 had comparatively stronger allele sharing with MG and weaker TG-related signals, indicating substantial heterogeneity in interspecific allele-sharing patterns among PG individuals (Figs. S16 and S17; Tables S16 and S17).

Recent admixture analysis using apoh detected no evidence of hybrid pedigrees within the past few generations, suggesting that the observed allele-sharing patterns are unlikely to result primarily from very recent hybridization (Table S18). Dsuite *f*-branch analysis showed the strongest excess allele sharing between PG and TG, consistent with the qpDstat results (Fig. S18). Together, these analyses support a complex history of interspecific allele sharing among *Procapra* species.

### Genetic diversity and linkage disequilibrium in *Procapra*

To compare genomic diversity among the three *Procapra* species while minimizing cross-species reference bias, resequencing reads were mapped to the corresponding species-specific reference genomes. Average genome coverage was 99.07% for MG, 99.34% for PG, and 98.28% for TG (Table S13), supporting reliable downstream comparisons.

Genome-wide heterozygosity differed markedly among the three species. MG exhibited the highest heterozygosity (2.26 × 10^−3^), followed by PG (1.43 × 10^−3^), whereas TG showed the lowest value (1.14 × 10^−3^) (Fig. 4a; Table S22). All pairwise comparisons were statistically significant, and TG showed greater variation in heterozygosity among individuals (Table S23). Nucleotide diversity (π) showed a similar pattern, with MG having the highest value (2.37 × 10^−3^), followed by PG (1.60 × 10^−3^) and TG (1.50 × 10^−3^) (Fig. 4b; Table S24). Linkage disequilibrium (LD) decay also differed substantially among species (Fig. 4c). MG exhibited the most rapid LD decay, TG the slowest, and PG an intermediate pattern, broadly consistent with their relative levels of genome-wide genetic diversity.

**Figure 4:**
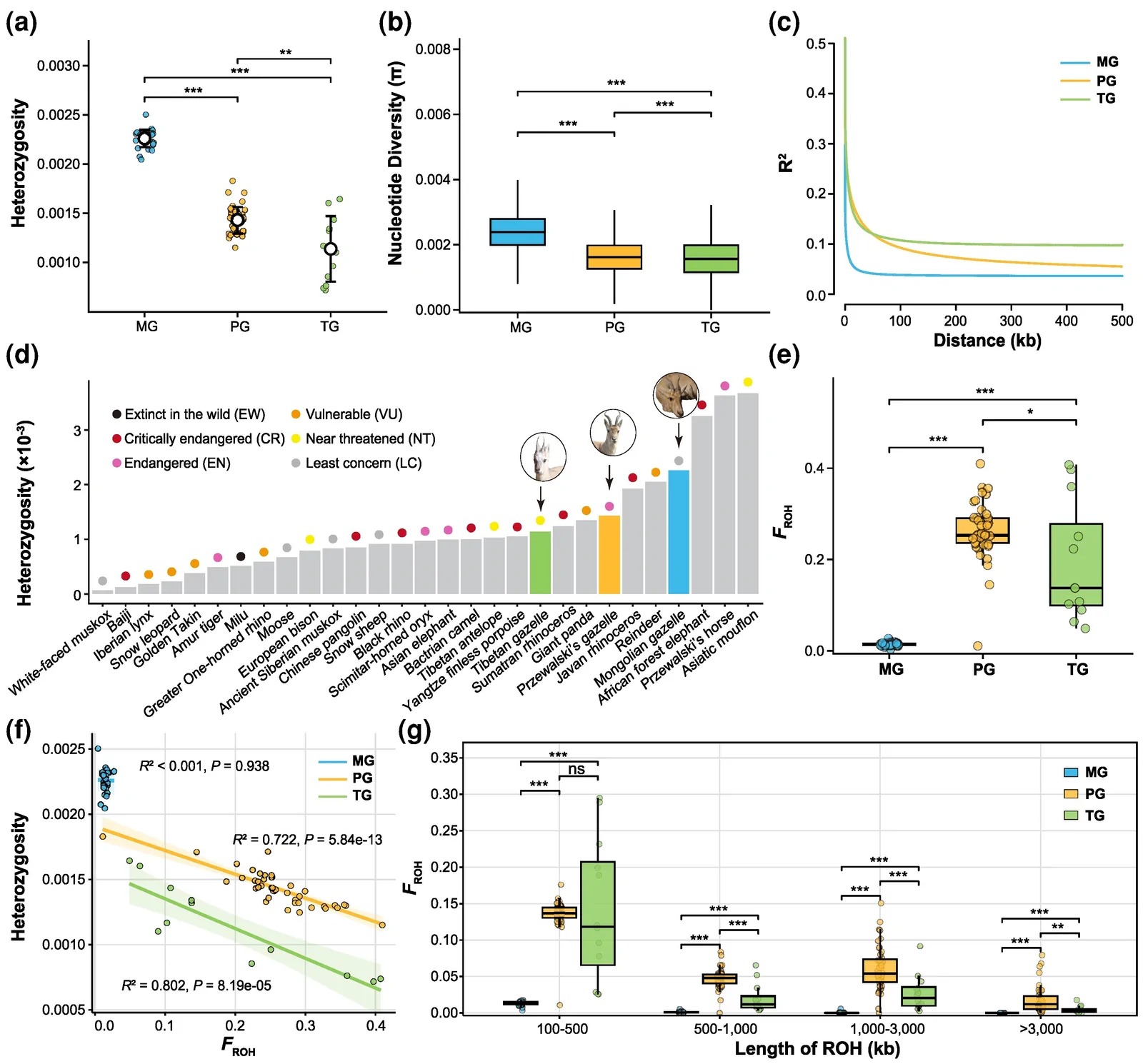
Genomic diversity, LD, and inbreeding in *Procapra* species. a) Genome-wide heterozygosity in MG, PG, and TG. b) Nucleotide diversity (π) among the three species. c) LD decay. d) Comparison of genome-wide heterozygosity across selected mammalian species, with colored points indicating IUCN conservation status. e) Total genomic inbreeding coefficient (*F*_ROH_). f) Relationship between genome-wide heterozygosity and *F*_ROH_; shaded areas indicate 95% confidence intervals. g) *F*_ROH_ contributed by ROHs of different length classes.

Despite having the smallest contemporary census size, PG retained intermediate levels of genetic diversity. Given the evidence of historical allele sharing between PG and TG, we further examined whether putative introgression regions were associated with elevated heterozygosity in PG. Dsuite Dinvestigate analyses identified individual-specific putative introgression regions, within which heterozygosity was consistently higher than in the corresponding genomic background. Excluding these regions resulted in a small but consistent reduction in genome-wide heterozygosity, suggesting that historical allele sharing with TG may have contributed to genetic variation retained in PG (Fig. S22; Table S25).

To place these estimates in a broader conservation context, we compared genome-wide heterozygosity with published estimates from other mammals of conservation concern (Fig. 4d; Table S26). MG showed heterozygosity similar to that reported for reindeer (*Rangifer tarandus*, 2.05 × 10^−3^), whereas TG showed values comparable to those of Sumatran rhinoceros (*Dicerorhinus sumatrensis*, 1.24 × 10^−3^) and Yangtze finless porpoise (*Neophocaena asiaeorientalis*, 1.05 × 10^−3^). PG retained higher heterozygosity than several threatened mammals included in the comparison, including Asian elephant (*Elephas maximus*, 9.93 × 10^−4^) and scimitar-horned oryx (*Oryx dammah*, 9.70 × 10^−4^). Overall, the three *Procapra* species retained appreciable genome-wide heterozygosity relative to several threatened mammals.

### Inbreeding profiles of *Procapra* species

To assess genomic inbreeding across the three *Procapra* species, we identified runs of homozygosity (ROHs) and calculated the genomic inbreeding coefficient (*F*_ROH_) as the proportion of the autosomal genome contained within ROHs. Mean *F*_ROH_ values were 0.014 for MG, 0.261 for PG, and 0.194 for TG (Fig. 4e; Table S27). Despite retaining intermediate genome-wide diversity, PG exhibited the highest level of genomic inbreeding, followed by TG, whereas MG showed substantially lower inbreeding. *F*_ROH_ was negatively associated with genome-wide heterozygosity, with more highly inbred individuals generally exhibiting lower heterozygosity (Fig. 4f).

To examine variation in ROH length distributions, ROHs were classified into four length categories: 100 to 500, 500 to 1,000, 1,000 to 3,000, and >3,000 kb, with longer ROHs generally reflecting more recent parental relatedness (Fig. 4g; Table S27). MG consistently showed the lowest *F*_ROH_ across all ROH length classes, and no MG individuals harbored ROHs longer than 3,000 kb. In contrast, PG showed the highest contribution from long ROHs, particularly those >1,000 kb, indicating a stronger signature of recent inbreeding. TG showed elevated contributions from shorter ROHs, particularly in the 100- to 500-kb class, together with substantial variation among individuals. This pattern is compatible with both more distant shared ancestry and the accumulation of background relatedness under long-term small effective population size.

### Genetic load and functional enrichment

To evaluate putative genetic load, we identified synonymous (SYN), missense, and loss-of-function (LOF) variants, with missense variants further classified as tolerated (TOL) or deleterious (DEL). We first compared the numbers of homozygous-derived and heterozygous variants across functional categories (Fig. 5a; Table S28). For heterozygous SYN, TOL, and DEL variants, MG carried the highest numbers, followed by PG, whereas TG consistently carried the fewest. MG also carried the highest number of heterozygous LOF variants, whereas homozygous-derived LOF counts were lower in PG and broadly comparable between MG and TG. TG individuals carried an average of 87 heterozygous LOF variants, significantly fewer than MG and PG. Normalization by the total number of protein-coding genes in each genome produced highly similar patterns (Fig. S23). However, when heterozygous LOF and DEL variant counts were normalized by the total number of genome-wide heterozygous single-nucleotide polymorphisms (SNPs) in each individual, neither ratio differed significantly between TG and PG, whereas both ratios were significantly higher in TG than in MG (Fig. S24). Heterozygous LOF and DEL variant counts were also normalized by the corresponding numbers of heterozygous SYN variants, with both LOF/SYN and DEL/SYN ratios significantly higher in TG than in MG and PG (Fig. S24).

**Figure 5:**
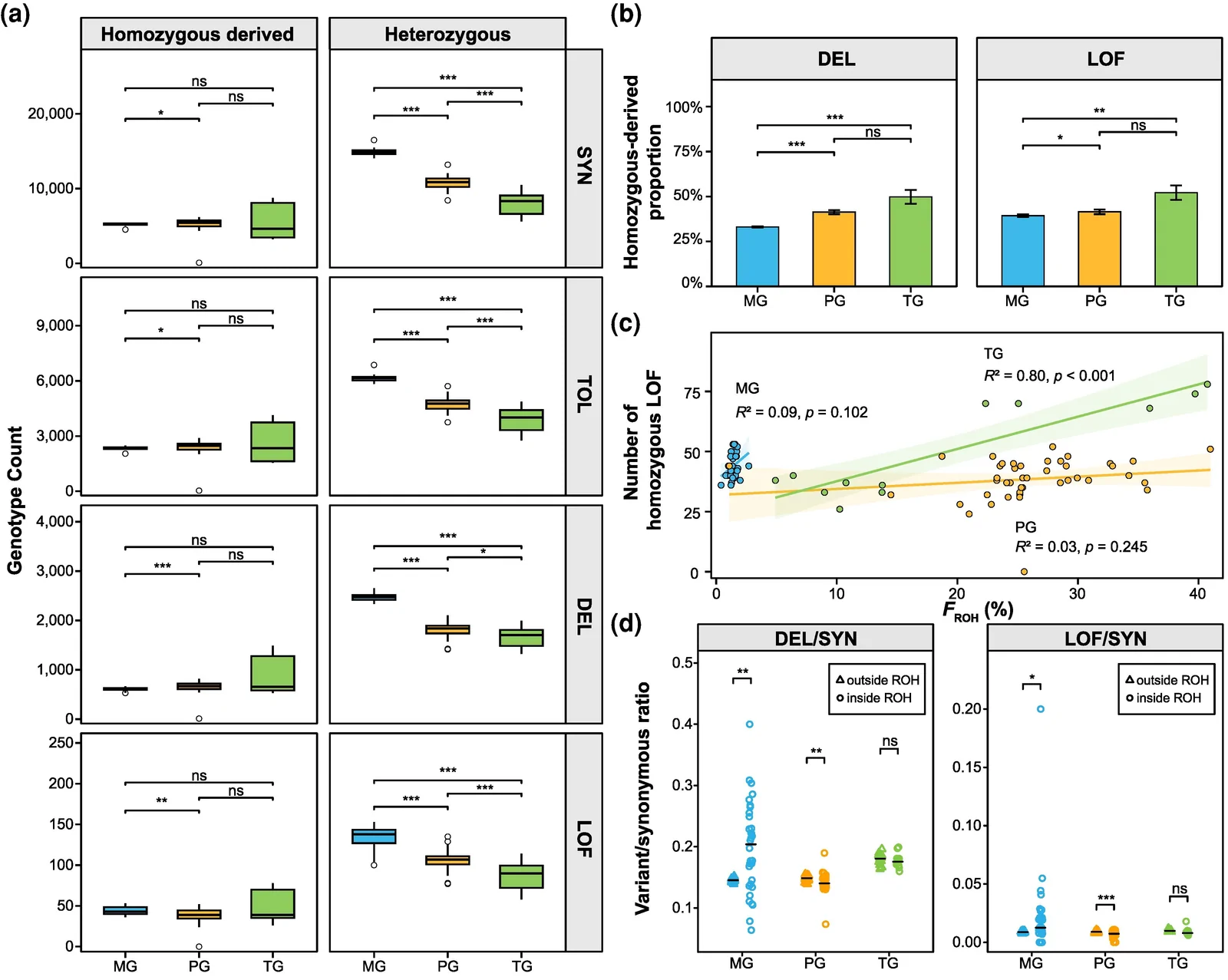
Putative genetic load in *Procapra* species. a) Numbers of SYN, TOL missense, DEL missense, and LOF variants in the homozygous-derived and heterozygous states. b) Proportions of derived DEL and LOF alleles carried in the homozygous state among MG, PG, and TG. c) Relationship between homozygous-derived LOF variant counts and *F*_ROH_; shaded areas indicate 95% confidence intervals. d) DEL/SYN and LOF/SYN ratios inside and outside ROHs; triangles and circles represent values outside and inside ROHs, respectively.

We next quantified the proportion of derived DEL and LOF alleles carried in the homozygous state. TG showed the highest homozygous-derived proportions for both categories, reaching 50% for DEL and 52% for LOF (Fig. 5b). The number of homozygous-derived LOF variants increased significantly with *F*_ROH_ in TG (Fig. 5c), indicating that individuals with greater genomic inbreeding tended to carry more LOF variants in the homozygous state.

We then compared derived LOF/SYN and DEL/SYN allele count ratios between ROH and non-ROH regions (Fig. 5d; Table S29). In TG, LOF/SYN was numerically lower within ROHs but did not differ significantly between ROH and non-ROH regions, whereas DEL/SYN showed no significant difference. PG showed higher LOF/SYN and DEL/SYN ratios outside ROHs, while ROH-based comparisons in MG were less informative because of its very low overall inbreeding and limited occurrence of ROHs.

To further characterize the functional associations of potentially damaging variation, we identified 495, 306, and 332 genes carrying LOF variants in MG, PG, and TG, respectively (Table S30). We then performed Gene Ontology (GO) Biological Process and Kyoto Encyclopedia of Genes and Genomes (KEGG) pathway enrichment analyses for genes carrying LOF and DEL variants (Fig. S25; Table S31). LOF-carrying genes showed species-specific top-ranked functional terms, including translation and microtubule organization in MG, RNA processing in PG, and immune-regulatory and translation-related functions in TG, although only a subset remained significant after multiple-testing correction. In contrast, DEL-carrying genes showed broader and more consistent enrichment across the three species, including several significantly enriched ribosomal and immune-related functions. These results indicate both shared and species-specific functional patterns among genes carrying potentially damaging variants.

## Discussion

### Population structure and historical connectivity in *Procapra*

Genome-wide analyses revealed clear differentiation among MG, PG, and TG, together with distinct patterns of within-species genetic variation. Among the sampled MG individuals, no consistent geographic subdivision was detected, possibly reflecting the extensive seasonal movements that maintain connectivity across northern China and Mongolia (Zhang et al. 1995; Okada et al. 2015). However, because our sampling covered only the Chinese portion of the species’ range, broader sampling will be required to assess population structure across its full distribution. By contrast, TG showed a more geographically structured pattern. ADMIXTURE favored *K* = 1 under the current sampling scheme, suggesting a broadly shared genetic background among the sampled individuals, whereas the TG-specific PCA revealed three geographically associated genetic clusters. Taken together, these results suggest that the sampled TG individuals share a broadly similar genetic background while also exhibiting clear geography-associated differentiation. Previous studies have likewise identified geographic differentiation within TG and proposed regional management units (Zhang and Jiang 2006), providing further support for spatial genetic heterogeneity across the species. Given the limited sampling of TG across its broad geographic range, the observed patterns primarily reflect the sampled individuals and regions. More extensive and geographically representative genomic sampling will be important for characterizing species-wide genetic variation and population structure and for further refining population boundaries and conservation units. PG likewise showed genetic heterogeneity, although discrete geographic populations were not clearly resolved. This pattern likely reflects both its highly fragmented distribution and the uneven representation of its numerous remnant groups in the present dataset. Earlier marker-based studies also reported differentiation among geographically isolated PG groups (Lei et al. 2003; Yang and Jiang 2011; Yu et al. 2017). Because severe fragmentation has intensified relatively recently (Jiang 2004), there may have been insufficient time for genetic drift to generate pronounced genome-wide divergence among all isolated groups. Nevertheless, the observed heterogeneity indicates that fine-scale population structure within PG remains to be resolved, and more extensive sampling across remnant groups will be necessary to clarify population boundaries and contemporary connectivity.

The genomic data further revealed heterogeneous historical connectivity among the three species. The strongest interspecific allele-sharing signal occurred between PG and TG, as supported by both qpDstat and Dsuite *f*-branch analyses. PG individuals also showed geographic variation in TG- and MG-related allele sharing, with PY70, sampled from the only region where PG and TG currently remain sympatric, showing the strongest TG-related signal. The mitochondrial phylogeny, in which four PG individuals clustered with TG, is also consistent with historical maternal introgression. In contrast, apoh detected no hybrid pedigrees within the past few generations, suggesting that the observed genome-wide patterns are unlikely to be driven primarily by very recent hybridization. Together, these results support a complex history of interspecific connectivity in *Procapra*, likely facilitated by broader historical range overlap documented in published distribution records (Chen 2015; Hu et al. 2015) (Table S32), whereas contemporary connectivity has become increasingly restricted by range contraction and habitat fragmentation (Li et al. 2010).

### Contrasting demographic histories shape evolutionary potential and genetic risks

The three *Procapra* species have experienced markedly different demographic histories, providing an important context for their contrasting levels of genetic diversity, inbreeding, and putative genetic load. TG diverged from the common ancestor of MG and PG at ∼1.51 Ma, followed by the divergence of MG and PG at ∼0.77 Ma. These events occurred against a background of continued tectonic and environmental reorganization of the Qinghai–Tibet Plateau and surrounding regions (An et al. 2001; Li et al. 2014). Subsequent demographic changes broadly overlapped with major Quaternary climatic oscillations, although the relationship between predicted climatic suitability and inferred effective population size varied among species. Around the LGM, contractions in predicted suitable habitat in PG and TG coincided with declines in effective population size, whereas MG retained a comparatively broad area of suitable habitat despite a different demographic trajectory. Overall, the two approaches showed broadly consistent but not fully concordant patterns, reflecting their complementary perspectives on past climatic suitability and demographic history.

Recent demographic reconstructions further emphasized these differences. MG maintained a comparatively large and stable recent metapopulation size, whereas PG experienced a pronounced recent contraction followed by a modest increase. The recent decline in PG broadly corresponds to the severe range loss, fragmentation, and isolation experienced by the species (Yu et al. 2017; Ping et al. 2018). Although MG and TG have also experienced anthropogenic pressures, both retain substantially larger and more continuous ranges than PG, likely buffering their populations from similarly severe demographic contraction. TG showed a different recent trajectory, with GONE2 indicating an increase from a relatively small metapopulation size toward the present, although with broader uncertainty. Thus, contemporary census size alone provides an incomplete representation of the demographic histories experienced by the three species.

These contrasting histories are reflected in present-day genomic diversity. MG showed the highest genome-wide diversity and the most rapid LD decay, whereas TG exhibited the lowest diversity and slowest LD decay, consistent with their historically large and small effective population sizes, respectively. PG retained intermediate genetic diversity despite having the smallest contemporary census size. Present-day heterozygosity is shaped largely by historical population size and the duration and severity of demographic bottlenecks (Nigenda-Morales et al. 2023). The historically larger population size of PG may therefore have buffered the loss of diversity during its relatively recent decline. Historical allele sharing with TG may have provided an additional contribution, although demographic history likely represents the primary explanation for the diversity retained in PG. Because genomic diversity can lag behind demographic decline, the diversity currently retained in PG should not be interpreted as long-term genetic security; continued small population size and restricted connectivity are expected to erode genetic diversity and intensify inbreeding over subsequent generations.

Inbreeding profiles further revealed contrasting ROH distributions. MG showed very low genomic inbreeding and few long ROHs. PG exhibited the highest overall *F*_ROH_ and the greatest contribution from long ROHs, indicating a strong recent component of inbreeding consistent with increasing fragmentation and isolation among remnant groups. TG also showed substantial inbreeding, but with a greater contribution from shorter ROHs, a pattern that may reflect older inbreeding and the effects of its long-term small effective population size.

These demographic differences were also reflected in patterns of putatively deleterious variation, including DEL and LOF variants. After accounting for genome-wide heterozygosity and SYN variation, TG showed relatively high levels of heterozygous deleterious variation, with both LOF/SYN and DEL/SYN ratios higher than those in MG and PG. In addition, LOF/SYN and DEL/SYN ratios were broadly similar between ROH and non-ROH regions in TG. The elevated DEL/SYN ratio in TG is consistent with reduced efficacy of purifying selection under its long-term small effective population size, where genetic drift can weaken selection against mildly deleterious variants (Robinson et al. 2023). By contrast, MG retained more deleterious variation in the heterozygous state, consistent with its high genomic diversity and low inbreeding, whereas the combination of substantial deleterious variation and high recent inbreeding in PG may increase the probability of recessive harmful alleles becoming homozygous. Functional enrichment analyses further identified associations with fundamental cellular and immune-related processes, with both shared and species-specific patterns among the three species. Although these associations do not demonstrate direct fitness consequences, they provide candidate genes and pathways that could be incorporated into future genomic monitoring. In particular, conservation assessments of small populations such as PG should consider not only genome-wide diversity and inbreeding, but also the distribution and homozygosity of potentially damaging variants when evaluating genetic risks and prioritizing future management.

### Species-specific conservation implications

The contrasting genomic profiles of the three *Procapra* species indicate that conservation priorities should not be based on contemporary census size alone. PG requires the most immediate attention because ongoing fragmentation and restricted connectivity are accompanied by pronounced recent inbreeding and demographic decline. Conservation efforts should therefore prioritize preventing further isolation, restoring habitat connectivity where feasible, and reducing barriers such as fencing that restrict dispersal (You et al. 2013; Yu et al. 2017). Managed translocations or other forms of assisted gene flow may be considered for highly isolated groups, but such interventions should be preceded by finer-scale genomic and ecological assessment because PG shows genetic heterogeneity and its population boundaries remain incompletely resolved.

TG presents a different challenge. Its low genetic diversity, elevated inbreeding, and geography-associated differentiation indicate that conservation planning should both preserve existing genetic variation and maintain connectivity among regional groups. More extensive sampling across the species’ range will also be necessary before formal conservation units can be robustly delineated.

MG currently retains the most favorable genetic status among the three species, but continued large-scale movement and connectivity are likely important for maintaining this status. Conservation should therefore focus on protecting migratory corridors, reducing infrastructure barriers, and strengthening cross-border habitat connectivity between China and Mongolia.

Overall, by integrating chromosome-level genome assemblies with population genomic analyses across all three extant *Procapra* species, our study provides a comparative genomic framework for the genus, reveals how contrasting demographic histories have shaped present-day genetic risks, and contributes genomic resources for an important component of regional ungulate biodiversity. These comparisons show that contemporary census size alone does not fully capture genomic condition, highlighting the value of integrating demographic history with genomic information in conservation assessment. Broader population-level sampling will be needed to resolve fine-scale genetic variation and inform population-specific conservation management.

## Materials and methods

### Sample collection and sequencing

To generate chromosome-level genome assemblies, we collected one male individual of each *Procapra* species. Muscle tissue from MG (*P. gutturosa*) was obtained from a naturally deceased wild individual in Hulunbuir, Inner Mongolia, China; blood samples from PG (*P. przewalskii*) and TG (*P. picticaudata*) were obtained from a wild individual in Haiyan, Qinghai, and a captive individual at Xining Zoo, Qinghai, respectively (Table S1). High-molecular-weight DNA was extracted using the cetyltrimethylammonium bromide (CTAB) method. SMRTbell libraries were prepared using the SMRTbell Prep Kit 3.0 and sequenced on the PacBio Revio platform. HiFi reads were generated using CCS software. Hi-C libraries were commercially prepared by GrandOmics Biosciences (Wuhan, China) using a standard DpnII-based protocol involving formaldehyde crosslinking, DpnII digestion, biotin labeling, proximity ligation, and DNA fragmentation, and were sequenced on the DNBSEQ-T7 platform.

For whole-genome resequencing, we sampled 93 individuals, including 32 MG, 49 PG, and 12 TG (Table S1). All samples were field-collected tissues; samples collected in earlier years were maintained in frozen tissue collections and processed using the same procedures as recently collected samples. A goitered gazelle (*G. subgutturosa*; EHL07) from Shanshan County, Xinjiang, China, was included as an outgroup. Genomic DNA was extracted using the CTAB method, and 200 to 400 bp paired-end libraries were sequenced on the DNBSEQ-T7 platform using a 2 × 150 bp strategy.

### Genome assembly, annotation, and assessment

Before genome assembly, genome sizes of the three *Procapra* species were estimated using 21-mer analysis based on clean paired-end reads (Fig. S1). PacBio HiFi reads were assembled with Hifiasm (v.0.20.0-r639) (Cheng et al. 2021) using default parameters. Purge_dups (v.1.2.5) (Guan et al. 2020) was used to remove haplotypic duplications and redundant haplotigs, generating a nonredundant primary assembly for each species. Hi-C reads were then used to scaffold contigs with YaHS (v.1.2) (Zhou et al. 2023), followed by manual curation and correction using Juicebox (v.2.17.00) (Durand et al. 2016). Hi-C contact maps were visualized at 1-Mb resolution using HiCExplorer (v.3.7.6) (Wolff et al. 2020).

For genome annotation, repetitive elements were first identified using multiple approaches. Tandem repeats were predicted with Tandem Repeat Finder (v.4.10.0) (Benson 1999) using the parameters “2 7 7 80 10 50 500 -f -d -h -r.” An ab initio repeat library was constructed with RepeatModeler (v.2.0.3) (Flynn et al. 2020). RepeatMasker (v.4.1.3) and RepeatProteinMask (v.4.1.3) (Tarailo-Graovac and Chen 2009) were then used to identify repetitive elements based on the RepBase database (v.20181026) (Jurka et al. 2005) and the ab initio repeat library. Protein-coding genes were predicted from the repeat-masked genomes by integrating de novo and homology-based approaches. Augustus (v.3.2.1) (Stanke et al. 2008) was used for de novo gene prediction, whereas homology-based gene prediction was performed using GeMoMa (v.1.9) (Keilwagen et al. 2019). Gene models from these approaches were integrated with EvidenceModeler (v.1.1.1) (Haas et al. 2008). The resulting gene models were further filtered to remove low-confidence predictions and integrated with retained high-confidence gene models to generate the final protein-coding gene set for each species. Gene function annotation was performed using the NR and Swiss-Prot databases with Diamond (v.2.0.14.152) (Buchfink et al. 2015).

Assembly and annotation completeness were evaluated separately using BUSCO (v.5.7.1) with the mammalia_odb10 dataset (Simão et al. 2015), based on genome sequences and predicted protein sets, respectively. Mapping rates and genome coverage were calculated by aligning HiFi reads and clean paired-end reads to the corresponding assemblies using BWA-MEM (v.0.7.17-r1188) (Li and Durbin 2009) and SAMtools (v.1.6) (Li et al. 2009). Base-level consensus QVs were estimated using Merqury (v.1.1) (Rhie et al. 2020) with k = 21. CIRCOS (Krzywinski et al. 2009) was used to visualize genome features. In addition, chromosome-level synteny among the three *Procapra* species was assessed using JCVI (Tang et al. 2024) based on their annotated gene sets.

### Phylogeny reconstruction and divergence time estimation

To investigate phylogenetic relationships and estimate divergence times within the genus *Procapra*, we constructed a genome-scale dataset including the three *Procapra* species, 14 additional ruminant species, and the lesser mouse-deer (*T. kanchil*) as the outgroup (Table S11). Whole-genome pairwise alignments were generated using LASTZ (Harris 2007) and combined into a reference-guided multiple-genome alignment using MULTIZ (Blanchette et al. 2004), with the *Bos taurus* ARS-UCD2.0 genome (GCF_002263795.3) used as the common coordinate reference. Annotated CDSs were extracted from the multiple-genome alignment and processed at the codon level using MACSE (v.2.06) (Ranwez et al. 2018) to remove poorly aligned sequences and account for frameshifts and premature stop codons. After quality filtering, 6,675 orthologous CDS alignments were retained for downstream analyses.

4D sites were extracted from the concatenated CDS alignments and used to reconstruct the maximum-likelihood (ML) phylogeny with IQ-TREE2 (Minh et al. 2020). ModelFinder selected GTR + F + R3 as the best-fitting nucleotide substitution model according to the Bayesian information criterion, and the concatenated 4D-site matrix was analyzed as a single partition. Branch support was assessed using 1,000 ultrafast bootstrap replicates and the Shimodaira-Hasegawa-like approximate likelihood ratio test (SH-aLRT) with 1,000 replicates. Divergence times were estimated using MCMCTree implemented in PAML (Yang 2007), with calibration constraints obtained from TimeTree and previous studies (Table S12) (Kumar et al. 2017).

To account for gene-tree discordance, ML trees were independently reconstructed for each of the 6,675 orthologous CDS alignments using IQ-TREE2. The best-fitting nucleotide substitution model for each gene was selected by ModelFinder according to the Bayesian information criterion, and the resulting gene trees were summarized using ASTRAL (Zhang et al. 2018) to infer the species tree and quantify quartet support.

### SNP calling and quality filtering

Raw paired-end resequencing reads were processed using fastp (v.0.23.2) (Chen et al. 2018) with default parameters to remove low-quality reads and adapter contamination. For species-specific analyses, clean reads were aligned to the corresponding reference genome using BWA-MEM. Alignments were sorted and indexed using SAMtools, and polymerase chain reaction duplicates were marked using Picard (v.2.25.5) (http://broadinstitute.github.io/picard). Scaffolds shorter than 1 Mb were excluded from downstream analyses to reduce potential artifacts associated with short or poorly assembled regions.

SNP calling was performed using GATK (v.3.8) (McKenna et al. 2010). “HaplotypeCaller” was run for each individual in Genomic Variant Call Format (GVCF) mode, followed by joint genotyping using “CombineGVCFs” and “GenotypeGVCFs.” Raw variants were filtered using “VariantFiltration” with the criteria “QUAL < 30.0 || QD < 2.0 || FS > 60.0 || MQ < 40.0 || MQRankSum < −12.5 || ReadPosRankSum < −8.0 || SOR > 3.0.” For each dataset, lower and upper depth thresholds were set to approximately one-third and three times the mean sequencing depth, respectively. Only high-quality SNPs passing these filters were retained for downstream population genomic analyses.

For interspecific population genomic analyses, clean reads from all individuals were additionally mapped to the PG reference genome to provide a common genomic coordinate system across species. Pairwise relatedness was evaluated using KING (v.2.2.8) (Manichaikul et al. 2010). No first-degree relationships were detected, whereas several second- and third-degree relationships were identified within PG. Related individuals were subsequently excluded from downstream analyses sensitive to relatedness to minimize potential bias arising from nonindependence among samples (Table S14).

### Population structure analysis

To investigate population structure, we analyzed both a combined dataset including all three *Procapra* species and species-specific datasets. SNPs were filtered using VCFtools (v.0.1.13) (Danecek et al. 2011) with “--maf 0.05 --max-missing 0.95.” LD pruning was then performed using PLINK (Purcell et al. 2007) with “--indep-pairwise 50 10 0.2,” corresponding to a 50-SNP sliding window, a 10-SNP step size, and an *r*^2^ threshold of 0.2. The resulting LD-pruned SNP datasets were used for PCA with smartpca in EIGENSOFT (Patterson et al. 2006).

Genetic ancestry patterns were further evaluated using ADMIXTURE (v.1.3.0) (Alexander et al. 2009). For the combined three-species dataset, *K* = 1 to 10 was evaluated, with 100 independent replicates performed for each *K* using different random seeds. Cross-validation errors and log-likelihoods were summarized across replicates to assess model fit and convergence, and the replicate with the highest log-likelihood for each *K* was retained as the representative solution. Within-species ADMIXTURE analyses were also performed to explore possible substructure, with *K* = 1 to 5 evaluated for MG and TG and *K* = 1 to 10 for PG.

We also reconstructed genetic relationships using NJ trees based on autosomal and Y-chromosomal SNPs, as well as a ML tree based on mitochondrial genomes. Pairwise genetic distances for autosomal and Y-chromosomal SNPs were calculated using PLINK with the “--distance-matrix” option, and the resulting matrices were converted to MEGA format. NJ trees were reconstructed using MEGA (Kumar et al. 2018), with *G. subgutturosa* designated as the outgroup. Mitochondrial genomes were assembled for each individual using MitoZ (v.3.6) (Meng et al. 2019), and the mitochondrial ML tree was reconstructed using MEGA with default settings.

Moreover, we calculated pairwise *F*_ST_ values among the three *Procapra* species using VCFtools with the parameters “--fst-window-size 50000 --fst-window-step 25000 --maf 0.05 --max-missing 0.95” to quantify genetic differentiation.

### Population demographic history

To characterize demographic changes across different timescales, we integrated several complementary approaches. Long-term demographic history was inferred using the PSMC model (Li and Durbin 2011). PSMC analyses were conducted with the parameters “-N25 -t15 -r5 -p 4 + 25*2 + 4 + 6,” with 100 bootstrap replicates used to assess the stability of effective population size (*N*_e_) estimates. A generation time (*g*) of 3 years and a mutation rate (*μ*) of 0.9 × 10^−8^ per site per generation were assumed (Leslie 2010; Chen et al. 2019).

To infer more recent demographic changes, genotype phasing was performed using Beagle v4.1 (Browning and Browning 2007) with default parameters. Phased data with repetitive regions masked were analyzed using SMC++ (Terhorst et al. 2017). Filtered VCF files were converted using vcf2smc, followed by demographic inference with the estimate command. The same mutation rate and generation time used for PSMC were applied. We further used PopSizeABC (Boitard et al. 2016) to reconstruct recent changes in *N*_e_. Minor allele count thresholds for the folded allele-frequency spectrum, identity-by-state statistics, and LD statistics were set to 20% of the haploid sample size, and 29 independent genomic segments were used to represent the autosomes.

To further resolve very recent demographic changes over the most recent 150 generations, we applied GONE2 (Santiago et al. 2025) using its structure-aware metapopulation framework. Autosomal biallelic SNPs from the species-specific datasets were used after excluding related individuals, resulting in 32 MG, 43 PG, and 12 TG individuals. Analyses were performed across 30 replicate runs, each based on 10,000 randomly sampled SNPs, assuming a recombination rate of 1 cM/Mb. The median trajectory of total metapopulation size (*N*_T_) across replicates was used to summarize recent population size changes, with empirical 50% and 95% intervals used to characterize uncertainty.

### Gene flow analysis

To investigate historical gene flow among the three *Procapra* species, we computed *D*-statistics using the qpDstat program in ADMIXTOOLS2 (Maier et al. 2023), with *G. subgutturosa* as the outgroup and standard errors estimated using a block jackknife (blgsize = 0.05). To assess whether the signals were widespread or driven by particular individuals, we additionally performed individual-level *D*-statistic tests of the form (PG_i, MG; TG_j, Outgroup) to quantify allele sharing between PG and TG individuals. Relative MG-related allele sharing among PG individuals was further evaluated using comparisons of the form (PG_i, PG_k; MG, Outgroup). We also applied Dsuite (Malinsky et al. 2021) to characterize genome-wide patterns of allele sharing among the three *Procapra* species, with *f*-branch statistics used to summarize branch-specific signals of excess allele sharing.

Given that TG and PG remain partially sympatric, we additionally assessed evidence of recent admixture using the apoh approach (Garcia-Erill et al. 2023). This method identifies hybrid pedigrees within the past few generations. We used ADMIXTURE-derived ancestry information in NGSremix (Nøhr et al. 2021) with the parameters “-bothanc 1 -boot 100 -block 1000.” The resulting paired ancestry estimates were then analyzed using apoh to evaluate recent admixture pedigrees.

### Species distribution modeling

Occurrence records for each *Procapra* species were compiled from field surveys and the Global Biodiversity Information Facility (GBIF; https://www.gbif.org/). We initially obtained 83, 205, and 137 records for MG, PG, and TG, respectively. Records with obvious geographic errors, duplicates, or inconsistent locality information were removed. To reduce spatial sampling bias and model overfitting, occurrence records were spatially thinned at a 5-km resolution, resulting in 58 MG, 43 PG, and 71 TG records retained for modeling.

Nineteen bioclimatic variables representing the current period, LGM, and LIG were obtained from WorldClim at 2.5-arc-min resolution (http://www.worldclim.org/). Environmental layers were clipped to the study region and aligned to a common spatial grid. To reduce multicollinearity, Pearson correlations among variables were calculated, and one variable from each highly correlated pair (|*r*| > 0.8) was removed (Table S19). The same selected variables were used across climatic periods. MG and TG models included BIO1 (annual mean temperature), BIO2 (mean diurnal range), BIO12 (annual precipitation), and BIO15 (precipitation seasonality), whereas PG models included BIO11 (mean temperature of the coldest quarter), BIO12, and BIO17 (precipitation of the driest quarter).

Model tuning was performed using the R package ENMeval (Muscarella et al. 2014) by testing combinations of linear (L), quadratic (Q), hinge (H), and product (P) feature classes (LQ, LQH, and LQHP) and regularization multipliers (1, 2, and 3) (Table S20). The optimal parameter combination was selected based on the lowest corrected Akaike information criterion. The LQH feature class was selected for all three species, with regularization multipliers of 1 for MG and 3 for PG and TG. Final species distribution models were generated using MaxEnt v.3.4.3 (Phillips et al. 2006), with 75% of occurrence records used for training and 25% for testing. Logistic outputs were used as habitat suitability indices, and suitability distributions under current, LGM, and LIG climatic conditions were visualized using ArcMap v.10.8 (Table S21).

### Genetic diversity, LD, and inbreeding estimates

To assess genetic diversity and inbreeding across the three *Procapra* species, we used VCF datasets generated by mapping reads to the corresponding species-specific reference genomes. Genome-wide heterozygosity was estimated from PLINK --het outputs as the proportion of heterozygous sites for each individual. Nucleotide diversity (π) was calculated using VCFtools with --window-pi 50000 in 50-kb windows. Genome-wide LD decay was estimated from unpruned SNP datasets using PopLDdecay (Zhang et al. 2019) with default parameters.

ROHs were identified using PLINK with the parameters --homozyg-window-missing 20 --homozyg-window-snp 20 --homozyg-kb 10. ROH segments shorter than 100 kb were excluded, and the remaining segments were classified into four length categories: 100 to 500, 500 to 1,000, 1,000 to 3,000, and >3,000 kb. These length classes provide information on the relative timing of inbreeding, as longer ROHs generally reflect more recent parental relatedness. The genomic inbreeding coefficient (*F*_ROH_) was calculated as the total length of ROHs divided by the total length of the 29 autosomes. To illustrate chromosome-wide ROH distributions, we visualized ROH patterns for six representative individuals covering all three species: MY10 and MY38 from MG, PY27 and PY58 from PG, and ZY17 and ZY20 from TG, following the approach described previously (Liu et al. 2024) (Figs. S19 to S21).

### Detection of introgression regions and heterozygosity analysis

Following the gene flow analyses, we detected consistent signals of excess allele sharing between PG and TG. We therefore examined whether PG genetic diversity may be partly associated with historical allele sharing with TG. To localize putative introgression regions in PG, we used the “Dinvestigate” module in Dsuite software, which computes *D*, *f_d_*, and *f_dM_* in sliding windows across the genome (Malinsky et al. 2015; Malinsky et al. 2021). For each PG individual (PG_i), we specified trios of the form (MG, PG_i, TG) and performed sliding window scans using “-w 50,25.” Candidate windows were retained if they showed *f_dM_* > 0 and fell within the top 5% of the genome-wide *f_dM_* distribution for that individual. Adjacent or overlapping candidate windows were merged into regions, and to reduce noise from isolated outliers, we required each inferred region to contain at least three consecutive windows. This procedure yielded a set of individual-specific putative introgression regions (Table S25). We then calculated heterozygosity in these putative introgression regions and the corresponding background regions.

### Genetic load assessment and functional enrichment analyses

To assess putative genetic load across the three *Procapra* species, we used species-specific VCF datasets generated by mapping sequencing reads to the corresponding reference genomes to minimize reference bias. Ancestral and derived allele states were inferred based on comparison with the outgroup genotype, and variants were polarized accordingly before genetic load analyses. Variants within annotated coding sequences were functionally annotated using SnpEff (Cingolani et al. 2012) and classified as LOF, missense, or SYN variants. Missense variants were further classified as DEL or TOL using SIFT4G (Vaser et al. 2016). For each individual, we quantified heterozygous and homozygous-derived variants in each functional category. Heterozygous LOF and DEL variant counts were additionally normalized separately by the total number of genome-wide heterozygous SNPs and by the corresponding number of heterozygous SYN variants in each individual. The proportion of derived alleles carried in the homozygous state was calculated as 2 × homozygous/(2 × homozygous + heterozygous), allowing standardized comparisons among species.

We calculated derived LOF/SYN and DEL/SYN allele count ratios separately for ROH and non-ROH regions in each individual (Xue et al. 2015). Non-ROH regions were defined as analyzed regions outside identified ROHs, and heterozygous and homozygous-derived genotypes contributed one and two derived allele copies, respectively.

To further investigate the potential functional consequences of putatively deleterious variation, we identified genes carrying LOF and DEL variants retained after MAF ≥0.05 filtering in each species. Candidate genes were functionally annotated using eggNOG-mapper v.2.1.15 (Cantalapiedra et al. 2021) with the eggNOG v.5.0.2 database (Huerta-Cepas et al. 2019). GO Biological Process (The Gene Ontology Consortium 2026) and KEGG (Kanehisa et al. 2025) enrichment analyses were performed separately for MG, PG, and TG using species-specific annotations and corresponding annotated gene sets as backgrounds. Enrichment was assessed using a hypergeometric test with Benjamini–Hochberg correction for multiple testing, and adjusted *P* values were used to evaluate statistical significance.

## Supplementary Material

msag258_Supplementary_Data

## Data Availability

The sequencing data generated in this study have been deposited in the National Genomics Data Center, China National Center for Bioinformation/Beijing Institute of Genomics, Chinese Academy of Sciences, under BioProject accession PRJCA042468. PacBio HiFi and Hi-C data used for reference genome assembly are available in the Genome Sequence Archive (GSA) under accession CRA027485, and whole-genome resequencing data are available under accession CRA027702 (https://ngdc.cncb.ac.cn/gsa) (Chen et al. 2021; CNCB-NGDC Members and Partners 2025). The assembled genomes of the three *Procapra* species have been deposited in the Genome Warehouse under accession numbers GWHGEPC00000000.1, GWHGEPD00000000.1, and GWHGEPE00000000.1 (https://ngdc.cncb.ac.cn/gwh) (CNCB-NGDC Members and Partners 2025; Ma et al. 2025).
