# Supplementary material for: Comparative population genomics reveals evolutionary potential and conservation priorities in three Procapra species
Source: Mol Biol Evol. 2026 Oct 7;43(10):msag258. doi: 10.1093/molbev/msag258 (PMC13685806; doi:10.1093/molbev/msag258)
Supplement: Supplementary file 1 [file msag258_supplementary_data.pdf]

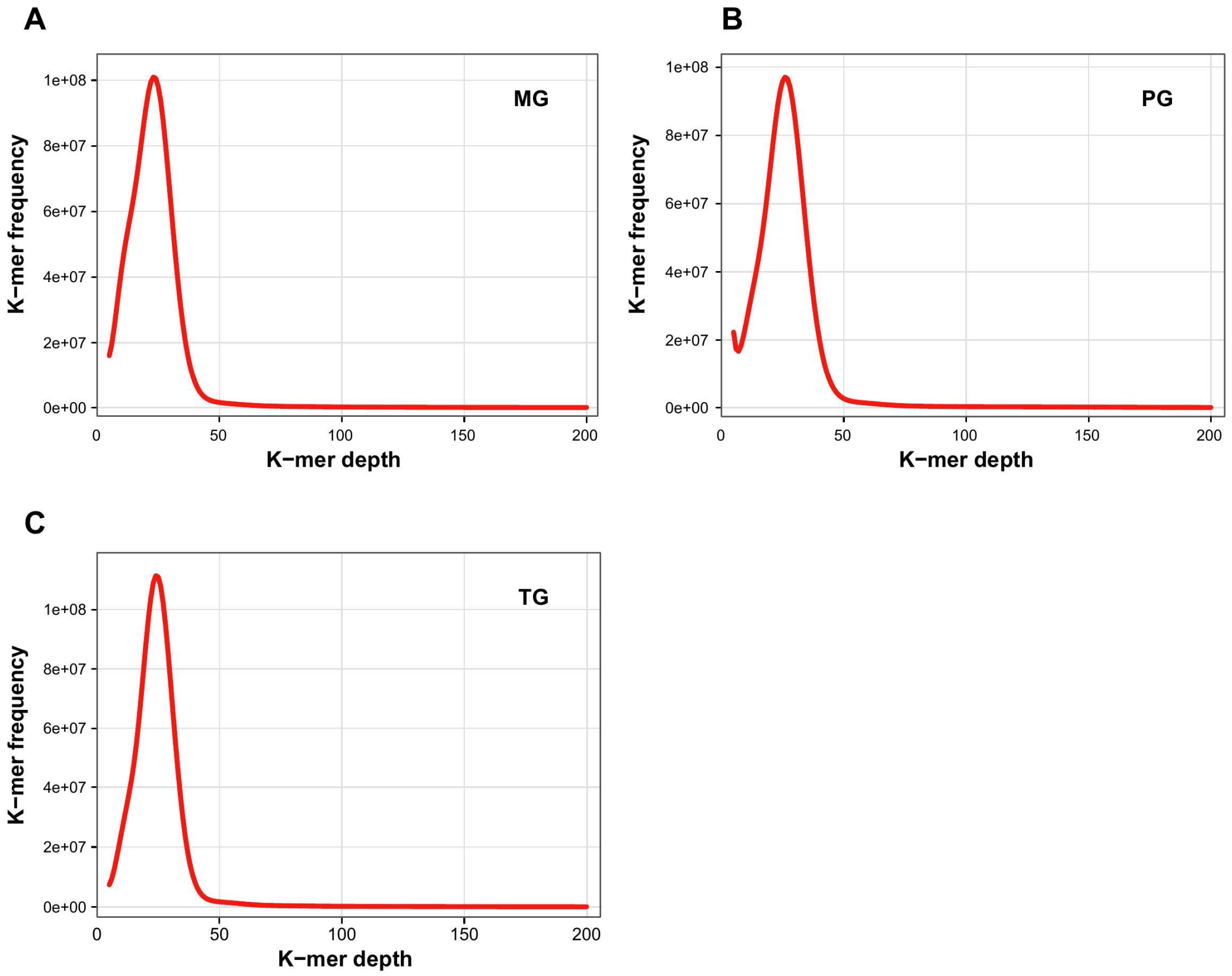

Figure S1. K-mer frequency distributions for the three *Procapra* species. K-mer frequency distributions were generated from clean paired-end sequencing reads using a k-mer length of 21 for (A) Mongolian gazelle (*Procapra gutturosa*, MG), (B) Przewalski's gazelle (*Procapra przewalskii*, PG), and (C) Tibetan gazelle (*Procapra picticaudata*, TG). The x-axis represents k-mer depth, and the y-axis represents k-mer frequency. The major peaks were used for genome survey and genome-size estimation.

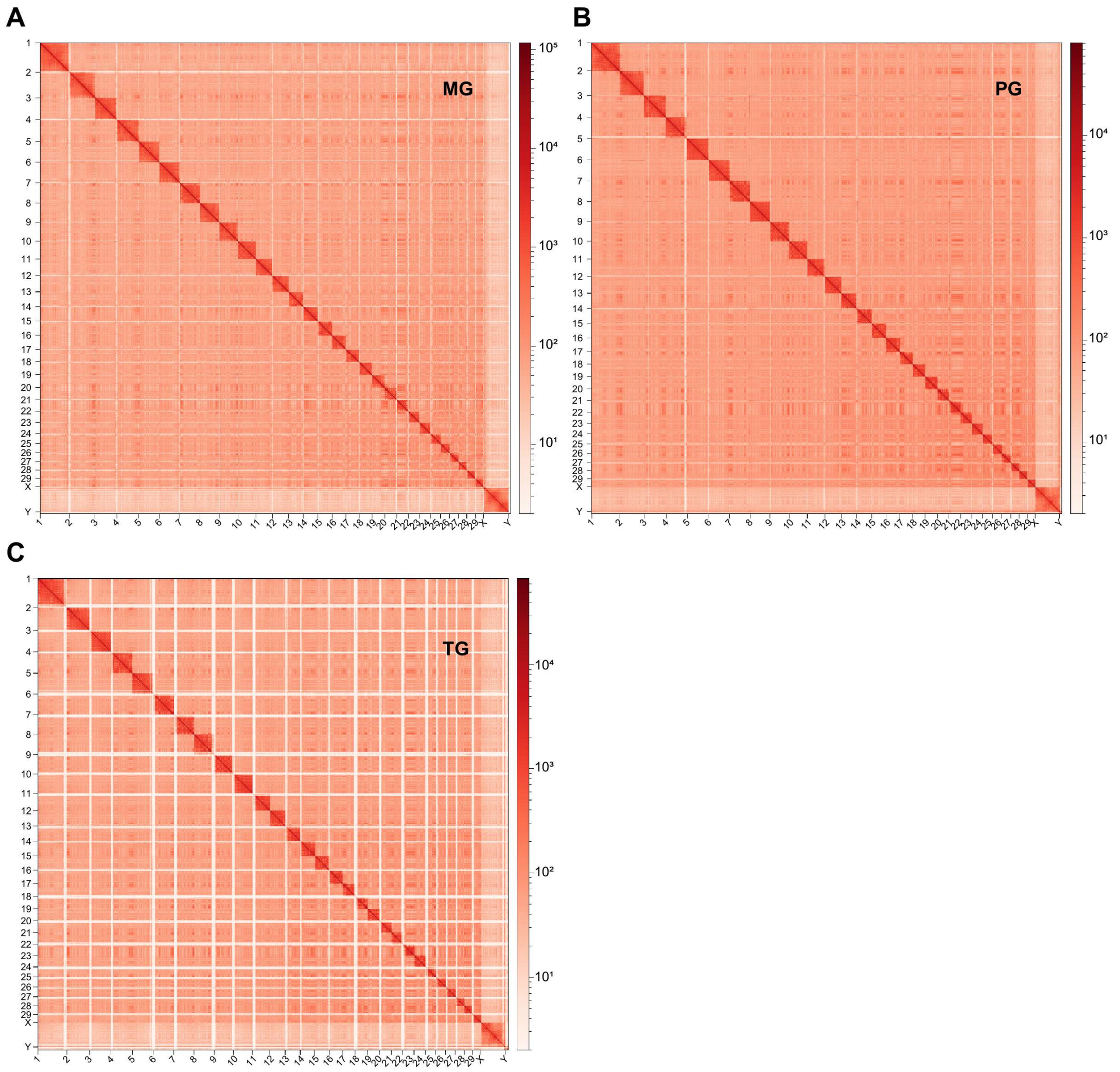

Figure S2. Genome-wide Hi-C contact maps for MG, PG, and TG. Hi-C interaction matrices are shown for (A) MG, (B) PG, and (C) TG based on the final curated chromosome-level genome assemblies. Contact maps were plotted at 1-Mb resolution using HiCExplorer with log-transformed contact counts. Chromosomes are ordered and labeled along both axes, with chromosome boundaries indicated by the matrix blocks. Darker red represents stronger Hi-C interaction signals.

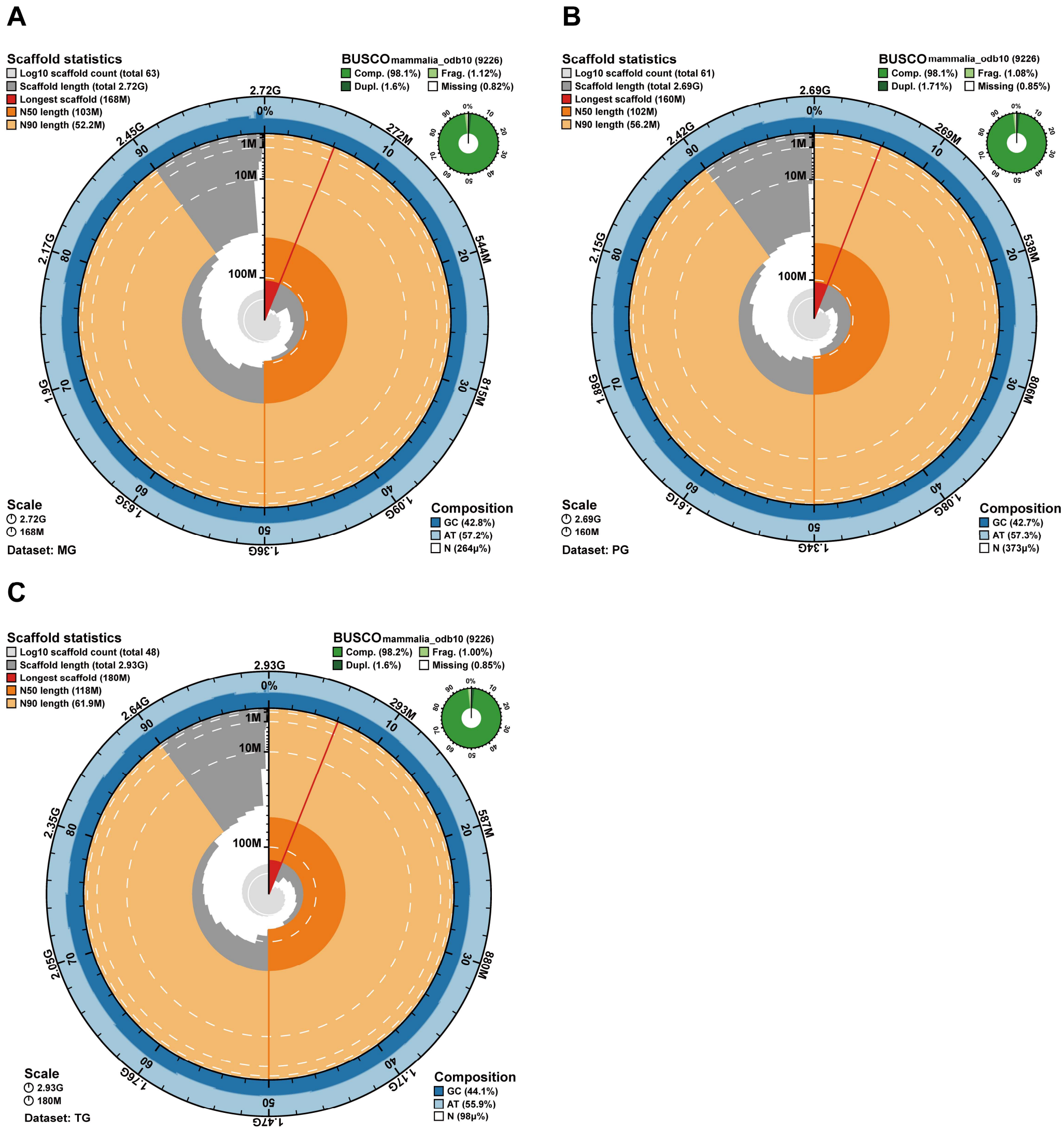

Figure S3. Snail plots summarizing assembly statistics for the three *Procapra* genome assemblies. Snail plots are shown for (A) MG, (B) PG, and (C) TG. Each plot summarizes chromosome-scale assembly statistics, including scaffold number and length distribution, cumulative assembly size, N50 and N90 values, nucleotide composition, and BUSCO completeness. These metrics collectively summarize assembly contiguity, sequence composition, and gene-space completeness.

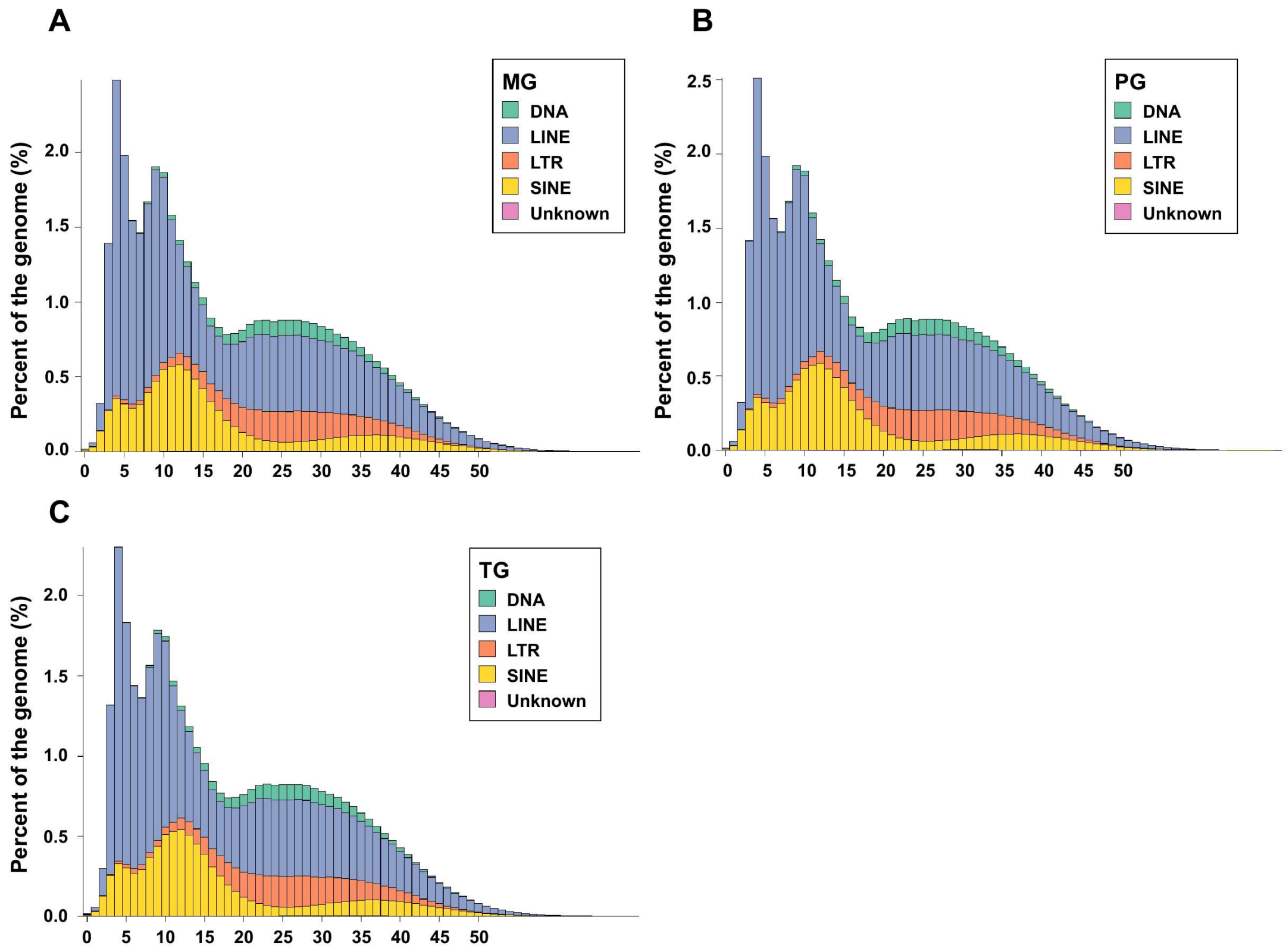

Figure S4. Sequence-divergence landscapes of transposable elements in the three *Procapra* genome assemblies. Sequence-divergence landscapes of annotated transposable elements are shown for (A) MG, (B) PG, and (C) TG. The x-axis represents sequence divergence from the corresponding repeat consensus sequences, and the y-axis represents the percentage of the genome occupied by each transposable element class within each divergence bin. Transposable elements are classified into DNA transposons, LINEs, LTRs, SINEs and unknown repeats.

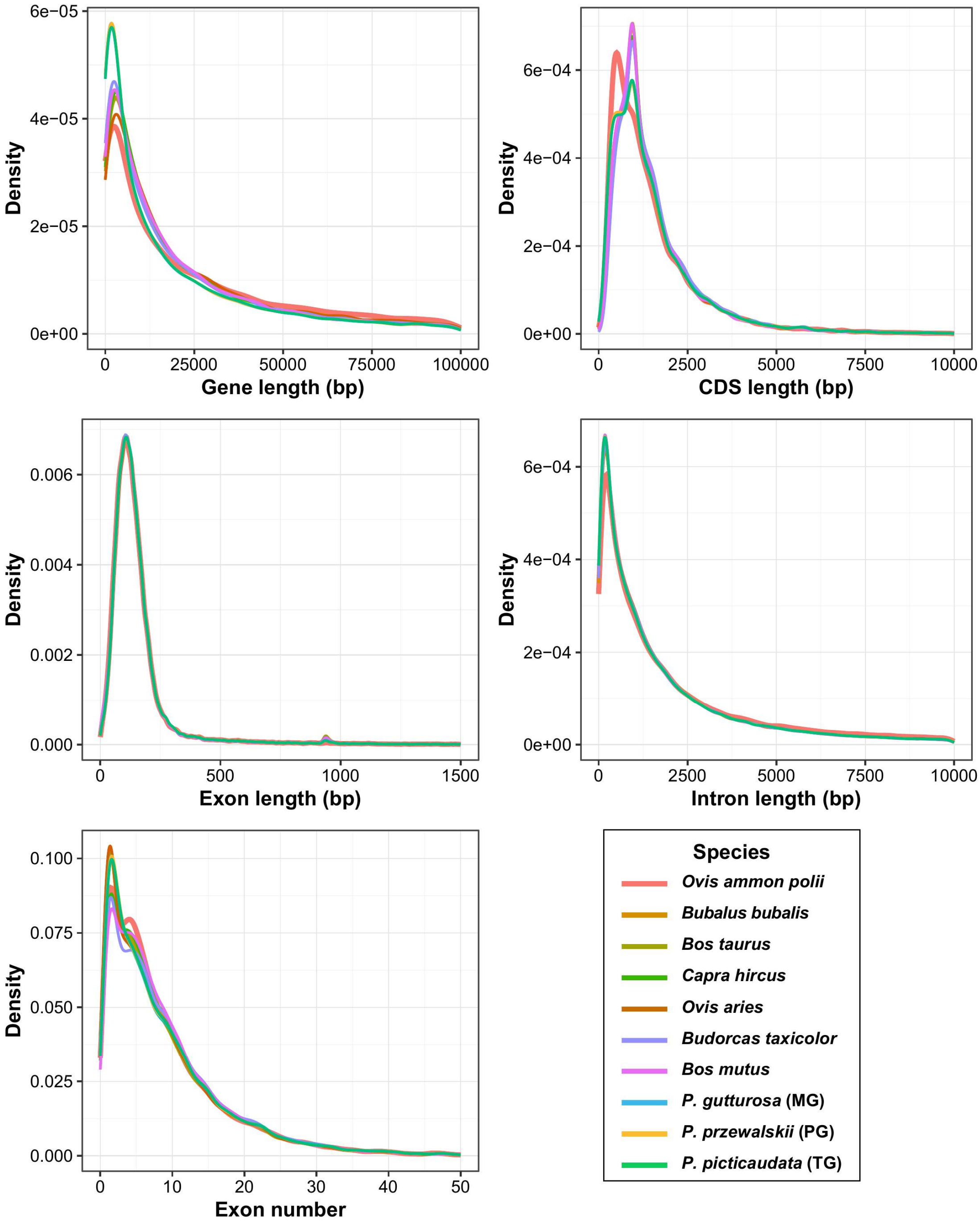

Figure S5. Comparison of gene-structure features between the three *Procapra* genome annotations and other representative ruminant species. Kernel density distributions are shown for gene length, CDS length, exon length, intron length and exon number. The three *Procapra* species show broadly similar distributions to those of other representative ruminants, supporting the overall consistency of their gene annotations. Different colors represent different species as indicated in the legend.

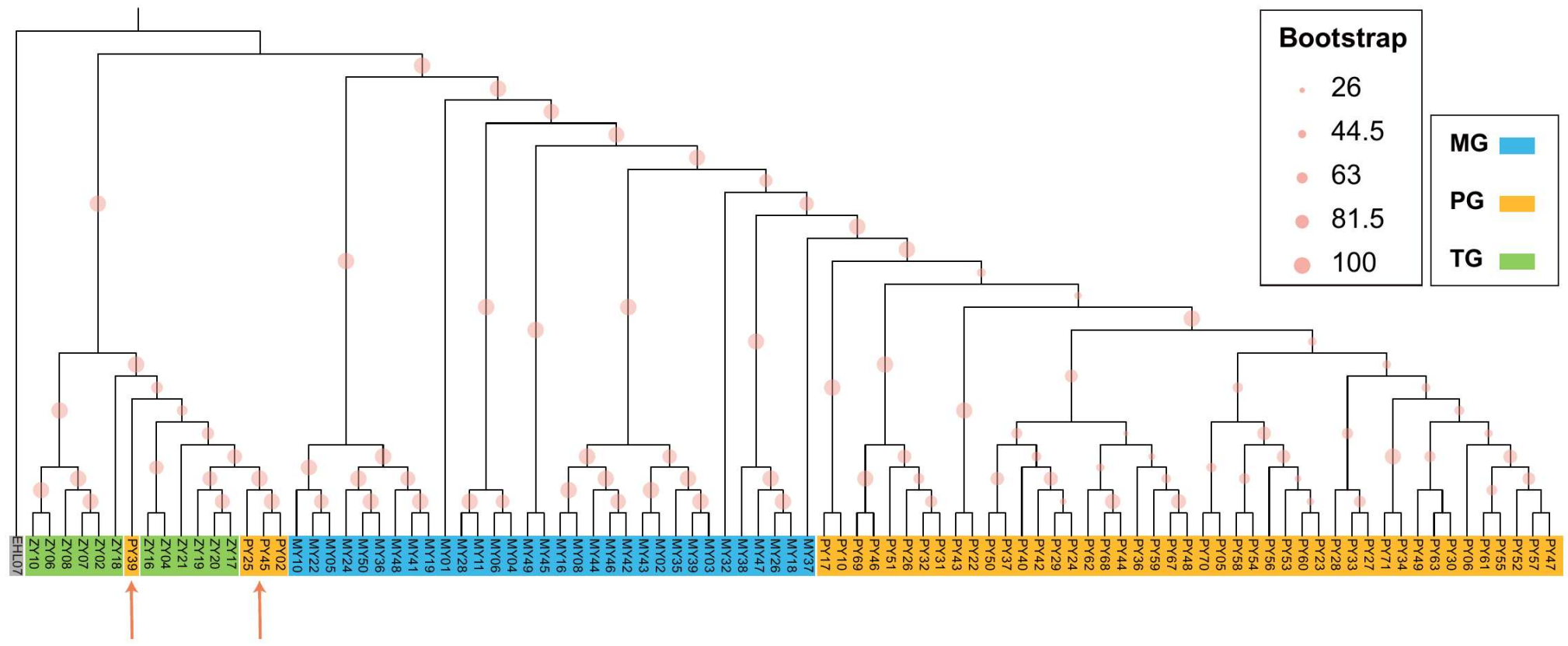

Figure S6. Maximum-likelihood phylogeny based on mitochondrial genome sequences of 93 *Procapra* individuals. The mitochondrial genome tree was rooted using EHL07 as the outgroup. Tip colors indicate species assignment, and circle size at internal nodes is proportional to bootstrap support. Arrows indicate PG individuals that cluster within or close to TG mitochondrial lineages, suggesting mitochondrial phylogenetic discordance that may reflect historical introgression.

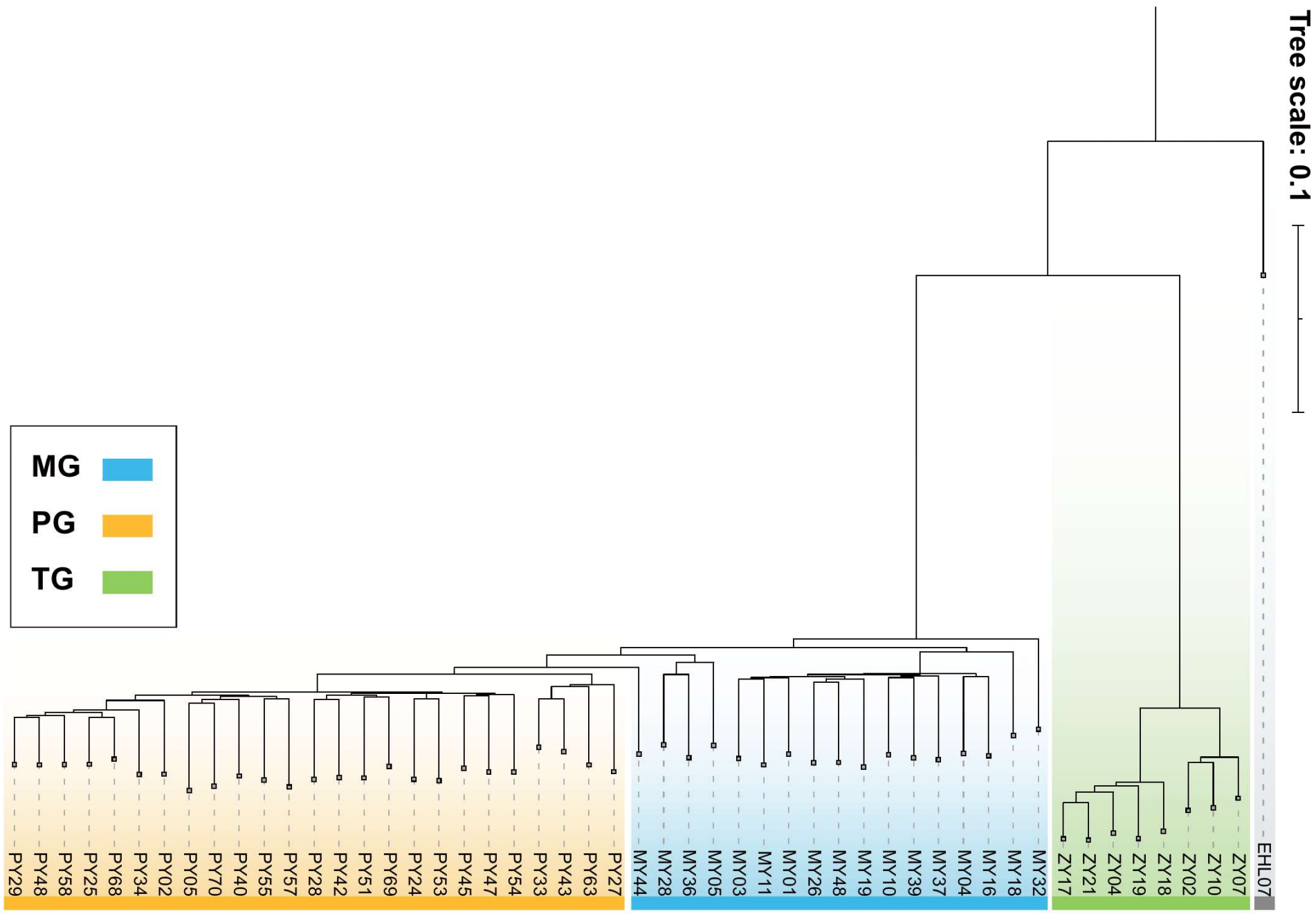

Figure S7. Neighbor-joining phylogeny based on Y-chromosomal SNPs from male *Procapra* individuals. The tree was constructed from Y-chromosomal SNPs and rooted using EHL07 as the outgroup. Tip colors indicate species assignment. The Y-chromosomal phylogeny largely separates MG, PG and TG into species-specific clusters, supporting differentiation of paternal lineages among the three species.

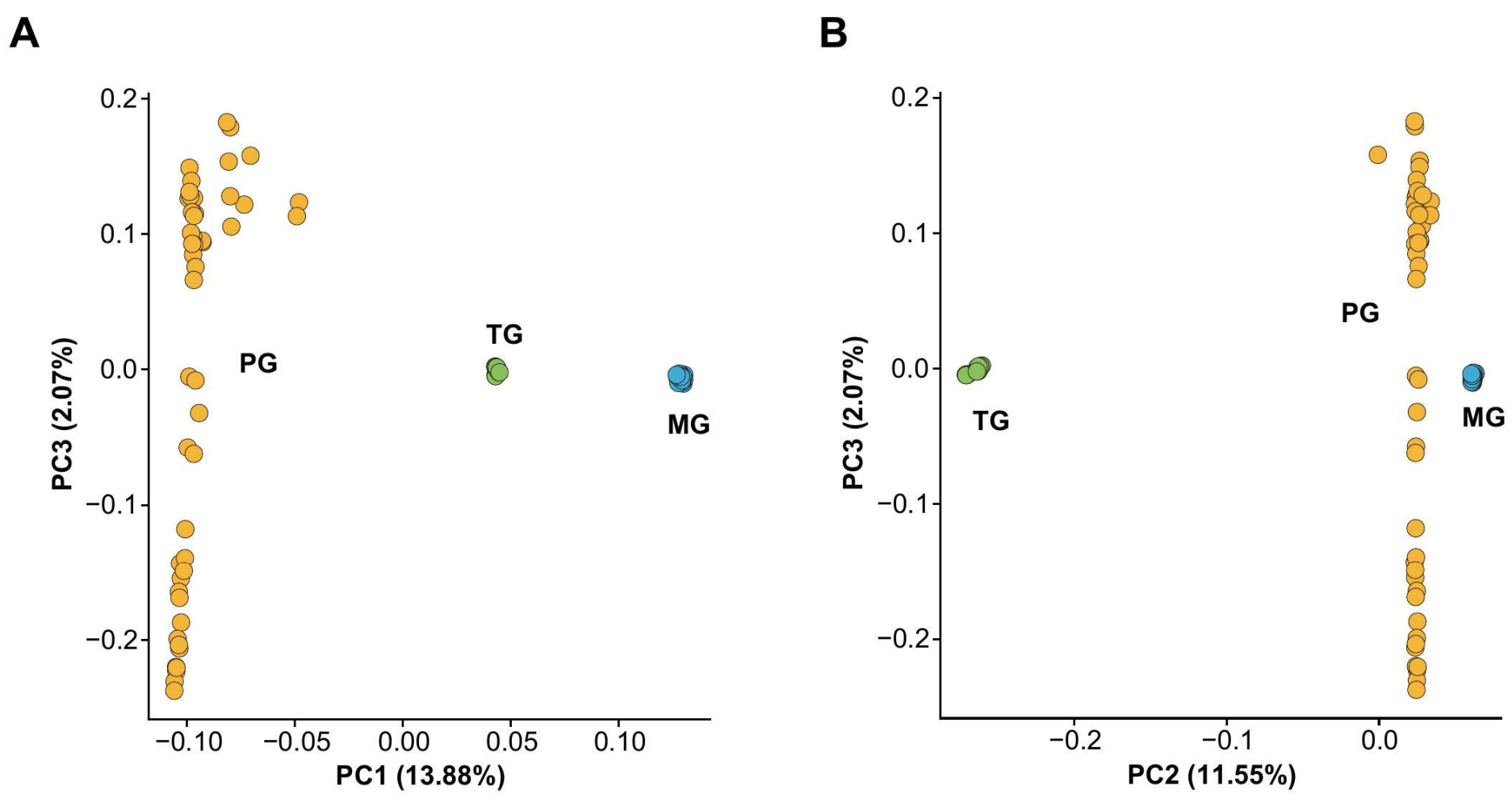

Figure S8. Additional principal component analysis of MG, PG, and TG. PCA results are shown for (A) PC1 versus PC3 and (B) PC2 versus PC3. Each point represents one individual, with MG, PG, and TG shown in blue, orange, and green, respectively. The percentages on the axes indicate the proportion of genetic variation explained by each principal component.

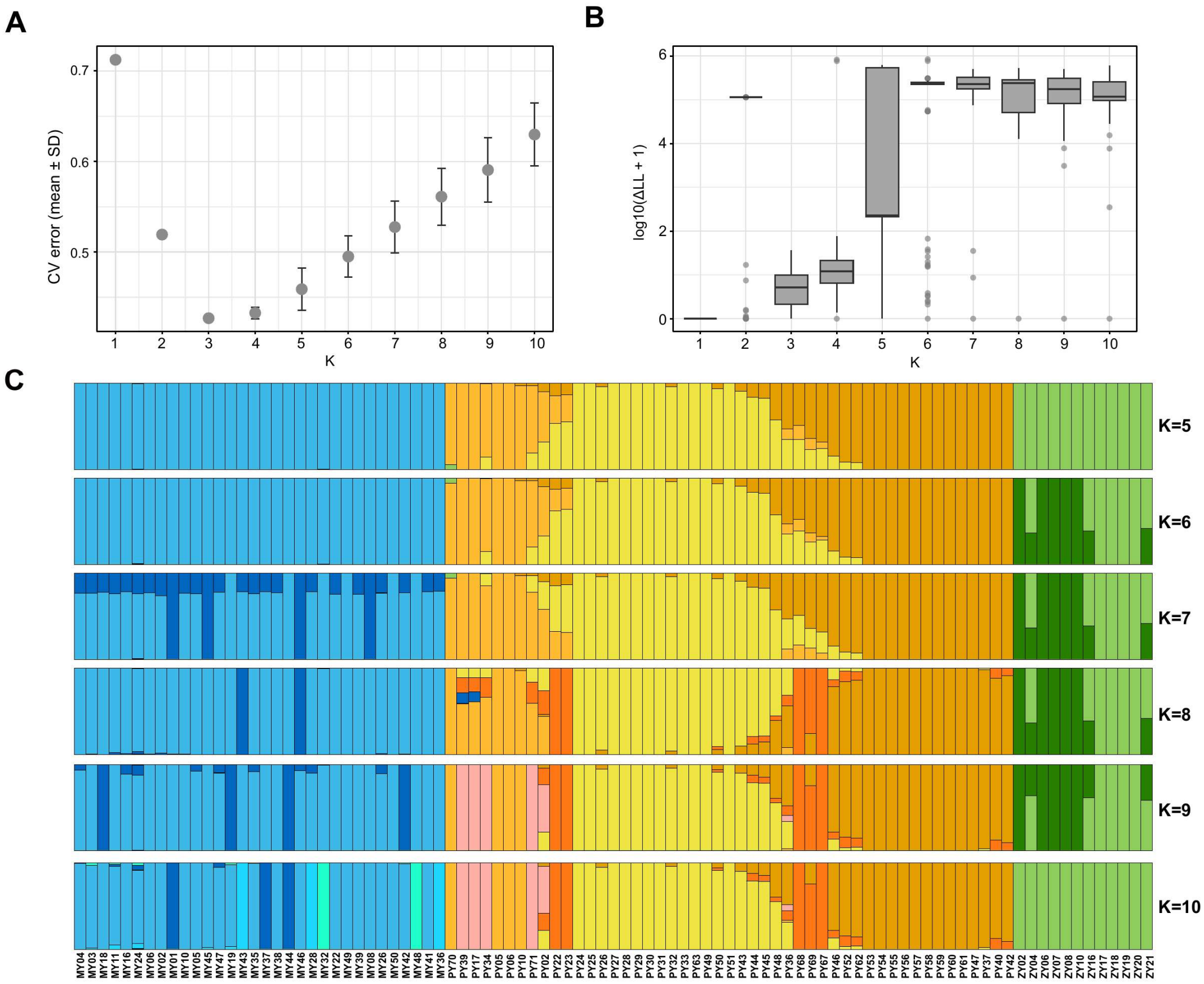

Figure S9. ADMIXTURE model-choice diagnostics and ancestry profiles for the three *Procapra* species. (A) Cross-validation (CV) error for *K* = 1–10. For each *K*, ADMIXTURE was run 100 times using different random seeds, and values are shown as mean ± SD. (B) Distribution of log-likelihood differences across the 100 replicate runs for each *K*. For each *K*, ΔLL was calculated as $LL_{max} - LL_i$ for replicate run i, where LL_{max} is the highest log-likelihood among the 100 runs at that *K*. Values were plotted as $\log_{10}(\Delta LL + 1)$, so that the best run for each *K* was retained as zero. Smaller values indicate more consistent convergence across random initializations, whereas larger values indicate less stable solutions or potential local optima. (C) ADMIXTURE ancestry profiles for *K* = 5–10. Each vertical bar represents one individual, grouped by species. Colors indicate inferred ancestry components.

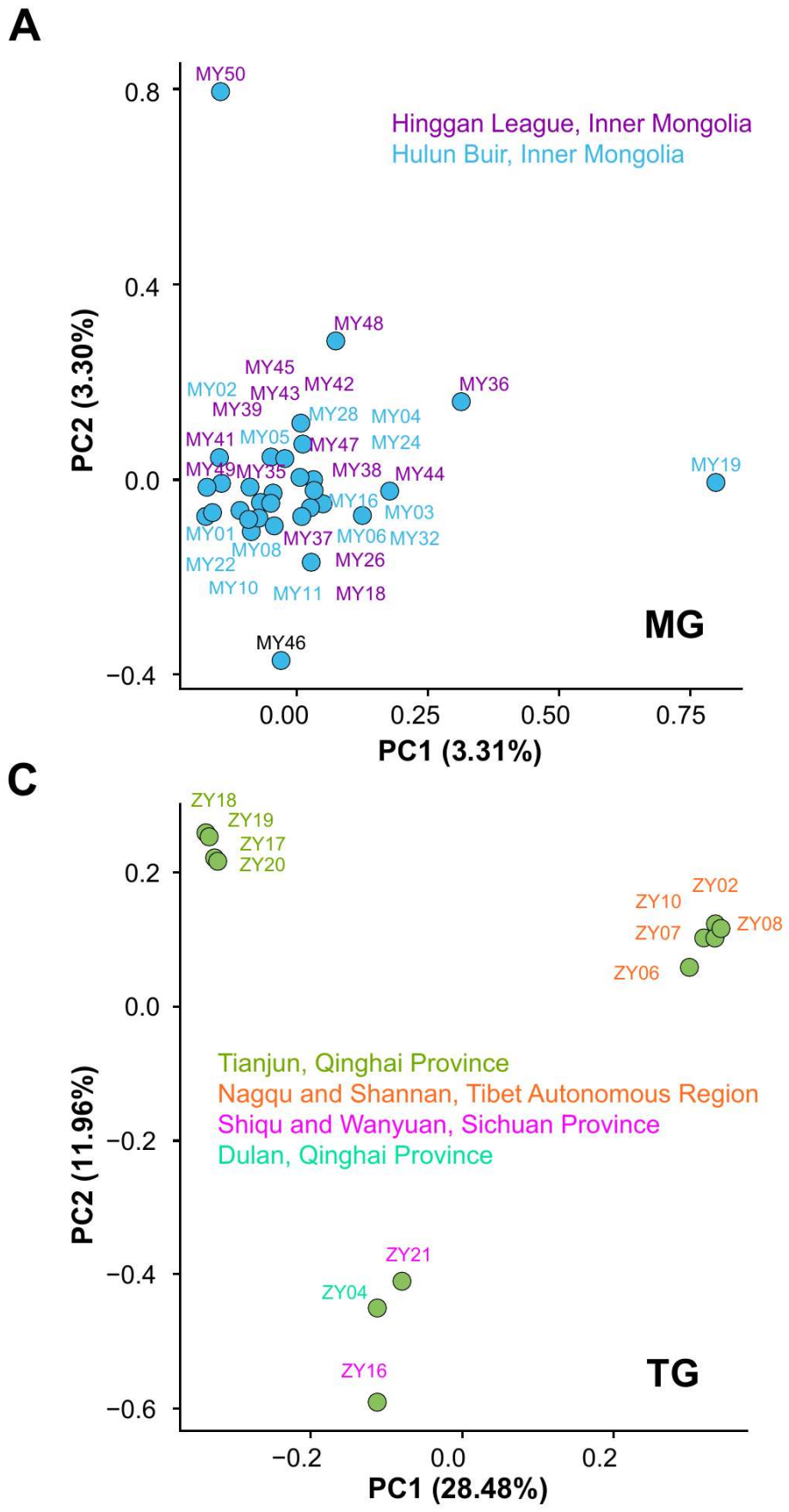

Figure S10. Within-species principal component analysis of genomic variation in the three *Procapra* species. Principal component analysis was performed separately for (A) MG, (B) PG and (C) TG. Each point represents one individual. Axes show the first two principal components, with the percentage of variance explained by each component indicated in parentheses. Individual labels are colored according to sampling region.

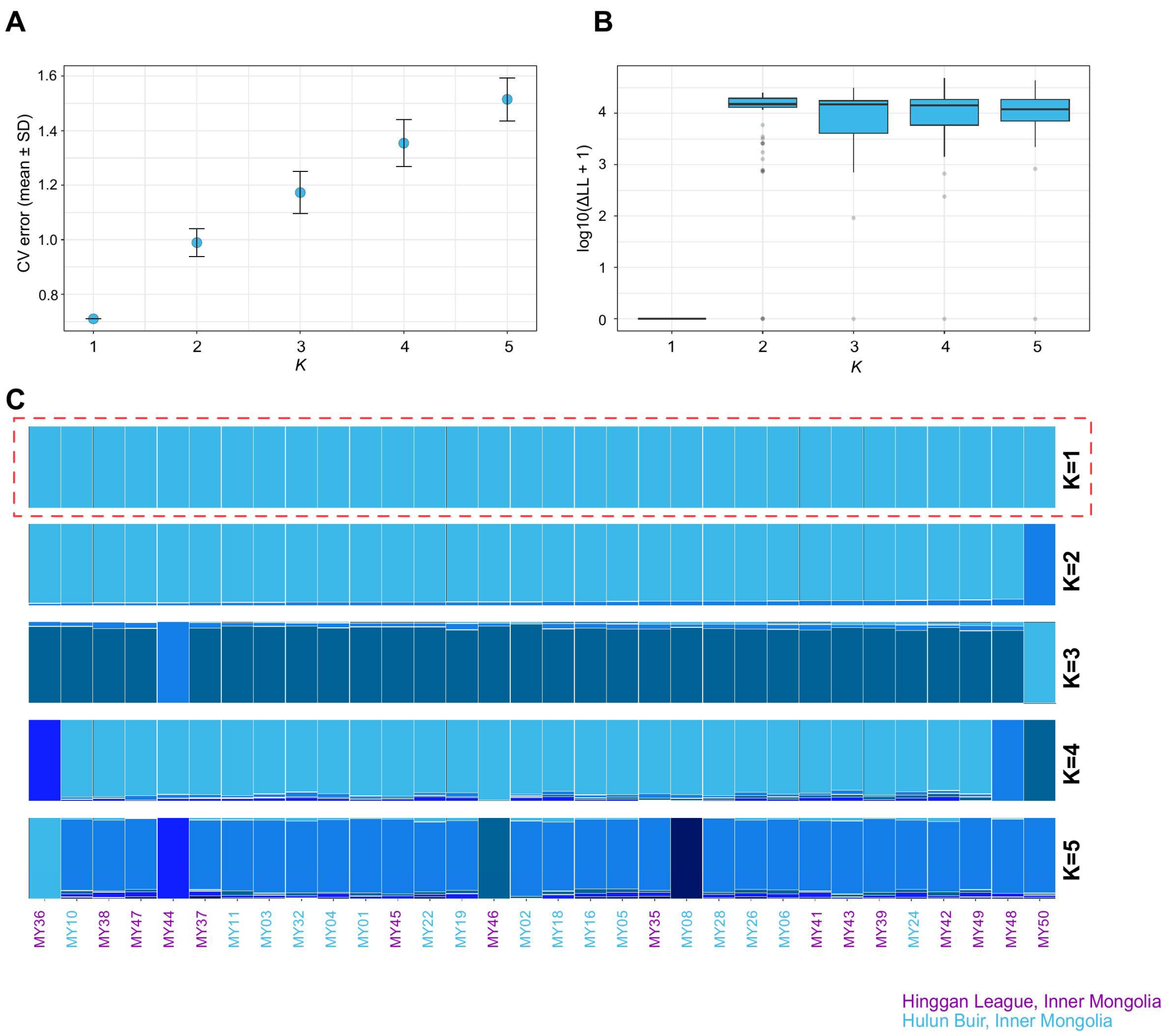

Figure S11. ADMIXTURE model-choice diagnostics and ancestry profiles for MG. (A) Cross-validation (CV) error for $K = 1–5$. For each K, ADMIXTURE was run 100 times using different random seeds, and values are shown as mean ± SD. (B) Distribution of log-likelihood differences across the 100 replicate runs for each K. For each K, ΔLL was calculated as $LL_{max} - LL_i$ for replicate run i, where LL_{max} is the highest log-likelihood among the 100 runs at that K. Values were plotted as $\log_{10}(\Delta LL + 1)$, so that the best run for each K was retained as zero. Smaller values indicate more consistent convergence across random initializations, whereas larger values indicate less stable solutions or potential local optima. (C) ADMIXTURE ancestry profiles for $K = 1–5$. Each vertical bar represents one individual, and colors indicate inferred ancestry components. The red dashed box highlights the $K = 1$ model, which had the lowest CV error. Individual labels are colored according to sampling region.

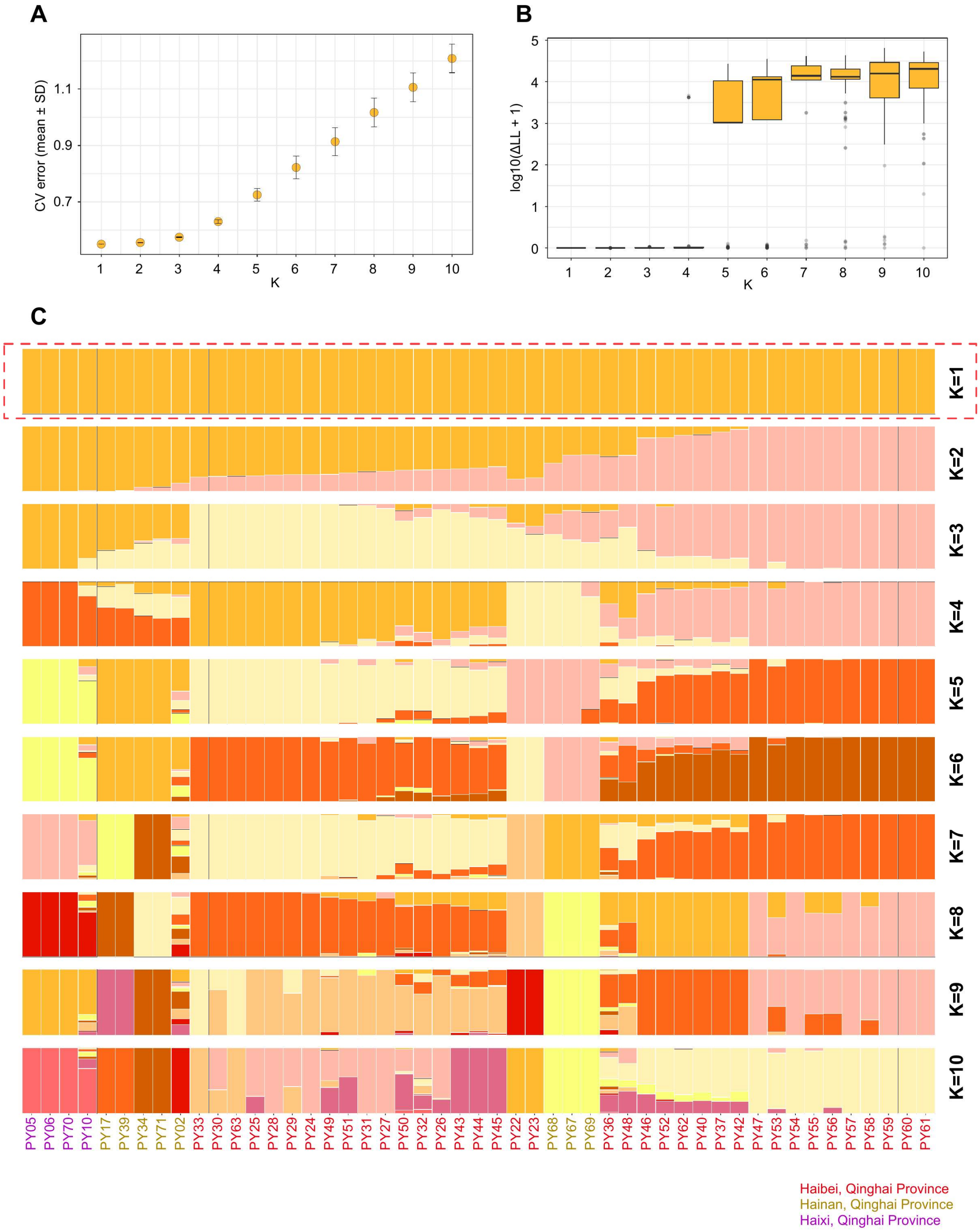

Figure S12. ADMIXTURE model-choice diagnostics and ancestry profiles for PG. (A) Cross-validation (CV) error for $K = 1$–10. For each K, ADMIXTURE was run 100 times using different random seeds, and values are shown as mean ± SD. (B) Distribution of log-likelihood differences across the 100 replicate runs for each K. For each K, ΔLL was calculated as $LL_{max} - LL_i$ for replicate run i, where LL_{max} is the highest log-likelihood among the 100 runs at that K. Values were plotted as $\log_{10}(\Delta LL + 1)$, so that the best run for each K was retained as zero. Smaller values indicate more consistent convergence across

random initializations, whereas larger values indicate less stable solutions or potential local optima. (C) ADMIXTURE ancestry profiles for K = 1–10. Each vertical bar represents one individual, and colors indicate inferred ancestry components. The red dashed box highlights the K = 1 model, which had the lowest CV error. Individual labels are colored according to sampling region.

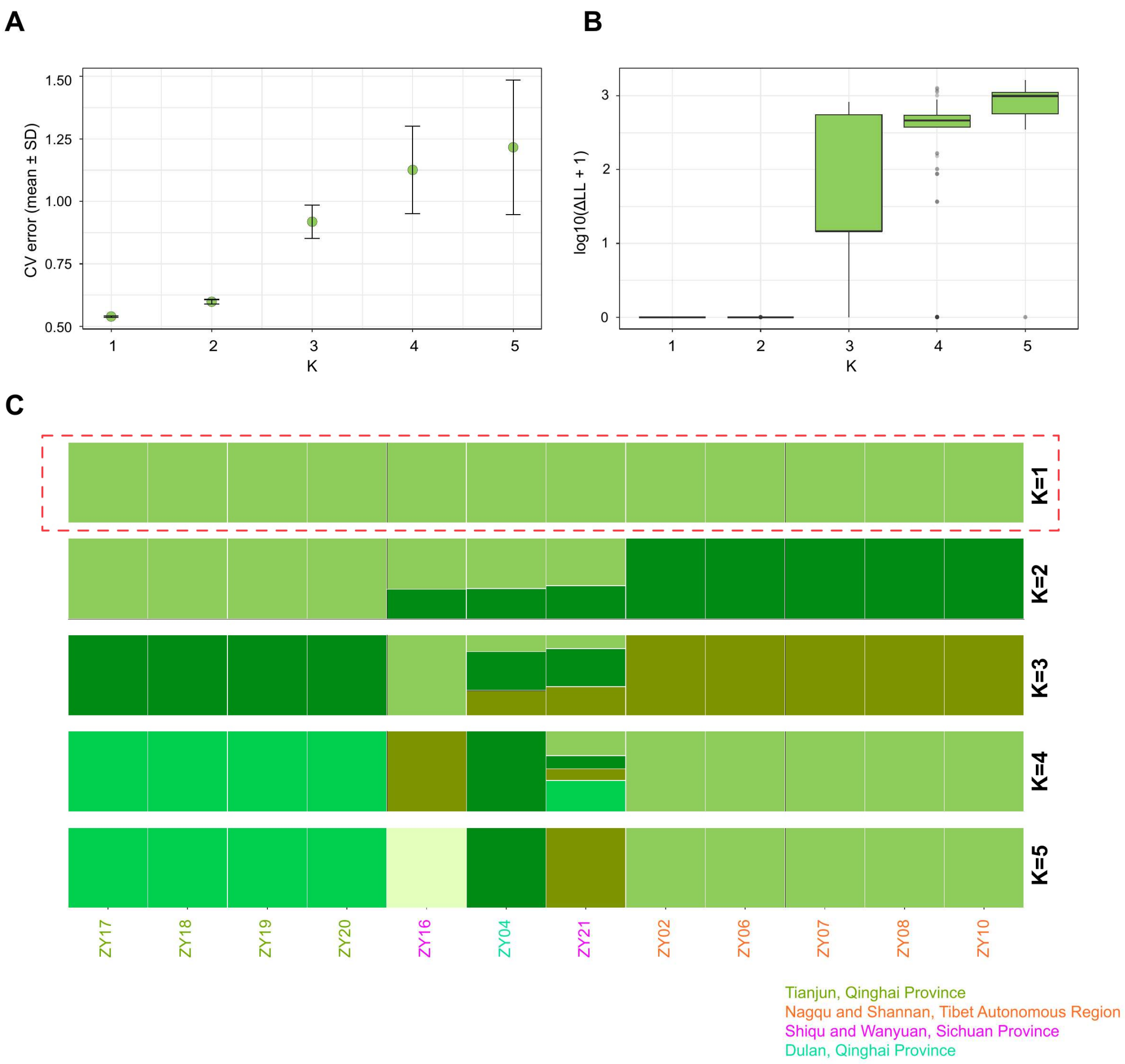

Figure S13. ADMIXTURE model-choice diagnostics and ancestry profiles for TG. (A) Cross-validation (CV) error for $K = 1–5$. For each K, ADMIXTURE was run 100 times using different random seeds, and values are shown as mean ± SD. (B) Distribution of log-likelihood differences across the 100 replicate runs for each K. For each K, ΔLL was calculated as $LL_{max} - LL_i$ for replicate run i, where LL_{max} is the highest log-likelihood among the 100 runs at that K. Values were plotted as $\log_{10}(\Delta LL + 1)$, so that the best run for each K was retained as zero. Smaller values indicate more consistent convergence across random initializations, whereas larger values indicate less stable solutions or potential local optima. (C) ADMIXTURE ancestry profiles for $K = 1–5$. Each vertical bar represents one individual, and colors indicate inferred ancestry components. The red dashed box highlights the $K = 1$ model, which had the lowest CV error. Individual labels are colored according to sampling region.

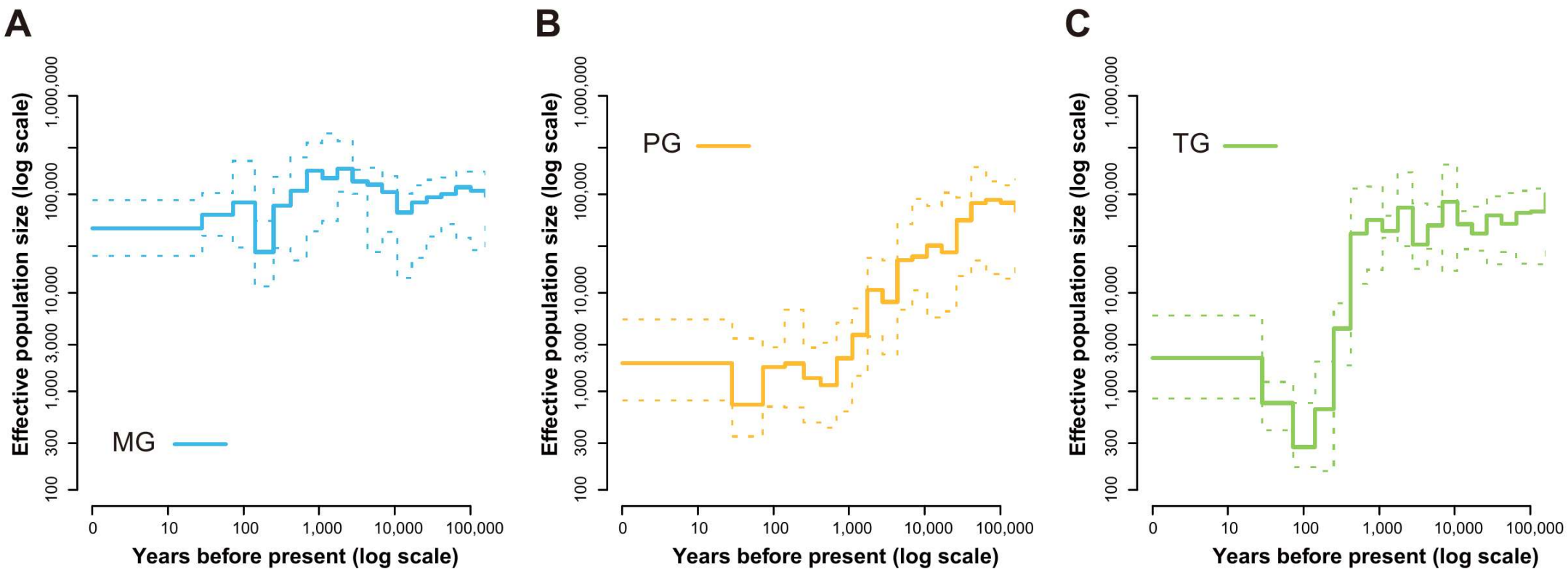

Figure S14. Historical effective population size trajectories inferred using PopSizeABC for the three *Procapra* species. PopSizeABC was used to infer changes in effective population size over time for (A) MG, (B) PG and (C) TG. The x-axis represents years before present on a log scale, and the y-axis represents effective population size on a log scale. Solid lines indicate the inferred effective population size trajectories, and dashed lines indicate the corresponding uncertainty intervals.

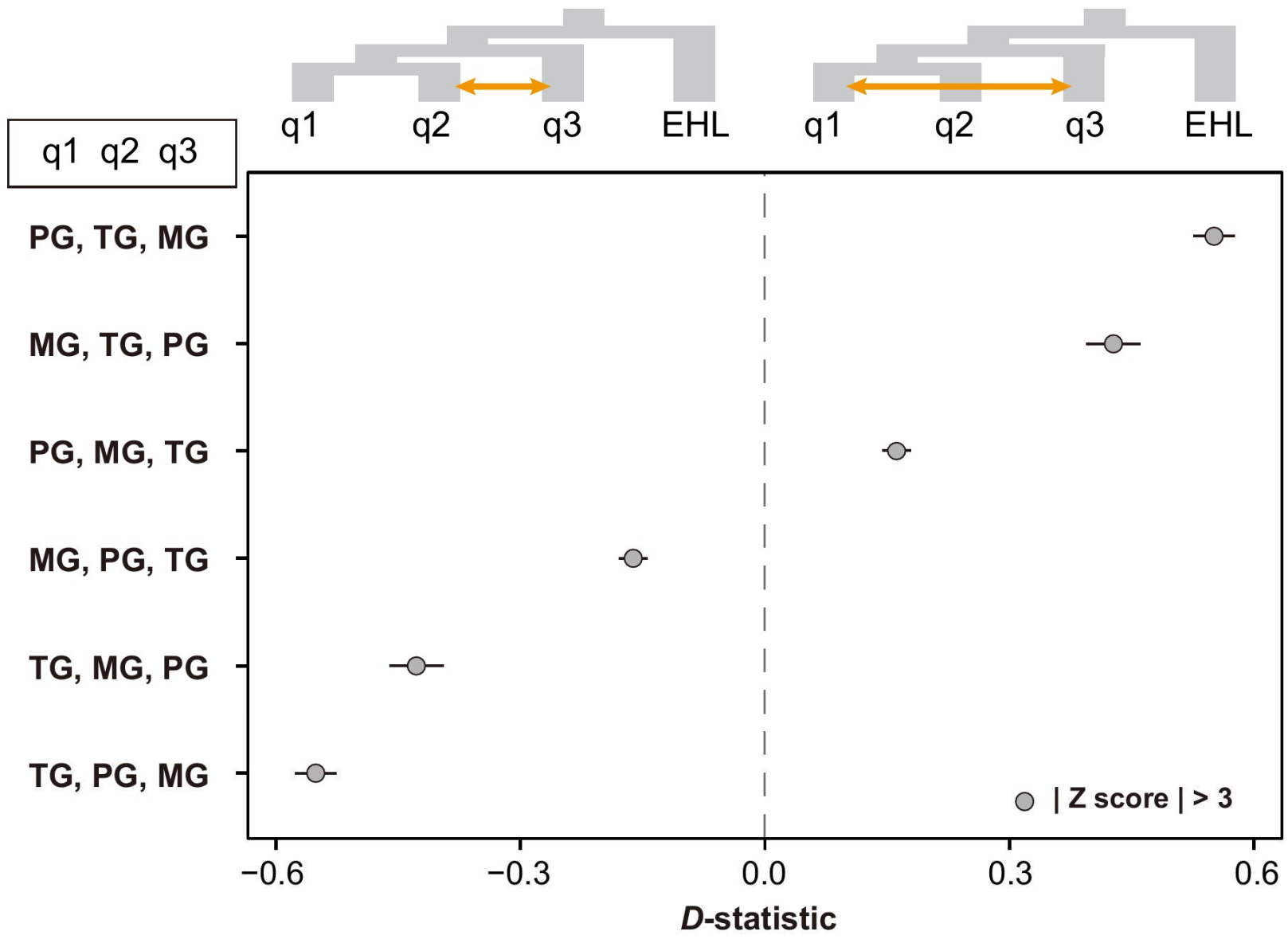

Figure S15. *D*-statistics testing allele-sharing asymmetry among the three *Procapra* species. *D*-statistics were calculated for all permutations of q1, q2 and q3 among MG, PG and TG using the topology (EHL, q3; q2, q1), with EHL as the outgroup. Points indicate *D*-statistic estimates, and horizontal error bars represent standard errors. Gray points denote significant deviations from zero with $|Z| > 3$. Positive and negative values indicate the direction of excess allele sharing under the specified population order.

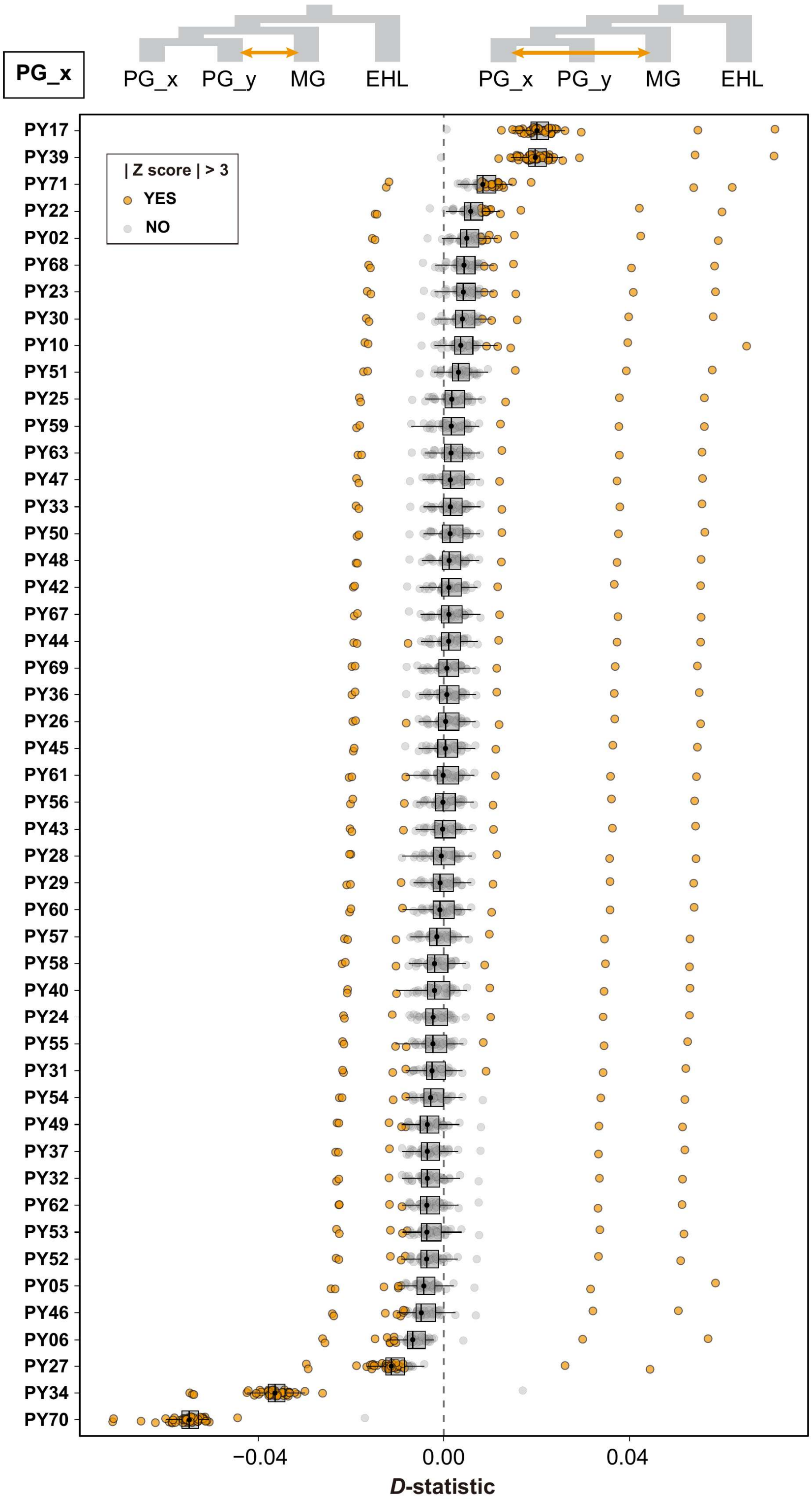

Figure S16. Individual-level *D*-statistics testing allele-sharing asymmetry between PG individuals and MG. *D*-statistics were calculated using the topology (PG_x, PG_y; MG, EHL), with EHL as the outgroup. PG_x on the y-axis represents the focal PG individual being tested, whereas PG_y represents each of the other PG individuals used for pairwise comparison. Yellow circles indicate tests with |Z| > 3,

and gray circles indicate tests with $|Z| \leq 3$. Boxplots summarize the distribution of *D*-statistic values across comparisons for each focal PG_x individual.

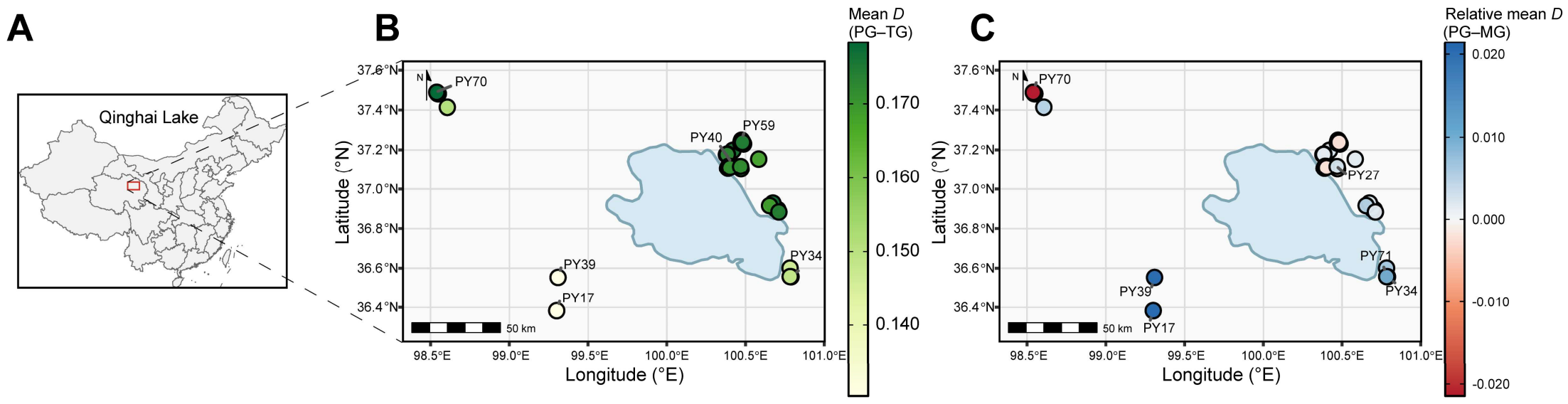

Figure S17. Geographic distribution of TG- and MG-related allele-sharing signals among PG individuals. (A) Location of the Qinghai Lake study area within China. (B) Mean *D*-statistic for each PG individual across comparisons with all 12 TG individuals, with darker green indicating greater TG-related allele sharing. (C) Relative MG-related allele-sharing signal for each PG individual, derived from pairwise *D*-statistic contrasts with the remaining PG individuals in comparisons involving MG. Positive values indicate relatively greater allele sharing with MG, whereas negative values indicate relatively lower allele sharing. Individuals sampled from identical or closely spaced locations were slightly offset to improve visualization.

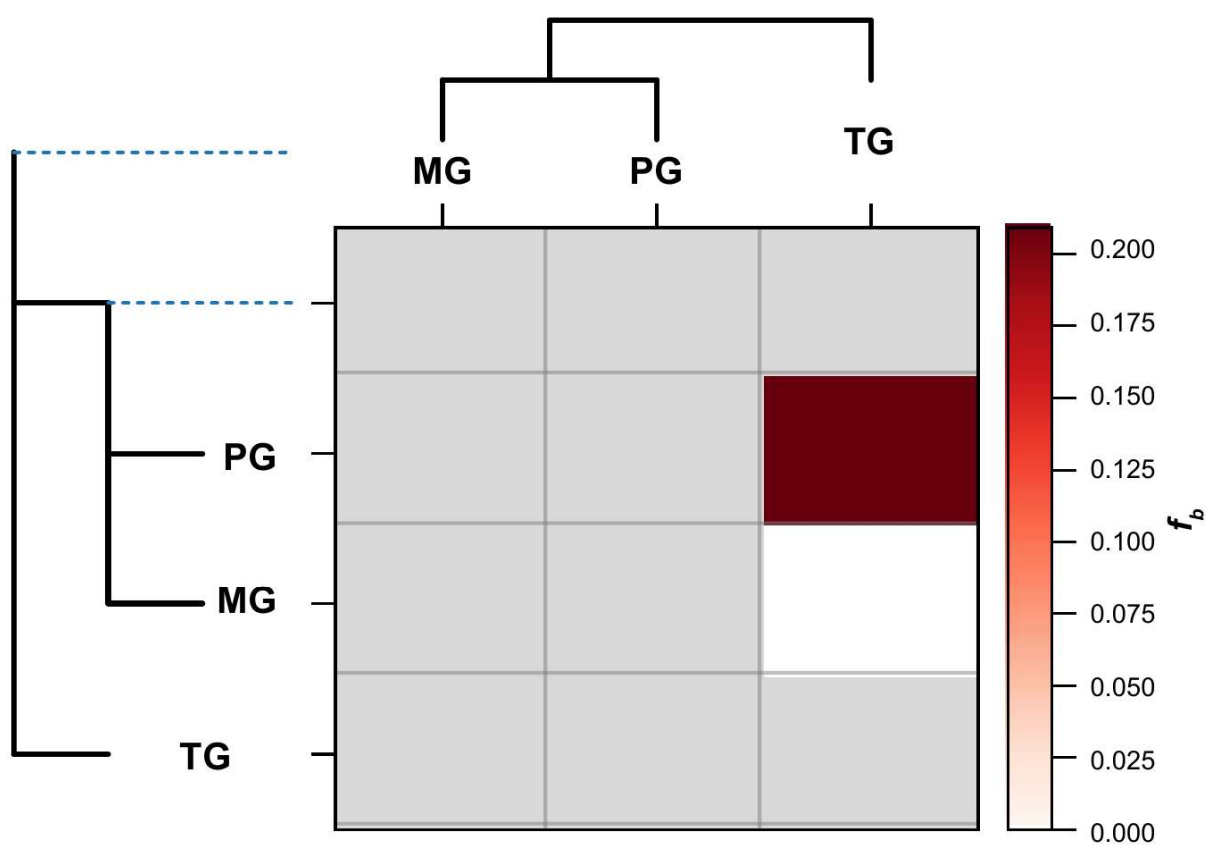

Figure S18. Dsuite f-branch analysis of allele sharing among MG, PG, and TG. The phylogenetic trees indicate the relationships among MG, PG, and TG, and the heatmap shows f-branch (f_b) statistics for the corresponding branch–taxon comparisons. Higher f_b values indicate greater excess allele sharing, with darker red representing stronger signals. Gray cells indicate comparisons that are not applicable under the tested topology. The strongest allele-sharing signal was observed between PG and TG.

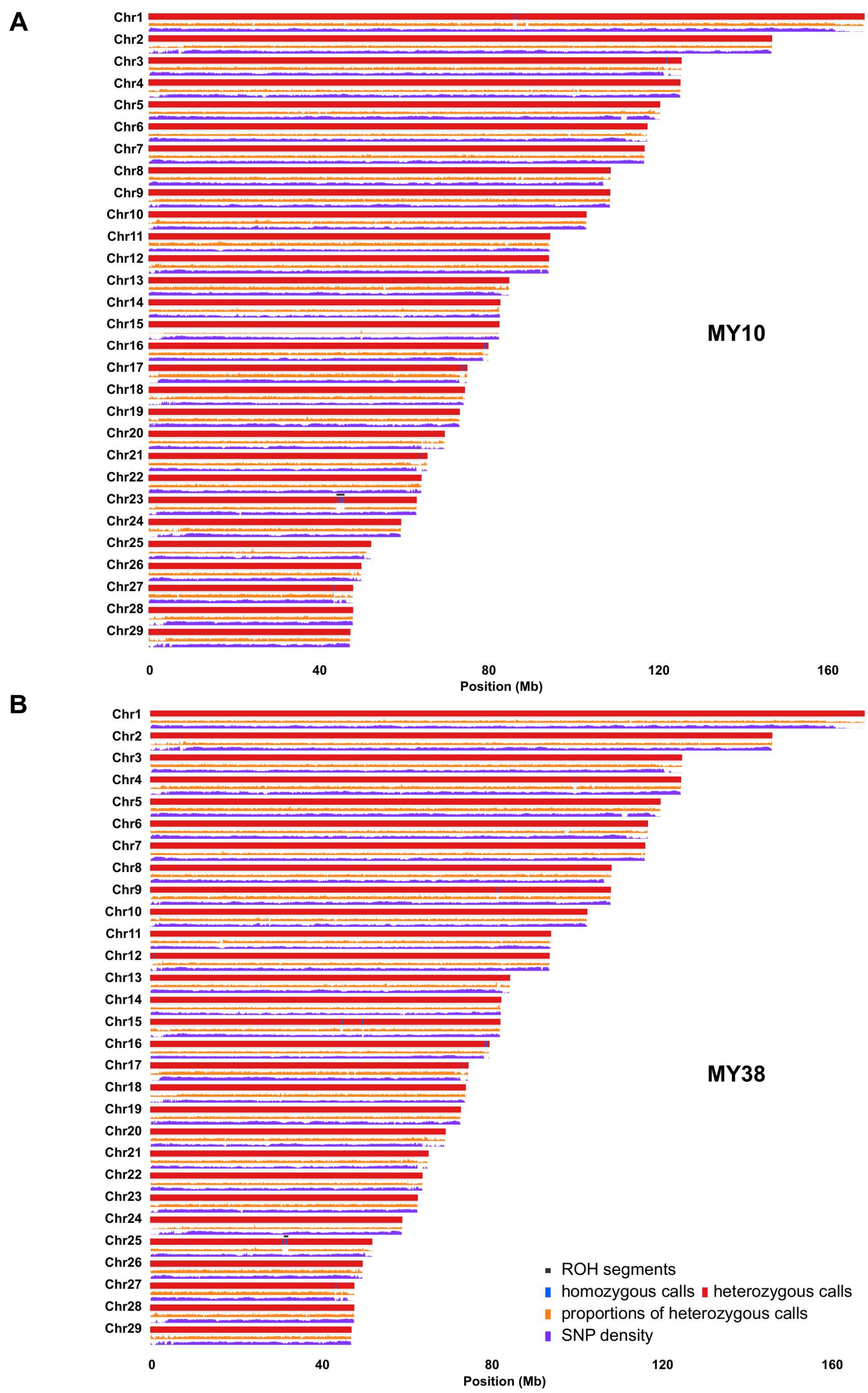

Figure S19. Genotype calls, proportions of heterozygous sites, and SNP density used for ROH validation in representative MG individuals. Results are shown for (A) MY10 and (B) MY38. For each chromosome, identified ROH segments longer than 1,000 kb are indicated by black horizontal bars at the top. Blue vertical bars represent homozygous sites, whereas red vertical bars mark heterozygous sites. The orange line shows the proportion of heterozygous sites in 100-kb windows, and the purple line indicates the number of SNPs per 100-kb window along the genome.

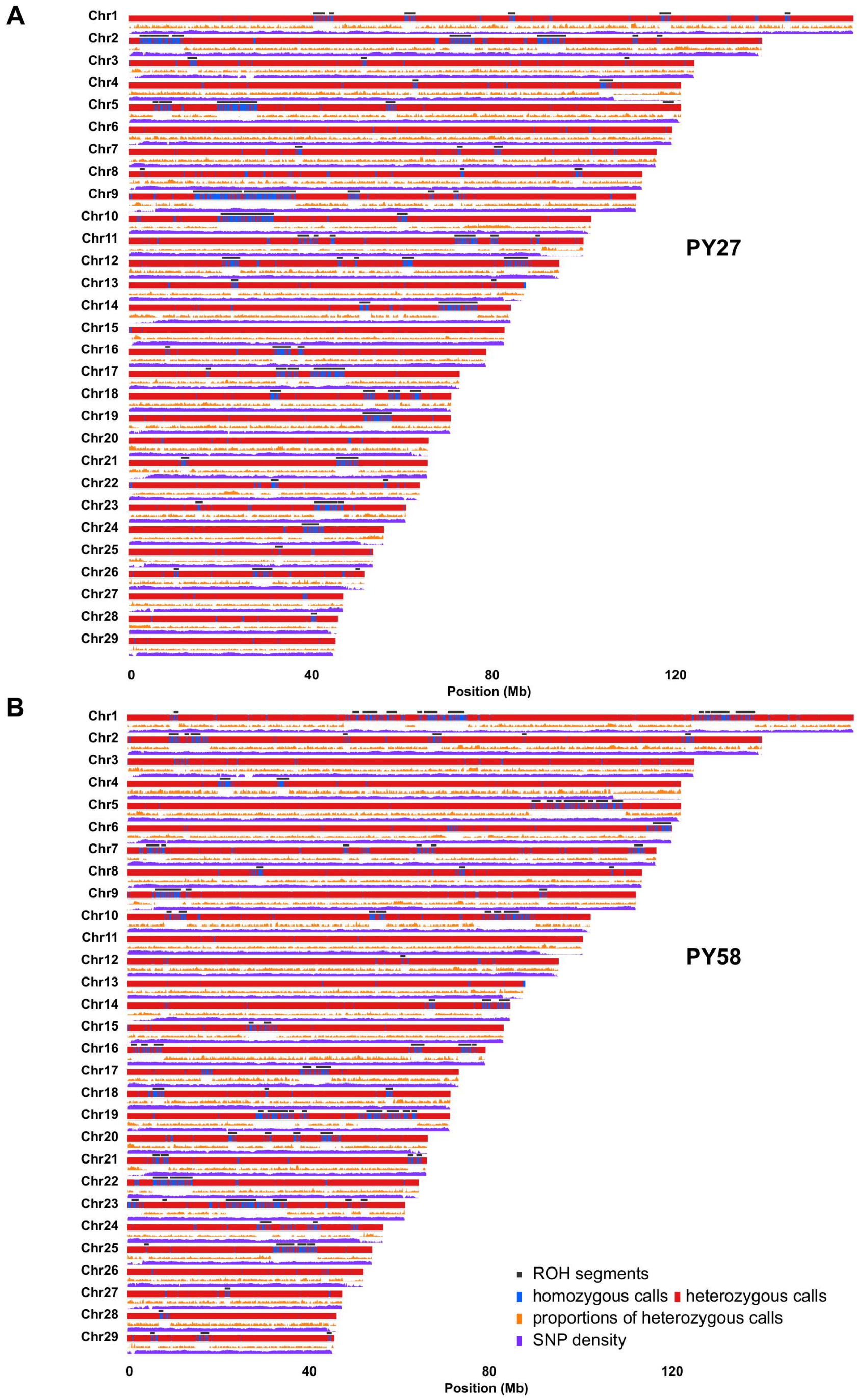

Figure S20. Genotype calls, proportions of heterozygous sites, and SNP density used for ROH validation in representative PG individuals. Results are shown for (A) PY27 and (B) PY58. For each chromosome, identified ROH segments longer than 1,000 kb are indicated by black horizontal bars at the top. Blue vertical bars represent homozygous sites, whereas red vertical bars mark heterozygous sites. The orange line shows the proportion of heterozygous sites in 100-kb windows, and the purple line indicates the number of SNPs per 100-kb window along the genome.

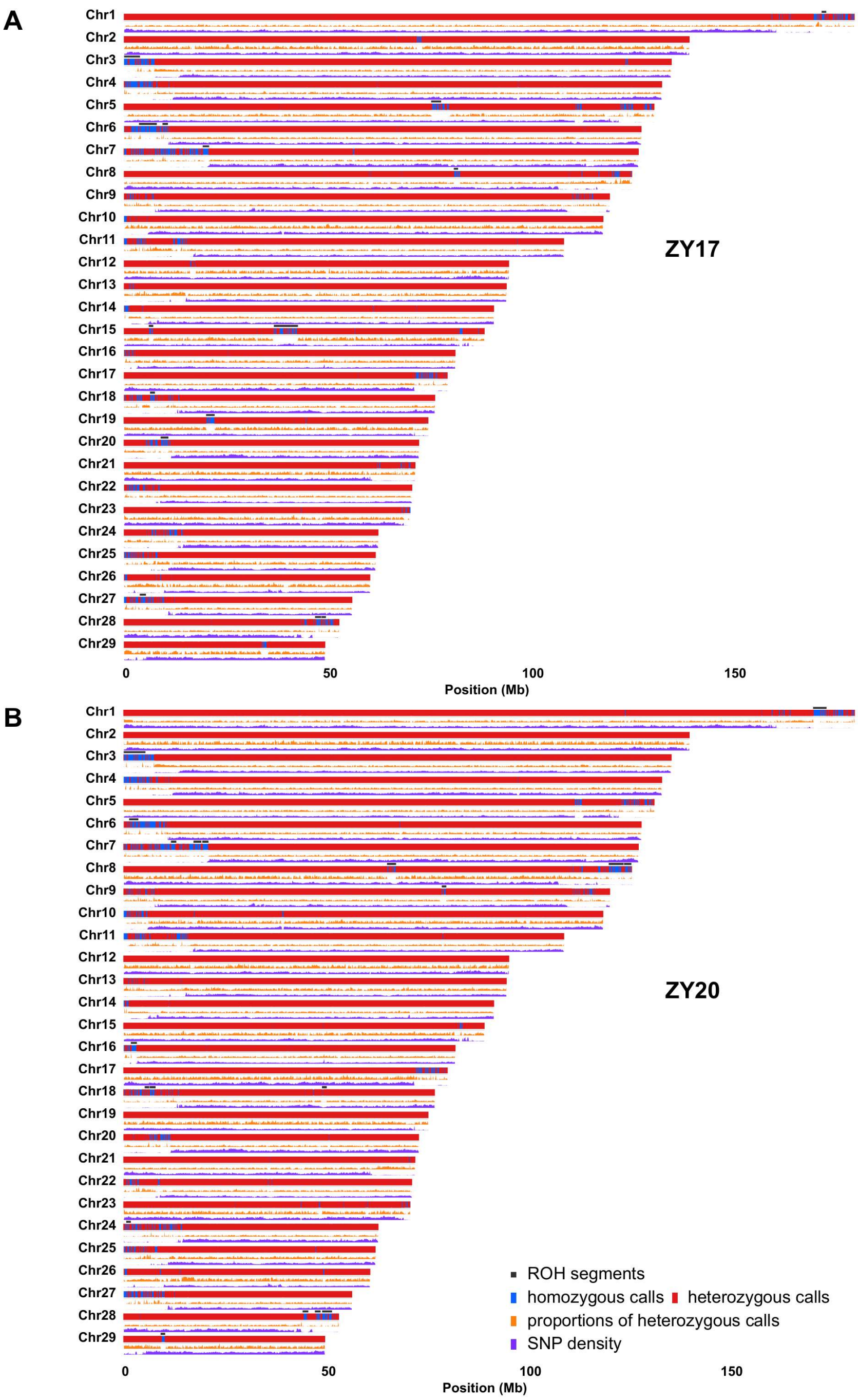

Figure S21. Genotype calls, proportions of heterozygous sites, and SNP density used for ROH validation in representative TG individuals. Results are shown for (A) ZY17 and (B) ZY20. For each chromosome, identified ROH segments longer than 1,000 kb are indicated by black horizontal bars at the top. Blue vertical bars represent homozygous sites, whereas red vertical bars mark heterozygous sites. The orange line shows the proportion of heterozygous sites in 100-kb windows, and the purple line indicates the number of SNPs per 100-kb window along the genome.

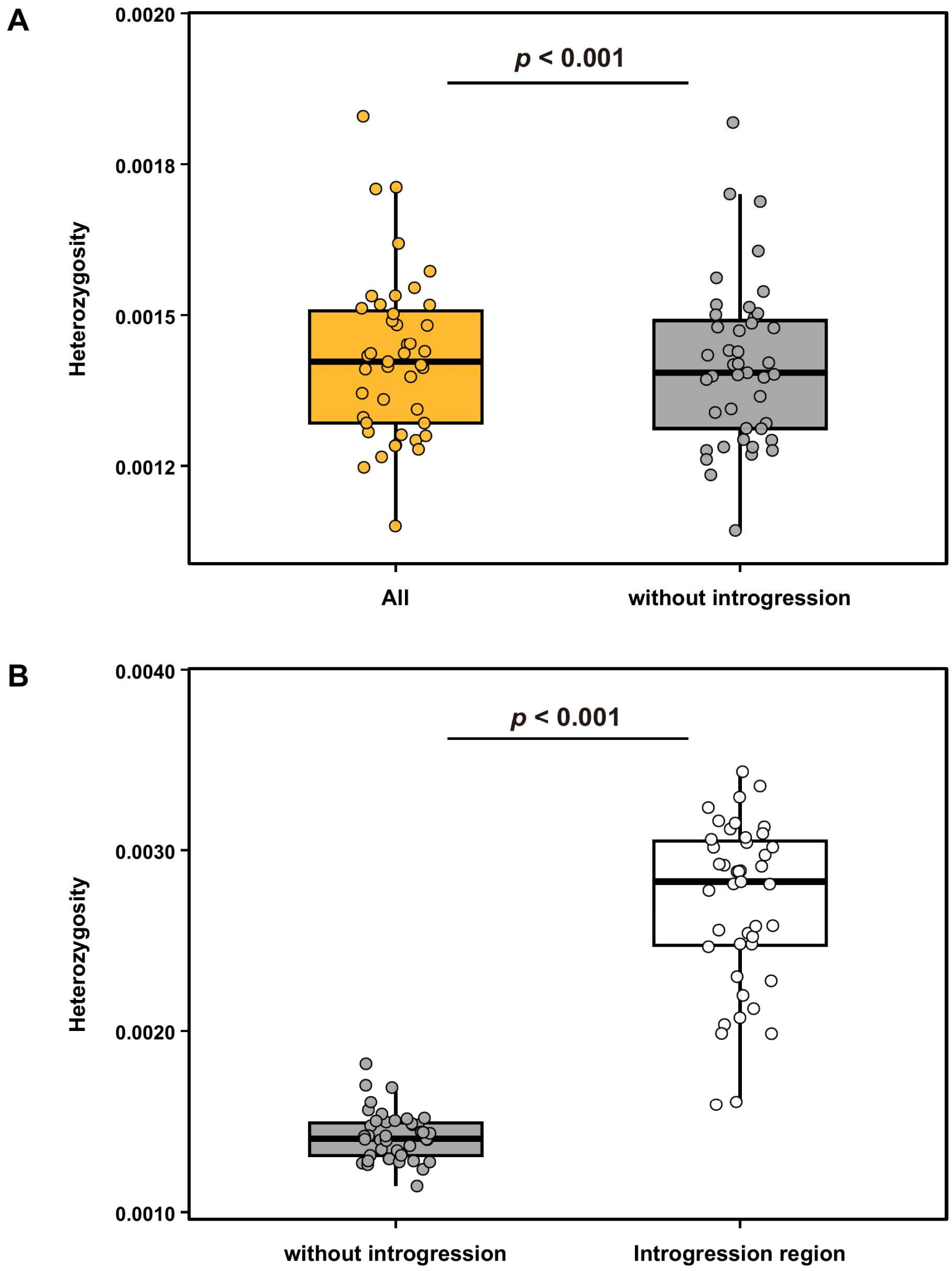

Figure S22. Comparison of heterozygosity in PG with and without inferred introgression regions. (A) Genome-wide heterozygosity compared with heterozygosity calculated after excluding the inferred introgression regions ("without introgression"). (B) Heterozygosity in the "without introgression" background compared with heterozygosity within the inferred introgression regions.

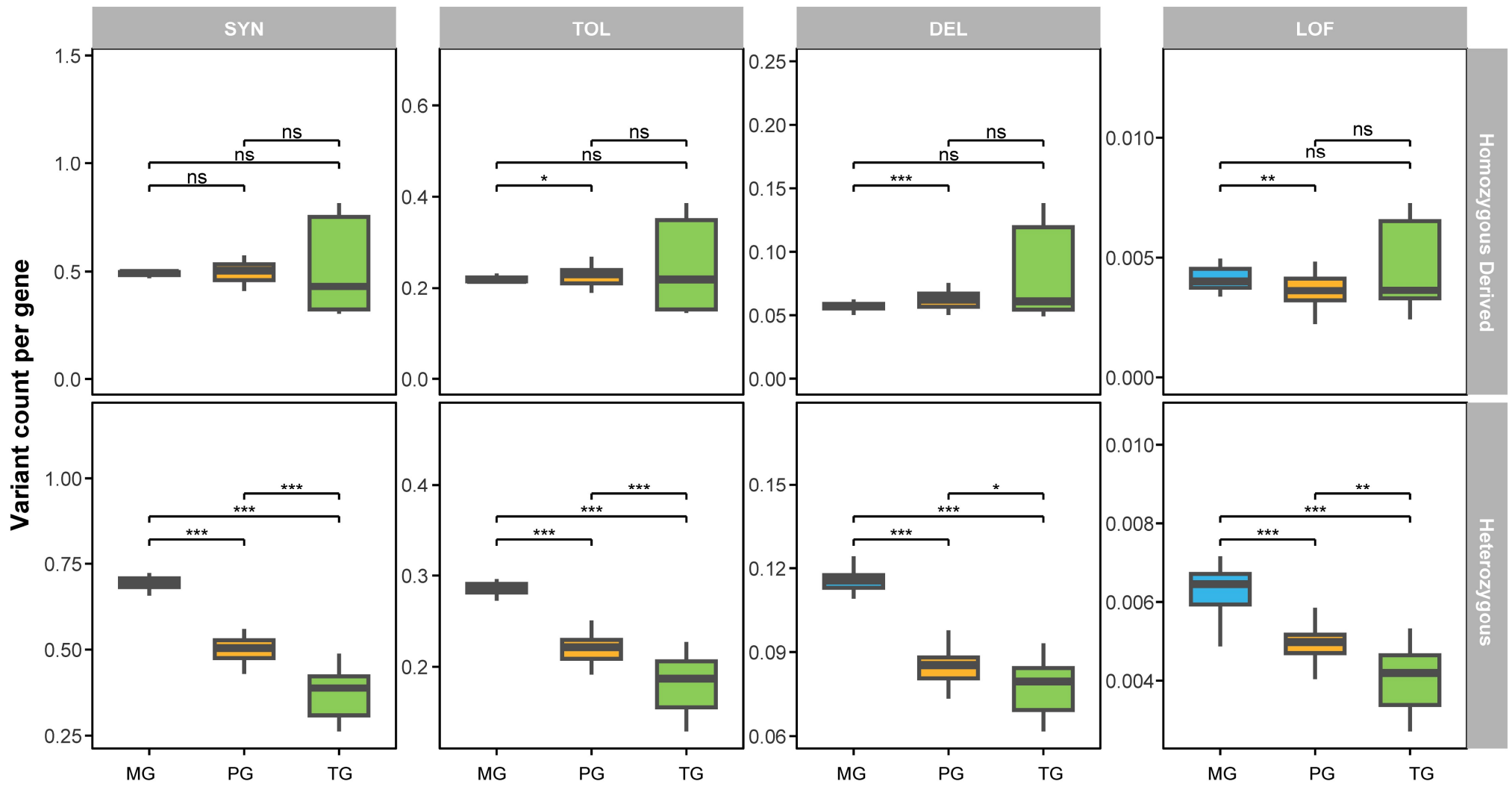

Figure S23. Gene-number-normalized counts of derived variants across the three *Procapra* species. Counts of derived synonymous (SYN), tolerated missense (TOL), deleterious missense (DEL) and loss-of-function (LOF) variants were normalized by the total number of annotated protein-coding genes in each species genome. Results are shown separately for homozygous-derived and heterozygous-derived variants. Boxplots show the distribution across individuals for each species.

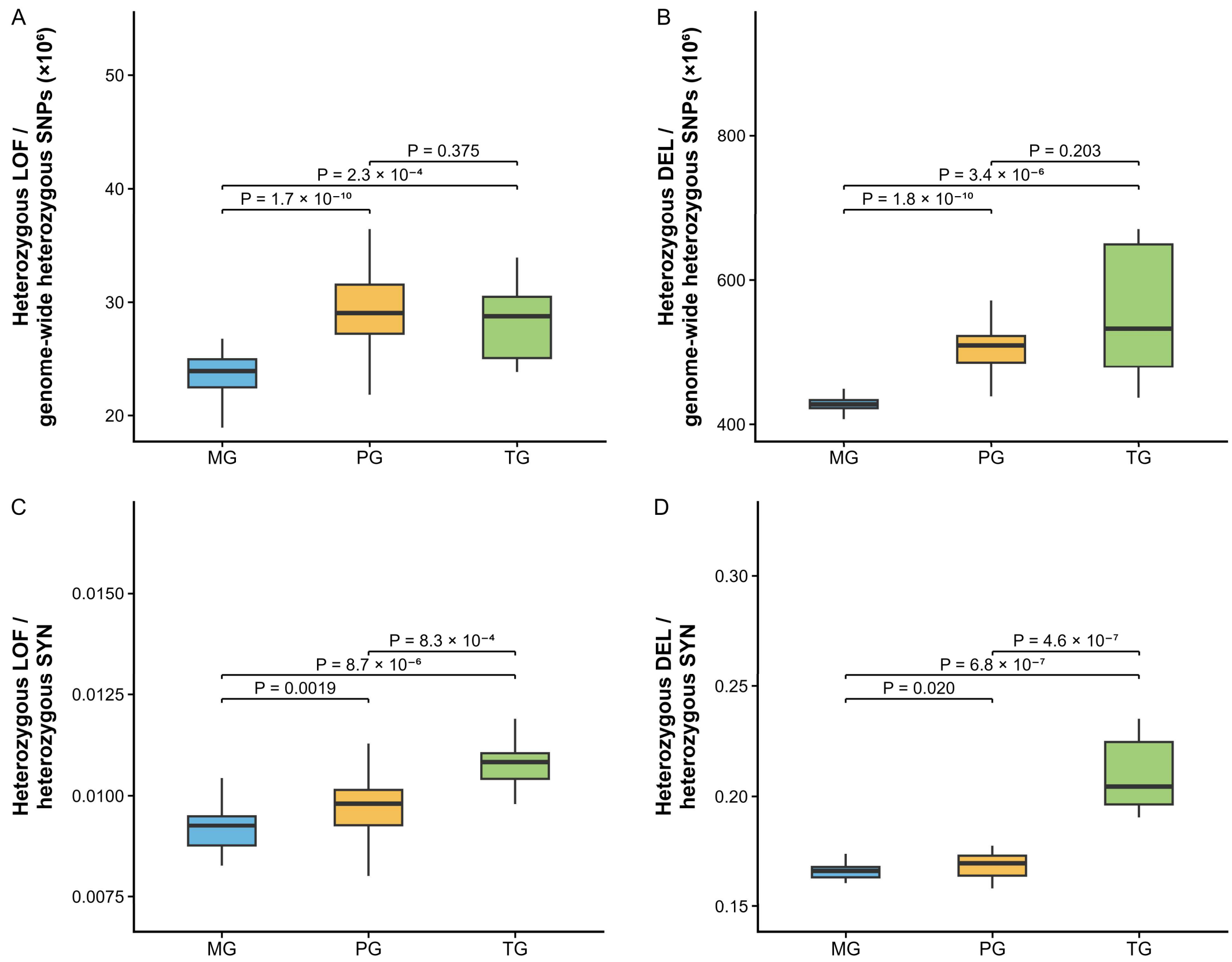

Figure S24. Normalized heterozygous deleterious variation among the three *Procapra* species. (A) Heterozygous LOF variant counts normalized by the total number of genome-wide heterozygous SNPs. (B) Heterozygous DEL variant counts normalized by the total number of genome-wide heterozygous SNPs. (C) Heterozygous LOF/SYN ratios. (D) Heterozygous DEL/SYN ratios. Pairwise comparisons between species were performed using two-sided Wilcoxon rank-sum tests with Benjamini–Hochberg correction.

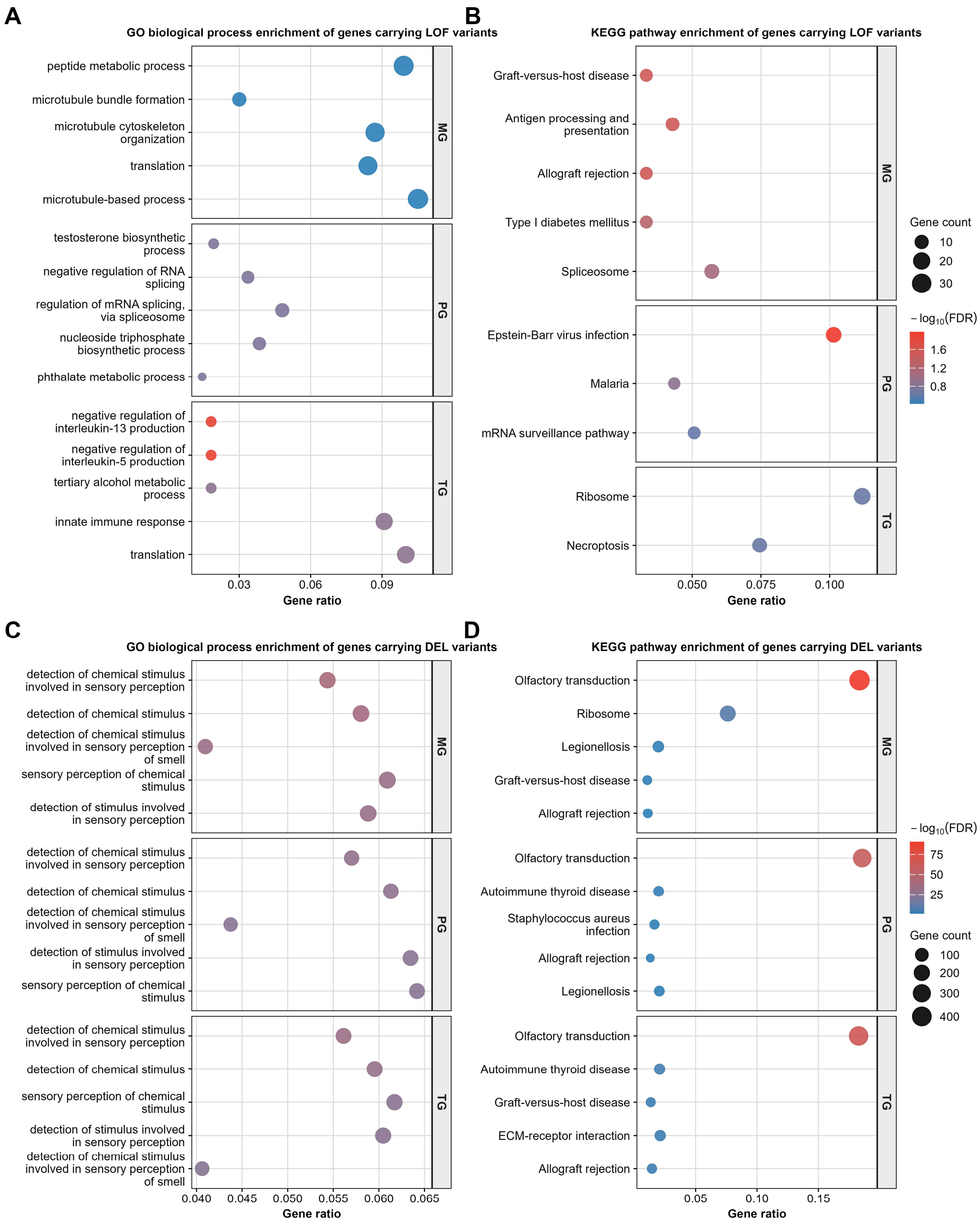

Figure S25. GO Biological Process and KEGG pathway enrichment of genes carrying LOF and DEL variants in MG, PG, and TG. Point size indicates gene count, and color indicates −log10(FDR) based on Benjamini–Hochberg-adjusted *P* values. Up to five top-ranked terms per species are shown for visualization. Full enrichment results are provided in Table S31.

Table S1. Sample information for genome assembly and whole-genome resequencing analyses.

	Species	Sample ID	Sampling Time	Location	Tissue type	Note
1	*Procapra gutturosa* (MG)	MY	2025.02.21	Manzhouli, Hulun Buir, Inner Mongolia	muscle	For *de novo* genome assembly
2	*Procapra przewalskii* (PG)	PY	2024.01.08	Haiyan, Haibei Tibetan Autonomous Prefecture, Qinghai	blood	For *de novo* genome assembly
3	*Procapra picticaudata* (TG)	ZY	2024.01.07	Xining Zoo, Xining, Qinghai	blood	For *de novo* genome assembly
4	MG	MY01	2010.11.21	Manzhouli, Hulun Buir, Inner Mongolia	skin	Resequencing
5	MG	MY02	2010.11.21	Manzhouli, Hulun Buir, Inner Mongolia	skin	Resequencing
6	MG	MY03	2010.11.21	Manzhouli, Hulun Buir, Inner Mongolia	skin	Resequencing
7	MG	MY04	2010.11.21	Manzhouli, Hulun Buir, Inner Mongolia	skin	Resequencing
8	MG	MY05	2010.11.21	Manzhouli, Hulun Buir, Inner Mongolia	skin	Resequencing
9	MG	MY06	2010.11.21	Manzhouli, Hulun Buir, Inner Mongolia	skin	Resequencing
10	MG	MY08	2010.11.21	Manzhouli, Hulun Buir, Inner Mongolia	skin	Resequencing
11	MG	MY10	2010.11.21	Manzhouli, Hulun Buir, Inner Mongolia	skin	Resequencing
12	MG	MY11	2010.11.21	Manzhouli, Hulun Buir, Inner Mongolia	skin	Resequencing
13	MG	MY16	2012.10.16	Manzhouli, Hulun Buir, Inner Mongolia	skin	Resequencing
14	MG	MY18	2012.10.19	Manzhouli, Hulun Buir, Inner Mongolia	skin	Resequencing
15	MG	MY19	2016.10.19	Manzhouli, Hulun Buir, Inner Mongolia	skin	Resequencing
16	MG	MY22	2024.04.16	New Barag West County,Hulun Buir, Inner Mongolia	skin	Resequencing
17	MG	MY24	2024.04.16	New Barag West County,Hulun Buir, Inner Mongolia	skin	Resequencing
18	MG	MY26	2024.04.16	New Barag West County,Hulun Buir, Inner Mongolia	muscle	Resequencing
19	MG	MY28	2024.04.16	New Barag West County,Hulun Buir, Inner Mongolia	muscle	Resequencing
20	MG	MY32	2024.04.16	New Barag West County,Hulun Buir, Inner Mongolia	muscle	Resequencing
21	MG	MY35	2024.04.17	Arxan, Hinggan League, Inner Mongolia	muscle	Resequencing
22	MG	MY36	2024.04.17	Arxan, Hinggan League, Inner Mongolia	muscle	Resequencing

23	MG	MY37	2024.04.17	Arxan, Hinggan League, Inner Mongolia	muscle	Resequencing
24	MG	MY38	2024.04.17	Arxan, Hinggan League, Inner Mongolia	muscle	Resequencing
25	MG	MY39	2024.04.17	Arxan, Hinggan League, Inner Mongolia	muscle	Resequencing
26	MG	MY41	2024.04.17	Arxan, Hinggan League, Inner Mongolia	muscle	Resequencing
27	MG	MY42	2024.04.17	Arxan, Hinggan League, Inner Mongolia	muscle	Resequencing
28	MG	MY43	2024.04.17	Arxan, Hinggan League, Inner Mongolia	muscle	Resequencing
29	MG	MY44	2024.04.17	Arxan, Hinggan League, Inner Mongolia	muscle	Resequencing
30	MG	MY45	2024.04.17	Arxan, Hinggan League, Inner Mongolia	muscle	Resequencing
31	MG	MY46	2024.04.17	Arxan, Hinggan League, Inner Mongolia	muscle	Resequencing
32	MG	MY47	2024.04.17	Arxan, Hinggan League, Inner Mongolia	muscle	Resequencing
33	MG	MY48	2024.04.17	Arxan, Hinggan League, Inner Mongolia	muscle	Resequencing
34	MG	MY49	2024.04.17	Arxan, Hinggan League, Inner Mongolia	muscle	Resequencing
35	MG	MY50	2024.04.17	Arxan, Hinggan League, Inner Mongolia	muscle	Resequencing
36	PG	PY02	2012.06	Gonghe, Hainan Tibetan Autonomous Prefecture, Qinghai	skin	Resequencing
37	PG	PY05	2012.06	Haixi Mongolian and Tibetan Autonomous Prefecture, Qinghai	skin	Resequencing
38	PG	PY06	2012.06	Haixi Mongolian and Tibetan Autonomous Prefecture, Qinghai	skin	Resequencing
39	PG	PY10	——	Haixi Mongolian and Tibetan Autonomous Prefecture, Qinghai	skin	Resequencing
40	PG	PY17	2023.10.01	Gonghe, Hainan Tibetan Autonomous Prefecture, Qinghai	skin	Resequencing
41	PG	PY22	2023.10.09	Haiyan, Haibei Tibetan Autonomous Prefecture, Qinghai	skin	Resequencing
42	PG	PY23	2023.10.22	Haiyan, Haibei Tibetan Autonomous Prefecture, Qinghai	skin	Resequencing
43	PG	PY24	2024.01.08	Haiyan, Haibei Tibetan Autonomous Prefecture, Qinghai	skin	Resequencing
44	PG	PY25	2024.01.08	Haiyan, Haibei Tibetan Autonomous Prefecture, Qinghai	skin	Resequencing
45	PG	PY26	2024.01.08	Haiyan, Haibei Tibetan Autonomous Prefecture, Qinghai	skin	Resequencing
46	PG	PY27	2024.01.08	Haiyan, Haibei Tibetan Autonomous Prefecture, Qinghai	skin	Resequencing
47	PG	PY28	2024.01.09	Haiyan, Haibei Tibetan Autonomous Prefecture, Qinghai	skin	Resequencing

48	PG	PY29	2024.01.09	Haiyan, Haibei Tibetan Autonomous Prefecture, Qinghai	skin	Resequencing
49	PG	PY30	2024.01.09	Haiyan, Haibei Tibetan Autonomous Prefecture, Qinghai	skin	Resequencing
50	PG	PY31	2024.01.09	Haiyan, Haibei Tibetan Autonomous Prefecture, Qinghai	skin	Resequencing
51	PG	PY32	2024.01.09	Haiyan, Haibei Tibetan Autonomous Prefecture, Qinghai	skin	Resequencing
52	PG	PY33	2024.01.09	Haiyan, Haibei Tibetan Autonomous Prefecture, Qinghai	skin	Resequencing
53	PG	PY34	2024.01.13	Gonghe, Hainan Tibetan Autonomous Prefecture, Qinghai	skin	Resequencing
54	PG	PY36	2024.01.13	Haiyan, Haibei Tibetan Autonomous Prefecture, Qinghai	skin	Resequencing
55	PG	PY37	2024.01.14	Gangcha, Haibei Tibetan Autonomous Prefecture, Qinghai	skin	Resequencing
56	PG	PY39	2024.01.17	Gonghe, Hainan Tibetan Autonomous Prefecture, Qinghai	skin	Resequencing
57	PG	PY40	2024.01.18	Gangcha, Haibei Tibetan Autonomous Prefecture, Qinghai	skin	Resequencing
58	PG	PY42	2024.01.18	Gangcha, Haibei Tibetan Autonomous Prefecture, Qinghai	skin	Resequencing
59	PG	PY43	2024.01.19	Gangcha, Haibei Tibetan Autonomous Prefecture, Qinghai	skin	Resequencing
60	PG	PY44	2024.01.19	Gangcha, Haibei Tibetan Autonomous Prefecture, Qinghai	skin	Resequencing
61	PG	PY45	2024.01.19	Gangcha, Haibei Tibetan Autonomous Prefecture, Qinghai	skin	Resequencing
62	PG	PY46	2024.01.20	Gangcha, Haibei Tibetan Autonomous Prefecture, Qinghai	skin	Resequencing
63	PG	PY47	2024.01.20	Gangcha, Haibei Tibetan Autonomous Prefecture, Qinghai	skin	Resequencing
64	PG	PY48	2024.01.20	Gangcha, Haibei Tibetan Autonomous Prefecture, Qinghai	skin	Resequencing
65	PG	PY49	2024.01.20	Gangcha, Haibei Tibetan Autonomous Prefecture, Qinghai	skin	Resequencing
66	PG	PY50	2024.01.20	Gangcha, Haibei Tibetan Autonomous Prefecture, Qinghai	skin	Resequencing
67	PG	PY51	2024.01.20	Gangcha, Haibei Tibetan Autonomous Prefecture, Qinghai	skin	Resequencing
68	PG	PY52	2024.01.20	Gangcha, Haibei Tibetan Autonomous Prefecture, Qinghai	skin	Resequencing
69	PG	PY53	2024.01.21	Gangcha, Haibei Tibetan Autonomous Prefecture, Qinghai	skin	Resequencing
70	PG	PY54	2024.01.21	Gangcha, Haibei Tibetan Autonomous Prefecture, Qinghai	skin	Resequencing
71	PG	PY55	2024.01.21	Gangcha, Haibei Tibetan Autonomous Prefecture, Qinghai	skin	Resequencing
72	PG	PY56	2024.01.21	Gangcha, Haibei Tibetan Autonomous Prefecture, Qinghai	skin	Resequencing

73	PG	PY57	2024.01.21	Gangcha, Haibei Tibetan Autonomous Prefecture, Qinghai	skin	Resequencing
74	PG	PY58	2024.01.21	Gangcha, Haibei Tibetan Autonomous Prefecture, Qinghai	skin	Resequencing
75	PG	PY59	2024.01.21	Gangcha, Haibei Tibetan Autonomous Prefecture, Qinghai	skin	Resequencing
76	PG	PY60	2024.01.21	Gangcha, Haibei Tibetan Autonomous Prefecture, Qinghai	skin	Resequencing
77	PG	PY61	2024.01.21	Gangcha, Haibei Tibetan Autonomous Prefecture, Qinghai	skin	Resequencing
78	PG	PY62	2024.01.21	Gangcha, Haibei Tibetan Autonomous Prefecture, Qinghai	skin	Resequencing
79	PG	PY63	2024.01.21	Haiyan, Haibei Tibetan Autonomous Prefecture, Qinghai	skin	Resequencing
80	PG	PY67	2024.03.16	Protection Station, Gonghe, Hainan Tibetan Autonomous Prefecture, Qinghai	muscle	Resequencing
81	PG	PY68	2024.03.16	Protection Station, Gonghe, Hainan Tibetan Autonomous Prefecture, Qinghai	muscle	Resequencing
82	PG	PY69	2024.03.16	Protection Station, Gonghe, Hainan Tibetan Autonomous Prefecture, Qinghai	muscle	Resequencing
83	PG	PY70	2024.03.03	Haixi Mongolian and Tibetan Autonomous Prefecture, Qinghai	skin	Resequencing
84	PG	PY71	2024.03.15	Gonghe, Hainan Tibetan Autonomous Prefecture, Qinghai	muscle	Resequencing
85	TG	ZY02	2020.4	Shuanghu, Nagqu, Tibet	skin	Resequencing
86	TG	ZY04	2006	Dulan, Haixi Mongolian and Tibetan Autonomous Prefecture, Qinghai	skin	Resequencing
87	TG	ZY06	2020.4	Shuanghu, Nagqu, Tibet	skin	Resequencing
88	TG	ZY07	2020.4	Shuanghu, Nagqu, Tibet	skin	Resequencing
89	TG	ZY08	2016	Comai, Shannan, Tibet	skin	Resequencing
90	TG	ZY10	——	Shuanghu, Nagqu, Tibet	skin	Resequencing
91	TG	ZY16	1964	Wanyuan, Dazhou, Sichuan	skin	Resequencing
92	TG	ZY17	2012.06	Tianjun, Haixi Mongolian and Tibetan Autonomous Prefecture, Qinghai	skin	Resequencing
93	TG	ZY18	2012.06	Tianjun, Haixi Mongolian and Tibetan Autonomous Prefecture, Qinghai	skin	Resequencing
94	TG	ZY19	2012.06	Tianjun, Haixi Mongolian and Tibetan Autonomous Prefecture, Qinghai	skin	Resequencing
95	TG	ZY20	2012.06	Tianjun, Haixi Mongolian and Tibetan Autonomous Prefecture, Qinghai	skin	Resequencing
96	TG	ZY21	2024.04.18	Shiqu, Tibetan Autonomous Prefecture of Garzê, Sichuan	blood	Resequencing
97	*Gazella subgutturosa*	EHL07	2012.02.13	Shanshan, Turpan, Xinjiang	skin	Resequencing

Table S2. Summary statistics of sequencing reads used for genome assembly.

Species	Sequencing platforms	Cell/Pair	Sum of lengths (bp)	Minimum length (bp)	Maximum length (bp)	Average length (bp)
Procapra gutturosa	HiFi	m84072_250320_113836_s2	95,148,925,518	127	56,515	18957.7
	Hi-C	R1	182,053,120,950	150	150	150
		R2	182,053,120,950	150	150	150
	DNBSEQ	R1	41,570,863,584	31	150	149.8
		R2	41,570,863,584	31	150	149.8
Procapra przewalskii	HiFi	m84178_240712_155445_s3	87,591,796,196	81	71,526	15266.9
	Hi-C	R1	178,089,118,350	150	150	150
		R2	178,089,118,350	150	150	150
	DNBSEQ	R1	50,313,976,689	31	150	149.12
		R2	50,313,976,689	31	150	149.12
Procapra picticaudata	HiFi	m84178_240318_140433_s3	97,714,025,233	117	61,106	18424.4
	Hi-C	R1	159,909,776,550	150	150	150
		R2	159,909,776,550	150	150	150
	DNBSEQ	R1	43,693,159,836	31	150	149.55
		R2	43,693,159,836	31	150	149.55

Table S3. Genome assembly statistics for the three *Procapra* species.

Features	MG	PG	TG
Genome size (Gb)	2.72	2.69	2.93
Contig N50 (Mb)	82.49	94.83	91.11
Scaffold N50 (Mb)	102.69	101.9	117.96
Number of contigs	102	412	57
Number of scaffolds	63	61	48
GC content	42.80%	42.70%	44.10%
HiFi mapping rate	99.99%	98.26%	99.95%
NGS mapping rate	99.82%	97.08%	99.95%
Base quality (QV)	47.99	48.98	48.46

Table S4. Lengths of pseudochromosomes and unplaced scaffolds in the three *Procapra* genome assemblies.

	MG		PG		TG	
**Type**	**Pseudochromosomes name**	**length (bp)**	**Pseudochromosomes name**	**length (bp)**	**Pseudochromosomes name**	**length (bp)**
Pseudochromosomes	Chr1	167,932,311	Chr1	159,793,977	Chr1	179,880,548
	Chr2	146,254,313	Chr2	139,547,079	Chr2	139,224,711
	Chr3	124,971,921	Chr3	124,637,495	Chr3	134,794,786
	Chr4	124,770,653	Chr4	121,744,452	Chr4	132,429,060
	Chr5	119,960,098	Chr5	121,733,871	Chr5	130,613,170
	Chr6	116,948,732	Chr6	119,734,509	Chr6	127,383,634
	Chr7	116,330,037	Chr7	116,324,554	Chr7	126,663,029
	Chr8	108,393,446	Chr8	113,135,289	Chr8	125,054,731
	Chr9	108,250,742	Chr9	111,820,528	Chr9	119,621,240
	Chr10	102,694,376	Chr10	101,896,419	Chr10	117,959,076
	Chr11	94,186,528	Chr11	100,207,238	Chr11	108,328,118
	Chr12	93,854,066	Chr12	94,825,760	Chr12	94,793,433
	Chr13	84,551,735	Chr13	87,546,938	Chr13	94,191,046
	Chr14	82,441,000	Chr14	84,181,183	Chr14	91,110,610
	Chr15	82,244,573	Chr15	82,711,171	Chr15	88,742,391
	Chr16	79,695,761	Chr16	78,715,309	Chr16	81,580,679
	Chr17	74,806,208	Chr17	72,868,955	Chr17	79,745,036
	Chr18	74,147,783	Chr18	71,000,603	Chr18	76,521,563
	Chr19	72,972,235	Chr19	70,918,857	Chr19	74,914,145
	Chr20	69,425,287	Chr20	65,998,585	Chr20	72,606,385
	Chr21	65,385,757	Chr21	65,799,844	Chr21	71,700,947

	Chr22	63,974,582	Chr22	64,035,916	Chr22	70,916,577
	Chr23	62,829,765	Chr23	61,008,221	Chr23	70,575,300
	Chr24	59,192,604	Chr24	56,197,597	Chr24	62,624,868
	Chr25	52,160,856	Chr25	53,763,403	Chr25	61,926,092
	Chr26	49,903,215	Chr26	51,846,943	Chr26	60,616,000
	Chr27	47,944,739	Chr27	47,167,411	Chr27	56,155,687
	Chr28	47,905,819	Chr28	45,983,462	Chr28	53,007,046
	Chr29	47,252,600	Chr29	45,477,689	Chr29	49,503,529
	ChrX	144,410,471	ChrX	139,896,603	ChrX	151,895,141
	ChrY	10,750,000	ChrY	12,013,862	ChrY	19,111,130
Unplaced scaffold	Scaffold_32	6,273,520	Scaffold_33	987,000	Scaffold_32	2,500,000
	Scaffold_33	3,828,035	Scaffold_34	701,818	Scaffold_33	1,752,499
	Scaffold_34	3,783,925	Scaffold_35	509,582	Scaffold_34	996,018
	Scaffold_35	2,002,934	Scaffold_36	250,000	Scaffold_35	863,927
	Scaffold_36	1,878,000	Scaffold_37	196,012	Scaffold_36	846,168
	Scaffold_37	729,213	Scaffold_38	177,119	Scaffold_37	657,000
	Scaffold_38	492,920	Scaffold_39	165,835	Scaffold_38	620,000
	Scaffold_39	279,000	Scaffold_40	157,771	Scaffold_39	299,000
	Scaffold_40	259,000	Scaffold_41	157,000	Scaffold_40	222,000
	Scaffold_41	229,702	Scaffold_42	154,093	Scaffold_41	202,068
	Scaffold_42	225,708	Scaffold_43	135,492	Scaffold_42	191,000
	Scaffold_43	224,302	Scaffold_44	134,509	Scaffold_43	148,000
	Scaffold_44	210,000	Scaffold_45	134,000	Scaffold_44	142,000
	Scaffold_45	167,945	Scaffold_46	118,733	Scaffold_45	131,000
	Scaffold_46	126,402	Scaffold_47	112,035	Scaffold_46	64,000

	Scaffold_47	105,000	Scaffold_48	106,859	Scaffold_47	59,100
	Scaffold_48	96,000	Scaffold_49	102,000	Scaffold_48	46,094
	Scaffold_49	94,000	Scaffold_50	89,599		
	Scaffold_50	72,000	Scaffold_51	77,528		
	Scaffold_51	71,000	Scaffold_52	73,000		
	Scaffold_52	62,000	Scaffold_53	71,940		
	Scaffold_53	60,000	Scaffold_54	71,839		
	Scaffold_54	52,757	Scaffold_55	50,729		
	Scaffold_55	51,015	Scaffold_56	50,116		
	Scaffold_56	51,000	Scaffold_57	39,000		
	Scaffold_57	43,000	Scaffold_58	35,874		
	Scaffold_58	35,000	Scaffold_59	35,118		
	Scaffold_59	33,941	Scaffold_60	31,473		
	Scaffold_60	32,296	Scaffold_61	23,865		
	Scaffold_61	29,813	Scaffold_62	23,136		
	Scaffold_62	26,081				
	Scaffold_63	19,000				
Total		2,718,186,722		2,687,506,798		2,933,929,582

Table S5. Assessment of genome-assembly completeness for the three *Procapra* species using BUSCO.

Species	Type	Number	Percent
MG	Complete BUSCOs (C)	9,047	98.10%
	Complete and single-copy BUSCOs (S)	8,899	96.50%
	Complete and duplicated BUSCOs (D)	148	1.60%
	Fragmented BUSCOs (F)	103	1.10%
	Missing BUSCOs (M)	76	0.80%
	Total BUSCO groups searched	9,226	100.00%
PG	Complete BUSCOs (C)	9,048	98.10%
	Complete and single-copy BUSCOs (S)	8,890	96.40%
	Complete and duplicated BUSCOs (D)	158	1.70%
	Fragmented BUSCOs (F)	100	1.10%
	Missing BUSCOs (M)	78	0.80%
	Total BUSCO groups searched	9,226	100.00%
TG	Complete BUSCOs (C)	9,056	98.20%
	Complete and single-copy BUSCOs (S)	8,908	96.60%
	Complete and duplicated BUSCOs (D)	148	1.60%
	Fragmented BUSCOs (F)	92	1.00%
	Missing BUSCOs (M)	78	0.80%
	Total BUSCO groups searched	9,226	100.00%

Table S6. Repeat content of the *Procapra* genome estimated by RepeatMasker software.

Species	Type	Super family	Number of elements	Length of sequence (bp)	Percentage of sequence (%)
MG	SINEs		1,926,114	255,351,120	9.39
		Alu/B1	2	147	0
		MIRs	532,665	58,304,851	2.14
	LINEs		1,436,112	700,844,228	25.78
		LINE1	670,120	291,348,746	10.72
		LINE2	249,604	58,483,479	2.15
		L3/CR1	36,041	6,975,981	0.26
		RTE	479,251	343,873,578	12.65
	LTR elements		501,664	138,865,193	5.11
		ERVL	73,526	28,255,422	1.04
		ERVL-MaLRs	117,461	37,786,618	1.39
		ERV_classI	110,859	43,832,113	1.61
		ERV_classII	181,987	23,842,726	0.88
	DNA elements		304,453	59,816,028	2.2
		hAT-Charlie	170,183	30,223,626	1.11
		TcMar-Tigger	55,261	13,693,241	0.5
	Unclassified		9,955	1,252,836	0.05
	Total interspersed repeats			1,156,129,405	42.53
	Small RNA		89	13,031	0
	Satellites		139,828	165,127,801	6.07
	Simple repeats		0	0	0
	Low complexity		0	0	0

	Total repeats			1,321,526,667	48.62
PG	SINEs		1,924,945	255,198,203	9.5
		Alu/B1	4	264	0
		MIRs	532,900	58,293,617	2.17
	LINEs		1,435,651	699,988,724	26.05
		LINE1	670,064	291,280,386	10.84
		LINE2	249,601	58,482,244	2.18
		L3/CR1	36,124	6,984,019	0.26
		RTE	478,764	343,079,930	12.77
	LTR elements		499,702	137,901,177	5.13
		ERVL	73,455	28,219,355	1.05
		ERVL-MaLRs	117,267	37,664,920	1.4
		ERV_classI	110,145	43,337,189	1.61
		ERV_classII	181,212	23,637,540	0.88
	DNA elements		304,338	59,771,527	2.22
		hAT-Charlie	170,077	30,224,820	1.12
		TcMar-Tigger	55,170	13,656,893	0.51
	Unclassified		9,889	1,249,415	0.05
	Total interspersed repeats			1,154,109,046	42.94
	Small RNA		86	12,526	0
	Satellites		132,634	140,126,998	5.21
	Simple repeats		0	0	0
	Low complexity		0	0	0
	Total repeats			1,294,500,708	48.17
TG	SINEs		1,934,636	256,386,477	8.74

		Alu/B1	4	321	0
		MIRs	535,442	58,529,771	1.99
	LINEs		1,442,327	706,291,871	24.07
		LINE1	671,982	293,479,247	10
		LINE2	249,972	58,656,153	2
		L3/CR1	36,273	6,990,464	0.24
		RTE	483,028	347,007,460	11.83
	LTR elements		504,909	139,792,498	4.76
		ERVL	73,663	28,347,460	0.97
		ERVL-MaLRs	117,803	37,997,737	1.3
		ERV_classI	111,589	44,093,965	1.5
		ERV_classII	184,086	24,279,058	0.83
	DNA elements		305,376	59,989,209	2.04
		hAT-Charlie	170,674	30,322,286	1.03
		TcMar-Tigger	55,577	13,756,605	0.47
	Unclassified		10,117	1,276,281	0.04
	Total interspersed repeats			1,163,736,336	39.66
	Small RNA		93	12,303	0
	Satellites		228,203	370,067,536	12.61
	Simple repeats		0	0	0
	Low complexity		0	0	0
	Total repeats			1,534,068,284	52.29

Table S7. Repeat sequence statistics of the *Procapra* species genome integrated by four software.

Species	Software	Repeat Size (bp)	Percentage of sequence (%)
MG	TRF	138,093,208	5.08
	RepeatMasker	1,321,526,667	48.618
	RepeatProteinMask	537,062,471	19.758
	RepeatModeler	1,220,030,541	44.884
	Total	1,452,702,278	53.444
PG	TRF	102,515,540	3.815
	RepeatMasker	1,294,500,708	48.167
	RepeatProteinMask	536,020,269	19.945
	RepeatModeler	1,111,962,129	41.375
	Total	1,422,157,420	52.917
TG	TRF	103,822,541	3.539
	RepeatMasker	1,534,068,284	52.287
	RepeatProteinMask	541,348,707	18.451
	RepeatModeler	1,106,271,023	37.706
	Total	1,666,062,018	56.786

Table S8. Basic statistics of the protein-coding genes for the *Procapra* and related species.

Scientific name	English name	GenBank accession	RefSeq accession	Total number of genes	Average gene Length (bp)	Average CDS Length (bp)	Average exon number per gene	Average exon Length (bp)	Average intron Length (bp)
Bubalus bubalis	water buffalo	GCA_019923935.1	GCF_019923935.1	21,359	53336.62	1632.75	9.16	178.27	5279.79
Budorcas tibetana	takin	GCA_023091745.2	GCF_023091745.1	20,434	47920.9	1713.17	9.66	177.26	4932.31
Bos mutus	Wild yak	GCA_000298355.1	GCF_000298355.1	20,519	42899.72	1607.11	9.58	167.8	4401.53
Bos taurus	domestic cattle	GCA_002263795.3	GCF_002263795.3	21,698	52944.47	1625.2	9.11	178.33	5209.06
Capra hircus	goat	GCA_001704415.2	GCF_001704415.2	20,572	51307.94	1667.68	9.48	175.84	5051.9
Ovis aries	sheep	GCA_016772045.2	GCF_016772045.2	21,341	54577.78	1639.51	9.13	179.66	5243.84
Ovis ammon polii	argali	GCA_028583565.1	NA	20,907	48630.44	1573.36	9.21	170.78	5729.59
Procapra gutturosa	Mongolian gazelle	This study	This study	21,397	43373.23	1625.58	9.39	173.12	4975.99
Procapra przewalskii	Przewalski's gazelle	This study	This study	21,532	42980.85	1611.59	9.33	172.76	4967.31
Procapra picticaudata	Tibetan gazelle	This study	This study	21,455	43266.7	1616.27	9.35	172.77	4985.22
Rangifer tarandus platyrhynchus	Svalbard reindeer	GCA_949782905.1	NA	32,643	33215.56	1339.52	8.49	157.84	4257.8

Table S9. Statistics of gene functional annotation.

Species	Database	Number	Percent
MG	Swiss-Prot	20,268	94.72%
	NR	20,698	96.73%
	Total Annotated	20,702	96.75%
	Total gene	21,397	100%
PG	Swiss-Prot	20,351	94.52%
	NR	20,736	96.30%
	Total Annotated	20,740	96.32%
	Total gene	21,532	100%
TG	Swiss-Prot	20,346	94.83%
	NR	20,735	96.64%
	Total Annotated	20,737	96.65%
	Total gene	21,455	100%

Table S10. Assessment of predicted protein-set completeness for the three *Procapra* species using BUSCO.

Species	Type	Number	Percent
MG	Complete BUSCOs (C)	9,068	98.30%
	Complete and single-copy BUSCOs (S)	8,937	96.90%
	Complete and duplicated BUSCOs (D)	131	1.40%
	Fragmented BUSCOs (F)	6	0.10%
	Missing BUSCOs (M)	152	1.60%
	Total BUSCO groups searched	9,226	100.00%
PG	Complete BUSCOs (C)	9,117	98.80%
	Complete and single-copy BUSCOs (S)	8,975	97.30%
	Complete and duplicated BUSCOs (D)	142	1.50%
	Fragmented BUSCOs (F)	16	0.20%
	Missing BUSCOs (M)	93	1.00%
	Total BUSCO groups searched	9,226	100.00%
TG	Complete BUSCOs (C)	9,129	99.00%
	Complete and single-copy BUSCOs (S)	9,001	97.60%
	Complete and duplicated BUSCOs (D)	128	1.40%
	Fragmented BUSCOs (F)	6	0.10%
	Missing BUSCOs (M)	91	0.99%
	Total BUSCO groups searched	9,226	100.00%

Table S11. Phylogenetic information of *Procapra* species and related taxa.

Scientific name	English name	RefSeq accession	Genbank accession
Tragulus kanchil	lesser mouse-deer	NA	GCA_022376925.1
Antidorcas marsupialis	springbok	NA	GCA_006408585.1
Bos mutus	wild yak	GCF_027580195.1	GCA_027580195.2
Budorcas tibetana	takin	GCF_023091745.1	GCA_023091745.2
Capra hircus	goat	GCF_001704415.2	GCA_001704415.2
Connochaetes taurinus	brindled gnu	NA	GCA_006408615.1
Elaphurus davidianus	Pere David's deer	NA	GCA_021018665.1
Eudorcas thomsonii	Thomson's gazelle	NA	GCA_006408755.1
Giraffa camelopardalis rothschildi	Giraffa	NA	GCA_017591445.1
Kobus ellipsiprymnus	waterbuck	NA	GCA_006410655.1
Litocranius walleri	gerenuk	NA	GCA_006410535.1
Oryx dammah	scimitar-horned oryx	GCF_014754425.2	GCA_014754425.2
Pantholops hodgsonii	chiru	NA	GCA_040182635.1
Philantomba maxwellii	Maxwell's duiker	NA	GCA_006410695.1
Raphicerus campestris	steenbok	NA	GCA_006410735.1
Procapra gutturosa	Mongolian gazelle	This study	This study
Procapra przewalskii	Przewalski’s gazelle	This study	This study
Procapra picticaudata	Tibetan gazelle	This study	This study

Table S12. Fossil-calibrated time points used for divergence-time estimation.

Node	Node minimum age (Ma)	Node maximum age (Ma)
Tragulus kanchil – Pecora split	38.3	49.8
Giraffa camelopardalis – (Cervidae + Bovidae) split	18.7	33.6
Cervidae – Bovidae split	18.5	27.8
Bos mutus – remaining Bovidae split	19.1	30.8
Kobus ellipsiprymnus – *Philantomba maxwellii* split	13	29.1
Pantholops hodgsonii – (*Budorcas* + *Capra*) split	6.6	11.6
Connochaetes taurinus – *Oryx dammah* split	11.3	13.2
Budorcas – *Capra hircus* split	5.7	11.7
Antidorcas marsupialis – *Litocranius walleri* split	7	8.3
Eudorcas thomsonii – (*Antidorcas marsupialis* + *Litocranius walleri*) split	7.9	12.1

Table S13. Summary information of the whole genome resequencing data.

	Species	Sample ID	Sex	Alignment to their respective genomes			Alignment to the PG reference genome		
				Mapping rate	Depth (×)	Coverage	Mapping rate	Depth (×)	Coverage
1	EHL	EHL07	Male	*NA*	*NA*	*NA*	98.47%	19.38	93.03%
2	MG	MY01	Male	99.90%	13.2	99.39%	99.90%	13.35	99.42%
3	MG	MY02	Female	99.93%	13.38	98.85%	99.93%	13.53	98.94%
4	MG	MY03	Male	99.85%	12.79	99.38%	99.85%	12.93	99.41%
5	MG	MY04	Male	99.91%	16.37	99.37%	99.92%	16.55	99.38%
6	MG	MY05	Male	99.89%	13.61	99.42%	99.89%	13.77	99.45%
7	MG	MY06	Female	99.94%	13.01	98.89%	99.94%	13.15	99.01%
8	MG	MY08	Female	99.91%	11.03	98.83%	99.91%	11.16	98.91%
9	MG	MY10	Male	99.93%	12.97	99.44%	99.93%	13.12	99.36%
10	MG	MY11	Male	99.94%	12.69	99.41%	99.94%	12.84	99.39%
11	MG	MY16	Male	99.91%	15.25	99.47%	99.91%	15.42	99.47%
12	MG	MY18	Male	99.29%	14.74	99.38%	99.29%	14.91	99.42%
13	MG	MY19	Male	97.53%	12.92	99.32%	97.53%	13.07	99.33%
14	MG	MY22	Female	99.90%	13.25	98.91%	99.90%	13.4	99.02%
15	MG	MY24	Female	96.31%	16.15	99.22%	96.29%	16.33	99.30%
16	MG	MY26	Male	99.80%	13.37	99.37%	99.80%	13.52	99.42%
17	MG	MY28	Male	99.95%	13.99	99.35%	99.95%	14.15	99.37%
18	MG	MY32	Male	83.99%	11.84	98.65%	83.93%	11.97	98.61%
19	MG	MY35	Female	99.79%	14.23	98.78%	99.79%	14.39	98.99%
20	MG	MY36	Male	99.93%	10.59	98.67%	99.93%	10.72	98.64%
21	MG	MY37	Male	99.88%	11.47	99.30%	99.88%	11.6	99.26%

22	MG	MY38	Female	99.96%	12.92	98.76%	99.96%	13.06	98.89%
23	MG	MY39	Male	99.56%	12.96	99.35%	99.56%	13.11	99.38%
24	MG	MY41	Female	99.85%	11.9	98.83%	99.85%	12.03	99.00%
25	MG	MY42	Female	99.84%	12.58	98.78%	99.84%	12.73	98.92%
26	MG	MY43	Female	99.91%	13.06	98.86%	99.91%	13.21	98.95%
27	MG	MY44	Male	99.95%	11.17	99.19%	99.95%	11.3	99.15%
28	MG	MY45	Female	99.95%	14.51	98.79%	99.95%	14.68	98.93%
29	MG	MY46	Female	99.74%	12.54	98.70%	99.74%	12.68	98.83%
30	MG	MY47	Female	99.89%	14.38	98.86%	99.89%	14.55	99.01%
31	MG	MY48	Male	99.93%	10.87	99.17%	99.93%	10.99	99.20%
32	MG	MY49	Female	99.94%	12.1	98.83%	99.94%	12.24	98.94%
33	MG	MY50	Female	99.81%	11.13	98.67%	99.81%	11.25	98.89%
34	PG	PY02	Male	99.96%	19.34	99.60%	99.96%	19.34	99.60%
35	PG	PY05	Male	99.78%	15.34	99.22%	99.78%	15.34	99.22%
36	PG	PY06	Female	99.94%	16.45	99.15%	99.94%	16.45	99.15%
37	PG	PY10	Female	99.94%	46.23	99.37%	99.94%	46.23	99.37%
38	PG	PY17	Female	99.86%	12.28	99.00%	99.86%	12.28	99.00%
39	PG	PY22	Female	89.15%	34.18	99.20%	89.15%	34.18	99.20%
40	PG	PY23	Female	98.65%	15.78	99.16%	98.65%	15.78	99.16%
41	PG	PY24	Male	99.36%	10.76	99.02%	99.36%	10.76	99.02%
42	PG	PY25	Male	99.95%	42.08	99.76%	99.95%	42.08	99.76%
43	PG	PY26	Female	99.95%	13.44	99.12%	99.95%	13.44	99.12%
44	PG	PY27	Male	99.54%	11.84	99.82%	99.54%	11.84	99.82%
45	PG	PY28	Male	99.96%	11.57	99.52%	99.96%	11.57	99.52%
46	PG	PY29	Male	99.97%	38.66	99.75%	99.97%	38.66	99.75%

47	PG	PY30	Female	99.97%	40.3	99.27%	99.97%	40.3	99.27%
48	PG	PY31	Female	99.96%	14.68	99.09%	99.96%	14.68	99.09%
49	PG	PY32	Female	99.85%	15.81	99.16%	99.85%	15.81	99.16%
50	PG	PY33	Male	99.96%	30.16	99.64%	99.96%	30.16	99.64%
51	PG	PY34	Male	96.15%	38.01	99.75%	96.15%	38.01	99.75%
52	PG	PY36	Female	99.96%	47.25	99.26%	99.96%	47.25	99.26%
53	PG	PY37	Female	99.91%	13.39	99.11%	99.91%	13.39	99.11%
54	PG	PY39	Female	99.97%	49.04	99.24%	99.97%	49.04	99.24%
55	PG	PY40	Male	99.93%	12.97	98.57%	99.93%	12.97	98.57%
56	PG	PY42	Male	99.96%	11.91	99.42%	99.96%	11.91	99.42%
57	PG	PY43	Male	99.94%	49.32	99.70%	99.94%	49.32	99.70%
58	PG	PY44	Female	99.92%	45.99	99.35%	99.92%	45.99	99.35%
59	PG	PY45	Male	99.96%	13.19	99.55%	99.96%	13.19	99.55%
60	PG	PY46	Female	99.93%	12.31	99.11%	99.93%	12.31	99.11%
61	PG	PY47	Male	99.97%	12.7	99.57%	99.97%	12.7	99.57%
62	PG	PY48	Male	99.95%	49.41	99.72%	99.95%	49.41	99.72%
63	PG	PY49	Female	99.97%	35.05	99.29%	99.97%	35.05	99.29%
64	PG	PY50	Female	99.98%	14.16	99.15%	99.98%	14.16	99.15%
65	PG	PY51	Male	99.96%	11.53	99.45%	99.96%	11.53	99.45%
66	PG	PY52	Female	99.96%	14.23	99.05%	99.96%	14.23	99.05%
67	PG	PY53	Male	99.93%	11.03	99.34%	99.93%	11.03	99.34%
68	PG	PY54	Male	99.76%	13.52	99.43%	99.76%	13.52	99.43%
69	PG	PY55	Male	99.85%	14.03	99.57%	99.85%	14.03	99.57%
70	PG	PY56	Female	99.95%	13.98	99.16%	99.95%	13.98	99.16%
71	PG	PY57	Male	99.77%	15.37	99.44%	99.77%	15.37	99.44%

72	PG	PY58	Male	99.91%	49.74	99.72%	99.91%	49.74	99.72%
73	PG	PY59	Female	99.94%	11.73	99.16%	99.94%	11.73	99.16%
74	PG	PY60	Female	99.81%	13.97	99.16%	99.81%	13.97	99.16%
75	PG	PY61	Female	98.41%	12.02	98.84%	98.41%	12.02	98.84%
76	PG	PY62	Female	99.94%	13.16	99.14%	99.94%	13.16	99.14%
77	PG	PY63	Male	99.97%	12.59	99.45%	99.97%	12.59	99.45%
78	PG	PY67	Female	99.95%	40.6	99.21%	99.95%	40.6	99.21%
79	PG	PY68	Male	99.90%	50.91	99.67%	99.90%	50.91	99.67%
80	PG	PY69	Male	99.95%	15.87	99.53%	99.95%	15.87	99.53%
81	PG	PY70	Male	93.35%	14.46	99.49%	93.35%	14.46	99.49%
82	PG	PY71	Female	99.87%	15.72	99.13%	99.87%	15.72	99.13%
83	TG	ZY02	Male	98.71%	12.81	98.33%	98.68%	13.97	97.95%
84	TG	ZY04	Male	99.25%	14.73	98.79%	99.22%	16.06	98.15%
85	TG	ZY06	Female	99.94%	14.04	97.53%	99.91%	15.31	97.65%
86	TG	ZY07	Male	99.94%	11.72	98.13%	99.92%	12.78	97.69%
87	TG	ZY08	Female	99.96%	13.89	97.44%	99.92%	15.15	97.57%
88	TG	ZY10	Male	99.96%	11.82	98.05%	99.93%	12.89	97.87%
89	TG	ZY16	Female	99.98%	10.82	97.53%	99.94%	11.8	97.40%
90	TG	ZY17	Male	99.89%	42.92	99.47%	99.85%	46.78	98.24%
91	TG	ZY18	Male	99.98%	14.55	97.86%	99.96%	15.86	96.43%
92	TG	ZY19	Male	99.93%	14.31	98.34%	99.91%	15.6	96.90%
93	TG	ZY20	Female	99.90%	42.09	98.56%	99.86%	45.89	97.79%
94	TG	ZY21	Male	99.83%	43.8	99.38%	99.79%	47.74	98.55%

Table S14. Second- and third-degree related pairs detected among PG individuals using KING.

Individual 1	Individual 2	No. SNPs	Kinship coefficient	Inferred relationship	Final decision
PY33	PY63	18,252,250	0.1725	Second-degree	Retain PY33; remove PY63
PY25	PY28	18,248,050	0.0985	Second-degree	Retain PY25; remove PY28
PY29	PY30	18,298,752	0.0921	Second-degree	Retain PY29; remove PY30
PY22	PY23	18,254,975	0.0681	Third-degree	Retain PY23; remove PY22
PY68	PY69	18,258,834	0.0579	Third-degree	Retain PY68; remove PY69
PY67	PY69	18,256,642	0.0551	Third-degree	Remove both
PY67	PY68	18,280,228	0.0521	Third-degree	Retain PY68; remove PY67

Table S15. Results of qpDstat analysis across all three species.

Pop1	Pop2	Pop3	Outgroup	*D*-statistic	SE	Z
MG	PG	TG	EHL	-0.1662	0.001954	-85.071
MG	TG	PG	EHL	0.4187	0.003407	100
PG	MG	TG	EHL	0.1662	0.001954	85.071
PG	TG	MG	EHL	0.5469	0.002618	100
TG	MG	PG	EHL	-0.4187	0.003407	-100
TG	PG	MG	EHL	-0.5469	0.002618	-100

Table S16. Results of qpDstat analysis across PG and TG individuals.

Pop1	Pop2	Pop3	Pop4	*D*-statistic	SE	Z	Pop1	Pop2	Pop3	Pop4	*D*-statistic	SE	Z
PY02	MG	ZY02	EHL	0.1494	0.003235	46.184	PY44	MG	ZY16	EHL	0.163	0.00305	53.424
PY02	MG	ZY04	EHL	0.1413	0.00321	44.022	PY44	MG	ZY17	EHL	0.1654	0.002978	55.552
PY02	MG	ZY06	EHL	0.1475	0.003207	46	PY44	MG	ZY18	EHL	0.1685	0.003003	56.102
PY02	MG	ZY07	EHL	0.1501	0.003307	45.383	PY44	MG	ZY19	EHL	0.1677	0.002949	56.85
PY02	MG	ZY08	EHL	0.1485	0.003272	45.4	PY44	MG	ZY20	EHL	0.1662	0.002968	55.993
PY02	MG	ZY10	EHL	0.1491	0.003215	46.37	PY44	MG	ZY21	EHL	0.1622	0.003017	53.746
PY02	MG	ZY16	EHL	0.1422	0.003258	43.651	PY45	MG	ZY02	EHL	0.1761	0.003357	52.46
PY02	MG	ZY17	EHL	0.1457	0.003297	44.208	PY45	MG	ZY04	EHL	0.169	0.003251	51.978
PY02	MG	ZY18	EHL	0.148	0.003391	43.636	PY45	MG	ZY06	EHL	0.1766	0.003326	53.104
PY02	MG	ZY19	EHL	0.1477	0.003257	45.336	PY45	MG	ZY07	EHL	0.1765	0.003362	52.486
PY02	MG	ZY20	EHL	0.1454	0.003324	43.73	PY45	MG	ZY08	EHL	0.1766	0.003357	52.607
PY02	MG	ZY21	EHL	0.142	0.0032	44.374	PY45	MG	ZY10	EHL	0.1768	0.003336	53.01
PY05	MG	ZY02	EHL	0.1554	0.003544	43.844	PY45	MG	ZY16	EHL	0.1679	0.003428	48.974
PY05	MG	ZY04	EHL	0.1497	0.003484	42.984	PY45	MG	ZY17	EHL	0.172	0.003413	50.391
PY05	MG	ZY06	EHL	0.1543	0.003515	43.912	PY45	MG	ZY18	EHL	0.1746	0.003375	51.74
PY05	MG	ZY07	EHL	0.156	0.003539	44.081	PY45	MG	ZY19	EHL	0.1731	0.003406	50.817
PY05	MG	ZY08	EHL	0.155	0.003534	43.867	PY45	MG	ZY20	EHL	0.1709	0.003432	49.79
PY05	MG	ZY10	EHL	0.1558	0.003522	44.244	PY45	MG	ZY21	EHL	0.1686	0.003345	50.412
PY05	MG	ZY16	EHL	0.1476	0.003516	41.997	PY46	MG	ZY02	EHL	0.1749	0.003229	54.15
PY05	MG	ZY17	EHL	0.1545	0.003674	42.068	PY46	MG	ZY04	EHL	0.1667	0.003127	53.291
PY05	MG	ZY18	EHL	0.1582	0.003712	42.613	PY46	MG	ZY06	EHL	0.1755	0.003188	55.047
PY05	MG	ZY19	EHL	0.156	0.003687	42.299	PY46	MG	ZY07	EHL	0.1751	0.003091	56.656

PY05	MG	ZY20	EHL	0.1522	0.003633	41.906	PY46	MG	ZY08	EHL	0.176	0.003142	56.015
PY05	MG	ZY21	EHL	0.1482	0.003539	41.879	PY46	MG	ZY10	EHL	0.1757	0.003185	55.168
PY06	MG	ZY02	EHL	0.1549	0.003578	43.28	PY46	MG	ZY16	EHL	0.168	0.003052	55.052
PY06	MG	ZY04	EHL	0.1499	0.00352	42.583	PY46	MG	ZY17	EHL	0.1704	0.003139	54.3
PY06	MG	ZY06	EHL	0.1541	0.003542	43.519	PY46	MG	ZY18	EHL	0.1733	0.003182	54.482
PY06	MG	ZY07	EHL	0.1546	0.003566	43.357	PY46	MG	ZY19	EHL	0.1715	0.003098	55.352
PY06	MG	ZY08	EHL	0.1547	0.003543	43.672	PY46	MG	ZY20	EHL	0.1718	0.003083	55.723
PY06	MG	ZY10	EHL	0.154	0.003544	43.462	PY46	MG	ZY21	EHL	0.1671	0.003047	54.819
PY06	MG	ZY16	EHL	0.1481	0.003577	41.398	PY47	MG	ZY02	EHL	0.1811	0.00324	55.882
PY06	MG	ZY17	EHL	0.1532	0.003741	40.949	PY47	MG	ZY04	EHL	0.173	0.003092	55.943
PY06	MG	ZY18	EHL	0.1559	0.003776	41.281	PY47	MG	ZY06	EHL	0.1797	0.003186	56.417
PY06	MG	ZY19	EHL	0.1557	0.003745	41.571	PY47	MG	ZY07	EHL	0.1804	0.003152	57.254
PY06	MG	ZY20	EHL	0.152	0.003742	40.623	PY47	MG	ZY08	EHL	0.1809	0.003198	56.567
PY06	MG	ZY21	EHL	0.1482	0.003544	41.819	PY47	MG	ZY10	EHL	0.1803	0.003221	55.964
PY10	MG	ZY02	EHL	0.1534	0.003197	47.988	PY47	MG	ZY16	EHL	0.1723	0.0032	53.849
PY10	MG	ZY04	EHL	0.1468	0.003184	46.109	PY47	MG	ZY17	EHL	0.1739	0.003132	55.515
PY10	MG	ZY06	EHL	0.1534	0.003225	47.57	PY47	MG	ZY18	EHL	0.1764	0.003207	54.993
PY10	MG	ZY07	EHL	0.1538	0.003263	47.138	PY47	MG	ZY19	EHL	0.1761	0.003205	54.928
PY10	MG	ZY08	EHL	0.1533	0.00324	47.307	PY47	MG	ZY20	EHL	0.1745	0.003126	55.818
PY10	MG	ZY10	EHL	0.1543	0.00324	47.617	PY47	MG	ZY21	EHL	0.172	0.003137	54.818
PY10	MG	ZY16	EHL	0.1475	0.00329	44.834	PY48	MG	ZY02	EHL	0.1794	0.003132	57.264
PY10	MG	ZY17	EHL	0.1519	0.003336	45.539	PY48	MG	ZY04	EHL	0.1729	0.003064	56.428
PY10	MG	ZY18	EHL	0.1536	0.003365	45.645	PY48	MG	ZY06	EHL	0.1794	0.00313	57.32
PY10	MG	ZY19	EHL	0.1533	0.003373	45.46	PY48	MG	ZY07	EHL	0.1804	0.003113	57.94
PY10	MG	ZY20	EHL	0.1497	0.003284	45.568	PY48	MG	ZY08	EHL	0.18	0.003117	57.756

PY10	MG	ZY21	EHL	0.1469	0.003136	46.847	PY48	MG	ZY10	EHL	0.1803	0.003123	57.736
PY17	MG	ZY02	EHL	0.1154	0.00297	38.843	PY48	MG	ZY16	EHL	0.1719	0.003087	55.693
PY17	MG	ZY04	EHL	0.1111	0.002939	37.811	PY48	MG	ZY17	EHL	0.1741	0.003104	56.097
PY17	MG	ZY06	EHL	0.1152	0.002996	38.459	PY48	MG	ZY18	EHL	0.1767	0.00323	54.71
PY17	MG	ZY07	EHL	0.1148	0.002949	38.919	PY48	MG	ZY19	EHL	0.1747	0.003184	54.865
PY17	MG	ZY08	EHL	0.1145	0.002977	38.467	PY48	MG	ZY20	EHL	0.1738	0.003164	54.921
PY17	MG	ZY10	EHL	0.1146	0.002967	38.62	PY48	MG	ZY21	EHL	0.1724	0.003093	55.723
PY17	MG	ZY16	EHL	0.1094	0.002922	37.437	PY49	MG	ZY02	EHL	0.1755	0.003208	54.695
PY17	MG	ZY17	EHL	0.1138	0.002937	38.761	PY49	MG	ZY04	EHL	0.1676	0.003239	51.744
PY17	MG	ZY18	EHL	0.1156	0.003082	37.526	PY49	MG	ZY06	EHL	0.1758	0.003222	54.555
PY17	MG	ZY19	EHL	0.1151	0.002933	39.247	PY49	MG	ZY07	EHL	0.1751	0.003217	54.44
PY17	MG	ZY20	EHL	0.1133	0.002956	38.328	PY49	MG	ZY08	EHL	0.1755	0.003269	53.675
PY17	MG	ZY21	EHL	0.1104	0.002975	37.107	PY49	MG	ZY10	EHL	0.1746	0.00322	54.232
PY22	MG	ZY02	EHL	0.1659	0.003081	53.853	PY49	MG	ZY16	EHL	0.1674	0.003322	50.391
PY22	MG	ZY04	EHL	0.1594	0.002982	53.475	PY49	MG	ZY17	EHL	0.17	0.00338	50.304
PY22	MG	ZY06	EHL	0.1669	0.003036	54.978	PY49	MG	ZY18	EHL	0.1724	0.0034	50.712
PY22	MG	ZY07	EHL	0.1658	0.003095	53.577	PY49	MG	ZY19	EHL	0.1723	0.003348	51.457
PY22	MG	ZY08	EHL	0.1674	0.003059	54.736	PY49	MG	ZY20	EHL	0.17	0.003297	51.558
PY22	MG	ZY10	EHL	0.1658	0.003085	53.755	PY49	MG	ZY21	EHL	0.1675	0.003257	51.439
PY22	MG	ZY16	EHL	0.1595	0.003049	52.311	PY50	MG	ZY02	EHL	0.1703	0.003119	54.604
PY22	MG	ZY17	EHL	0.1627	0.002958	55.01	PY50	MG	ZY04	EHL	0.1627	0.002997	54.279
PY22	MG	ZY18	EHL	0.1646	0.003105	53.002	PY50	MG	ZY06	EHL	0.1704	0.003091	55.13
PY22	MG	ZY19	EHL	0.1634	0.003033	53.868	PY50	MG	ZY07	EHL	0.1715	0.003112	55.086
PY22	MG	ZY20	EHL	0.1628	0.002958	55.042	PY50	MG	ZY08	EHL	0.1708	0.003161	54.034
PY22	MG	ZY21	EHL	0.1591	0.003024	52.608	PY50	MG	ZY10	EHL	0.1706	0.003158	54.037

PY23	MG	ZY02	EHL	0.1636	0.003127	52.32	PY50	MG	ZY16	EHL	0.1622	0.003108	52.18
PY23	MG	ZY04	EHL	0.1581	0.003002	52.673	PY50	MG	ZY17	EHL	0.1662	0.002997	55.477
PY23	MG	ZY06	EHL	0.1646	0.003062	53.753	PY50	MG	ZY18	EHL	0.1692	0.003167	53.443
PY23	MG	ZY07	EHL	0.1647	0.00312	52.802	PY50	MG	ZY19	EHL	0.1683	0.002994	56.215
PY23	MG	ZY08	EHL	0.1641	0.003072	53.415	PY50	MG	ZY20	EHL	0.1662	0.002981	55.769
PY23	MG	ZY10	EHL	0.1632	0.003124	52.248	PY50	MG	ZY21	EHL	0.1633	0.003017	54.129
PY23	MG	ZY16	EHL	0.1574	0.003089	50.971	PY51	MG	ZY02	EHL	0.1719	0.00319	53.886
PY23	MG	ZY17	EHL	0.1605	0.003119	51.454	PY51	MG	ZY04	EHL	0.1633	0.003134	52.115
PY23	MG	ZY18	EHL	0.1625	0.003128	51.958	PY51	MG	ZY06	EHL	0.1715	0.003203	53.528
PY23	MG	ZY19	EHL	0.1616	0.003067	52.669	PY51	MG	ZY07	EHL	0.1725	0.003199	53.907
PY23	MG	ZY20	EHL	0.1604	0.003081	52.061	PY51	MG	ZY08	EHL	0.1714	0.00319	53.75
PY23	MG	ZY21	EHL	0.157	0.003047	51.517	PY51	MG	ZY10	EHL	0.1707	0.003186	53.58
PY24	MG	ZY02	EHL	0.1674	0.003078	54.386	PY51	MG	ZY16	EHL	0.163	0.003159	51.599
PY24	MG	ZY04	EHL	0.1609	0.003083	52.171	PY51	MG	ZY17	EHL	0.1654	0.003223	51.312
PY24	MG	ZY06	EHL	0.1676	0.003082	54.386	PY51	MG	ZY18	EHL	0.1698	0.003236	52.484
PY24	MG	ZY07	EHL	0.1695	0.003176	53.378	PY51	MG	ZY19	EHL	0.1688	0.003191	52.915
PY24	MG	ZY08	EHL	0.1677	0.003066	54.673	PY51	MG	ZY20	EHL	0.1669	0.0032	52.173
PY24	MG	ZY10	EHL	0.1686	0.0031	54.376	PY51	MG	ZY21	EHL	0.1627	0.00312	52.145
PY24	MG	ZY16	EHL	0.1608	0.003095	51.943	PY52	MG	ZY02	EHL	0.1754	0.003159	55.533
PY24	MG	ZY17	EHL	0.1619	0.003103	52.171	PY52	MG	ZY04	EHL	0.1658	0.003026	54.79
PY24	MG	ZY18	EHL	0.1674	0.003275	51.109	PY52	MG	ZY06	EHL	0.1745	0.003132	55.703
PY24	MG	ZY19	EHL	0.1655	0.003154	52.452	PY52	MG	ZY07	EHL	0.1752	0.003126	56.049
PY24	MG	ZY20	EHL	0.1632	0.003163	51.586	PY52	MG	ZY08	EHL	0.1748	0.003132	55.823
PY24	MG	ZY21	EHL	0.1602	0.003051	52.495	PY52	MG	ZY10	EHL	0.1756	0.003107	56.535
PY25	MG	ZY02	EHL	0.171	0.003258	52.481	PY52	MG	ZY16	EHL	0.1665	0.003135	53.086

PY25	MG	ZY04	EHL	0.1635	0.003182	51.384	PY52	MG	ZY17	EHL	0.1699	0.00309	54.986
PY25	MG	ZY06	EHL	0.171	0.003271	52.287	PY52	MG	ZY18	EHL	0.1722	0.003165	54.392
PY25	MG	ZY07	EHL	0.1719	0.003291	52.247	PY52	MG	ZY19	EHL	0.1719	0.0031	55.468
PY25	MG	ZY08	EHL	0.1715	0.003264	52.549	PY52	MG	ZY20	EHL	0.1693	0.003045	55.603
PY25	MG	ZY10	EHL	0.1712	0.003261	52.507	PY52	MG	ZY21	EHL	0.1662	0.003038	54.706
PY25	MG	ZY16	EHL	0.1633	0.003296	49.544	PY53	MG	ZY02	EHL	0.1793	0.003227	55.581
PY25	MG	ZY17	EHL	0.1664	0.003236	51.42	PY53	MG	ZY04	EHL	0.1717	0.003118	55.05
PY25	MG	ZY18	EHL	0.1691	0.003297	51.296	PY53	MG	ZY06	EHL	0.1789	0.003243	55.163
PY25	MG	ZY19	EHL	0.1676	0.003274	51.181	PY53	MG	ZY07	EHL	0.1797	0.003217	55.862
PY25	MG	ZY20	EHL	0.1653	0.003224	51.266	PY53	MG	ZY08	EHL	0.1795	0.003246	55.292
PY25	MG	ZY21	EHL	0.1641	0.003253	50.438	PY53	MG	ZY10	EHL	0.1796	0.003233	55.564
PY26	MG	ZY02	EHL	0.1705	0.003175	53.695	PY53	MG	ZY16	EHL	0.1711	0.00321	53.297
PY26	MG	ZY04	EHL	0.1613	0.003048	52.905	PY53	MG	ZY17	EHL	0.1731	0.003304	52.392
PY26	MG	ZY06	EHL	0.1706	0.003161	53.976	PY53	MG	ZY18	EHL	0.1774	0.003292	53.909
PY26	MG	ZY07	EHL	0.171	0.003168	53.958	PY53	MG	ZY19	EHL	0.1753	0.0033	53.119
PY26	MG	ZY08	EHL	0.1707	0.003145	54.262	PY53	MG	ZY20	EHL	0.1739	0.003172	54.824
PY26	MG	ZY10	EHL	0.1709	0.003157	54.138	PY53	MG	ZY21	EHL	0.1713	0.003201	53.531
PY26	MG	ZY16	EHL	0.1623	0.003157	51.404	PY54	MG	ZY02	EHL	0.1789	0.003154	56.711
PY26	MG	ZY17	EHL	0.1641	0.003109	52.795	PY54	MG	ZY04	EHL	0.17	0.00295	57.624
PY26	MG	ZY18	EHL	0.1683	0.003254	51.716	PY54	MG	ZY06	EHL	0.1786	0.003106	57.507
PY26	MG	ZY19	EHL	0.1668	0.003099	53.824	PY54	MG	ZY07	EHL	0.1783	0.003066	58.14
PY26	MG	ZY20	EHL	0.1659	0.003124	53.094	PY54	MG	ZY08	EHL	0.1781	0.0031	57.44
PY26	MG	ZY21	EHL	0.1622	0.00312	52.005	PY54	MG	ZY10	EHL	0.179	0.00314	57.01
PY27	MG	ZY02	EHL	0.1477	0.003172	46.573	PY54	MG	ZY16	EHL	0.1696	0.003069	55.267
PY27	MG	ZY04	EHL	0.1414	0.003059	46.22	PY54	MG	ZY17	EHL	0.1729	0.003118	55.452

PY27	MG	ZY06	EHL	0.1479	0.003111	47.553	PY54	MG	ZY18	EHL	0.1758	0.003157	55.684
PY27	MG	ZY07	EHL	0.151	0.003148	47.961	PY54	MG	ZY19	EHL	0.1746	0.003084	56.628
PY27	MG	ZY08	EHL	0.1469	0.003162	46.441	PY54	MG	ZY20	EHL	0.1722	0.003139	54.849
PY27	MG	ZY10	EHL	0.1487	0.003132	47.482	PY54	MG	ZY21	EHL	0.17	0.003032	56.06
PY27	MG	ZY16	EHL	0.1407	0.003132	44.93	PY55	MG	ZY02	EHL	0.173	0.003203	54.017
PY27	MG	ZY17	EHL	0.1428	0.003132	45.607	PY55	MG	ZY04	EHL	0.1643	0.003061	53.682
PY27	MG	ZY18	EHL	0.1533	0.003229	47.472	PY55	MG	ZY06	EHL	0.1724	0.00312	55.267
PY27	MG	ZY19	EHL	0.1507	0.003079	48.943	PY55	MG	ZY07	EHL	0.1735	0.003207	54.091
PY27	MG	ZY20	EHL	0.1434	0.003081	46.536	PY55	MG	ZY08	EHL	0.1732	0.003171	54.605
PY27	MG	ZY21	EHL	0.1391	0.003042	45.711	PY55	MG	ZY10	EHL	0.1733	0.003193	54.282
PY28	MG	ZY02	EHL	0.1794	0.003208	55.931	PY55	MG	ZY16	EHL	0.1642	0.003123	52.599
PY28	MG	ZY04	EHL	0.1704	0.00312	54.608	PY55	MG	ZY17	EHL	0.168	0.003041	55.256
PY28	MG	ZY06	EHL	0.1797	0.003192	56.301	PY55	MG	ZY18	EHL	0.1702	0.003139	54.223
PY28	MG	ZY07	EHL	0.1796	0.003257	55.137	PY55	MG	ZY19	EHL	0.1688	0.003053	55.286
PY28	MG	ZY08	EHL	0.1804	0.003172	56.883	PY55	MG	ZY20	EHL	0.1672	0.003007	55.61
PY28	MG	ZY10	EHL	0.1804	0.003184	56.657	PY55	MG	ZY21	EHL	0.1648	0.003041	54.194
PY28	MG	ZY16	EHL	0.1701	0.003129	54.358	PY56	MG	ZY02	EHL	0.1766	0.003248	54.354
PY28	MG	ZY17	EHL	0.1721	0.003173	54.241	PY56	MG	ZY04	EHL	0.1667	0.003072	54.278
PY28	MG	ZY18	EHL	0.1766	0.003224	54.779	PY56	MG	ZY06	EHL	0.1755	0.003198	54.884
PY28	MG	ZY19	EHL	0.1752	0.003193	54.876	PY56	MG	ZY07	EHL	0.1763	0.003189	55.296
PY28	MG	ZY20	EHL	0.1719	0.003132	54.896	PY56	MG	ZY08	EHL	0.176	0.003212	54.798
PY28	MG	ZY21	EHL	0.171	0.003095	55.244	PY56	MG	ZY10	EHL	0.1765	0.003215	54.921
PY29	MG	ZY02	EHL	0.177	0.003072	57.605	PY56	MG	ZY16	EHL	0.1669	0.003253	51.316
PY29	MG	ZY04	EHL	0.1692	0.003015	56.124	PY56	MG	ZY17	EHL	0.1694	0.003213	52.717
PY29	MG	ZY06	EHL	0.1763	0.003083	57.18	PY56	MG	ZY18	EHL	0.172	0.003207	53.633

PY29	MG	ZY07	EHL	0.1772	0.0031	57.149	PY56	MG	ZY19	EHL	0.172	0.003188	53.961
PY29	MG	ZY08	EHL	0.1773	0.003092	57.35	PY56	MG	ZY20	EHL	0.1699	0.003097	54.853
PY29	MG	ZY10	EHL	0.1765	0.003085	57.203	PY56	MG	ZY21	EHL	0.1677	0.003122	53.722
PY29	MG	ZY16	EHL	0.1685	0.003111	54.152	PY57	MG	ZY02	EHL	0.1774	0.003184	55.707
PY29	MG	ZY17	EHL	0.172	0.003067	56.083	PY57	MG	ZY04	EHL	0.1666	0.003152	52.852
PY29	MG	ZY18	EHL	0.1774	0.003123	56.813	PY57	MG	ZY06	EHL	0.1763	0.003158	55.832
PY29	MG	ZY19	EHL	0.1735	0.003007	57.701	PY57	MG	ZY07	EHL	0.1774	0.003156	56.225
PY29	MG	ZY20	EHL	0.1718	0.00304	56.505	PY57	MG	ZY08	EHL	0.176	0.003145	55.943
PY29	MG	ZY21	EHL	0.1695	0.002967	57.143	PY57	MG	ZY10	EHL	0.1772	0.003176	55.776
PY30	MG	ZY02	EHL	0.1719	0.003058	56.191	PY57	MG	ZY16	EHL	0.1662	0.003163	52.552
PY30	MG	ZY04	EHL	0.165	0.002953	55.87	PY57	MG	ZY17	EHL	0.1701	0.003161	53.804
PY30	MG	ZY06	EHL	0.1724	0.003102	55.573	PY57	MG	ZY18	EHL	0.1724	0.003217	53.588
PY30	MG	ZY07	EHL	0.173	0.003057	56.578	PY57	MG	ZY19	EHL	0.1732	0.00319	54.304
PY30	MG	ZY08	EHL	0.1722	0.003063	56.219	PY57	MG	ZY20	EHL	0.1695	0.003108	54.546
PY30	MG	ZY10	EHL	0.1716	0.003048	56.31	PY57	MG	ZY21	EHL	0.1683	0.00308	54.633
PY30	MG	ZY16	EHL	0.1649	0.003032	54.371	PY58	MG	ZY02	EHL	0.1795	0.003239	55.405
PY30	MG	ZY17	EHL	0.1671	0.00296	56.454	PY58	MG	ZY04	EHL	0.1715	0.003185	53.845
PY30	MG	ZY18	EHL	0.172	0.003033	56.723	PY58	MG	ZY06	EHL	0.1784	0.003267	54.599
PY30	MG	ZY19	EHL	0.1691	0.002993	56.508	PY58	MG	ZY07	EHL	0.1794	0.00325	55.195
PY30	MG	ZY20	EHL	0.1678	0.002932	57.237	PY58	MG	ZY08	EHL	0.1789	0.003297	54.255
PY30	MG	ZY21	EHL	0.1644	0.002988	55.016	PY58	MG	ZY10	EHL	0.1785	0.00332	53.755
PY31	MG	ZY02	EHL	0.1737	0.003284	52.892	PY58	MG	ZY16	EHL	0.1708	0.003239	52.722
PY31	MG	ZY04	EHL	0.1661	0.003223	51.543	PY58	MG	ZY17	EHL	0.1738	0.003193	54.441
PY31	MG	ZY06	EHL	0.1736	0.003242	53.544	PY58	MG	ZY18	EHL	0.1763	0.003099	56.888
PY31	MG	ZY07	EHL	0.1746	0.00331	52.755	PY58	MG	ZY19	EHL	0.1748	0.003193	54.754

PY31	MG	ZY08	EHL	0.1744	0.003275	53.248	PY58	MG	ZY20	EHL	0.1737	0.003119	55.674
PY31	MG	ZY10	EHL	0.1737	0.003234	53.71	PY58	MG	ZY21	EHL	0.1712	0.003133	54.654
PY31	MG	ZY16	EHL	0.1656	0.003304	50.131	PY59	MG	ZY02	EHL	0.1823	0.003147	57.939
PY31	MG	ZY17	EHL	0.1698	0.003297	51.491	PY59	MG	ZY04	EHL	0.1732	0.002955	58.62
PY31	MG	ZY18	EHL	0.1713	0.003403	50.333	PY59	MG	ZY06	EHL	0.1822	0.003128	58.254
PY31	MG	ZY19	EHL	0.1701	0.003397	50.061	PY59	MG	ZY07	EHL	0.1822	0.00314	58.012
PY31	MG	ZY20	EHL	0.1706	0.00327	52.18	PY59	MG	ZY08	EHL	0.1833	0.003107	58.998
PY31	MG	ZY21	EHL	0.1665	0.00326	51.078	PY59	MG	ZY10	EHL	0.1832	0.003168	57.839
PY32	MG	ZY02	EHL	0.1742	0.003451	50.491	PY59	MG	ZY16	EHL	0.1735	0.003089	56.171
PY32	MG	ZY04	EHL	0.166	0.003371	49.224	PY59	MG	ZY17	EHL	0.1764	0.003061	57.63
PY32	MG	ZY06	EHL	0.1735	0.003421	50.732	PY59	MG	ZY18	EHL	0.1787	0.003109	57.476
PY32	MG	ZY07	EHL	0.1741	0.003519	49.481	PY59	MG	ZY19	EHL	0.1778	0.003112	57.155
PY32	MG	ZY08	EHL	0.174	0.003447	50.471	PY59	MG	ZY20	EHL	0.1779	0.003047	58.381
PY32	MG	ZY10	EHL	0.1738	0.003446	50.437	PY59	MG	ZY21	EHL	0.1738	0.002999	57.943
PY32	MG	ZY16	EHL	0.1674	0.003378	49.557	PY60	MG	ZY02	EHL	0.1808	0.003156	57.295
PY32	MG	ZY17	EHL	0.1699	0.00343	49.526	PY60	MG	ZY04	EHL	0.1721	0.003014	57.098
PY32	MG	ZY18	EHL	0.1739	0.003526	49.312	PY60	MG	ZY06	EHL	0.18	0.003096	58.132
PY32	MG	ZY19	EHL	0.1722	0.003368	51.141	PY60	MG	ZY07	EHL	0.1799	0.003096	58.104
PY32	MG	ZY20	EHL	0.1697	0.003401	49.898	PY60	MG	ZY08	EHL	0.1802	0.003129	57.606
PY32	MG	ZY21	EHL	0.1662	0.003334	49.854	PY60	MG	ZY10	EHL	0.1805	0.003102	58.182
PY33	MG	ZY02	EHL	0.1757	0.003134	56.055	PY60	MG	ZY16	EHL	0.1719	0.003083	55.756
PY33	MG	ZY04	EHL	0.1681	0.003074	54.683	PY60	MG	ZY17	EHL	0.175	0.003093	56.573
PY33	MG	ZY06	EHL	0.1754	0.003119	56.235	PY60	MG	ZY18	EHL	0.1772	0.003158	56.12
PY33	MG	ZY07	EHL	0.1765	0.00314	56.201	PY60	MG	ZY19	EHL	0.1763	0.003157	55.855
PY33	MG	ZY08	EHL	0.1758	0.003108	56.579	PY60	MG	ZY20	EHL	0.176	0.003	58.654

PY33	MG	ZY10	EHL	0.1752	0.003091	56.67	PY60	MG	ZY21	EHL	0.1726	0.003084	55.968
PY33	MG	ZY16	EHL	0.1676	0.003147	53.249	PY61	MG	ZY02	EHL	0.1754	0.003111	56.374
PY33	MG	ZY17	EHL	0.1713	0.00312	54.89	PY61	MG	ZY04	EHL	0.1671	0.002962	56.415
PY33	MG	ZY18	EHL	0.1753	0.003218	54.487	PY61	MG	ZY06	EHL	0.174	0.003083	56.431
PY33	MG	ZY19	EHL	0.1721	0.003146	54.712	PY61	MG	ZY07	EHL	0.1752	0.003077	56.932
PY33	MG	ZY20	EHL	0.1713	0.003195	53.61	PY61	MG	ZY08	EHL	0.1753	0.003017	58.096
PY33	MG	ZY21	EHL	0.1681	0.003045	55.217	PY61	MG	ZY10	EHL	0.1749	0.003102	56.398
PY34	MG	ZY02	EHL	0.0999	0.0034	29.37	PY61	MG	ZY16	EHL	0.1659	0.003059	54.238
PY34	MG	ZY04	EHL	0.0936	0.003262	28.687	PY61	MG	ZY17	EHL	0.1685	0.002932	57.472
PY34	MG	ZY06	EHL	0.0988	0.003331	29.664	PY61	MG	ZY18	EHL	0.1719	0.003115	55.174
PY34	MG	ZY07	EHL	0.1009	0.003367	29.984	PY61	MG	ZY19	EHL	0.1711	0.003015	56.727
PY34	MG	ZY08	EHL	0.0996	0.003389	29.39	PY61	MG	ZY20	EHL	0.1685	0.002976	56.624
PY34	MG	ZY10	EHL	0.1009	0.003313	30.446	PY61	MG	ZY21	EHL	0.1671	0.002955	56.553
PY34	MG	ZY16	EHL	0.0923	0.003311	27.87	PY62	MG	ZY02	EHL	0.1789	0.003514	50.906
PY34	MG	ZY17	EHL	0.0989	0.003296	30.011	PY62	MG	ZY04	EHL	0.17	0.003374	50.398
PY34	MG	ZY18	EHL	0.1007	0.003325	30.297	PY62	MG	ZY06	EHL	0.1782	0.003407	52.302
PY34	MG	ZY19	EHL	0.0982	0.003366	29.165	PY62	MG	ZY07	EHL	0.1789	0.003452	51.811
PY34	MG	ZY20	EHL	0.0973	0.003247	29.981	PY62	MG	ZY08	EHL	0.1783	0.00345	51.685
PY34	MG	ZY21	EHL	0.0944	0.003287	28.72	PY62	MG	ZY10	EHL	0.1779	0.00344	51.721
PY36	MG	ZY02	EHL	0.1708	0.003277	52.121	PY62	MG	ZY16	EHL	0.1682	0.003459	48.617
PY36	MG	ZY04	EHL	0.164	0.003268	50.174	PY62	MG	ZY17	EHL	0.173	0.00345	50.13
PY36	MG	ZY06	EHL	0.172	0.003294	52.233	PY62	MG	ZY18	EHL	0.176	0.003467	50.768
PY36	MG	ZY07	EHL	0.1719	0.003369	51.033	PY62	MG	ZY19	EHL	0.1742	0.003395	51.329
PY36	MG	ZY08	EHL	0.1728	0.003268	52.883	PY62	MG	ZY20	EHL	0.1737	0.003393	51.2
PY36	MG	ZY10	EHL	0.1725	0.003243	53.173	PY62	MG	ZY21	EHL	0.1691	0.003392	49.86

PY36	MG	ZY16	EHL	0.1642	0.003265	50.296	PY63	MG	ZY02	EHL	0.1745	0.003219	54.189
PY36	MG	ZY17	EHL	0.1667	0.003335	49.988	PY63	MG	ZY04	EHL	0.1671	0.003157	52.936
PY36	MG	ZY18	EHL	0.1695	0.003476	48.766	PY63	MG	ZY06	EHL	0.1744	0.003163	55.126
PY36	MG	ZY19	EHL	0.1674	0.003418	48.984	PY63	MG	ZY07	EHL	0.1758	0.003206	54.829
PY36	MG	ZY20	EHL	0.1675	0.003335	50.226	PY63	MG	ZY08	EHL	0.1751	0.003197	54.786
PY36	MG	ZY21	EHL	0.1634	0.003273	49.917	PY63	MG	ZY10	EHL	0.1744	0.003184	54.757
PY37	MG	ZY02	EHL	0.1808	0.003422	52.831	PY63	MG	ZY16	EHL	0.1678	0.003135	53.53
PY37	MG	ZY04	EHL	0.1739	0.003203	54.286	PY63	MG	ZY17	EHL	0.1713	0.00327	52.374
PY37	MG	ZY06	EHL	0.1806	0.003284	54.99	PY63	MG	ZY18	EHL	0.1752	0.00331	52.95
PY37	MG	ZY07	EHL	0.1809	0.003324	54.416	PY63	MG	ZY19	EHL	0.1714	0.003229	53.076
PY37	MG	ZY08	EHL	0.1813	0.003304	54.877	PY63	MG	ZY20	EHL	0.1712	0.003251	52.65
PY37	MG	ZY10	EHL	0.1811	0.003335	54.315	PY63	MG	ZY21	EHL	0.1663	0.003151	52.773
PY37	MG	ZY16	EHL	0.1719	0.003294	52.175	PY67	MG	ZY02	EHL	0.1807	0.003338	54.142
PY37	MG	ZY17	EHL	0.1764	0.003302	53.402	PY67	MG	ZY04	EHL	0.1719	0.003159	54.433
PY37	MG	ZY18	EHL	0.1798	0.003361	53.487	PY67	MG	ZY06	EHL	0.1803	0.003208	56.189
PY37	MG	ZY19	EHL	0.1772	0.003327	53.262	PY67	MG	ZY07	EHL	0.18	0.003306	54.46
PY37	MG	ZY20	EHL	0.1749	0.00329	53.153	PY67	MG	ZY08	EHL	0.1807	0.003305	54.659
PY37	MG	ZY21	EHL	0.1728	0.003266	52.926	PY67	MG	ZY10	EHL	0.1804	0.003267	55.207
PY39	MG	ZY02	EHL	0.122	0.002892	42.194	PY67	MG	ZY16	EHL	0.1726	0.003199	53.943
PY39	MG	ZY04	EHL	0.1175	0.00279	42.097	PY67	MG	ZY17	EHL	0.1765	0.003275	53.902
PY39	MG	ZY06	EHL	0.1222	0.002847	42.911	PY67	MG	ZY18	EHL	0.1782	0.003244	54.929
PY39	MG	ZY07	EHL	0.1211	0.002836	42.691	PY67	MG	ZY19	EHL	0.1767	0.003239	54.564
PY39	MG	ZY08	EHL	0.1215	0.002912	41.731	PY67	MG	ZY20	EHL	0.1758	0.003169	55.457
PY39	MG	ZY10	EHL	0.1215	0.00286	42.469	PY67	MG	ZY21	EHL	0.1722	0.003209	53.672
PY39	MG	ZY16	EHL	0.1157	0.002915	39.681	PY68	MG	ZY02	EHL	0.1726	0.003208	53.785

PY39	MG	ZY17	EHL	0.1195	0.002893	41.319	PY68	MG	ZY04	EHL	0.1642	0.003068	53.538
PY39	MG	ZY18	EHL	0.1216	0.002873	42.333	PY68	MG	ZY06	EHL	0.1722	0.003132	54.957
PY39	MG	ZY19	EHL	0.1204	0.002832	42.535	PY68	MG	ZY07	EHL	0.1727	0.003173	54.42
PY39	MG	ZY20	EHL	0.1193	0.002831	42.152	PY68	MG	ZY08	EHL	0.1726	0.003182	54.256
PY39	MG	ZY21	EHL	0.117	0.002874	40.702	PY68	MG	ZY10	EHL	0.1735	0.003196	54.289
PY40	MG	ZY02	EHL	0.1823	0.003297	55.306	PY68	MG	ZY16	EHL	0.1646	0.003145	52.32
PY40	MG	ZY04	EHL	0.174	0.003231	53.857	PY68	MG	ZY17	EHL	0.1668	0.0031	53.809
PY40	MG	ZY06	EHL	0.1807	0.003256	55.502	PY68	MG	ZY18	EHL	0.1685	0.003144	53.598
PY40	MG	ZY07	EHL	0.1821	0.00332	54.843	PY68	MG	ZY19	EHL	0.1677	0.003052	54.962
PY40	MG	ZY08	EHL	0.1819	0.003301	55.091	PY68	MG	ZY20	EHL	0.166	0.003063	54.218
PY40	MG	ZY10	EHL	0.182	0.003305	55.05	PY68	MG	ZY21	EHL	0.1642	0.003147	52.168
PY40	MG	ZY16	EHL	0.173	0.003333	51.902	PY69	MG	ZY02	EHL	0.1779	0.00329	54.092
PY40	MG	ZY17	EHL	0.1771	0.003387	52.302	PY69	MG	ZY04	EHL	0.1696	0.003227	52.574
PY40	MG	ZY18	EHL	0.1806	0.003408	52.997	PY69	MG	ZY06	EHL	0.1765	0.003165	55.773
PY40	MG	ZY19	EHL	0.1802	0.003292	54.736	PY69	MG	ZY07	EHL	0.1768	0.003267	54.12
PY40	MG	ZY20	EHL	0.1768	0.003336	53.018	PY69	MG	ZY08	EHL	0.1777	0.003237	54.887
PY40	MG	ZY21	EHL	0.1735	0.003311	52.384	PY69	MG	ZY10	EHL	0.178	0.003278	54.304
PY42	MG	ZY02	EHL	0.1796	0.00326	55.106	PY69	MG	ZY16	EHL	0.169	0.003234	52.265
PY42	MG	ZY04	EHL	0.1721	0.003109	55.362	PY69	MG	ZY17	EHL	0.1723	0.003344	51.539
PY42	MG	ZY06	EHL	0.1792	0.003175	56.441	PY69	MG	ZY18	EHL	0.1751	0.003391	51.651
PY42	MG	ZY07	EHL	0.1795	0.003253	55.166	PY69	MG	ZY19	EHL	0.1745	0.003376	51.7
PY42	MG	ZY08	EHL	0.1801	0.003242	55.569	PY69	MG	ZY20	EHL	0.1727	0.003285	52.551
PY42	MG	ZY10	EHL	0.1801	0.003256	55.298	PY69	MG	ZY21	EHL	0.1698	0.003273	51.897
PY42	MG	ZY16	EHL	0.1717	0.003262	52.637	PY70	MG	ZY02	EHL	0.2034	0.005373	37.854
PY42	MG	ZY17	EHL	0.1742	0.003222	54.069	PY70	MG	ZY04	EHL	0.1994	0.005571	35.783

PY42	MG	ZY18	EHL	0.1778	0.00325	54.718	PY70	MG	ZY06	EHL	0.2025	0.005425	37.319
PY42	MG	ZY19	EHL	0.1748	0.003233	54.072	PY70	MG	ZY07	EHL	0.2037	0.005373	37.92
PY42	MG	ZY20	EHL	0.1725	0.003145	54.84	PY70	MG	ZY08	EHL	0.2036	0.005433	37.475
PY42	MG	ZY21	EHL	0.1722	0.003113	55.305	PY70	MG	ZY10	EHL	0.2026	0.005386	37.619
PY43	MG	ZY02	EHL	0.1746	0.003193	54.677	PY70	MG	ZY16	EHL	0.1988	0.005615	35.407
PY43	MG	ZY04	EHL	0.1665	0.003023	55.093	PY70	MG	ZY17	EHL	0.2108	0.006248	33.74
PY43	MG	ZY06	EHL	0.1739	0.003161	55.016	PY70	MG	ZY18	EHL	0.2134	0.006154	34.671
PY43	MG	ZY07	EHL	0.175	0.003171	55.191	PY70	MG	ZY19	EHL	0.2119	0.006222	34.062
PY43	MG	ZY08	EHL	0.1761	0.003154	55.83	PY70	MG	ZY20	EHL	0.2098	0.006207	33.803
PY43	MG	ZY10	EHL	0.1755	0.003161	55.509	PY70	MG	ZY21	EHL	0.2001	0.005604	35.716
PY43	MG	ZY16	EHL	0.1667	0.003124	53.366	PY71	MG	ZY02	EHL	0.152	0.003547	42.866
PY43	MG	ZY17	EHL	0.1705	0.003075	55.457	PY71	MG	ZY04	EHL	0.1452	0.003384	42.896
PY43	MG	ZY18	EHL	0.1728	0.003167	54.546	PY71	MG	ZY06	EHL	0.1521	0.003501	43.439
PY43	MG	ZY19	EHL	0.1715	0.003156	54.337	PY71	MG	ZY07	EHL	0.1517	0.00359	42.25
PY43	MG	ZY20	EHL	0.1694	0.003064	55.288	PY71	MG	ZY08	EHL	0.1519	0.003561	42.657
PY43	MG	ZY21	EHL	0.1657	0.003015	54.96	PY71	MG	ZY10	EHL	0.1526	0.003515	43.42
PY44	MG	ZY02	EHL	0.1694	0.003087	54.897	PY71	MG	ZY16	EHL	0.1442	0.00346	41.689
PY44	MG	ZY04	EHL	0.1624	0.002953	55.002	PY71	MG	ZY17	EHL	0.148	0.003389	43.685
PY44	MG	ZY06	EHL	0.1701	0.003067	55.447	PY71	MG	ZY18	EHL	0.1496	0.003462	43.225
PY44	MG	ZY07	EHL	0.1693	0.003101	54.607	PY71	MG	ZY19	EHL	0.148	0.003513	42.127
PY44	MG	ZY08	EHL	0.1711	0.003031	56.449	PY71	MG	ZY20	EHL	0.1482	0.00333	44.519
PY44	MG	ZY10	EHL	0.1696	0.003086	54.959	PY71	MG	ZY21	EHL	0.1468	0.003393	43.25

Table S17. Results of qpDstat analysis across PG individuals and MG.

Pop1	Pop2	Pop3	Pop4	*D*-stat	SE	Z	Pop1	Pop2	Pop3	Pop4	*D*-stat	SE	Z
PY02	PY05	MG	EHL	0.0098	0.003049	3.201	PY44	PY45	MG	EHL	0.0009	0.002853	0.309
PY02	PY06	MG	EHL	0.0116	0.003277	3.533	PY44	PY46	MG	EHL	0.0057	0.002545	2.246
PY02	PY10	MG	EHL	0.0014	0.00276	0.507	PY44	PY47	MG	EHL	-0.0003	0.002268	-0.112
PY02	PY17	MG	EHL	-0.0154	0.002349	-6.553	PY44	PY48	MG	EHL	-0.0002	0.00243	-0.075
PY02	PY22	MG	EHL	-0.0004	0.002545	-0.175	PY44	PY49	MG	EHL	0.0044	0.002656	1.669
PY02	PY23	MG	EHL	0.001	0.002649	0.371	PY44	PY50	MG	EHL	-0.0005	0.002238	-0.219
PY02	PY24	MG	EHL	0.007	0.002554	2.741	PY44	PY51	MG	EHL	-0.0024	0.002591	-0.917
PY02	PY25	MG	EHL	0.0033	0.002551	1.282	PY44	PY52	MG	EHL	0.0043	0.002525	1.714
PY02	PY26	MG	EHL	0.0046	0.002615	1.744	PY44	PY53	MG	EHL	0.0039	0.002612	1.49
PY02	PY27	MG	EHL	0.0152	0.00273	5.562	PY44	PY54	MG	EHL	0.0035	0.002418	1.438
PY02	PY28	MG	EHL	0.006	0.002935	2.038	PY44	PY55	MG	EHL	0.003	0.002263	1.324
PY02	PY29	MG	EHL	0.0056	0.002422	2.297	PY44	PY56	MG	EHL	0.0012	0.002271	0.535
PY02	PY30	MG	EHL	0.0013	0.002297	0.552	PY44	PY57	MG	EHL	0.0022	0.002205	1.01
PY02	PY31	MG	EHL	0.0072	0.002793	2.59	PY44	PY58	MG	EHL	0.0028	0.002246	1.236
PY02	PY32	MG	EHL	0.0082	0.002995	2.729	PY44	PY59	MG	EHL	-0.0005	0.002276	-0.234
PY02	PY33	MG	EHL	0.0036	0.002422	1.507	PY44	PY60	MG	EHL	0.0014	0.002232	0.619
PY02	PY34	MG	EHL	0.0424	0.00263	16.12	PY44	PY61	MG	EHL	0.0007	0.002338	0.32
PY02	PY36	MG	EHL	0.0045	0.00295	1.538	PY44	PY62	MG	EHL	0.0043	0.002808	1.522
PY02	PY37	MG	EHL	0.008	0.002812	2.842	PY44	PY63	MG	EHL	-0.0006	0.002301	-0.275
PY02	PY39	MG	EHL	-0.0148	0.002376	-6.23	PY44	PY67	MG	EHL	-0.0001	0.002667	-0.032
PY02	PY40	MG	EHL	0.0067	0.002831	2.361	PY44	PY68	MG	EHL	-0.0032	0.002378	-1.342
PY02	PY42	MG	EHL	0.0041	0.002586	1.574	PY44	PY69	MG	EHL	0.0006	0.002649	0.227

PY02	PY43	MG	EHL	0.0053	0.002592	2.032	PY44	PY70	MG	EHL	0.0553	0.005821	9.507
PY02	PY44	MG	EHL	0.0041	0.002512	1.643	PY44	PY71	MG	EHL	-0.0077	0.002482	-3.1
PY02	PY45	MG	EHL	0.0047	0.00275	1.724	PY45	PY02	MG	EHL	-0.0047	0.00275	-1.724
PY02	PY46	MG	EHL	0.0093	0.002656	3.516	PY45	PY05	MG	EHL	0.0044	0.003204	1.374
PY02	PY47	MG	EHL	0.0038	0.002459	1.553	PY45	PY06	MG	EHL	0.0068	0.003352	2.034
PY02	PY48	MG	EHL	0.0038	0.002612	1.471	PY45	PY10	MG	EHL	-0.0031	0.002893	-1.078
PY02	PY49	MG	EHL	0.0082	0.002717	3.018	PY45	PY17	MG	EHL	-0.0195	0.002384	-8.175
PY02	PY50	MG	EHL	0.0037	0.00251	1.489	PY45	PY22	MG	EHL	-0.0054	0.002737	-1.974
PY02	PY51	MG	EHL	0.0019	0.002699	0.722	PY45	PY23	MG	EHL	-0.0038	0.002462	-1.552
PY02	PY52	MG	EHL	0.0083	0.002614	3.156	PY45	PY24	MG	EHL	0.0024	0.002767	0.859
PY02	PY53	MG	EHL	0.0079	0.002582	3.053	PY45	PY25	MG	EHL	-0.0015	0.002699	-0.547
PY02	PY54	MG	EHL	0.0074	0.002615	2.844	PY45	PY26	MG	EHL	-0.0003	0.002733	-0.094
PY02	PY55	MG	EHL	0.0068	0.002578	2.624	PY45	PY27	MG	EHL	0.0112	0.003038	3.703
PY02	PY56	MG	EHL	0.0052	0.002391	2.156	PY45	PY28	MG	EHL	0.0006	0.003243	0.171
PY02	PY57	MG	EHL	0.0066	0.002419	2.723	PY45	PY29	MG	EHL	0.001	0.002576	0.383
PY02	PY58	MG	EHL	0.0067	0.002437	2.747	PY45	PY30	MG	EHL	-0.0035	0.002612	-1.358
PY02	PY59	MG	EHL	0.0036	0.002581	1.412	PY45	PY31	MG	EHL	0.0028	0.002687	1.051
PY02	PY60	MG	EHL	0.0057	0.002493	2.296	PY45	PY32	MG	EHL	0.0038	0.00329	1.145
PY02	PY61	MG	EHL	0.0044	0.002562	1.737	PY45	PY33	MG	EHL	-0.0011	0.002756	-0.397
PY02	PY62	MG	EHL	0.0081	0.002913	2.783	PY45	PY34	MG	EHL	0.0364	0.002735	13.325
PY02	PY63	MG	EHL	0.0036	0.002547	1.405	PY45	PY36	MG	EHL	-0.0002	0.00311	-0.059
PY02	PY67	MG	EHL	0.004	0.002739	1.475	PY45	PY37	MG	EHL	0.0036	0.003232	1.112
PY02	PY68	MG	EHL	0.001	0.002412	0.428	PY45	PY39	MG	EHL	-0.0193	0.002478	-7.781
PY02	PY69	MG	EHL	0.0046	0.002795	1.637	PY45	PY40	MG	EHL	0.002	0.003181	0.635
PY02	PY70	MG	EHL	0.0591	0.005756	10.265	PY45	PY42	MG	EHL	-0.0005	0.002951	-0.165

PY02	PY71	MG	EHL	-0.0035	0.002521	-1.395	PY45	PY43	MG	EHL	0.0007	0.002781	0.244
PY05	PY02	MG	EHL	-0.0098	0.003049	-3.201	PY45	PY44	MG	EHL	-0.0009	0.002853	-0.309
PY05	PY06	MG	EHL	0.0021	0.004265	0.485	PY45	PY46	MG	EHL	0.0051	0.003001	1.713
PY05	PY10	MG	EHL	-0.0092	0.003033	-3.03	PY45	PY47	MG	EHL	-0.0012	0.002808	-0.436
PY05	PY17	MG	EHL	-0.0243	0.002715	-8.937	PY45	PY48	MG	EHL	-0.001	0.002844	-0.348
PY05	PY22	MG	EHL	-0.0098	0.003236	-3.036	PY45	PY49	MG	EHL	0.0037	0.003121	1.186
PY05	PY23	MG	EHL	-0.0082	0.003187	-2.56	PY45	PY50	MG	EHL	-0.0013	0.002661	-0.471
PY05	PY24	MG	EHL	-0.0026	0.002995	-0.855	PY45	PY51	MG	EHL	-0.0029	0.002961	-0.977
PY05	PY25	MG	EHL	-0.0061	0.003074	-1.992	PY45	PY52	MG	EHL	0.0039	0.002978	1.302
PY05	PY26	MG	EHL	-0.0049	0.002914	-1.695	PY45	PY53	MG	EHL	0.0031	0.002715	1.144
PY05	PY27	MG	EHL	0.0066	0.003147	2.085	PY45	PY54	MG	EHL	0.0029	0.002764	1.048
PY05	PY28	MG	EHL	-0.0038	0.003451	-1.096	PY45	PY55	MG	EHL	0.0023	0.002703	0.841
PY05	PY29	MG	EHL	-0.0038	0.002903	-1.292	PY45	PY56	MG	EHL	0.0004	0.002629	0.15
PY05	PY30	MG	EHL	-0.008	0.002862	-2.811	PY45	PY57	MG	EHL	0.0014	0.002672	0.512
PY05	PY31	MG	EHL	-0.002	0.003272	-0.611	PY45	PY58	MG	EHL	0.0023	0.002713	0.831
PY05	PY32	MG	EHL	-0.0012	0.00348	-0.336	PY45	PY59	MG	EHL	-0.0011	0.002738	-0.402
PY05	PY33	MG	EHL	-0.0053	0.00311	-1.702	PY45	PY60	MG	EHL	0.0009	0.002666	0.329
PY05	PY34	MG	EHL	0.0316	0.003089	10.225	PY45	PY61	MG	EHL	0.0003	0.002851	0.088
PY05	PY36	MG	EHL	-0.0048	0.003393	-1.416	PY45	PY62	MG	EHL	0.0038	0.003266	1.171
PY05	PY37	MG	EHL	-0.001	0.003369	-0.304	PY45	PY63	MG	EHL	-0.0013	0.002739	-0.489
PY05	PY39	MG	EHL	-0.0234	0.002741	-8.554	PY45	PY67	MG	EHL	-0.0009	0.00284	-0.309
PY05	PY40	MG	EHL	-0.0026	0.003345	-0.776	PY45	PY68	MG	EHL	-0.0039	0.002692	-1.436
PY05	PY42	MG	EHL	-0.0048	0.003164	-1.502	PY45	PY69	MG	EHL	-0.0003	0.003134	-0.088
PY05	PY43	MG	EHL	-0.0039	0.0031	-1.273	PY45	PY70	MG	EHL	0.0546	0.005772	9.465
PY05	PY44	MG	EHL	-0.0051	0.002826	-1.797	PY45	PY71	MG	EHL	-0.0083	0.002816	-2.931

PY05	PY45	MG	EHL	-0.0044	0.003204	-1.374	PY46	PY02	MG	EHL	-0.0093	0.002656	-3.516
PY05	PY46	MG	EHL	0.0005	0.003026	0.161	PY46	PY05	MG	EHL	-0.0005	0.003026	-0.161
PY05	PY47	MG	EHL	-0.0058	0.003201	-1.804	PY46	PY06	MG	EHL	0.0025	0.003308	0.767
PY05	PY48	MG	EHL	-0.0054	0.003057	-1.762	PY46	PY10	MG	EHL	-0.0077	0.00284	-2.702
PY05	PY49	MG	EHL	-0.0011	0.003212	-0.337	PY46	PY17	MG	EHL	-0.024	0.002231	-10.751
PY05	PY50	MG	EHL	-0.0055	0.002901	-1.891	PY46	PY22	MG	EHL	-0.0101	0.002852	-3.548
PY05	PY51	MG	EHL	-0.0072	0.003233	-2.221	PY46	PY23	MG	EHL	-0.0087	0.002786	-3.116
PY05	PY52	MG	EHL	-0.0011	0.003211	-0.329	PY46	PY24	MG	EHL	-0.0022	0.002592	-0.853
PY05	PY53	MG	EHL	-0.0011	0.003147	-0.362	PY46	PY25	MG	EHL	-0.0061	0.002654	-2.31
PY05	PY54	MG	EHL	-0.0018	0.003098	-0.565	PY46	PY26	MG	EHL	-0.0049	0.002644	-1.836
PY05	PY55	MG	EHL	-0.0021	0.003078	-0.685	PY46	PY27	MG	EHL	0.0069	0.002763	2.488
PY05	PY56	MG	EHL	-0.0042	0.003068	-1.383	PY46	PY28	MG	EHL	-0.0036	0.003042	-1.189
PY05	PY57	MG	EHL	-0.003	0.003026	-0.997	PY46	PY29	MG	EHL	-0.0039	0.002595	-1.485
PY05	PY58	MG	EHL	-0.0024	0.003026	-0.79	PY46	PY30	MG	EHL	-0.0083	0.002508	-3.328
PY05	PY59	MG	EHL	-0.0056	0.003061	-1.828	PY46	PY31	MG	EHL	-0.0019	0.002744	-0.707
PY05	PY60	MG	EHL	-0.0037	0.002986	-1.248	PY46	PY32	MG	EHL	-0.0012	0.003157	-0.392
PY05	PY61	MG	EHL	-0.0042	0.003143	-1.332	PY46	PY33	MG	EHL	-0.0058	0.002548	-2.281
PY05	PY62	MG	EHL	-0.0013	0.003357	-0.379	PY46	PY34	MG	EHL	0.0321	0.002614	12.298
PY05	PY63	MG	EHL	-0.0059	0.002959	-1.988	PY46	PY36	MG	EHL	-0.0048	0.002922	-1.629
PY05	PY67	MG	EHL	-0.0052	0.003244	-1.617	PY46	PY37	MG	EHL	-0.0011	0.003189	-0.349
PY05	PY68	MG	EHL	-0.0083	0.002964	-2.8	PY46	PY39	MG	EHL	-0.0237	0.002331	-10.173
PY05	PY69	MG	EHL	-0.0048	0.003261	-1.484	PY46	PY40	MG	EHL	-0.0029	0.003057	-0.947
PY05	PY70	MG	EHL	0.0585	0.007139	8.195	PY46	PY42	MG	EHL	-0.006	0.002696	-2.212
PY05	PY71	MG	EHL	-0.0129	0.003081	-4.182	PY46	PY43	MG	EHL	-0.0042	0.002808	-1.497
PY06	PY02	MG	EHL	-0.0116	0.003277	-3.533	PY46	PY44	MG	EHL	-0.0057	0.002545	-2.246

PY06	PY05	MG	EHL	-0.0021	0.004265	-0.485	PY46	PY45	MG	EHL	-0.0051	0.003001	-1.713
PY06	PY10	MG	EHL	-0.0116	0.003712	-3.113	PY46	PY47	MG	EHL	-0.0058	0.002713	-2.149
PY06	PY17	MG	EHL	-0.0261	0.003084	-8.457	PY46	PY48	MG	EHL	-0.0058	0.002731	-2.107
PY06	PY22	MG	EHL	-0.0122	0.003412	-3.578	PY46	PY49	MG	EHL	-0.0009	0.002941	-0.321
PY06	PY23	MG	EHL	-0.0107	0.003413	-3.127	PY46	PY50	MG	EHL	-0.0057	0.002456	-2.327
PY06	PY24	MG	EHL	-0.0047	0.003237	-1.466	PY46	PY51	MG	EHL	-0.0077	0.002799	-2.764
PY06	PY25	MG	EHL	-0.0082	0.003217	-2.545	PY46	PY52	MG	EHL	-0.0007	0.002889	-0.254
PY06	PY26	MG	EHL	-0.0069	0.003155	-2.182	PY46	PY53	MG	EHL	-0.0015	0.0028	-0.526
PY06	PY27	MG	EHL	0.0042	0.003524	1.179	PY46	PY54	MG	EHL	-0.0016	0.002659	-0.591
PY06	PY28	MG	EHL	-0.0061	0.003709	-1.658	PY46	PY55	MG	EHL	-0.0023	0.002702	-0.868
PY06	PY29	MG	EHL	-0.0059	0.00313	-1.897	PY46	PY56	MG	EHL	-0.0042	0.002633	-1.578
PY06	PY30	MG	EHL	-0.0103	0.003178	-3.235	PY46	PY57	MG	EHL	-0.0032	0.002637	-1.231
PY06	PY31	MG	EHL	-0.004	0.003423	-1.176	PY46	PY58	MG	EHL	-0.0026	0.002678	-0.958
PY06	PY32	MG	EHL	-0.0035	0.003746	-0.933	PY46	PY59	MG	EHL	-0.006	0.002558	-2.33
PY06	PY33	MG	EHL	-0.0079	0.003251	-2.427	PY46	PY60	MG	EHL	-0.0038	0.002652	-1.427
PY06	PY34	MG	EHL	0.0299	0.003265	9.146	PY46	PY61	MG	EHL	-0.0049	0.002789	-1.744
PY06	PY36	MG	EHL	-0.007	0.003541	-1.966	PY46	PY62	MG	EHL	-0.0008	0.003059	-0.265
PY06	PY37	MG	EHL	-0.0031	0.003597	-0.868	PY46	PY63	MG	EHL	-0.0059	0.002583	-2.288
PY06	PY39	MG	EHL	-0.0256	0.003027	-8.461	PY46	PY67	MG	EHL	-0.0055	0.00287	-1.918
PY06	PY40	MG	EHL	-0.005	0.00362	-1.388	PY46	PY68	MG	EHL	-0.0087	0.002574	-3.37
PY06	PY42	MG	EHL	-0.0072	0.003364	-2.133	PY46	PY69	MG	EHL	-0.005	0.002876	-1.753
PY06	PY43	MG	EHL	-0.0062	0.003412	-1.817	PY46	PY70	MG	EHL	0.0505	0.00596	8.469
PY06	PY44	MG	EHL	-0.0073	0.003141	-2.333	PY46	PY71	MG	EHL	-0.0126	0.002668	-4.708
PY06	PY45	MG	EHL	-0.0068	0.003352	-2.034	PY47	PY02	MG	EHL	-0.0038	0.002459	-1.553
PY06	PY46	MG	EHL	-0.0025	0.003308	-0.767	PY47	PY05	MG	EHL	0.0058	0.003201	1.804

PY06	PY47	MG	EHL	-0.0078	0.003183	-2.454	PY47	PY06	MG	EHL	0.0078	0.003183	2.454
PY06	PY48	MG	EHL	-0.0076	0.003252	-2.342	PY47	PY10	MG	EHL	-0.0024	0.002787	-0.856
PY06	PY49	MG	EHL	-0.0034	0.003529	-0.964	PY47	PY17	MG	EHL	-0.0188	0.002196	-8.552
PY06	PY50	MG	EHL	-0.0078	0.003334	-2.325	PY47	PY22	MG	EHL	-0.0046	0.002573	-1.783
PY06	PY51	MG	EHL	-0.0095	0.003434	-2.771	PY47	PY23	MG	EHL	-0.0028	0.002556	-1.114
PY06	PY52	MG	EHL	-0.003	0.003504	-0.862	PY47	PY24	MG	EHL	0.0031	0.002535	1.209
PY06	PY53	MG	EHL	-0.0038	0.003342	-1.129	PY47	PY25	MG	EHL	-0.0007	0.002502	-0.294
PY06	PY54	MG	EHL	-0.004	0.003335	-1.2	PY47	PY26	MG	EHL	0.001	0.002383	0.416
PY06	PY55	MG	EHL	-0.0042	0.003291	-1.282	PY47	PY27	MG	EHL	0.012	0.00272	4.399
PY06	PY56	MG	EHL	-0.0065	0.003167	-2.047	PY47	PY28	MG	EHL	0.0017	0.003094	0.552
PY06	PY57	MG	EHL	-0.0054	0.003268	-1.65	PY47	PY29	MG	EHL	0.0019	0.002435	0.78
PY06	PY58	MG	EHL	-0.0048	0.003238	-1.492	PY47	PY30	MG	EHL	-0.0025	0.002232	-1.111
PY06	PY59	MG	EHL	-0.0076	0.003254	-2.333	PY47	PY31	MG	EHL	0.0035	0.00267	1.319
PY06	PY60	MG	EHL	-0.0059	0.003354	-1.757	PY47	PY32	MG	EHL	0.0045	0.003074	1.45
PY06	PY61	MG	EHL	-0.0066	0.003314	-2.006	PY47	PY33	MG	EHL	-0.0002	0.002373	-0.096
PY06	PY62	MG	EHL	-0.0032	0.003624	-0.876	PY47	PY34	MG	EHL	0.0373	0.002501	14.923
PY06	PY63	MG	EHL	-0.0078	0.003264	-2.375	PY47	PY36	MG	EHL	0.0007	0.002862	0.256
PY06	PY67	MG	EHL	-0.0079	0.003379	-2.324	PY47	PY37	MG	EHL	0.0044	0.003204	1.377
PY06	PY68	MG	EHL	-0.0107	0.003152	-3.399	PY47	PY39	MG	EHL	-0.0183	0.002395	-7.643
PY06	PY69	MG	EHL	-0.0069	0.003442	-2.01	PY47	PY40	MG	EHL	0.0027	0.00284	0.949
PY06	PY70	MG	EHL	0.0569	0.007067	8.047	PY47	PY42	MG	EHL	0.0003	0.002573	0.116
PY06	PY71	MG	EHL	-0.0148	0.003364	-4.388	PY47	PY43	MG	EHL	0.0014	0.002585	0.541
PY10	PY02	MG	EHL	-0.0014	0.00276	-0.507	PY47	PY44	MG	EHL	0.0003	0.002268	0.112
PY10	PY05	MG	EHL	0.0092	0.003033	3.03	PY47	PY45	MG	EHL	0.0012	0.002808	0.436
PY10	PY06	MG	EHL	0.0116	0.003712	3.113	PY47	PY46	MG	EHL	0.0058	0.002713	2.149

PY10	PY17	MG	EHL	-0.017	0.002469	-6.901	PY47	PY48	MG	EHL	0.0002	0.002549	0.062
PY10	PY22	MG	EHL	-0.002	0.002766	-0.715	PY47	PY49	MG	EHL	0.0044	0.002862	1.545
PY10	PY23	MG	EHL	-0.0005	0.002817	-0.182	PY47	PY50	MG	EHL	0.0003	0.002404	0.108
PY10	PY24	MG	EHL	0.0053	0.002705	1.967	PY47	PY51	MG	EHL	-0.0018	0.002768	-0.651
PY10	PY25	MG	EHL	0.0019	0.002786	0.678	PY47	PY52	MG	EHL	0.0047	0.002744	1.706
PY10	PY26	MG	EHL	0.0033	0.002633	1.247	PY47	PY53	MG	EHL	0.0049	0.002718	1.789
PY10	PY27	MG	EHL	0.0144	0.002871	5.011	PY47	PY54	MG	EHL	0.0044	0.002605	1.676
PY10	PY28	MG	EHL	0.0041	0.003168	1.307	PY47	PY55	MG	EHL	0.0038	0.002374	1.597
PY10	PY29	MG	EHL	0.0042	0.002615	1.623	PY47	PY56	MG	EHL	0.0019	0.00247	0.775
PY10	PY30	MG	EHL	-0.0001	0.00249	-0.034	PY47	PY57	MG	EHL	0.003	0.002564	1.166
PY10	PY31	MG	EHL	0.0059	0.002753	2.146	PY47	PY58	MG	EHL	0.0035	0.002438	1.431
PY10	PY32	MG	EHL	0.0068	0.003282	2.083	PY47	PY59	MG	EHL	0.0003	0.002514	0.118
PY10	PY33	MG	EHL	0.0023	0.002697	0.845	PY47	PY60	MG	EHL	0.0022	0.002369	0.925
PY10	PY34	MG	EHL	0.0396	0.002796	14.17	PY47	PY61	MG	EHL	0.0014	0.002425	0.559
PY10	PY36	MG	EHL	0.0032	0.00309	1.024	PY47	PY62	MG	EHL	0.005	0.002883	1.744
PY10	PY37	MG	EHL	0.0068	0.003043	2.249	PY47	PY63	MG	EHL	-0.0002	0.002526	-0.077
PY10	PY39	MG	EHL	-0.0163	0.002453	-6.63	PY47	PY67	MG	EHL	0	0.002775	0.013
PY10	PY40	MG	EHL	0.0053	0.003169	1.678	PY47	PY68	MG	EHL	-0.003	0.002349	-1.292
PY10	PY42	MG	EHL	0.0024	0.002836	0.862	PY47	PY69	MG	EHL	0.0008	0.002763	0.272
PY10	PY43	MG	EHL	0.0038	0.002765	1.386	PY47	PY70	MG	EHL	0.0557	0.005873	9.475
PY10	PY44	MG	EHL	0.0027	0.002561	1.059	PY47	PY71	MG	EHL	-0.0073	0.002611	-2.792
PY10	PY45	MG	EHL	0.0031	0.002893	1.078	PY48	PY02	MG	EHL	-0.0038	0.002612	-1.471
PY10	PY46	MG	EHL	0.0077	0.00284	2.702	PY48	PY05	MG	EHL	0.0054	0.003057	1.762
PY10	PY47	MG	EHL	0.0024	0.002787	0.856	PY48	PY06	MG	EHL	0.0076	0.003252	2.342
PY10	PY48	MG	EHL	0.0025	0.00277	0.916	PY48	PY10	MG	EHL	-0.0025	0.00277	-0.916

PY10	PY49	MG	EHL	0.0068	0.002933	2.332	PY48	PY17	MG	EHL	-0.0189	0.002311	-8.18
PY10	PY50	MG	EHL	0.0027	0.002525	1.054	PY48	PY22	MG	EHL	-0.0047	0.002747	-1.717
PY10	PY51	MG	EHL	0.0008	0.002883	0.263	PY48	PY23	MG	EHL	-0.0032	0.002587	-1.247
PY10	PY52	MG	EHL	0.0072	0.002955	2.427	PY48	PY24	MG	EHL	0.003	0.002684	1.113
PY10	PY53	MG	EHL	0.0064	0.002888	2.232	PY48	PY25	MG	EHL	-0.0007	0.002564	-0.279
PY10	PY54	MG	EHL	0.0062	0.002796	2.204	PY48	PY26	MG	EHL	0.0006	0.002633	0.238
PY10	PY55	MG	EHL	0.0056	0.002694	2.073	PY48	PY27	MG	EHL	0.0124	0.00281	4.409
PY10	PY56	MG	EHL	0.0036	0.002717	1.322	PY48	PY28	MG	EHL	0.0017	0.002948	0.565
PY10	PY57	MG	EHL	0.005	0.002558	1.956	PY48	PY29	MG	EHL	0.0018	0.002459	0.722
PY10	PY58	MG	EHL	0.0054	0.002729	1.973	PY48	PY30	MG	EHL	-0.0028	0.002372	-1.177
PY10	PY59	MG	EHL	0.0023	0.002686	0.857	PY48	PY31	MG	EHL	0.0035	0.002835	1.244
PY10	PY60	MG	EHL	0.0041	0.002723	1.49	PY48	PY32	MG	EHL	0.0046	0.003014	1.534
PY10	PY61	MG	EHL	0.0037	0.002781	1.335	PY48	PY33	MG	EHL	-0.0003	0.002563	-0.124
PY10	PY62	MG	EHL	0.0068	0.003028	2.255	PY48	PY34	MG	EHL	0.0373	0.00261	14.302
PY10	PY63	MG	EHL	0.0021	0.002648	0.808	PY48	PY36	MG	EHL	0.0006	0.002869	0.207
PY10	PY67	MG	EHL	0.0027	0.002858	0.929	PY48	PY37	MG	EHL	0.0048	0.003081	1.555
PY10	PY68	MG	EHL	-0.0004	0.00266	-0.147	PY48	PY39	MG	EHL	-0.0186	0.002383	-7.789
PY10	PY69	MG	EHL	0.0032	0.003013	1.078	PY48	PY40	MG	EHL	0.0032	0.00289	1.099
PY10	PY70	MG	EHL	0.0652	0.006545	9.963	PY48	PY42	MG	EHL	0.0004	0.002632	0.153
PY10	PY71	MG	EHL	-0.0048	0.002899	-1.66	PY48	PY43	MG	EHL	0.0014	0.002756	0.493
PY17	PY02	MG	EHL	0.0154	0.002349	6.553	PY48	PY44	MG	EHL	0.0002	0.00243	0.075
PY17	PY05	MG	EHL	0.0243	0.002715	8.937	PY48	PY45	MG	EHL	0.001	0.002844	0.348
PY17	PY06	MG	EHL	0.0261	0.003084	8.457	PY48	PY46	MG	EHL	0.0058	0.002731	2.107
PY17	PY10	MG	EHL	0.017	0.002469	6.901	PY48	PY47	MG	EHL	-0.0002	0.002549	-0.062
PY17	PY22	MG	EHL	0.0148	0.00229	6.473	PY48	PY49	MG	EHL	0.0046	0.002942	1.569

PY17	PY23	MG	EHL	0.0165	0.002176	7.564	PY48	PY50	MG	EHL	-0.0002	0.002402	-0.093
PY17	PY24	MG	EHL	0.0216	0.002356	9.183	PY48	PY51	MG	EHL	-0.0023	0.002796	-0.816
PY17	PY25	MG	EHL	0.0182	0.002231	8.164	PY48	PY52	MG	EHL	0.0047	0.002815	1.671
PY17	PY26	MG	EHL	0.0196	0.00207	9.481	PY48	PY53	MG	EHL	0.0041	0.002776	1.461
PY17	PY27	MG	EHL	0.0296	0.002471	11.966	PY48	PY54	MG	EHL	0.0035	0.002647	1.327
PY17	PY28	MG	EHL	0.02	0.002734	7.333	PY48	PY55	MG	EHL	0.0033	0.002483	1.323
PY17	PY29	MG	EHL	0.0209	0.002122	9.828	PY48	PY56	MG	EHL	0.0013	0.002561	0.517
PY17	PY30	MG	EHL	0.0167	0.002093	7.955	PY48	PY57	MG	EHL	0.0025	0.002539	1.004
PY17	PY31	MG	EHL	0.0216	0.002342	9.221	PY48	PY58	MG	EHL	0.003	0.002545	1.191
PY17	PY32	MG	EHL	0.0231	0.002718	8.503	PY48	PY59	MG	EHL	-0.0002	0.002512	-0.095
PY17	PY33	MG	EHL	0.0189	0.002211	8.535	PY48	PY60	MG	EHL	0.0018	0.002555	0.706
PY17	PY34	MG	EHL	0.0547	0.002333	23.449	PY48	PY61	MG	EHL	0.001	0.002591	0.401
PY17	PY36	MG	EHL	0.0198	0.002586	7.671	PY48	PY62	MG	EHL	0.0046	0.003051	1.507
PY17	PY37	MG	EHL	0.0233	0.002655	8.761	PY48	PY63	MG	EHL	-0.0003	0.002614	-0.122
PY17	PY39	MG	EHL	0.0006	0.002015	0.29	PY48	PY67	MG	EHL	0.0001	0.002811	0.029
PY17	PY40	MG	EHL	0.0207	0.002593	7.98	PY48	PY68	MG	EHL	-0.003	0.002556	-1.187
PY17	PY42	MG	EHL	0.0195	0.002321	8.402	PY48	PY69	MG	EHL	0.0007	0.002884	0.253
PY17	PY43	MG	EHL	0.0202	0.002236	9.031	PY48	PY70	MG	EHL	0.0554	0.005912	9.363
PY17	PY44	MG	EHL	0.0194	0.00219	8.854	PY48	PY71	MG	EHL	-0.0074	0.002821	-2.608
PY17	PY45	MG	EHL	0.0195	0.002384	8.175	PY49	PY02	MG	EHL	-0.0082	0.002717	-3.018
PY17	PY46	MG	EHL	0.024	0.002231	10.751	PY49	PY05	MG	EHL	0.0011	0.003212	0.337
PY17	PY47	MG	EHL	0.0188	0.002196	8.552	PY49	PY06	MG	EHL	0.0034	0.003529	0.964
PY17	PY48	MG	EHL	0.0189	0.002311	8.18	PY49	PY10	MG	EHL	-0.0068	0.002933	-2.332
PY17	PY49	MG	EHL	0.023	0.002585	8.911	PY49	PY17	MG	EHL	-0.023	0.002585	-8.911
PY17	PY50	MG	EHL	0.0187	0.002107	8.877	PY49	PY22	MG	EHL	-0.0091	0.002945	-3.08

PY17	PY51	MG	EHL	0.0173	0.002324	7.441	PY49	PY23	MG	EHL	-0.0076	0.002964	-2.552
PY17	PY52	MG	EHL	0.0232	0.002364	9.797	PY49	PY24	MG	EHL	-0.0014	0.002725	-0.519
PY17	PY53	MG	EHL	0.0231	0.002311	10	PY49	PY25	MG	EHL	-0.0054	0.002966	-1.816
PY17	PY54	MG	EHL	0.0225	0.00236	9.536	PY49	PY26	MG	EHL	-0.0042	0.002814	-1.486
PY17	PY55	MG	EHL	0.0218	0.002236	9.745	PY49	PY27	MG	EHL	0.008	0.002872	2.79
PY17	PY56	MG	EHL	0.0201	0.002122	9.464	PY49	PY28	MG	EHL	-0.0032	0.002628	-1.233
PY17	PY57	MG	EHL	0.0214	0.002096	10.218	PY49	PY29	MG	EHL	-0.0029	0.002704	-1.062
PY17	PY58	MG	EHL	0.0219	0.002212	9.882	PY49	PY30	MG	EHL	-0.0077	0.002701	-2.836
PY17	PY59	MG	EHL	0.0188	0.002112	8.909	PY49	PY31	MG	EHL	-0.0011	0.002979	-0.374
PY17	PY60	MG	EHL	0.0203	0.002125	9.548	PY49	PY32	MG	EHL	-0.0001	0.002605	-0.046
PY17	PY61	MG	EHL	0.0204	0.002338	8.738	PY49	PY33	MG	EHL	-0.005	0.002616	-1.909
PY17	PY62	MG	EHL	0.0226	0.002695	8.404	PY49	PY34	MG	EHL	0.0334	0.002747	12.145
PY17	PY63	MG	EHL	0.0185	0.002268	8.167	PY49	PY36	MG	EHL	-0.0039	0.003112	-1.258
PY17	PY67	MG	EHL	0.0193	0.002482	7.783	PY49	PY37	MG	EHL	-0.0001	0.003293	-0.039
PY17	PY68	MG	EHL	0.0162	0.002214	7.312	PY49	PY39	MG	EHL	-0.0226	0.002485	-9.091
PY17	PY69	MG	EHL	0.0198	0.002525	7.834	PY49	PY40	MG	EHL	-0.0014	0.003077	-0.467
PY17	PY70	MG	EHL	0.0713	0.005603	12.717	PY49	PY42	MG	EHL	-0.0046	0.002872	-1.585
PY17	PY71	MG	EHL	0.0124	0.002318	5.329	PY49	PY43	MG	EHL	-0.0032	0.002988	-1.075
PY22	PY02	MG	EHL	0.0004	0.002545	0.175	PY49	PY44	MG	EHL	-0.0044	0.002656	-1.669
PY22	PY05	MG	EHL	0.0098	0.003236	3.036	PY49	PY45	MG	EHL	-0.0037	0.003121	-1.186
PY22	PY06	MG	EHL	0.0122	0.003412	3.578	PY49	PY46	MG	EHL	0.0009	0.002941	0.321
PY22	PY10	MG	EHL	0.002	0.002766	0.715	PY49	PY47	MG	EHL	-0.0044	0.002862	-1.545
PY22	PY17	MG	EHL	-0.0148	0.00229	-6.473	PY49	PY48	MG	EHL	-0.0046	0.002942	-1.569
PY22	PY23	MG	EHL	0.002	0.002792	0.728	PY49	PY50	MG	EHL	-0.005	0.00271	-1.847
PY22	PY24	MG	EHL	0.0074	0.002778	2.672	PY49	PY51	MG	EHL	-0.0071	0.002829	-2.499

PY22	PY25	MG	EHL	0.004	0.002619	1.53	PY49	PY52	MG	EHL	0	0.002891	0.012
PY22	PY26	MG	EHL	0.0054	0.002609	2.059	PY49	PY53	MG	EHL	-0.0003	0.00292	-0.116
PY22	PY27	MG	EHL	0.0166	0.002874	5.788	PY49	PY54	MG	EHL	-0.0008	0.002975	-0.282
PY22	PY28	MG	EHL	0.0064	0.003003	2.139	PY49	PY55	MG	EHL	-0.0013	0.002755	-0.465
PY22	PY29	MG	EHL	0.0065	0.002544	2.537	PY49	PY56	MG	EHL	-0.0032	0.0027	-1.183
PY22	PY30	MG	EHL	0.0019	0.002489	0.782	PY49	PY57	MG	EHL	-0.0018	0.002759	-0.67
PY22	PY31	MG	EHL	0.0082	0.002674	3.063	PY49	PY58	MG	EHL	-0.0015	0.002813	-0.538
PY22	PY32	MG	EHL	0.009	0.003197	2.823	PY49	PY59	MG	EHL	-0.005	0.002801	-1.796
PY22	PY33	MG	EHL	0.0044	0.002579	1.715	PY49	PY60	MG	EHL	-0.0027	0.002789	-0.97
PY22	PY34	MG	EHL	0.0421	0.002579	16.307	PY49	PY61	MG	EHL	-0.0035	0.002841	-1.241
PY22	PY36	MG	EHL	0.0053	0.002866	1.844	PY49	PY62	MG	EHL	0	0.003213	0.009
PY22	PY37	MG	EHL	0.009	0.003097	2.907	PY49	PY63	MG	EHL	-0.0053	0.002582	-2.037
PY22	PY39	MG	EHL	-0.0144	0.002416	-5.941	PY49	PY67	MG	EHL	-0.0044	0.003071	-1.449
PY22	PY40	MG	EHL	0.0078	0.002965	2.64	PY49	PY68	MG	EHL	-0.0075	0.002846	-2.631
PY22	PY42	MG	EHL	0.0052	0.002727	1.923	PY49	PY69	MG	EHL	-0.0036	0.003111	-1.165
PY22	PY43	MG	EHL	0.0061	0.002704	2.24	PY49	PY70	MG	EHL	0.0514	0.006059	8.487
PY22	PY44	MG	EHL	0.0049	0.002575	1.888	PY49	PY71	MG	EHL	-0.0118	0.002818	-4.197
PY22	PY45	MG	EHL	0.0054	0.002737	1.974	PY50	PY02	MG	EHL	-0.0037	0.00251	-1.489
PY22	PY46	MG	EHL	0.0101	0.002852	3.548	PY50	PY05	MG	EHL	0.0055	0.002901	1.891
PY22	PY47	MG	EHL	0.0046	0.002573	1.783	PY50	PY06	MG	EHL	0.0078	0.003334	2.325
PY22	PY48	MG	EHL	0.0047	0.002747	1.717	PY50	PY10	MG	EHL	-0.0027	0.002525	-1.054
PY22	PY49	MG	EHL	0.0091	0.002945	3.08	PY50	PY17	MG	EHL	-0.0187	0.002107	-8.877
PY22	PY50	MG	EHL	0.0044	0.002475	1.79	PY50	PY22	MG	EHL	-0.0044	0.002475	-1.79
PY22	PY51	MG	EHL	0.0021	0.00279	0.764	PY50	PY23	MG	EHL	-0.0031	0.002254	-1.393
PY22	PY52	MG	EHL	0.0092	0.002726	3.364	PY50	PY24	MG	EHL	0.0039	0.002455	1.575

PY22	PY53	MG	EHL	0.0089	0.002782	3.186	PY50	PY25	MG	EHL	-0.0003	0.002592	-0.101
PY22	PY54	MG	EHL	0.0082	0.002698	3.022	PY50	PY26	MG	EHL	0.0011	0.002306	0.463
PY22	PY55	MG	EHL	0.0081	0.002513	3.204	PY50	PY27	MG	EHL	0.0125	0.002585	4.85
PY22	PY56	MG	EHL	0.0059	0.002482	2.358	PY50	PY28	MG	EHL	0.0018	0.002892	0.627
PY22	PY57	MG	EHL	0.0072	0.002543	2.834	PY50	PY29	MG	EHL	0.0019	0.002291	0.848
PY22	PY58	MG	EHL	0.0076	0.00254	2.972	PY50	PY30	MG	EHL	-0.0027	0.002143	-1.264
PY22	PY59	MG	EHL	0.0043	0.002608	1.663	PY50	PY31	MG	EHL	0.0037	0.002467	1.495
PY22	PY60	MG	EHL	0.0065	0.002407	2.687	PY50	PY32	MG	EHL	0.0047	0.002956	1.58
PY22	PY61	MG	EHL	0.0057	0.002671	2.146	PY50	PY33	MG	EHL	0.0001	0.002256	0.042
PY22	PY62	MG	EHL	0.009	0.002981	3.016	PY50	PY34	MG	EHL	0.0376	0.002374	15.859
PY22	PY63	MG	EHL	0.0041	0.002626	1.565	PY50	PY36	MG	EHL	0.0008	0.002657	0.294
PY22	PY67	MG	EHL	0.005	0.002839	1.762	PY50	PY37	MG	EHL	0.0048	0.002696	1.794
PY22	PY68	MG	EHL	0.0019	0.002559	0.733	PY50	PY39	MG	EHL	-0.0183	0.002131	-8.608
PY22	PY69	MG	EHL	0.0057	0.002972	1.925	PY50	PY40	MG	EHL	0.0032	0.002743	1.149
PY22	PY70	MG	EHL	0.0599	0.005834	10.276	PY50	PY42	MG	EHL	0.0003	0.002379	0.143
PY22	PY71	MG	EHL	-0.003	0.002659	-1.13	PY50	PY43	MG	EHL	0.0017	0.002351	0.704
PY23	PY02	MG	EHL	-0.001	0.002649	-0.371	PY50	PY44	MG	EHL	0.0005	0.002238	0.219
PY23	PY05	MG	EHL	0.0082	0.003187	2.56	PY50	PY45	MG	EHL	0.0013	0.002661	0.471
PY23	PY06	MG	EHL	0.0107	0.003413	3.127	PY50	PY46	MG	EHL	0.0057	0.002456	2.327
PY23	PY10	MG	EHL	0.0005	0.002817	0.182	PY50	PY47	MG	EHL	-0.0003	0.002404	-0.108
PY23	PY17	MG	EHL	-0.0165	0.002176	-7.564	PY50	PY48	MG	EHL	0.0002	0.002402	0.093
PY23	PY22	MG	EHL	-0.002	0.002792	-0.728	PY50	PY49	MG	EHL	0.005	0.00271	1.847
PY23	PY24	MG	EHL	0.0058	0.002792	2.068	PY50	PY51	MG	EHL	-0.0022	0.002379	-0.931
PY23	PY25	MG	EHL	0.0026	0.002605	0.997	PY50	PY52	MG	EHL	0.0049	0.002457	2.01
PY23	PY26	MG	EHL	0.0037	0.002643	1.392	PY50	PY53	MG	EHL	0.0047	0.002654	1.754

PY23	PY27	MG	EHL	0.0155	0.002942	5.282	PY50	PY54	MG	EHL	0.0039	0.002373	1.656
PY23	PY28	MG	EHL	0.0051	0.003093	1.652	PY50	PY55	MG	EHL	0.0033	0.002203	1.491
PY23	PY29	MG	EHL	0.0049	0.002494	1.962	PY50	PY56	MG	EHL	0.0014	0.002259	0.605
PY23	PY30	MG	EHL	0.0005	0.002495	0.185	PY50	PY57	MG	EHL	0.0027	0.00222	1.196
PY23	PY31	MG	EHL	0.0065	0.002615	2.498	PY50	PY58	MG	EHL	0.0029	0.002329	1.241
PY23	PY32	MG	EHL	0.0077	0.003138	2.462	PY50	PY59	MG	EHL	0.0002	0.002322	0.084
PY23	PY33	MG	EHL	0.0027	0.002516	1.068	PY50	PY60	MG	EHL	0.0017	0.002164	0.763
PY23	PY34	MG	EHL	0.0408	0.002511	16.249	PY50	PY61	MG	EHL	0.0013	0.002326	0.557
PY23	PY36	MG	EHL	0.0037	0.002825	1.325	PY50	PY62	MG	EHL	0.0049	0.002704	1.813
PY23	PY37	MG	EHL	0.0075	0.003102	2.404	PY50	PY63	MG	EHL	-0.0003	0.002328	-0.115
PY23	PY39	MG	EHL	-0.0157	0.002355	-6.654	PY50	PY67	MG	EHL	0.0004	0.002442	0.176
PY23	PY40	MG	EHL	0.0061	0.002975	2.066	PY50	PY68	MG	EHL	-0.0028	0.002241	-1.245
PY23	PY42	MG	EHL	0.0037	0.002665	1.376	PY50	PY69	MG	EHL	0.001	0.00271	0.36
PY23	PY43	MG	EHL	0.0044	0.002569	1.715	PY50	PY70	MG	EHL	0.0562	0.005882	9.554
PY23	PY44	MG	EHL	0.0033	0.002615	1.265	PY50	PY71	MG	EHL	-0.0074	0.002513	-2.955
PY23	PY45	MG	EHL	0.0038	0.002462	1.552	PY51	PY02	MG	EHL	-0.0019	0.002699	-0.722
PY23	PY46	MG	EHL	0.0087	0.002786	3.116	PY51	PY05	MG	EHL	0.0072	0.003233	2.221
PY23	PY47	MG	EHL	0.0028	0.002556	1.114	PY51	PY06	MG	EHL	0.0095	0.003434	2.771
PY23	PY48	MG	EHL	0.0032	0.002587	1.247	PY51	PY10	MG	EHL	-0.0008	0.002883	-0.263
PY23	PY49	MG	EHL	0.0076	0.002964	2.552	PY51	PY17	MG	EHL	-0.0173	0.002324	-7.441
PY23	PY50	MG	EHL	0.0031	0.002254	1.393	PY51	PY22	MG	EHL	-0.0021	0.00279	-0.764
PY23	PY51	MG	EHL	0.0006	0.002765	0.215	PY51	PY23	MG	EHL	-0.0006	0.002765	-0.215
PY23	PY52	MG	EHL	0.0072	0.002843	2.529	PY51	PY24	MG	EHL	0.0053	0.002805	1.894
PY23	PY53	MG	EHL	0.0073	0.002736	2.674	PY51	PY25	MG	EHL	0.0018	0.002644	0.662
PY23	PY54	MG	EHL	0.0066	0.002652	2.49	PY51	PY26	MG	EHL	0.003	0.002575	1.163

PY23	PY55	MG	EHL	0.0064	0.002474	2.574	PY51	PY27	MG	EHL	0.0154	0.002952	5.212
PY23	PY56	MG	EHL	0.0043	0.002497	1.736	PY51	PY28	MG	EHL	0.004	0.003218	1.25
PY23	PY57	MG	EHL	0.0056	0.002507	2.233	PY51	PY29	MG	EHL	0.0042	0.00266	1.596
PY23	PY58	MG	EHL	0.006	0.002633	2.284	PY51	PY30	MG	EHL	-0.0007	0.002568	-0.259
PY23	PY59	MG	EHL	0.003	0.002551	1.165	PY51	PY31	MG	EHL	0.0054	0.00283	1.915
PY23	PY60	MG	EHL	0.0048	0.002411	1.986	PY51	PY32	MG	EHL	0.0068	0.003261	2.074
PY23	PY61	MG	EHL	0.0041	0.002589	1.593	PY51	PY33	MG	EHL	0.0019	0.002411	0.776
PY23	PY62	MG	EHL	0.0074	0.003021	2.445	PY51	PY34	MG	EHL	0.0393	0.00259	15.173
PY23	PY63	MG	EHL	0.0023	0.002495	0.934	PY51	PY36	MG	EHL	0.0028	0.002934	0.943
PY23	PY67	MG	EHL	0.0034	0.002541	1.329	PY51	PY37	MG	EHL	0.0065	0.003087	2.109
PY23	PY68	MG	EHL	0.0002	0.002509	0.096	PY51	PY39	MG	EHL	-0.0164	0.002346	-7.001
PY23	PY69	MG	EHL	0.004	0.002891	1.397	PY51	PY40	MG	EHL	0.0041	0.002909	1.417
PY23	PY70	MG	EHL	0.0585	0.005869	9.974	PY51	PY42	MG	EHL	0.0024	0.002681	0.88
PY23	PY71	MG	EHL	-0.0043	0.002697	-1.597	PY51	PY43	MG	EHL	0.0036	0.002633	1.382
PY24	PY02	MG	EHL	-0.007	0.002554	-2.741	PY51	PY44	MG	EHL	0.0024	0.002591	0.917
PY24	PY05	MG	EHL	0.0026	0.002995	0.855	PY51	PY45	MG	EHL	0.0029	0.002961	0.977
PY24	PY06	MG	EHL	0.0047	0.003237	1.466	PY51	PY46	MG	EHL	0.0077	0.002799	2.764
PY24	PY10	MG	EHL	-0.0053	0.002705	-1.967	PY51	PY47	MG	EHL	0.0018	0.002768	0.651
PY24	PY17	MG	EHL	-0.0216	0.002356	-9.183	PY51	PY48	MG	EHL	0.0023	0.002796	0.816
PY24	PY22	MG	EHL	-0.0074	0.002778	-2.672	PY51	PY49	MG	EHL	0.0071	0.002829	2.499
PY24	PY23	MG	EHL	-0.0058	0.002792	-2.068	PY51	PY50	MG	EHL	0.0022	0.002379	0.931
PY24	PY25	MG	EHL	-0.0042	0.002693	-1.551	PY51	PY52	MG	EHL	0.0065	0.002889	2.252
PY24	PY26	MG	EHL	-0.0031	0.002389	-1.298	PY51	PY53	MG	EHL	0.0056	0.002817	1.992
PY24	PY27	MG	EHL	0.0101	0.002694	3.732	PY51	PY54	MG	EHL	0.0054	0.002706	1.996
PY24	PY28	MG	EHL	-0.0023	0.002864	-0.81	PY51	PY55	MG	EHL	0.0052	0.002501	2.073

PY24	PY29	MG	EHL	-0.0009	0.002518	-0.371	PY51	PY56	MG	EHL	0.003	0.002442	1.248
PY24	PY30	MG	EHL	-0.0065	0.002401	-2.687	PY51	PY57	MG	EHL	0.0048	0.0025	1.933
PY24	PY31	MG	EHL	0.0002	0.002629	0.066	PY51	PY58	MG	EHL	0.0048	0.00254	1.881
PY24	PY32	MG	EHL	0.0012	0.002959	0.391	PY51	PY59	MG	EHL	0.0012	0.002593	0.461
PY24	PY33	MG	EHL	-0.0039	0.002594	-1.495	PY51	PY60	MG	EHL	0.0036	0.002457	1.467
PY24	PY34	MG	EHL	0.0343	0.002561	13.387	PY51	PY61	MG	EHL	0.0033	0.002675	1.243
PY24	PY36	MG	EHL	-0.0023	0.002949	-0.764	PY51	PY62	MG	EHL	0.0064	0.00305	2.089
PY24	PY37	MG	EHL	0.0014	0.003052	0.467	PY51	PY63	MG	EHL	0.0019	0.002454	0.79
PY24	PY39	MG	EHL	-0.0214	0.002293	-9.33	PY51	PY67	MG	EHL	0.0024	0.002886	0.841
PY24	PY40	MG	EHL	-0.0007	0.002924	-0.241	PY51	PY68	MG	EHL	-0.0006	0.002568	-0.247
PY24	PY42	MG	EHL	-0.003	0.00268	-1.105	PY51	PY69	MG	EHL	0.0028	0.003026	0.923
PY24	PY43	MG	EHL	-0.002	0.002744	-0.725	PY51	PY70	MG	EHL	0.0578	0.005786	9.991
PY24	PY44	MG	EHL	-0.003	0.0025	-1.185	PY51	PY71	MG	EHL	-0.0052	0.002711	-1.922
PY24	PY45	MG	EHL	-0.0024	0.002767	-0.859	PY52	PY02	MG	EHL	-0.0083	0.002614	-3.156
PY24	PY46	MG	EHL	0.0022	0.002592	0.853	PY52	PY05	MG	EHL	0.0011	0.003211	0.329
PY24	PY47	MG	EHL	-0.0031	0.002535	-1.209	PY52	PY06	MG	EHL	0.003	0.003504	0.862
PY24	PY48	MG	EHL	-0.003	0.002684	-1.113	PY52	PY10	MG	EHL	-0.0072	0.002955	-2.427
PY24	PY49	MG	EHL	0.0014	0.002725	0.519	PY52	PY17	MG	EHL	-0.0232	0.002364	-9.797
PY24	PY50	MG	EHL	-0.0039	0.002455	-1.575	PY52	PY22	MG	EHL	-0.0092	0.002726	-3.364
PY24	PY51	MG	EHL	-0.0053	0.002805	-1.894	PY52	PY23	MG	EHL	-0.0072	0.002843	-2.529
PY24	PY52	MG	EHL	0.0016	0.002744	0.596	PY52	PY24	MG	EHL	-0.0016	0.002744	-0.596
PY24	PY53	MG	EHL	0.0019	0.002542	0.764	PY52	PY25	MG	EHL	-0.005	0.002657	-1.894
PY24	PY54	MG	EHL	0.0007	0.002564	0.255	PY52	PY26	MG	EHL	-0.0037	0.002698	-1.365
PY24	PY55	MG	EHL	0.0008	0.002546	0.306	PY52	PY27	MG	EHL	0.0071	0.002798	2.541
PY24	PY56	MG	EHL	-0.0012	0.002471	-0.489	PY52	PY28	MG	EHL	-0.0024	0.00309	-0.784

PY24	PY57	MG	EHL	-0.0004	0.002422	-0.145	PY52	PY29	MG	EHL	-0.0027	0.002606	-1.046
PY24	PY58	MG	EHL	0	0.002415	0.002	PY52	PY30	MG	EHL	-0.007	0.00246	-2.853
PY24	PY59	MG	EHL	-0.0034	0.002522	-1.342	PY52	PY31	MG	EHL	-0.0012	0.002894	-0.403
PY24	PY60	MG	EHL	-0.0006	0.002454	-0.247	PY52	PY32	MG	EHL	-0.0003	0.003158	-0.106
PY24	PY61	MG	EHL	-0.0019	0.002621	-0.729	PY52	PY33	MG	EHL	-0.0049	0.002599	-1.89
PY24	PY62	MG	EHL	0.001	0.00294	0.346	PY52	PY34	MG	EHL	0.0333	0.002659	12.511
PY24	PY63	MG	EHL	-0.0036	0.002617	-1.382	PY52	PY36	MG	EHL	-0.004	0.003049	-1.317
PY24	PY67	MG	EHL	-0.0028	0.002935	-0.953	PY52	PY37	MG	EHL	-0.001	0.003167	-0.307
PY24	PY68	MG	EHL	-0.0058	0.002597	-2.237	PY52	PY39	MG	EHL	-0.0226	0.002429	-9.314
PY24	PY69	MG	EHL	-0.0026	0.002929	-0.884	PY52	PY40	MG	EHL	-0.0019	0.002931	-0.645
PY24	PY70	MG	EHL	0.0529	0.00584	9.064	PY52	PY42	MG	EHL	-0.0046	0.002836	-1.631
PY24	PY71	MG	EHL	-0.0111	0.002669	-4.162	PY52	PY43	MG	EHL	-0.0031	0.002839	-1.102
PY25	PY02	MG	EHL	-0.0033	0.002551	-1.282	PY52	PY44	MG	EHL	-0.0043	0.002525	-1.714
PY25	PY05	MG	EHL	0.0061	0.003074	1.992	PY52	PY45	MG	EHL	-0.0039	0.002978	-1.302
PY25	PY06	MG	EHL	0.0082	0.003217	2.545	PY52	PY46	MG	EHL	0.0007	0.002889	0.254
PY25	PY10	MG	EHL	-0.0019	0.002786	-0.678	PY52	PY47	MG	EHL	-0.0047	0.002744	-1.706
PY25	PY17	MG	EHL	-0.0182	0.002231	-8.164	PY52	PY48	MG	EHL	-0.0047	0.002815	-1.671
PY25	PY22	MG	EHL	-0.004	0.002619	-1.53	PY52	PY49	MG	EHL	0	0.002891	-0.012
PY25	PY23	MG	EHL	-0.0026	0.002605	-0.997	PY52	PY50	MG	EHL	-0.0049	0.002457	-2.01
PY25	PY24	MG	EHL	0.0042	0.002693	1.551	PY52	PY51	MG	EHL	-0.0065	0.002889	-2.252
PY25	PY26	MG	EHL	0.0015	0.00258	0.566	PY52	PY53	MG	EHL	-0.0002	0.00295	-0.083
PY25	PY27	MG	EHL	0.0133	0.002867	4.651	PY52	PY54	MG	EHL	-0.0008	0.002768	-0.274
PY25	PY28	MG	EHL	0.0027	0.003119	0.853	PY52	PY55	MG	EHL	-0.0016	0.002642	-0.598
PY25	PY29	MG	EHL	0.0026	0.002368	1.079	PY52	PY56	MG	EHL	-0.0037	0.002709	-1.352
PY25	PY30	MG	EHL	-0.0022	0.002446	-0.88	PY52	PY57	MG	EHL	-0.0021	0.002735	-0.772

PY25	PY31	MG	EHL	0.0044	0.002849	1.555	PY52	PY58	MG	EHL	-0.0017	0.00272	-0.638
PY25	PY32	MG	EHL	0.0053	0.002803	1.879	PY52	PY59	MG	EHL	-0.0049	0.002683	-1.822
PY25	PY33	MG	EHL	0.0004	0.002468	0.161	PY52	PY60	MG	EHL	-0.0029	0.002697	-1.07
PY25	PY34	MG	EHL	0.0378	0.002589	14.599	PY52	PY61	MG	EHL	-0.0033	0.002695	-1.226
PY25	PY36	MG	EHL	0.0013	0.002804	0.452	PY52	PY62	MG	EHL	-0.0001	0.002666	-0.044
PY25	PY37	MG	EHL	0.0051	0.002959	1.71	PY52	PY63	MG	EHL	-0.0052	0.002664	-1.963
PY25	PY39	MG	EHL	-0.0179	0.002273	-7.864	PY52	PY67	MG	EHL	-0.0046	0.002899	-1.579
PY25	PY40	MG	EHL	0.0036	0.002851	1.266	PY52	PY68	MG	EHL	-0.0072	0.002705	-2.674
PY25	PY42	MG	EHL	0.0007	0.002715	0.254	PY52	PY69	MG	EHL	-0.0039	0.002859	-1.37
PY25	PY43	MG	EHL	0.0021	0.002594	0.804	PY52	PY70	MG	EHL	0.051	0.005958	8.567
PY25	PY44	MG	EHL	0.0009	0.002529	0.35	PY52	PY71	MG	EHL	-0.0115	0.002803	-4.117
PY25	PY45	MG	EHL	0.0015	0.002699	0.547	PY53	PY02	MG	EHL	-0.0079	0.002582	-3.053
PY25	PY46	MG	EHL	0.0061	0.002654	2.31	PY53	PY05	MG	EHL	0.0011	0.003147	0.362
PY25	PY47	MG	EHL	0.0007	0.002502	0.294	PY53	PY06	MG	EHL	0.0038	0.003342	1.129
PY25	PY48	MG	EHL	0.0007	0.002564	0.279	PY53	PY10	MG	EHL	-0.0064	0.002888	-2.232
PY25	PY49	MG	EHL	0.0054	0.002966	1.816	PY53	PY17	MG	EHL	-0.0231	0.002311	-10
PY25	PY50	MG	EHL	0.0003	0.002592	0.101	PY53	PY22	MG	EHL	-0.0089	0.002782	-3.186
PY25	PY51	MG	EHL	-0.0018	0.002644	-0.662	PY53	PY23	MG	EHL	-0.0073	0.002736	-2.674
PY25	PY52	MG	EHL	0.005	0.002657	1.894	PY53	PY24	MG	EHL	-0.0019	0.002542	-0.764
PY25	PY53	MG	EHL	0.0048	0.002659	1.815	PY53	PY25	MG	EHL	-0.0048	0.002659	-1.815
PY25	PY54	MG	EHL	0.0043	0.00261	1.663	PY53	PY26	MG	EHL	-0.0036	0.002598	-1.404
PY25	PY55	MG	EHL	0.0039	0.0025	1.553	PY53	PY27	MG	EHL	0.0076	0.002885	2.626
PY25	PY56	MG	EHL	0.0019	0.00244	0.787	PY53	PY28	MG	EHL	-0.0026	0.003129	-0.816
PY25	PY57	MG	EHL	0.0032	0.002529	1.255	PY53	PY29	MG	EHL	-0.0022	0.002659	-0.831
PY25	PY58	MG	EHL	0.0036	0.002544	1.409	PY53	PY30	MG	EHL	-0.0073	0.002509	-2.927

PY25	PY59	MG	EHL	0.0002	0.002521	0.077	PY53	PY31	MG	EHL	-0.0006	0.002762	-0.201
PY25	PY60	MG	EHL	0.0025	0.002484	1.004	PY53	PY32	MG	EHL	0.0003	0.003116	0.089
PY25	PY61	MG	EHL	0.0015	0.002654	0.581	PY53	PY33	MG	EHL	-0.004	0.002588	-1.548
PY25	PY62	MG	EHL	0.0052	0.003007	1.722	PY53	PY34	MG	EHL	0.0336	0.00267	12.585
PY25	PY63	MG	EHL	0.0003	0.002627	0.132	PY53	PY36	MG	EHL	-0.0037	0.003026	-1.209
PY25	PY67	MG	EHL	0.0007	0.002723	0.271	PY53	PY37	MG	EHL	0.0007	0.003091	0.232
PY25	PY68	MG	EHL	-0.0023	0.002441	-0.945	PY53	PY39	MG	EHL	-0.0225	0.002293	-9.823
PY25	PY69	MG	EHL	0.0014	0.002849	0.477	PY53	PY40	MG	EHL	-0.0015	0.003151	-0.48
PY25	PY70	MG	EHL	0.0561	0.00587	9.565	PY53	PY42	MG	EHL	-0.0043	0.002841	-1.527
PY25	PY71	MG	EHL	-0.0068	0.002619	-2.579	PY53	PY43	MG	EHL	-0.0028	0.002762	-1.002
PY26	PY02	MG	EHL	-0.0046	0.002615	-1.744	PY53	PY44	MG	EHL	-0.0039	0.002612	-1.49
PY26	PY05	MG	EHL	0.0049	0.002914	1.695	PY53	PY45	MG	EHL	-0.0031	0.002715	-1.144
PY26	PY06	MG	EHL	0.0069	0.003155	2.182	PY53	PY46	MG	EHL	0.0015	0.0028	0.526
PY26	PY10	MG	EHL	-0.0033	0.002633	-1.247	PY53	PY47	MG	EHL	-0.0049	0.002718	-1.789
PY26	PY17	MG	EHL	-0.0196	0.00207	-9.481	PY53	PY48	MG	EHL	-0.0041	0.002776	-1.461
PY26	PY22	MG	EHL	-0.0054	0.002609	-2.059	PY53	PY49	MG	EHL	0.0003	0.00292	0.116
PY26	PY23	MG	EHL	-0.0037	0.002643	-1.392	PY53	PY50	MG	EHL	-0.0047	0.002654	-1.754
PY26	PY24	MG	EHL	0.0031	0.002389	1.298	PY53	PY51	MG	EHL	-0.0056	0.002817	-1.992
PY26	PY25	MG	EHL	-0.0015	0.00258	-0.566	PY53	PY52	MG	EHL	0.0002	0.00295	0.083
PY26	PY27	MG	EHL	0.0119	0.002658	4.486	PY53	PY54	MG	EHL	-0.0005	0.002785	-0.192
PY26	PY28	MG	EHL	0.001	0.003112	0.328	PY53	PY55	MG	EHL	-0.0013	0.002568	-0.519
PY26	PY29	MG	EHL	0.0008	0.002344	0.348	PY53	PY56	MG	EHL	-0.0037	0.00262	-1.429
PY26	PY30	MG	EHL	-0.0036	0.002256	-1.574	PY53	PY57	MG	EHL	-0.0017	0.002619	-0.662
PY26	PY31	MG	EHL	0.0032	0.002682	1.197	PY53	PY58	MG	EHL	-0.0014	0.002654	-0.53
PY26	PY32	MG	EHL	0.004	0.003173	1.259	PY53	PY59	MG	EHL	-0.0048	0.002734	-1.769

PY26	PY33	MG	EHL	-0.0012	0.002477	-0.479	PY53	PY60	MG	EHL	-0.003	0.002725	-1.098
PY26	PY34	MG	EHL	0.0368	0.002564	14.338	PY53	PY61	MG	EHL	-0.0038	0.002736	-1.394
PY26	PY36	MG	EHL	0.0001	0.00278	0.04	PY53	PY62	MG	EHL	0.0003	0.003142	0.097
PY26	PY37	MG	EHL	0.0038	0.002939	1.278	PY53	PY63	MG	EHL	-0.0042	0.002678	-1.553
PY26	PY39	MG	EHL	-0.019	0.002243	-8.455	PY53	PY67	MG	EHL	-0.0039	0.002999	-1.286
PY26	PY40	MG	EHL	0.0025	0.00279	0.891	PY53	PY68	MG	EHL	-0.0068	0.002522	-2.681
PY26	PY42	MG	EHL	-0.0008	0.002617	-0.314	PY53	PY69	MG	EHL	-0.0038	0.002979	-1.266
PY26	PY43	MG	EHL	0.0005	0.002513	0.197	PY53	PY70	MG	EHL	0.0517	0.005884	8.785
PY26	PY44	MG	EHL	-0.0006	0.002306	-0.241	PY53	PY71	MG	EHL	-0.0115	0.00269	-4.27
PY26	PY45	MG	EHL	0.0003	0.002733	0.094	PY54	PY02	MG	EHL	-0.0074	0.002615	-2.844
PY26	PY46	MG	EHL	0.0049	0.002644	1.836	PY54	PY05	MG	EHL	0.0018	0.003098	0.565
PY26	PY47	MG	EHL	-0.001	0.002383	-0.416	PY54	PY06	MG	EHL	0.004	0.003335	1.2
PY26	PY48	MG	EHL	-0.0006	0.002633	-0.238	PY54	PY10	MG	EHL	-0.0062	0.002796	-2.204
PY26	PY49	MG	EHL	0.0042	0.002814	1.486	PY54	PY17	MG	EHL	-0.0225	0.00236	-9.536
PY26	PY50	MG	EHL	-0.0011	0.002306	-0.463	PY54	PY22	MG	EHL	-0.0082	0.002698	-3.022
PY26	PY51	MG	EHL	-0.003	0.002575	-1.163	PY54	PY23	MG	EHL	-0.0066	0.002652	-2.49
PY26	PY52	MG	EHL	0.0037	0.002698	1.365	PY54	PY24	MG	EHL	-0.0007	0.002564	-0.255
PY26	PY53	MG	EHL	0.0036	0.002598	1.404	PY54	PY25	MG	EHL	-0.0043	0.00261	-1.663
PY26	PY54	MG	EHL	0.0028	0.002556	1.113	PY54	PY26	MG	EHL	-0.0028	0.002556	-1.113
PY26	PY55	MG	EHL	0.0024	0.002419	0.993	PY54	PY27	MG	EHL	0.0084	0.002834	2.949
PY26	PY56	MG	EHL	0.0007	0.002371	0.304	PY54	PY28	MG	EHL	-0.0022	0.003068	-0.731
PY26	PY57	MG	EHL	0.0022	0.002468	0.91	PY54	PY29	MG	EHL	-0.002	0.002543	-0.804
PY26	PY58	MG	EHL	0.0021	0.002476	0.854	PY54	PY30	MG	EHL	-0.0064	0.002478	-2.566
PY26	PY59	MG	EHL	-0.0009	0.002437	-0.384	PY54	PY31	MG	EHL	-0.0003	0.002596	-0.109
PY26	PY60	MG	EHL	0.0012	0.002397	0.517	PY54	PY32	MG	EHL	0.0006	0.003185	0.2

PY26	PY61	MG	EHL	0.0003	0.002642	0.123	PY54	PY33	MG	EHL	-0.0038	0.002434	-1.562
PY26	PY62	MG	EHL	0.0037	0.00302	1.226	PY54	PY34	MG	EHL	0.0338	0.002633	12.829
PY26	PY63	MG	EHL	-0.0012	0.002401	-0.503	PY54	PY36	MG	EHL	-0.0031	0.002843	-1.104
PY26	PY67	MG	EHL	-0.0007	0.002725	-0.245	PY54	PY37	MG	EHL	0.0004	0.003037	0.118
PY26	PY68	MG	EHL	-0.0036	0.002428	-1.477	PY54	PY39	MG	EHL	-0.0219	0.002389	-9.174
PY26	PY69	MG	EHL	0	0.002949	-0.013	PY54	PY40	MG	EHL	-0.0015	0.002988	-0.497
PY26	PY70	MG	EHL	0.0553	0.006006	9.209	PY54	PY42	MG	EHL	-0.0036	0.002519	-1.443
PY26	PY71	MG	EHL	-0.0081	0.002644	-3.076	PY54	PY43	MG	EHL	-0.0024	0.002656	-0.921
PY27	PY02	MG	EHL	-0.0152	0.00273	-5.562	PY54	PY44	MG	EHL	-0.0035	0.002418	-1.438
PY27	PY05	MG	EHL	-0.0066	0.003147	-2.085	PY54	PY45	MG	EHL	-0.0029	0.002764	-1.048
PY27	PY06	MG	EHL	-0.0042	0.003524	-1.179	PY54	PY46	MG	EHL	0.0016	0.002659	0.591
PY27	PY10	MG	EHL	-0.0144	0.002871	-5.011	PY54	PY47	MG	EHL	-0.0044	0.002605	-1.676
PY27	PY17	MG	EHL	-0.0296	0.002471	-11.966	PY54	PY48	MG	EHL	-0.0035	0.002647	-1.327
PY27	PY22	MG	EHL	-0.0166	0.002874	-5.788	PY54	PY49	MG	EHL	0.0008	0.002975	0.282
PY27	PY23	MG	EHL	-0.0155	0.002942	-5.282	PY54	PY50	MG	EHL	-0.0039	0.002373	-1.656
PY27	PY24	MG	EHL	-0.0101	0.002694	-3.732	PY54	PY51	MG	EHL	-0.0054	0.002706	-1.996
PY27	PY25	MG	EHL	-0.0133	0.002867	-4.651	PY54	PY52	MG	EHL	0.0008	0.002768	0.274
PY27	PY26	MG	EHL	-0.0119	0.002658	-4.486	PY54	PY53	MG	EHL	0.0005	0.002785	0.192
PY27	PY28	MG	EHL	-0.0114	0.003133	-3.629	PY54	PY55	MG	EHL	-0.0005	0.002631	-0.178
PY27	PY29	MG	EHL	-0.0106	0.002598	-4.094	PY54	PY56	MG	EHL	-0.0023	0.002542	-0.888
PY27	PY30	MG	EHL	-0.0158	0.002641	-5.995	PY54	PY57	MG	EHL	-0.0013	0.002521	-0.528
PY27	PY31	MG	EHL	-0.0091	0.002846	-3.184	PY54	PY58	MG	EHL	-0.0007	0.002571	-0.258
PY27	PY32	MG	EHL	-0.0075	0.003215	-2.343	PY54	PY59	MG	EHL	-0.0043	0.0027	-1.596
PY27	PY33	MG	EHL	-0.0125	0.002804	-4.461	PY54	PY60	MG	EHL	-0.0021	0.002508	-0.835
PY27	PY34	MG	EHL	0.0261	0.002748	9.495	PY54	PY61	MG	EHL	-0.0032	0.0024	-1.343

PY27	PY36	MG	EHL	-0.0114	0.003062	-3.718	PY54	PY62	MG	EHL	0.0009	0.003049	0.279
PY27	PY37	MG	EHL	-0.0079	0.003185	-2.494	PY54	PY63	MG	EHL	-0.0036	0.002642	-1.354
PY27	PY39	MG	EHL	-0.0292	0.002498	-11.687	PY54	PY67	MG	EHL	-0.0039	0.002775	-1.393
PY27	PY40	MG	EHL	-0.0099	0.003029	-3.285	PY54	PY68	MG	EHL	-0.0067	0.002436	-2.766
PY27	PY42	MG	EHL	-0.0116	0.002803	-4.121	PY54	PY69	MG	EHL	-0.0031	0.002916	-1.052
PY27	PY43	MG	EHL	-0.0107	0.002823	-3.779	PY54	PY70	MG	EHL	0.0519	0.005888	8.819
PY27	PY44	MG	EHL	-0.0118	0.002587	-4.568	PY54	PY71	MG	EHL	-0.0109	0.002649	-4.099
PY27	PY45	MG	EHL	-0.0112	0.003038	-3.703	PY55	PY02	MG	EHL	-0.0068	0.002578	-2.624
PY27	PY46	MG	EHL	-0.0069	0.002763	-2.488	PY55	PY05	MG	EHL	0.0021	0.003078	0.685
PY27	PY47	MG	EHL	-0.012	0.00272	-4.399	PY55	PY06	MG	EHL	0.0042	0.003291	1.282
PY27	PY48	MG	EHL	-0.0124	0.00281	-4.409	PY55	PY10	MG	EHL	-0.0056	0.002694	-2.073
PY27	PY49	MG	EHL	-0.008	0.002872	-2.79	PY55	PY17	MG	EHL	-0.0218	0.002236	-9.745
PY27	PY50	MG	EHL	-0.0125	0.002585	-4.85	PY55	PY22	MG	EHL	-0.0081	0.002513	-3.204
PY27	PY51	MG	EHL	-0.0154	0.002952	-5.212	PY55	PY23	MG	EHL	-0.0064	0.002474	-2.574
PY27	PY52	MG	EHL	-0.0071	0.002798	-2.541	PY55	PY24	MG	EHL	-0.0008	0.002546	-0.306
PY27	PY53	MG	EHL	-0.0076	0.002885	-2.626	PY55	PY25	MG	EHL	-0.0039	0.0025	-1.553
PY27	PY54	MG	EHL	-0.0084	0.002834	-2.949	PY55	PY26	MG	EHL	-0.0024	0.002419	-0.993
PY27	PY55	MG	EHL	-0.0085	0.002718	-3.132	PY55	PY27	MG	EHL	0.0085	0.002718	3.132
PY27	PY56	MG	EHL	-0.0106	0.0027	-3.927	PY55	PY28	MG	EHL	-0.0016	0.002993	-0.525
PY27	PY57	MG	EHL	-0.0098	0.002689	-3.656	PY55	PY29	MG	EHL	-0.002	0.002383	-0.818
PY27	PY58	MG	EHL	-0.0088	0.002647	-3.33	PY55	PY30	MG	EHL	-0.0061	0.002261	-2.692
PY27	PY59	MG	EHL	-0.0122	0.002839	-4.286	PY55	PY31	MG	EHL	0	0.002603	0.012
PY27	PY60	MG	EHL	-0.0103	0.002685	-3.822	PY55	PY32	MG	EHL	0.001	0.003011	0.327
PY27	PY61	MG	EHL	-0.0111	0.00285	-3.899	PY55	PY33	MG	EHL	-0.0036	0.002428	-1.475
PY27	PY62	MG	EHL	-0.0075	0.003196	-2.356	PY55	PY34	MG	EHL	0.0345	0.002512	13.719

PY27	PY63	MG	EHL	-0.0125	0.002726	-4.59	PY55	PY36	MG	EHL	-0.0029	0.00278	-1.043
PY27	PY67	MG	EHL	-0.012	0.00302	-3.971	PY55	PY37	MG	EHL	0.0016	0.002837	0.58
PY27	PY68	MG	EHL	-0.015	0.002744	-5.479	PY55	PY39	MG	EHL	-0.0215	0.002299	-9.345
PY27	PY69	MG	EHL	-0.0114	0.003111	-3.668	PY55	PY40	MG	EHL	-0.0005	0.002777	-0.174
PY27	PY70	MG	EHL	0.0444	0.006091	7.291	PY55	PY42	MG	EHL	-0.0029	0.00238	-1.2
PY27	PY71	MG	EHL	-0.0188	0.002839	-6.62	PY55	PY43	MG	EHL	-0.0021	0.002442	-0.84
PY28	PY02	MG	EHL	-0.006	0.002935	-2.038	PY55	PY44	MG	EHL	-0.003	0.002263	-1.324
PY28	PY05	MG	EHL	0.0038	0.003451	1.096	PY55	PY45	MG	EHL	-0.0023	0.002703	-0.841
PY28	PY06	MG	EHL	0.0061	0.003709	1.658	PY55	PY46	MG	EHL	0.0023	0.002702	0.868
PY28	PY10	MG	EHL	-0.0041	0.003168	-1.307	PY55	PY47	MG	EHL	-0.0038	0.002374	-1.597
PY28	PY17	MG	EHL	-0.02	0.002734	-7.333	PY55	PY48	MG	EHL	-0.0033	0.002483	-1.323
PY28	PY22	MG	EHL	-0.0064	0.003003	-2.139	PY55	PY49	MG	EHL	0.0013	0.002755	0.465
PY28	PY23	MG	EHL	-0.0051	0.003093	-1.652	PY55	PY50	MG	EHL	-0.0033	0.002203	-1.491
PY28	PY24	MG	EHL	0.0023	0.002864	0.81	PY55	PY51	MG	EHL	-0.0052	0.002501	-2.073
PY28	PY25	MG	EHL	-0.0027	0.003119	-0.853	PY55	PY52	MG	EHL	0.0016	0.002642	0.598
PY28	PY26	MG	EHL	-0.001	0.003112	-0.328	PY55	PY53	MG	EHL	0.0013	0.002568	0.519
PY28	PY27	MG	EHL	0.0114	0.003133	3.629	PY55	PY54	MG	EHL	0.0005	0.002631	0.178
PY28	PY29	MG	EHL	0	0.002948	0.016	PY55	PY56	MG	EHL	-0.0019	0.002223	-0.864
PY28	PY30	MG	EHL	-0.0045	0.003075	-1.471	PY55	PY57	MG	EHL	-0.0008	0.00232	-0.346
PY28	PY31	MG	EHL	0.0021	0.003129	0.684	PY55	PY58	MG	EHL	-0.0002	0.002368	-0.081
PY28	PY32	MG	EHL	0.0029	0.002555	1.123	PY55	PY59	MG	EHL	-0.004	0.002408	-1.677
PY28	PY33	MG	EHL	-0.002	0.00301	-0.677	PY55	PY60	MG	EHL	-0.0018	0.002358	-0.749
PY28	PY34	MG	EHL	0.0357	0.002903	12.282	PY55	PY61	MG	EHL	-0.0027	0.002471	-1.112
PY28	PY36	MG	EHL	-0.001	0.003223	-0.322	PY55	PY62	MG	EHL	0.0016	0.00292	0.543
PY28	PY37	MG	EHL	0.0028	0.003384	0.838	PY55	PY63	MG	EHL	-0.004	0.002387	-1.662

PY28	PY39	MG	EHL	-0.0203	0.002786	-7.289	PY55	PY67	MG	EHL	-0.0035	0.002696	-1.286
PY28	PY40	MG	EHL	0.002	0.003378	0.6	PY55	PY68	MG	EHL	-0.0061	0.002343	-2.599
PY28	PY42	MG	EHL	-0.0016	0.003041	-0.514	PY55	PY69	MG	EHL	-0.0026	0.002755	-0.952
PY28	PY43	MG	EHL	-0.0002	0.003031	-0.073	PY55	PY70	MG	EHL	0.0525	0.00592	8.863
PY28	PY44	MG	EHL	-0.0014	0.002921	-0.484	PY55	PY71	MG	EHL	-0.0104	0.002565	-4.043
PY28	PY45	MG	EHL	-0.0006	0.003243	-0.171	PY56	PY02	MG	EHL	-0.0052	0.002391	-2.156
PY28	PY46	MG	EHL	0.0036	0.003042	1.189	PY56	PY05	MG	EHL	0.0042	0.003068	1.383
PY28	PY47	MG	EHL	-0.0017	0.003094	-0.552	PY56	PY06	MG	EHL	0.0065	0.003167	2.047
PY28	PY48	MG	EHL	-0.0017	0.002948	-0.565	PY56	PY10	MG	EHL	-0.0036	0.002717	-1.322
PY28	PY49	MG	EHL	0.0032	0.002628	1.233	PY56	PY17	MG	EHL	-0.0201	0.002122	-9.464
PY28	PY50	MG	EHL	-0.0018	0.002892	-0.627	PY56	PY22	MG	EHL	-0.0059	0.002482	-2.358
PY28	PY51	MG	EHL	-0.004	0.003218	-1.25	PY56	PY23	MG	EHL	-0.0043	0.002497	-1.736
PY28	PY52	MG	EHL	0.0024	0.00309	0.784	PY56	PY24	MG	EHL	0.0012	0.002471	0.489
PY28	PY53	MG	EHL	0.0026	0.003129	0.816	PY56	PY25	MG	EHL	-0.0019	0.00244	-0.787
PY28	PY54	MG	EHL	0.0022	0.003068	0.731	PY56	PY26	MG	EHL	-0.0007	0.002371	-0.304
PY28	PY55	MG	EHL	0.0016	0.002993	0.525	PY56	PY27	MG	EHL	0.0106	0.0027	3.927
PY28	PY56	MG	EHL	-0.0004	0.002872	-0.13	PY56	PY28	MG	EHL	0.0004	0.002872	0.13
PY28	PY57	MG	EHL	0.001	0.002878	0.343	PY56	PY29	MG	EHL	0.0006	0.002312	0.253
PY28	PY58	MG	EHL	0.0012	0.002963	0.396	PY56	PY30	MG	EHL	-0.0041	0.002165	-1.879
PY28	PY59	MG	EHL	-0.0022	0.002972	-0.725	PY56	PY31	MG	EHL	0.002	0.002489	0.801
PY28	PY60	MG	EHL	-0.0001	0.002851	-0.027	PY56	PY32	MG	EHL	0.003	0.003009	1.013
PY28	PY61	MG	EHL	-0.0005	0.003052	-0.167	PY56	PY33	MG	EHL	-0.0014	0.00224	-0.624
PY28	PY62	MG	EHL	0.0026	0.003377	0.774	PY56	PY34	MG	EHL	0.0361	0.002439	14.798
PY28	PY63	MG	EHL	-0.0023	0.003091	-0.729	PY56	PY36	MG	EHL	-0.0007	0.002871	-0.241
PY28	PY67	MG	EHL	-0.0016	0.003155	-0.496	PY56	PY37	MG	EHL	0.0034	0.003053	1.112

PY28	PY68	MG	EHL	-0.0046	0.003001	-1.549	PY56	PY39	MG	EHL	-0.0196	0.002238	-8.756
PY28	PY69	MG	EHL	-0.0009	0.003334	-0.276	PY56	PY40	MG	EHL	0.0016	0.002815	0.561
PY28	PY70	MG	EHL	0.0543	0.006131	8.859	PY56	PY42	MG	EHL	-0.0013	0.002403	-0.555
PY28	PY71	MG	EHL	-0.009	0.003105	-2.893	PY56	PY43	MG	EHL	0	0.002388	-0.018
PY29	PY02	MG	EHL	-0.0056	0.002422	-2.297	PY56	PY44	MG	EHL	-0.0012	0.002271	-0.535
PY29	PY05	MG	EHL	0.0038	0.002903	1.292	PY56	PY45	MG	EHL	-0.0004	0.002629	-0.15
PY29	PY06	MG	EHL	0.0059	0.00313	1.897	PY56	PY46	MG	EHL	0.0042	0.002633	1.578
PY29	PY10	MG	EHL	-0.0042	0.002615	-1.623	PY56	PY47	MG	EHL	-0.0019	0.00247	-0.775
PY29	PY17	MG	EHL	-0.0209	0.002122	-9.828	PY56	PY48	MG	EHL	-0.0013	0.002561	-0.517
PY29	PY22	MG	EHL	-0.0065	0.002544	-2.537	PY56	PY49	MG	EHL	0.0032	0.0027	1.183
PY29	PY23	MG	EHL	-0.0049	0.002494	-1.962	PY56	PY50	MG	EHL	-0.0014	0.002259	-0.605
PY29	PY24	MG	EHL	0.0009	0.002518	0.371	PY56	PY51	MG	EHL	-0.003	0.002442	-1.248
PY29	PY25	MG	EHL	-0.0026	0.002368	-1.079	PY56	PY52	MG	EHL	0.0037	0.002709	1.352
PY29	PY26	MG	EHL	-0.0008	0.002344	-0.348	PY56	PY53	MG	EHL	0.0037	0.00262	1.429
PY29	PY27	MG	EHL	0.0106	0.002598	4.094	PY56	PY54	MG	EHL	0.0023	0.002542	0.888
PY29	PY28	MG	EHL	0	0.002948	-0.016	PY56	PY55	MG	EHL	0.0019	0.002223	0.864
PY29	PY30	MG	EHL	-0.0052	0.002127	-2.454	PY56	PY57	MG	EHL	0.0013	0.002281	0.559
PY29	PY31	MG	EHL	0.0017	0.002562	0.649	PY56	PY58	MG	EHL	0.0016	0.002399	0.657
PY29	PY32	MG	EHL	0.0029	0.002959	0.98	PY56	PY59	MG	EHL	-0.0018	0.002357	-0.753
PY29	PY33	MG	EHL	-0.0022	0.002527	-0.888	PY56	PY60	MG	EHL	0.0005	0.002406	0.211
PY29	PY34	MG	EHL	0.0358	0.002549	14.06	PY56	PY61	MG	EHL	0	0.002424	-0.013
PY29	PY36	MG	EHL	-0.0012	0.002718	-0.424	PY56	PY62	MG	EHL	0.0036	0.003022	1.175
PY29	PY37	MG	EHL	0.0028	0.003015	0.915	PY56	PY63	MG	EHL	-0.0016	0.002439	-0.636
PY29	PY39	MG	EHL	-0.0201	0.002106	-9.543	PY56	PY67	MG	EHL	-0.0012	0.002649	-0.446
PY29	PY40	MG	EHL	0.0013	0.002889	0.441	PY56	PY68	MG	EHL	-0.0044	0.002265	-1.927

PY29	PY42	MG	EHL	-0.0016	0.00247	-0.667	PY56	PY69	MG	EHL	-0.0008	0.002831	-0.27
PY29	PY43	MG	EHL	-0.0004	0.002446	-0.172	PY56	PY70	MG	EHL	0.054	0.005881	9.186
PY29	PY44	MG	EHL	-0.0016	0.002331	-0.694	PY56	PY71	MG	EHL	-0.0085	0.002535	-3.345
PY29	PY45	MG	EHL	-0.001	0.002576	-0.383	PY57	PY02	MG	EHL	-0.0066	0.002419	-2.723
PY29	PY46	MG	EHL	0.0039	0.002595	1.485	PY57	PY05	MG	EHL	0.003	0.003026	0.997
PY29	PY47	MG	EHL	-0.0019	0.002435	-0.78	PY57	PY06	MG	EHL	0.0054	0.003268	1.65
PY29	PY48	MG	EHL	-0.0018	0.002459	-0.722	PY57	PY10	MG	EHL	-0.005	0.002558	-1.956
PY29	PY49	MG	EHL	0.0029	0.002704	1.062	PY57	PY17	MG	EHL	-0.0214	0.002096	-10.218
PY29	PY50	MG	EHL	-0.0019	0.002291	-0.848	PY57	PY22	MG	EHL	-0.0072	0.002543	-2.834
PY29	PY51	MG	EHL	-0.0042	0.00266	-1.596	PY57	PY23	MG	EHL	-0.0056	0.002507	-2.233
PY29	PY52	MG	EHL	0.0027	0.002606	1.046	PY57	PY24	MG	EHL	0.0004	0.002422	0.145
PY29	PY53	MG	EHL	0.0022	0.002659	0.831	PY57	PY25	MG	EHL	-0.0032	0.002529	-1.255
PY29	PY54	MG	EHL	0.002	0.002543	0.804	PY57	PY26	MG	EHL	-0.0022	0.002468	-0.91
PY29	PY55	MG	EHL	0.002	0.002383	0.818	PY57	PY27	MG	EHL	0.0098	0.002689	3.656
PY29	PY56	MG	EHL	-0.0006	0.002312	-0.253	PY57	PY28	MG	EHL	-0.001	0.002878	-0.343
PY29	PY57	MG	EHL	0.0007	0.002326	0.296	PY57	PY29	MG	EHL	-0.0007	0.002326	-0.296
PY29	PY58	MG	EHL	0.0012	0.002385	0.496	PY57	PY30	MG	EHL	-0.0054	0.002144	-2.531
PY29	PY59	MG	EHL	-0.0019	0.00246	-0.756	PY57	PY31	MG	EHL	0.0005	0.002655	0.184
PY29	PY60	MG	EHL	0.0001	0.0023	0.046	PY57	PY32	MG	EHL	0.0017	0.00295	0.585
PY29	PY61	MG	EHL	-0.0008	0.002459	-0.326	PY57	PY33	MG	EHL	-0.0027	0.002484	-1.085
PY29	PY62	MG	EHL	0.0027	0.002836	0.945	PY57	PY34	MG	EHL	0.0346	0.002468	14.012
PY29	PY63	MG	EHL	-0.0025	0.002523	-0.976	PY57	PY36	MG	EHL	-0.0022	0.002857	-0.774
PY29	PY67	MG	EHL	-0.0017	0.002748	-0.609	PY57	PY37	MG	EHL	0.0019	0.002808	0.67
PY29	PY68	MG	EHL	-0.0048	0.002467	-1.934	PY57	PY39	MG	EHL	-0.0207	0.002152	-9.601
PY29	PY69	MG	EHL	-0.001	0.002804	-0.351	PY57	PY40	MG	EHL	-0.0003	0.002917	-0.1

PY29	PY70	MG	EHL	0.0538	0.005849	9.203	PY57	PY42	MG	EHL	-0.0023	0.002461	-0.94
PY29	PY71	MG	EHL	-0.0092	0.002566	-3.604	PY57	PY43	MG	EHL	-0.0012	0.002513	-0.472
PY30	PY02	MG	EHL	-0.0013	0.002297	-0.552	PY57	PY44	MG	EHL	-0.0022	0.002205	-1.01
PY30	PY05	MG	EHL	0.008	0.002862	2.811	PY57	PY45	MG	EHL	-0.0014	0.002672	-0.512
PY30	PY06	MG	EHL	0.0103	0.003178	3.235	PY57	PY46	MG	EHL	0.0032	0.002637	1.231
PY30	PY10	MG	EHL	0.0001	0.00249	0.034	PY57	PY47	MG	EHL	-0.003	0.002564	-1.166
PY30	PY17	MG	EHL	-0.0167	0.002093	-7.955	PY57	PY48	MG	EHL	-0.0025	0.002539	-1.004
PY30	PY22	MG	EHL	-0.0019	0.002489	-0.782	PY57	PY49	MG	EHL	0.0018	0.002759	0.67
PY30	PY23	MG	EHL	-0.0005	0.002495	-0.185	PY57	PY50	MG	EHL	-0.0027	0.00222	-1.196
PY30	PY24	MG	EHL	0.0065	0.002401	2.687	PY57	PY51	MG	EHL	-0.0048	0.0025	-1.933
PY30	PY25	MG	EHL	0.0022	0.002446	0.88	PY57	PY52	MG	EHL	0.0021	0.002735	0.772
PY30	PY26	MG	EHL	0.0036	0.002256	1.574	PY57	PY53	MG	EHL	0.0017	0.002619	0.662
PY30	PY27	MG	EHL	0.0158	0.002641	5.995	PY57	PY54	MG	EHL	0.0013	0.002521	0.528
PY30	PY28	MG	EHL	0.0045	0.003075	1.471	PY57	PY55	MG	EHL	0.0008	0.00232	0.346
PY30	PY29	MG	EHL	0.0052	0.002127	2.454	PY57	PY56	MG	EHL	-0.0013	0.002281	-0.559
PY30	PY31	MG	EHL	0.0066	0.002561	2.561	PY57	PY58	MG	EHL	0.0005	0.002341	0.217
PY30	PY32	MG	EHL	0.0074	0.002993	2.466	PY57	PY59	MG	EHL	-0.0031	0.00239	-1.308
PY30	PY33	MG	EHL	0.0026	0.002361	1.115	PY57	PY60	MG	EHL	-0.0007	0.002389	-0.296
PY30	PY34	MG	EHL	0.0398	0.002337	17.05	PY57	PY61	MG	EHL	-0.0016	0.002362	-0.657
PY30	PY36	MG	EHL	0.0033	0.002678	1.246	PY57	PY62	MG	EHL	0.002	0.002932	0.665
PY30	PY37	MG	EHL	0.0072	0.002881	2.505	PY57	PY63	MG	EHL	-0.003	0.002354	-1.281
PY30	PY39	MG	EHL	-0.0161	0.002123	-7.586	PY57	PY67	MG	EHL	-0.0025	0.00276	-0.917
PY30	PY40	MG	EHL	0.0055	0.002782	1.984	PY57	PY68	MG	EHL	-0.0055	0.002234	-2.448
PY30	PY42	MG	EHL	0.0025	0.002405	1.053	PY57	PY69	MG	EHL	-0.0021	0.002751	-0.747
PY30	PY43	MG	EHL	0.0042	0.002478	1.686	PY57	PY70	MG	EHL	0.053	0.005827	9.089

PY30	PY44	MG	EHL	0.003	0.00221	1.336	PY57	PY71	MG	EHL	-0.0103	0.002544	-4.04
PY30	PY45	MG	EHL	0.0035	0.002612	1.358	PY58	PY02	MG	EHL	-0.0067	0.002437	-2.747
PY30	PY46	MG	EHL	0.0083	0.002508	3.328	PY58	PY05	MG	EHL	0.0024	0.003026	0.79
PY30	PY47	MG	EHL	0.0025	0.002232	1.111	PY58	PY06	MG	EHL	0.0048	0.003238	1.492
PY30	PY48	MG	EHL	0.0028	0.002372	1.177	PY58	PY10	MG	EHL	-0.0054	0.002729	-1.973
PY30	PY49	MG	EHL	0.0077	0.002701	2.836	PY58	PY17	MG	EHL	-0.0219	0.002212	-9.882
PY30	PY50	MG	EHL	0.0027	0.002143	1.264	PY58	PY22	MG	EHL	-0.0076	0.00254	-2.972
PY30	PY51	MG	EHL	0.0007	0.002568	0.259	PY58	PY23	MG	EHL	-0.006	0.002633	-2.284
PY30	PY52	MG	EHL	0.007	0.00246	2.853	PY58	PY24	MG	EHL	0	0.002415	-0.002
PY30	PY53	MG	EHL	0.0073	0.002509	2.927	PY58	PY25	MG	EHL	-0.0036	0.002544	-1.409
PY30	PY54	MG	EHL	0.0064	0.002478	2.566	PY58	PY26	MG	EHL	-0.0021	0.002476	-0.854
PY30	PY55	MG	EHL	0.0061	0.002261	2.692	PY58	PY27	MG	EHL	0.0088	0.002647	3.33
PY30	PY56	MG	EHL	0.0041	0.002165	1.879	PY58	PY28	MG	EHL	-0.0012	0.002963	-0.396
PY30	PY57	MG	EHL	0.0054	0.002144	2.531	PY58	PY29	MG	EHL	-0.0012	0.002385	-0.496
PY30	PY58	MG	EHL	0.0056	0.00219	2.556	PY58	PY30	MG	EHL	-0.0056	0.00219	-2.556
PY30	PY59	MG	EHL	0.0025	0.002334	1.067	PY58	PY31	MG	EHL	0.0005	0.002618	0.172
PY30	PY60	MG	EHL	0.0045	0.002276	1.964	PY58	PY32	MG	EHL	0.0016	0.002953	0.528
PY30	PY61	MG	EHL	0.0039	0.002379	1.63	PY58	PY33	MG	EHL	-0.0032	0.002434	-1.316
PY30	PY62	MG	EHL	0.0072	0.002806	2.579	PY58	PY34	MG	EHL	0.0348	0.002582	13.46
PY30	PY63	MG	EHL	0.0027	0.002468	1.074	PY58	PY36	MG	EHL	-0.0024	0.002851	-0.836
PY30	PY67	MG	EHL	0.0028	0.002636	1.051	PY58	PY37	MG	EHL	0.0016	0.002897	0.536
PY30	PY68	MG	EHL	-0.0003	0.002301	-0.13	PY58	PY39	MG	EHL	-0.0212	0.00228	-9.299
PY30	PY69	MG	EHL	0.0035	0.002689	1.296	PY58	PY40	MG	EHL	-0.0003	0.00297	-0.091
PY30	PY70	MG	EHL	0.058	0.005842	9.934	PY58	PY42	MG	EHL	-0.0031	0.00264	-1.19
PY30	PY71	MG	EHL	-0.0049	0.002435	-2.002	PY58	PY43	MG	EHL	-0.0016	0.002513	-0.638

PY31	PY02	MG	EHL	-0.0072	0.002793	-2.59	PY58	PY44	MG	EHL	-0.0028	0.002246	-1.236
PY31	PY05	MG	EHL	0.002	0.003272	0.611	PY58	PY45	MG	EHL	-0.0023	0.002713	-0.831
PY31	PY06	MG	EHL	0.004	0.003423	1.176	PY58	PY46	MG	EHL	0.0026	0.002678	0.958
PY31	PY10	MG	EHL	-0.0059	0.002753	-2.146	PY58	PY47	MG	EHL	-0.0035	0.002438	-1.431
PY31	PY17	MG	EHL	-0.0216	0.002342	-9.221	PY58	PY48	MG	EHL	-0.003	0.002545	-1.191
PY31	PY22	MG	EHL	-0.0082	0.002674	-3.063	PY58	PY49	MG	EHL	0.0015	0.002813	0.538
PY31	PY23	MG	EHL	-0.0065	0.002615	-2.498	PY58	PY50	MG	EHL	-0.0029	0.002329	-1.241
PY31	PY24	MG	EHL	-0.0002	0.002629	-0.066	PY58	PY51	MG	EHL	-0.0048	0.00254	-1.881
PY31	PY25	MG	EHL	-0.0044	0.002849	-1.555	PY58	PY52	MG	EHL	0.0017	0.00272	0.638
PY31	PY26	MG	EHL	-0.0032	0.002682	-1.197	PY58	PY53	MG	EHL	0.0014	0.002654	0.53
PY31	PY27	MG	EHL	0.0091	0.002846	3.184	PY58	PY54	MG	EHL	0.0007	0.002571	0.258
PY31	PY28	MG	EHL	-0.0021	0.003129	-0.684	PY58	PY55	MG	EHL	0.0002	0.002368	0.081
PY31	PY29	MG	EHL	-0.0017	0.002562	-0.649	PY58	PY56	MG	EHL	-0.0016	0.002399	-0.657
PY31	PY30	MG	EHL	-0.0066	0.002561	-2.561	PY58	PY57	MG	EHL	-0.0005	0.002341	-0.217
PY31	PY32	MG	EHL	0.001	0.003208	0.298	PY58	PY59	MG	EHL	-0.0036	0.002492	-1.464
PY31	PY33	MG	EHL	-0.0039	0.002644	-1.458	PY58	PY60	MG	EHL	-0.0014	0.00241	-0.585
PY31	PY34	MG	EHL	0.0343	0.002639	12.984	PY58	PY61	MG	EHL	-0.0019	0.002397	-0.803
PY31	PY36	MG	EHL	-0.0028	0.002952	-0.946	PY58	PY62	MG	EHL	0.0017	0.002949	0.59
PY31	PY37	MG	EHL	0.001	0.00319	0.303	PY58	PY63	MG	EHL	-0.0033	0.002439	-1.341
PY31	PY39	MG	EHL	-0.0218	0.002343	-9.31	PY58	PY67	MG	EHL	-0.0029	0.002748	-1.06
PY31	PY40	MG	EHL	-0.0008	0.003081	-0.263	PY58	PY68	MG	EHL	-0.006	0.002301	-2.595
PY31	PY42	MG	EHL	-0.0033	0.002853	-1.17	PY58	PY69	MG	EHL	-0.0023	0.002747	-0.829
PY31	PY43	MG	EHL	-0.0023	0.002633	-0.872	PY58	PY70	MG	EHL	0.0529	0.005868	9.024
PY31	PY44	MG	EHL	-0.0035	0.002637	-1.346	PY58	PY71	MG	EHL	-0.0103	0.002482	-4.141
PY31	PY45	MG	EHL	-0.0028	0.002687	-1.051	PY59	PY02	MG	EHL	-0.0036	0.002581	-1.412

PY31	PY46	MG	EHL	0.0019	0.002744	0.707	PY59	PY05	MG	EHL	0.0056	0.003061	1.828
PY31	PY47	MG	EHL	-0.0035	0.00267	-1.319	PY59	PY06	MG	EHL	0.0076	0.003254	2.333
PY31	PY48	MG	EHL	-0.0035	0.002835	-1.244	PY59	PY10	MG	EHL	-0.0023	0.002686	-0.857
PY31	PY49	MG	EHL	0.0011	0.002979	0.374	PY59	PY17	MG	EHL	-0.0188	0.002112	-8.909
PY31	PY50	MG	EHL	-0.0037	0.002467	-1.495	PY59	PY22	MG	EHL	-0.0043	0.002608	-1.663
PY31	PY51	MG	EHL	-0.0054	0.00283	-1.915	PY59	PY23	MG	EHL	-0.003	0.002551	-1.165
PY31	PY52	MG	EHL	0.0012	0.002894	0.403	PY59	PY24	MG	EHL	0.0034	0.002522	1.342
PY31	PY53	MG	EHL	0.0006	0.002762	0.201	PY59	PY25	MG	EHL	-0.0002	0.002521	-0.077
PY31	PY54	MG	EHL	0.0003	0.002596	0.109	PY59	PY26	MG	EHL	0.0009	0.002437	0.384
PY31	PY55	MG	EHL	0	0.002603	-0.012	PY59	PY27	MG	EHL	0.0122	0.002839	4.286
PY31	PY56	MG	EHL	-0.002	0.002489	-0.801	PY59	PY28	MG	EHL	0.0022	0.002972	0.725
PY31	PY57	MG	EHL	-0.0005	0.002655	-0.184	PY59	PY29	MG	EHL	0.0019	0.00246	0.756
PY31	PY58	MG	EHL	-0.0005	0.002618	-0.172	PY59	PY30	MG	EHL	-0.0025	0.002334	-1.067
PY31	PY59	MG	EHL	-0.0034	0.00266	-1.292	PY59	PY31	MG	EHL	0.0034	0.00266	1.292
PY31	PY60	MG	EHL	-0.0019	0.00265	-0.702	PY59	PY32	MG	EHL	0.0046	0.003055	1.514
PY31	PY61	MG	EHL	-0.0024	0.002785	-0.869	PY59	PY33	MG	EHL	0.0001	0.002462	0.058
PY31	PY62	MG	EHL	0.0009	0.003162	0.291	PY59	PY34	MG	EHL	0.0377	0.002561	14.706
PY31	PY63	MG	EHL	-0.0037	0.002715	-1.354	PY59	PY36	MG	EHL	0.0008	0.002757	0.301
PY31	PY67	MG	EHL	-0.0033	0.002888	-1.155	PY59	PY37	MG	EHL	0.0053	0.002892	1.831
PY31	PY68	MG	EHL	-0.0065	0.002687	-2.413	PY59	PY39	MG	EHL	-0.0181	0.002241	-8.07
PY31	PY69	MG	EHL	-0.0027	0.003064	-0.87	PY59	PY40	MG	EHL	0.0035	0.002738	1.267
PY31	PY70	MG	EHL	0.0521	0.006038	8.629	PY59	PY42	MG	EHL	0.0004	0.002586	0.138
PY31	PY71	MG	EHL	-0.011	0.002826	-3.91	PY59	PY43	MG	EHL	0.0019	0.002515	0.763
PY32	PY02	MG	EHL	-0.0082	0.002995	-2.729	PY59	PY44	MG	EHL	0.0005	0.002276	0.234
PY32	PY05	MG	EHL	0.0012	0.00348	0.336	PY59	PY45	MG	EHL	0.0011	0.002738	0.402

PY32	PY06	MG	EHL	0.0035	0.003746	0.933	PY59	PY46	MG	EHL	0.006	0.002558	2.33
PY32	PY10	MG	EHL	-0.0068	0.003282	-2.083	PY59	PY47	MG	EHL	-0.0003	0.002514	-0.118
PY32	PY17	MG	EHL	-0.0231	0.002718	-8.503	PY59	PY48	MG	EHL	0.0002	0.002512	0.095
PY32	PY22	MG	EHL	-0.009	0.003197	-2.823	PY59	PY49	MG	EHL	0.005	0.002801	1.796
PY32	PY23	MG	EHL	-0.0077	0.003138	-2.462	PY59	PY50	MG	EHL	-0.0002	0.002322	-0.084
PY32	PY24	MG	EHL	-0.0012	0.002959	-0.391	PY59	PY51	MG	EHL	-0.0012	0.002593	-0.461
PY32	PY25	MG	EHL	-0.0053	0.002803	-1.879	PY59	PY52	MG	EHL	0.0049	0.002683	1.822
PY32	PY26	MG	EHL	-0.004	0.003173	-1.259	PY59	PY53	MG	EHL	0.0048	0.002734	1.769
PY32	PY27	MG	EHL	0.0075	0.003215	2.343	PY59	PY54	MG	EHL	0.0043	0.0027	1.596
PY32	PY28	MG	EHL	-0.0029	0.002555	-1.123	PY59	PY55	MG	EHL	0.004	0.002408	1.677
PY32	PY29	MG	EHL	-0.0029	0.002959	-0.98	PY59	PY56	MG	EHL	0.0018	0.002357	0.753
PY32	PY30	MG	EHL	-0.0074	0.002993	-2.466	PY59	PY57	MG	EHL	0.0031	0.00239	1.308
PY32	PY31	MG	EHL	-0.001	0.003208	-0.298	PY59	PY58	MG	EHL	0.0036	0.002492	1.464
PY32	PY33	MG	EHL	-0.0048	0.00304	-1.578	PY59	PY60	MG	EHL	0.0024	0.002442	0.982
PY32	PY34	MG	EHL	0.0335	0.002958	11.311	PY59	PY61	MG	EHL	0.0014	0.002493	0.546
PY32	PY36	MG	EHL	-0.0039	0.00331	-1.167	PY59	PY62	MG	EHL	0.0052	0.002916	1.79
PY32	PY37	MG	EHL	0	0.003427	0.012	PY59	PY63	MG	EHL	-0.0003	0.002397	-0.114
PY32	PY39	MG	EHL	-0.0226	0.002786	-8.093	PY59	PY67	MG	EHL	0.0002	0.002764	0.081
PY32	PY40	MG	EHL	-0.0013	0.003308	-0.379	PY59	PY68	MG	EHL	-0.003	0.002384	-1.261
PY32	PY42	MG	EHL	-0.0044	0.00311	-1.419	PY59	PY69	MG	EHL	0.0008	0.002765	0.289
PY32	PY43	MG	EHL	-0.0033	0.003147	-1.038	PY59	PY70	MG	EHL	0.0561	0.005915	9.48
PY32	PY44	MG	EHL	-0.0044	0.002884	-1.508	PY59	PY71	MG	EHL	-0.007	0.002558	-2.739
PY32	PY45	MG	EHL	-0.0038	0.00329	-1.145	PY60	PY02	MG	EHL	-0.0057	0.002493	-2.296
PY32	PY46	MG	EHL	0.0012	0.003157	0.392	PY60	PY05	MG	EHL	0.0037	0.002986	1.248
PY32	PY47	MG	EHL	-0.0045	0.003074	-1.45	PY60	PY06	MG	EHL	0.0059	0.003354	1.757

PY32	PY48	MG	EHL	-0.0046	0.003014	-1.534	PY60	PY10	MG	EHL	-0.0041	0.002723	-1.49
PY32	PY49	MG	EHL	0.0001	0.002605	0.046	PY60	PY17	MG	EHL	-0.0203	0.002125	-9.548
PY32	PY50	MG	EHL	-0.0047	0.002956	-1.58	PY60	PY22	MG	EHL	-0.0065	0.002407	-2.687
PY32	PY51	MG	EHL	-0.0068	0.003261	-2.074	PY60	PY23	MG	EHL	-0.0048	0.002411	-1.986
PY32	PY52	MG	EHL	0.0003	0.003158	0.106	PY60	PY24	MG	EHL	0.0006	0.002454	0.247
PY32	PY53	MG	EHL	-0.0003	0.003116	-0.089	PY60	PY25	MG	EHL	-0.0025	0.002484	-1.004
PY32	PY54	MG	EHL	-0.0006	0.003185	-0.2	PY60	PY26	MG	EHL	-0.0012	0.002397	-0.517
PY32	PY55	MG	EHL	-0.001	0.003011	-0.327	PY60	PY27	MG	EHL	0.0103	0.002685	3.822
PY32	PY56	MG	EHL	-0.003	0.003009	-1.013	PY60	PY28	MG	EHL	0.0001	0.002851	0.027
PY32	PY57	MG	EHL	-0.0017	0.00295	-0.585	PY60	PY29	MG	EHL	-0.0001	0.0023	-0.046
PY32	PY58	MG	EHL	-0.0016	0.002953	-0.528	PY60	PY30	MG	EHL	-0.0045	0.002276	-1.964
PY32	PY59	MG	EHL	-0.0046	0.003055	-1.514	PY60	PY31	MG	EHL	0.0019	0.00265	0.702
PY32	PY60	MG	EHL	-0.0028	0.002965	-0.936	PY60	PY32	MG	EHL	0.0028	0.002965	0.936
PY32	PY61	MG	EHL	-0.0035	0.003092	-1.132	PY60	PY33	MG	EHL	-0.002	0.002348	-0.852
PY32	PY62	MG	EHL	0	0.003418	-0.003	PY60	PY34	MG	EHL	0.0358	0.002536	14.119
PY32	PY63	MG	EHL	-0.005	0.003055	-1.648	PY60	PY36	MG	EHL	-0.001	0.002836	-0.361
PY32	PY67	MG	EHL	-0.0045	0.003339	-1.342	PY60	PY37	MG	EHL	0.0031	0.002861	1.085
PY32	PY68	MG	EHL	-0.0076	0.003068	-2.475	PY60	PY39	MG	EHL	-0.02	0.002272	-8.782
PY32	PY69	MG	EHL	-0.0036	0.003322	-1.093	PY60	PY40	MG	EHL	0.0013	0.002885	0.456
PY32	PY70	MG	EHL	0.0514	0.006336	8.118	PY60	PY42	MG	EHL	-0.0011	0.00247	-0.457
PY32	PY71	MG	EHL	-0.0118	0.003058	-3.874	PY60	PY43	MG	EHL	-0.0004	0.002468	-0.174
PY33	PY02	MG	EHL	-0.0036	0.002422	-1.507	PY60	PY44	MG	EHL	-0.0014	0.002232	-0.619
PY33	PY05	MG	EHL	0.0053	0.00311	1.702	PY60	PY45	MG	EHL	-0.0009	0.002666	-0.329
PY33	PY06	MG	EHL	0.0079	0.003251	2.427	PY60	PY46	MG	EHL	0.0038	0.002652	1.427
PY33	PY10	MG	EHL	-0.0023	0.002697	-0.845	PY60	PY47	MG	EHL	-0.0022	0.002369	-0.925

PY33	PY17	MG	EHL	-0.0189	0.002211	-8.535	PY60	PY48	MG	EHL	-0.0018	0.002555	-0.706
PY33	PY22	MG	EHL	-0.0044	0.002579	-1.715	PY60	PY49	MG	EHL	0.0027	0.002789	0.97
PY33	PY23	MG	EHL	-0.0027	0.002516	-1.068	PY60	PY50	MG	EHL	-0.0017	0.002164	-0.763
PY33	PY24	MG	EHL	0.0039	0.002594	1.495	PY60	PY51	MG	EHL	-0.0036	0.002457	-1.467
PY33	PY25	MG	EHL	-0.0004	0.002468	-0.161	PY60	PY52	MG	EHL	0.0029	0.002697	1.07
PY33	PY26	MG	EHL	0.0012	0.002477	0.479	PY60	PY53	MG	EHL	0.003	0.002725	1.098
PY33	PY27	MG	EHL	0.0125	0.002804	4.461	PY60	PY54	MG	EHL	0.0021	0.002508	0.835
PY33	PY28	MG	EHL	0.002	0.00301	0.677	PY60	PY55	MG	EHL	0.0018	0.002358	0.749
PY33	PY29	MG	EHL	0.0022	0.002527	0.888	PY60	PY56	MG	EHL	-0.0005	0.002406	-0.211
PY33	PY30	MG	EHL	-0.0026	0.002361	-1.115	PY60	PY57	MG	EHL	0.0007	0.002389	0.296
PY33	PY31	MG	EHL	0.0039	0.002644	1.458	PY60	PY58	MG	EHL	0.0014	0.00241	0.585
PY33	PY32	MG	EHL	0.0048	0.00304	1.578	PY60	PY59	MG	EHL	-0.0024	0.002442	-0.982
PY33	PY34	MG	EHL	0.0379	0.002414	15.684	PY60	PY61	MG	EHL	-0.0008	0.002443	-0.334
PY33	PY36	MG	EHL	0.0009	0.002691	0.331	PY60	PY62	MG	EHL	0.0033	0.002992	1.088
PY33	PY37	MG	EHL	0.0047	0.003024	1.565	PY60	PY63	MG	EHL	-0.0023	0.002415	-0.969
PY33	PY39	MG	EHL	-0.0183	0.002166	-8.455	PY60	PY67	MG	EHL	-0.0016	0.002693	-0.609
PY33	PY40	MG	EHL	0.0028	0.002797	1	PY60	PY68	MG	EHL	-0.0047	0.002341	-2.027
PY33	PY42	MG	EHL	0.0006	0.002551	0.219	PY60	PY69	MG	EHL	-0.0009	0.002717	-0.316
PY33	PY43	MG	EHL	0.0017	0.002557	0.667	PY60	PY70	MG	EHL	0.0539	0.005876	9.177
PY33	PY44	MG	EHL	0.0005	0.002383	0.204	PY60	PY71	MG	EHL	-0.0089	0.002658	-3.362
PY33	PY45	MG	EHL	0.0011	0.002756	0.397	PY61	PY02	MG	EHL	-0.0044	0.002562	-1.737
PY33	PY46	MG	EHL	0.0058	0.002548	2.281	PY61	PY05	MG	EHL	0.0042	0.003143	1.332
PY33	PY47	MG	EHL	0.0002	0.002373	0.096	PY61	PY06	MG	EHL	0.0066	0.003314	2.006
PY33	PY48	MG	EHL	0.0003	0.002563	0.124	PY61	PY10	MG	EHL	-0.0037	0.002781	-1.335
PY33	PY49	MG	EHL	0.005	0.002616	1.909	PY61	PY17	MG	EHL	-0.0204	0.002338	-8.738

PY33	PY50	MG	EHL	-0.0001	0.002256	-0.042	PY61	PY22	MG	EHL	-0.0057	0.002671	-2.146
PY33	PY51	MG	EHL	-0.0019	0.002411	-0.776	PY61	PY23	MG	EHL	-0.0041	0.002589	-1.593
PY33	PY52	MG	EHL	0.0049	0.002599	1.89	PY61	PY24	MG	EHL	0.0019	0.002621	0.729
PY33	PY53	MG	EHL	0.004	0.002588	1.548	PY61	PY25	MG	EHL	-0.0015	0.002654	-0.581
PY33	PY54	MG	EHL	0.0038	0.002434	1.562	PY61	PY26	MG	EHL	-0.0003	0.002642	-0.123
PY33	PY55	MG	EHL	0.0036	0.002428	1.475	PY61	PY27	MG	EHL	0.0111	0.00285	3.899
PY33	PY56	MG	EHL	0.0014	0.00224	0.624	PY61	PY28	MG	EHL	0.0005	0.003052	0.167
PY33	PY57	MG	EHL	0.0027	0.002484	1.085	PY61	PY29	MG	EHL	0.0008	0.002459	0.326
PY33	PY58	MG	EHL	0.0032	0.002434	1.316	PY61	PY30	MG	EHL	-0.0039	0.002379	-1.63
PY33	PY59	MG	EHL	-0.0001	0.002462	-0.058	PY61	PY31	MG	EHL	0.0024	0.002785	0.869
PY33	PY60	MG	EHL	0.002	0.002348	0.852	PY61	PY32	MG	EHL	0.0035	0.003092	1.132
PY33	PY61	MG	EHL	0.0014	0.002471	0.549	PY61	PY33	MG	EHL	-0.0014	0.002471	-0.549
PY33	PY62	MG	EHL	0.0046	0.002925	1.565	PY61	PY34	MG	EHL	0.0359	0.002647	13.572
PY33	PY63	MG	EHL	-0.0002	0.00244	-0.085	PY61	PY36	MG	EHL	-0.0006	0.00285	-0.219
PY33	PY67	MG	EHL	0.0004	0.002668	0.14	PY61	PY37	MG	EHL	0.0037	0.002887	1.271
PY33	PY68	MG	EHL	-0.0027	0.002506	-1.084	PY61	PY39	MG	EHL	-0.0198	0.002367	-8.364
PY33	PY69	MG	EHL	0.001	0.002833	0.361	PY61	PY40	MG	EHL	0.0013	0.002918	0.456
PY33	PY70	MG	EHL	0.0556	0.005974	9.312	PY61	PY42	MG	EHL	-0.0011	0.002456	-0.434
PY33	PY71	MG	EHL	-0.0072	0.002625	-2.744	PY61	PY43	MG	EHL	0	0.002694	0.011
PY34	PY02	MG	EHL	-0.0424	0.00263	-16.12	PY61	PY44	MG	EHL	-0.0007	0.002338	-0.32
PY34	PY05	MG	EHL	-0.0316	0.003089	-10.225	PY61	PY45	MG	EHL	-0.0003	0.002851	-0.088
PY34	PY06	MG	EHL	-0.0299	0.003265	-9.146	PY61	PY46	MG	EHL	0.0049	0.002789	1.744
PY34	PY10	MG	EHL	-0.0396	0.002796	-14.17	PY61	PY47	MG	EHL	-0.0014	0.002425	-0.559
PY34	PY17	MG	EHL	-0.0547	0.002333	-23.449	PY61	PY48	MG	EHL	-0.001	0.002591	-0.401
PY34	PY22	MG	EHL	-0.0421	0.002579	-16.307	PY61	PY49	MG	EHL	0.0035	0.002841	1.241

PY34	PY23	MG	EHL	-0.0408	0.002511	-16.249	PY61	PY50	MG	EHL	-0.0013	0.002326	-0.557
PY34	PY24	MG	EHL	-0.0343	0.002561	-13.387	PY61	PY51	MG	EHL	-0.0033	0.002675	-1.243
PY34	PY25	MG	EHL	-0.0378	0.002589	-14.599	PY61	PY52	MG	EHL	0.0033	0.002695	1.226
PY34	PY26	MG	EHL	-0.0368	0.002564	-14.338	PY61	PY53	MG	EHL	0.0038	0.002736	1.394
PY34	PY27	MG	EHL	-0.0261	0.002748	-9.495	PY61	PY54	MG	EHL	0.0032	0.0024	1.343
PY34	PY28	MG	EHL	-0.0357	0.002903	-12.282	PY61	PY55	MG	EHL	0.0027	0.002471	1.112
PY34	PY29	MG	EHL	-0.0358	0.002549	-14.06	PY61	PY56	MG	EHL	0	0.002424	0.013
PY34	PY30	MG	EHL	-0.0398	0.002337	-17.05	PY61	PY57	MG	EHL	0.0016	0.002362	0.657
PY34	PY31	MG	EHL	-0.0343	0.002639	-12.984	PY61	PY58	MG	EHL	0.0019	0.002397	0.803
PY34	PY32	MG	EHL	-0.0335	0.002958	-11.311	PY61	PY59	MG	EHL	-0.0014	0.002493	-0.546
PY34	PY33	MG	EHL	-0.0379	0.002414	-15.684	PY61	PY60	MG	EHL	0.0008	0.002443	0.334
PY34	PY36	MG	EHL	-0.0367	0.002805	-13.098	PY61	PY62	MG	EHL	0.0032	0.002789	1.148
PY34	PY37	MG	EHL	-0.0333	0.002891	-11.508	PY61	PY63	MG	EHL	-0.0015	0.002464	-0.589
PY34	PY39	MG	EHL	-0.0541	0.002361	-22.93	PY61	PY67	MG	EHL	-0.001	0.002831	-0.341
PY34	PY40	MG	EHL	-0.0345	0.002816	-12.24	PY61	PY68	MG	EHL	-0.004	0.002365	-1.701
PY34	PY42	MG	EHL	-0.0367	0.002657	-13.817	PY61	PY69	MG	EHL	-0.0007	0.002828	-0.233
PY34	PY43	MG	EHL	-0.0363	0.002627	-13.825	PY61	PY70	MG	EHL	0.0544	0.005986	9.096
PY34	PY44	MG	EHL	-0.0373	0.002488	-14.998	PY61	PY71	MG	EHL	-0.0082	0.002721	-3.012
PY34	PY45	MG	EHL	-0.0364	0.002735	-13.325	PY62	PY02	MG	EHL	-0.0081	0.002913	-2.783
PY34	PY46	MG	EHL	-0.0321	0.002614	-12.298	PY62	PY05	MG	EHL	0.0013	0.003357	0.379
PY34	PY47	MG	EHL	-0.0373	0.002501	-14.923	PY62	PY06	MG	EHL	0.0032	0.003624	0.876
PY34	PY48	MG	EHL	-0.0373	0.00261	-14.302	PY62	PY10	MG	EHL	-0.0068	0.003028	-2.255
PY34	PY49	MG	EHL	-0.0334	0.002747	-12.145	PY62	PY17	MG	EHL	-0.0226	0.002695	-8.404
PY34	PY50	MG	EHL	-0.0376	0.002374	-15.859	PY62	PY22	MG	EHL	-0.009	0.002981	-3.016
PY34	PY51	MG	EHL	-0.0393	0.00259	-15.173	PY62	PY23	MG	EHL	-0.0074	0.003021	-2.445

PY34	PY52	MG	EHL	-0.0333	0.002659	-12.511	PY62	PY24	MG	EHL	-0.001	0.00294	-0.346
PY34	PY53	MG	EHL	-0.0336	0.00267	-12.585	PY62	PY25	MG	EHL	-0.0052	0.003007	-1.722
PY34	PY54	MG	EHL	-0.0338	0.002633	-12.829	PY62	PY26	MG	EHL	-0.0037	0.00302	-1.226
PY34	PY55	MG	EHL	-0.0345	0.002512	-13.719	PY62	PY27	MG	EHL	0.0075	0.003196	2.356
PY34	PY56	MG	EHL	-0.0361	0.002439	-14.798	PY62	PY28	MG	EHL	-0.0026	0.003377	-0.774
PY34	PY57	MG	EHL	-0.0346	0.002468	-14.012	PY62	PY29	MG	EHL	-0.0027	0.002836	-0.945
PY34	PY58	MG	EHL	-0.0348	0.002582	-13.46	PY62	PY30	MG	EHL	-0.0072	0.002806	-2.579
PY34	PY59	MG	EHL	-0.0377	0.002561	-14.706	PY62	PY31	MG	EHL	-0.0009	0.003162	-0.291
PY34	PY60	MG	EHL	-0.0358	0.002536	-14.119	PY62	PY32	MG	EHL	0	0.003418	0.003
PY34	PY61	MG	EHL	-0.0359	0.002647	-13.572	PY62	PY33	MG	EHL	-0.0046	0.002925	-1.565
PY34	PY62	MG	EHL	-0.0332	0.00293	-11.339	PY62	PY34	MG	EHL	0.0332	0.00293	11.339
PY34	PY63	MG	EHL	-0.0378	0.002481	-15.225	PY62	PY36	MG	EHL	-0.0041	0.003142	-1.29
PY34	PY67	MG	EHL	-0.0375	0.002852	-13.165	PY62	PY37	MG	EHL	-0.0002	0.003322	-0.059
PY34	PY68	MG	EHL	-0.0404	0.002416	-16.719	PY62	PY39	MG	EHL	-0.0225	0.00272	-8.268
PY34	PY69	MG	EHL	-0.0369	0.00286	-12.912	PY62	PY40	MG	EHL	-0.0017	0.003122	-0.548
PY34	PY70	MG	EHL	0.017	0.005717	2.968	PY62	PY42	MG	EHL	-0.0044	0.002975	-1.495
PY34	PY71	MG	EHL	-0.0538	0.002754	-19.515	PY62	PY43	MG	EHL	-0.0029	0.003018	-0.964
PY36	PY02	MG	EHL	-0.0045	0.00295	-1.538	PY62	PY44	MG	EHL	-0.0043	0.002808	-1.522
PY36	PY05	MG	EHL	0.0048	0.003393	1.416	PY62	PY45	MG	EHL	-0.0038	0.003266	-1.171
PY36	PY06	MG	EHL	0.007	0.003541	1.966	PY62	PY46	MG	EHL	0.0008	0.003059	0.265
PY36	PY10	MG	EHL	-0.0032	0.00309	-1.024	PY62	PY47	MG	EHL	-0.005	0.002883	-1.744
PY36	PY17	MG	EHL	-0.0198	0.002586	-7.671	PY62	PY48	MG	EHL	-0.0046	0.003051	-1.507
PY36	PY22	MG	EHL	-0.0053	0.002866	-1.844	PY62	PY49	MG	EHL	0	0.003213	-0.009
PY36	PY23	MG	EHL	-0.0037	0.002825	-1.325	PY62	PY50	MG	EHL	-0.0049	0.002704	-1.813
PY36	PY24	MG	EHL	0.0023	0.002949	0.764	PY62	PY51	MG	EHL	-0.0064	0.00305	-2.089

PY36	PY25	MG	EHL	-0.0013	0.002804	-0.452	PY62	PY52	MG	EHL	0.0001	0.002666	0.044
PY36	PY26	MG	EHL	-0.0001	0.00278	-0.04	PY62	PY53	MG	EHL	-0.0003	0.003142	-0.097
PY36	PY27	MG	EHL	0.0114	0.003062	3.718	PY62	PY54	MG	EHL	-0.0009	0.003049	-0.279
PY36	PY28	MG	EHL	0.001	0.003223	0.322	PY62	PY55	MG	EHL	-0.0016	0.00292	-0.543
PY36	PY29	MG	EHL	0.0012	0.002718	0.424	PY62	PY56	MG	EHL	-0.0036	0.003022	-1.175
PY36	PY30	MG	EHL	-0.0033	0.002678	-1.246	PY62	PY57	MG	EHL	-0.002	0.002932	-0.665
PY36	PY31	MG	EHL	0.0028	0.002952	0.946	PY62	PY58	MG	EHL	-0.0017	0.002949	-0.59
PY36	PY32	MG	EHL	0.0039	0.00331	1.167	PY62	PY59	MG	EHL	-0.0052	0.002916	-1.79
PY36	PY33	MG	EHL	-0.0009	0.002691	-0.331	PY62	PY60	MG	EHL	-0.0033	0.002992	-1.088
PY36	PY34	MG	EHL	0.0367	0.002805	13.098	PY62	PY61	MG	EHL	-0.0032	0.002789	-1.148
PY36	PY37	MG	EHL	0.004	0.003256	1.233	PY62	PY63	MG	EHL	-0.0047	0.002781	-1.704
PY36	PY39	MG	EHL	-0.0191	0.002568	-7.43	PY62	PY67	MG	EHL	-0.0045	0.003089	-1.456
PY36	PY40	MG	EHL	0.0021	0.003203	0.669	PY62	PY68	MG	EHL	-0.0074	0.002933	-2.517
PY36	PY42	MG	EHL	-0.0002	0.002886	-0.083	PY62	PY69	MG	EHL	-0.0037	0.003217	-1.155
PY36	PY43	MG	EHL	0.0007	0.002867	0.256	PY62	PY70	MG	EHL	0.0513	0.00612	8.375
PY36	PY44	MG	EHL	-0.0004	0.002642	-0.16	PY62	PY71	MG	EHL	-0.0117	0.003141	-3.739
PY36	PY45	MG	EHL	0.0002	0.00311	0.059	PY63	PY02	MG	EHL	-0.0036	0.002547	-1.405
PY36	PY46	MG	EHL	0.0048	0.002922	1.629	PY63	PY05	MG	EHL	0.0059	0.002959	1.988
PY36	PY47	MG	EHL	-0.0007	0.002862	-0.256	PY63	PY06	MG	EHL	0.0078	0.003264	2.375
PY36	PY48	MG	EHL	-0.0006	0.002869	-0.207	PY63	PY10	MG	EHL	-0.0021	0.002648	-0.808
PY36	PY49	MG	EHL	0.0039	0.003112	1.258	PY63	PY17	MG	EHL	-0.0185	0.002268	-8.167
PY36	PY50	MG	EHL	-0.0008	0.002657	-0.294	PY63	PY22	MG	EHL	-0.0041	0.002626	-1.565
PY36	PY51	MG	EHL	-0.0028	0.002934	-0.943	PY63	PY23	MG	EHL	-0.0023	0.002495	-0.934
PY36	PY52	MG	EHL	0.004	0.003049	1.317	PY63	PY24	MG	EHL	0.0036	0.002617	1.382
PY36	PY53	MG	EHL	0.0037	0.003026	1.209	PY63	PY25	MG	EHL	-0.0003	0.002627	-0.132

PY36	PY54	MG	EHL	0.0031	0.002843	1.104	PY63	PY26	MG	EHL	0.0012	0.002401	0.503
PY36	PY55	MG	EHL	0.0029	0.00278	1.043	PY63	PY27	MG	EHL	0.0125	0.002726	4.59
PY36	PY56	MG	EHL	0.0007	0.002871	0.241	PY63	PY28	MG	EHL	0.0023	0.003091	0.729
PY36	PY57	MG	EHL	0.0022	0.002857	0.774	PY63	PY29	MG	EHL	0.0025	0.002523	0.976
PY36	PY58	MG	EHL	0.0024	0.002851	0.836	PY63	PY30	MG	EHL	-0.0027	0.002468	-1.074
PY36	PY59	MG	EHL	-0.0008	0.002757	-0.301	PY63	PY31	MG	EHL	0.0037	0.002715	1.354
PY36	PY60	MG	EHL	0.001	0.002836	0.361	PY63	PY32	MG	EHL	0.005	0.003055	1.648
PY36	PY61	MG	EHL	0.0006	0.00285	0.219	PY63	PY33	MG	EHL	0.0002	0.00244	0.085
PY36	PY62	MG	EHL	0.0041	0.003142	1.29	PY63	PY34	MG	EHL	0.0378	0.002481	15.225
PY36	PY63	MG	EHL	-0.0015	0.002799	-0.536	PY63	PY36	MG	EHL	0.0015	0.002799	0.536
PY36	PY67	MG	EHL	-0.0005	0.003101	-0.173	PY63	PY37	MG	EHL	0.0046	0.002924	1.568
PY36	PY68	MG	EHL	-0.0036	0.002797	-1.303	PY63	PY39	MG	EHL	-0.0177	0.002293	-7.704
PY36	PY69	MG	EHL	0	0.003196	0.009	PY63	PY40	MG	EHL	0.0036	0.002694	1.341
PY36	PY70	MG	EHL	0.055	0.006119	8.986	PY63	PY42	MG	EHL	0.0004	0.002465	0.163
PY36	PY71	MG	EHL	-0.0081	0.002933	-2.754	PY63	PY43	MG	EHL	0.002	0.002653	0.751
PY37	PY02	MG	EHL	-0.008	0.002812	-2.842	PY63	PY44	MG	EHL	0.0006	0.002301	0.275
PY37	PY05	MG	EHL	0.001	0.003369	0.304	PY63	PY45	MG	EHL	0.0013	0.002739	0.489
PY37	PY06	MG	EHL	0.0031	0.003597	0.868	PY63	PY46	MG	EHL	0.0059	0.002583	2.288
PY37	PY10	MG	EHL	-0.0068	0.003043	-2.249	PY63	PY47	MG	EHL	0.0002	0.002526	0.077
PY37	PY17	MG	EHL	-0.0233	0.002655	-8.761	PY63	PY48	MG	EHL	0.0003	0.002614	0.122
PY37	PY22	MG	EHL	-0.009	0.003097	-2.907	PY63	PY49	MG	EHL	0.0053	0.002582	2.037
PY37	PY23	MG	EHL	-0.0075	0.003102	-2.404	PY63	PY50	MG	EHL	0.0003	0.002328	0.115
PY37	PY24	MG	EHL	-0.0014	0.003052	-0.467	PY63	PY51	MG	EHL	-0.0019	0.002454	-0.79
PY37	PY25	MG	EHL	-0.0051	0.002959	-1.71	PY63	PY52	MG	EHL	0.0052	0.002664	1.963
PY37	PY26	MG	EHL	-0.0038	0.002939	-1.278	PY63	PY53	MG	EHL	0.0042	0.002678	1.553

PY37	PY27	MG	EHL	0.0079	0.003185	2.494	PY63	PY54	MG	EHL	0.0036	0.002642	1.354
PY37	PY28	MG	EHL	-0.0028	0.003384	-0.838	PY63	PY55	MG	EHL	0.004	0.002387	1.662
PY37	PY29	MG	EHL	-0.0028	0.003015	-0.915	PY63	PY56	MG	EHL	0.0016	0.002439	0.636
PY37	PY30	MG	EHL	-0.0072	0.002881	-2.505	PY63	PY57	MG	EHL	0.003	0.002354	1.281
PY37	PY31	MG	EHL	-0.001	0.00319	-0.303	PY63	PY58	MG	EHL	0.0033	0.002439	1.341
PY37	PY32	MG	EHL	0	0.003427	-0.012	PY63	PY59	MG	EHL	0.0003	0.002397	0.114
PY37	PY33	MG	EHL	-0.0047	0.003024	-1.565	PY63	PY60	MG	EHL	0.0023	0.002415	0.969
PY37	PY34	MG	EHL	0.0333	0.002891	11.508	PY63	PY61	MG	EHL	0.0015	0.002464	0.589
PY37	PY36	MG	EHL	-0.004	0.003256	-1.233	PY63	PY62	MG	EHL	0.0047	0.002781	1.704
PY37	PY39	MG	EHL	-0.0227	0.002609	-8.697	PY63	PY67	MG	EHL	0.0007	0.002762	0.242
PY37	PY40	MG	EHL	-0.0013	0.003446	-0.378	PY63	PY68	MG	EHL	-0.0024	0.002626	-0.914
PY37	PY42	MG	EHL	-0.0048	0.00302	-1.58	PY63	PY69	MG	EHL	0.0013	0.002844	0.458
PY37	PY43	MG	EHL	-0.003	0.003043	-0.989	PY63	PY70	MG	EHL	0.0556	0.005885	9.444
PY37	PY44	MG	EHL	-0.0042	0.002819	-1.5	PY63	PY71	MG	EHL	-0.0069	0.002685	-2.578
PY37	PY45	MG	EHL	-0.0036	0.003232	-1.112	PY67	PY02	MG	EHL	-0.004	0.002739	-1.475
PY37	PY46	MG	EHL	0.0011	0.003189	0.349	PY67	PY05	MG	EHL	0.0052	0.003244	1.617
PY37	PY47	MG	EHL	-0.0044	0.003204	-1.377	PY67	PY06	MG	EHL	0.0079	0.003379	2.324
PY37	PY48	MG	EHL	-0.0048	0.003081	-1.555	PY67	PY10	MG	EHL	-0.0027	0.002858	-0.929
PY37	PY49	MG	EHL	0.0001	0.003293	0.039	PY67	PY17	MG	EHL	-0.0193	0.002482	-7.783
PY37	PY50	MG	EHL	-0.0048	0.002696	-1.794	PY67	PY22	MG	EHL	-0.005	0.002839	-1.762
PY37	PY51	MG	EHL	-0.0065	0.003087	-2.109	PY67	PY23	MG	EHL	-0.0034	0.002541	-1.329
PY37	PY52	MG	EHL	0.001	0.003167	0.307	PY67	PY24	MG	EHL	0.0028	0.002935	0.953
PY37	PY53	MG	EHL	-0.0007	0.003091	-0.232	PY67	PY25	MG	EHL	-0.0007	0.002723	-0.271
PY37	PY54	MG	EHL	-0.0004	0.003037	-0.118	PY67	PY26	MG	EHL	0.0007	0.002725	0.245
PY37	PY55	MG	EHL	-0.0016	0.002837	-0.58	PY67	PY27	MG	EHL	0.012	0.00302	3.971

PY37	PY56	MG	EHL	-0.0034	0.003053	-1.112	PY67	PY28	MG	EHL	0.0016	0.003155	0.496
PY37	PY57	MG	EHL	-0.0019	0.002808	-0.67	PY67	PY29	MG	EHL	0.0017	0.002748	0.609
PY37	PY58	MG	EHL	-0.0016	0.002897	-0.536	PY67	PY30	MG	EHL	-0.0028	0.002636	-1.051
PY37	PY59	MG	EHL	-0.0053	0.002892	-1.831	PY67	PY31	MG	EHL	0.0033	0.002888	1.155
PY37	PY60	MG	EHL	-0.0031	0.002861	-1.085	PY67	PY32	MG	EHL	0.0045	0.003339	1.342
PY37	PY61	MG	EHL	-0.0037	0.002887	-1.271	PY67	PY33	MG	EHL	-0.0004	0.002668	-0.14
PY37	PY62	MG	EHL	0.0002	0.003322	0.059	PY67	PY34	MG	EHL	0.0375	0.002852	13.165
PY37	PY63	MG	EHL	-0.0046	0.002924	-1.568	PY67	PY36	MG	EHL	0.0005	0.003101	0.173
PY37	PY67	MG	EHL	-0.0044	0.003081	-1.439	PY67	PY37	MG	EHL	0.0044	0.003081	1.439
PY37	PY68	MG	EHL	-0.0073	0.002887	-2.532	PY67	PY39	MG	EHL	-0.0186	0.002522	-7.395
PY37	PY69	MG	EHL	-0.0035	0.003175	-1.092	PY67	PY40	MG	EHL	0.0032	0.002688	1.187
PY37	PY70	MG	EHL	0.0519	0.006316	8.218	PY67	PY42	MG	EHL	0.0002	0.002852	0.059
PY37	PY71	MG	EHL	-0.0117	0.002853	-4.101	PY67	PY43	MG	EHL	0.0013	0.00277	0.458
PY39	PY02	MG	EHL	0.0148	0.002376	6.23	PY67	PY44	MG	EHL	0.0001	0.002667	0.032
PY39	PY05	MG	EHL	0.0234	0.002741	8.554	PY67	PY45	MG	EHL	0.0009	0.00284	0.309
PY39	PY06	MG	EHL	0.0256	0.003027	8.461	PY67	PY46	MG	EHL	0.0055	0.00287	1.918
PY39	PY10	MG	EHL	0.0163	0.002453	6.63	PY67	PY47	MG	EHL	0	0.002775	-0.013
PY39	PY17	MG	EHL	-0.0006	0.002015	-0.29	PY67	PY48	MG	EHL	-0.0001	0.002811	-0.029
PY39	PY22	MG	EHL	0.0144	0.002416	5.941	PY67	PY49	MG	EHL	0.0044	0.003071	1.449
PY39	PY23	MG	EHL	0.0157	0.002355	6.654	PY67	PY50	MG	EHL	-0.0004	0.002442	-0.176
PY39	PY24	MG	EHL	0.0214	0.002293	9.33	PY67	PY51	MG	EHL	-0.0024	0.002886	-0.841
PY39	PY25	MG	EHL	0.0179	0.002273	7.864	PY67	PY52	MG	EHL	0.0046	0.002899	1.579
PY39	PY26	MG	EHL	0.019	0.002243	8.455	PY67	PY53	MG	EHL	0.0039	0.002999	1.286
PY39	PY27	MG	EHL	0.0292	0.002498	11.687	PY67	PY54	MG	EHL	0.0039	0.002775	1.393
PY39	PY28	MG	EHL	0.0203	0.002786	7.289	PY67	PY55	MG	EHL	0.0035	0.002696	1.286

PY39	PY29	MG	EHL	0.0201	0.002106	9.543	PY67	PY56	MG	EHL	0.0012	0.002649	0.446
PY39	PY30	MG	EHL	0.0161	0.002123	7.586	PY67	PY57	MG	EHL	0.0025	0.00276	0.917
PY39	PY31	MG	EHL	0.0218	0.002343	9.31	PY67	PY58	MG	EHL	0.0029	0.002748	1.06
PY39	PY32	MG	EHL	0.0226	0.002786	8.093	PY67	PY59	MG	EHL	-0.0002	0.002764	-0.081
PY39	PY33	MG	EHL	0.0183	0.002166	8.455	PY67	PY60	MG	EHL	0.0016	0.002693	0.609
PY39	PY34	MG	EHL	0.0541	0.002361	22.93	PY67	PY61	MG	EHL	0.001	0.002831	0.341
PY39	PY36	MG	EHL	0.0191	0.002568	7.43	PY67	PY62	MG	EHL	0.0045	0.003089	1.456
PY39	PY37	MG	EHL	0.0227	0.002609	8.697	PY67	PY63	MG	EHL	-0.0007	0.002762	-0.242
PY39	PY40	MG	EHL	0.0208	0.002609	7.974	PY67	PY68	MG	EHL	-0.0039	0.002967	-1.327
PY39	PY42	MG	EHL	0.0192	0.002387	8.039	PY67	PY69	MG	EHL	0.0008	0.002778	0.296
PY39	PY43	MG	EHL	0.0198	0.002362	8.375	PY67	PY70	MG	EHL	0.0554	0.005951	9.312
PY39	PY44	MG	EHL	0.0187	0.00211	8.876	PY67	PY71	MG	EHL	-0.0075	0.00282	-2.646
PY39	PY45	MG	EHL	0.0193	0.002478	7.781	PY68	PY02	MG	EHL	-0.001	0.002412	-0.428
PY39	PY46	MG	EHL	0.0237	0.002331	10.173	PY68	PY05	MG	EHL	0.0083	0.002964	2.8
PY39	PY47	MG	EHL	0.0183	0.002395	7.643	PY68	PY06	MG	EHL	0.0107	0.003152	3.399
PY39	PY48	MG	EHL	0.0186	0.002383	7.789	PY68	PY10	MG	EHL	0.0004	0.00266	0.147
PY39	PY49	MG	EHL	0.0226	0.002485	9.091	PY68	PY17	MG	EHL	-0.0162	0.002214	-7.312
PY39	PY50	MG	EHL	0.0183	0.002131	8.608	PY68	PY22	MG	EHL	-0.0019	0.002559	-0.733
PY39	PY51	MG	EHL	0.0164	0.002346	7.001	PY68	PY23	MG	EHL	-0.0002	0.002509	-0.096
PY39	PY52	MG	EHL	0.0226	0.002429	9.314	PY68	PY24	MG	EHL	0.0058	0.002597	2.237
PY39	PY53	MG	EHL	0.0225	0.002293	9.823	PY68	PY25	MG	EHL	0.0023	0.002441	0.945
PY39	PY54	MG	EHL	0.0219	0.002389	9.174	PY68	PY26	MG	EHL	0.0036	0.002428	1.477
PY39	PY55	MG	EHL	0.0215	0.002299	9.345	PY68	PY27	MG	EHL	0.015	0.002744	5.479
PY39	PY56	MG	EHL	0.0196	0.002238	8.756	PY68	PY28	MG	EHL	0.0046	0.003001	1.549
PY39	PY57	MG	EHL	0.0207	0.002152	9.601	PY68	PY29	MG	EHL	0.0048	0.002467	1.934

PY39	PY58	MG	EHL	0.0212	0.00228	9.299	PY68	PY30	MG	EHL	0.0003	0.002301	0.13
PY39	PY59	MG	EHL	0.0181	0.002241	8.07	PY68	PY31	MG	EHL	0.0065	0.002687	2.413
PY39	PY60	MG	EHL	0.02	0.002272	8.782	PY68	PY32	MG	EHL	0.0076	0.003068	2.475
PY39	PY61	MG	EHL	0.0198	0.002367	8.364	PY68	PY33	MG	EHL	0.0027	0.002506	1.084
PY39	PY62	MG	EHL	0.0225	0.00272	8.268	PY68	PY34	MG	EHL	0.0404	0.002416	16.719
PY39	PY63	MG	EHL	0.0177	0.002293	7.704	PY68	PY36	MG	EHL	0.0036	0.002797	1.303
PY39	PY67	MG	EHL	0.0186	0.002522	7.395	PY68	PY37	MG	EHL	0.0073	0.002887	2.532
PY39	PY68	MG	EHL	0.0158	0.002223	7.12	PY68	PY39	MG	EHL	-0.0158	0.002223	-7.12
PY39	PY69	MG	EHL	0.0191	0.002649	7.212	PY68	PY40	MG	EHL	0.0058	0.00282	2.059
PY39	PY70	MG	EHL	0.0711	0.00549	12.955	PY68	PY42	MG	EHL	0.0032	0.002455	1.314
PY39	PY71	MG	EHL	0.0118	0.0022	5.366	PY68	PY43	MG	EHL	0.0043	0.002509	1.731
PY40	PY02	MG	EHL	-0.0067	0.002831	-2.361	PY68	PY44	MG	EHL	0.0032	0.002378	1.342
PY40	PY05	MG	EHL	0.0026	0.003345	0.776	PY68	PY45	MG	EHL	0.0039	0.002692	1.436
PY40	PY06	MG	EHL	0.005	0.00362	1.388	PY68	PY46	MG	EHL	0.0087	0.002574	3.37
PY40	PY10	MG	EHL	-0.0053	0.003169	-1.678	PY68	PY47	MG	EHL	0.003	0.002349	1.292
PY40	PY17	MG	EHL	-0.0207	0.002593	-7.98	PY68	PY48	MG	EHL	0.003	0.002556	1.187
PY40	PY22	MG	EHL	-0.0078	0.002965	-2.64	PY68	PY49	MG	EHL	0.0075	0.002846	2.631
PY40	PY23	MG	EHL	-0.0061	0.002975	-2.066	PY68	PY50	MG	EHL	0.0028	0.002241	1.245
PY40	PY24	MG	EHL	0.0007	0.002924	0.241	PY68	PY51	MG	EHL	0.0006	0.002568	0.247
PY40	PY25	MG	EHL	-0.0036	0.002851	-1.266	PY68	PY52	MG	EHL	0.0072	0.002705	2.674
PY40	PY26	MG	EHL	-0.0025	0.00279	-0.891	PY68	PY53	MG	EHL	0.0068	0.002522	2.681
PY40	PY27	MG	EHL	0.0099	0.003029	3.285	PY68	PY54	MG	EHL	0.0067	0.002436	2.766
PY40	PY28	MG	EHL	-0.002	0.003378	-0.6	PY68	PY55	MG	EHL	0.0061	0.002343	2.599
PY40	PY29	MG	EHL	-0.0013	0.002889	-0.441	PY68	PY56	MG	EHL	0.0044	0.002265	1.927
PY40	PY30	MG	EHL	-0.0055	0.002782	-1.984	PY68	PY57	MG	EHL	0.0055	0.002234	2.448

PY40	PY31	MG	EHL	0.0008	0.003081	0.263	PY68	PY58	MG	EHL	0.006	0.002301	2.595
PY40	PY32	MG	EHL	0.0013	0.003308	0.379	PY68	PY59	MG	EHL	0.003	0.002384	1.261
PY40	PY33	MG	EHL	-0.0028	0.002797	-1	PY68	PY60	MG	EHL	0.0047	0.002341	2.027
PY40	PY34	MG	EHL	0.0345	0.002816	12.24	PY68	PY61	MG	EHL	0.004	0.002365	1.701
PY40	PY36	MG	EHL	-0.0021	0.003203	-0.669	PY68	PY62	MG	EHL	0.0074	0.002933	2.517
PY40	PY37	MG	EHL	0.0013	0.003446	0.378	PY68	PY63	MG	EHL	0.0024	0.002626	0.914
PY40	PY39	MG	EHL	-0.0208	0.002609	-7.974	PY68	PY67	MG	EHL	0.0039	0.002967	1.327
PY40	PY42	MG	EHL	-0.0029	0.002884	-0.99	PY68	PY69	MG	EHL	0.0045	0.002947	1.512
PY40	PY43	MG	EHL	-0.0015	0.002904	-0.52	PY68	PY70	MG	EHL	0.0583	0.005766	10.102
PY40	PY44	MG	EHL	-0.0028	0.002778	-1.009	PY68	PY71	MG	EHL	-0.0045	0.002472	-1.839
PY40	PY45	MG	EHL	-0.002	0.003181	-0.635	PY69	PY02	MG	EHL	-0.0046	0.002795	-1.637
PY40	PY46	MG	EHL	0.0029	0.003057	0.947	PY69	PY05	MG	EHL	0.0048	0.003261	1.484
PY40	PY47	MG	EHL	-0.0027	0.00284	-0.949	PY69	PY06	MG	EHL	0.0069	0.003442	2.01
PY40	PY48	MG	EHL	-0.0032	0.00289	-1.099	PY69	PY10	MG	EHL	-0.0032	0.003013	-1.078
PY40	PY49	MG	EHL	0.0014	0.003077	0.467	PY69	PY17	MG	EHL	-0.0198	0.002525	-7.834
PY40	PY50	MG	EHL	-0.0032	0.002743	-1.149	PY69	PY22	MG	EHL	-0.0057	0.002972	-1.925
PY40	PY51	MG	EHL	-0.0041	0.002909	-1.417	PY69	PY23	MG	EHL	-0.004	0.002891	-1.397
PY40	PY52	MG	EHL	0.0019	0.002931	0.645	PY69	PY24	MG	EHL	0.0026	0.002929	0.884
PY40	PY53	MG	EHL	0.0015	0.003151	0.48	PY69	PY25	MG	EHL	-0.0014	0.002849	-0.477
PY40	PY54	MG	EHL	0.0015	0.002988	0.497	PY69	PY26	MG	EHL	0	0.002949	0.013
PY40	PY55	MG	EHL	0.0005	0.002777	0.174	PY69	PY27	MG	EHL	0.0114	0.003111	3.668
PY40	PY56	MG	EHL	-0.0016	0.002815	-0.561	PY69	PY28	MG	EHL	0.0009	0.003334	0.276
PY40	PY57	MG	EHL	0.0003	0.002917	0.1	PY69	PY29	MG	EHL	0.001	0.002804	0.351
PY40	PY58	MG	EHL	0.0003	0.00297	0.091	PY69	PY30	MG	EHL	-0.0035	0.002689	-1.296
PY40	PY59	MG	EHL	-0.0035	0.002738	-1.267	PY69	PY31	MG	EHL	0.0027	0.003064	0.87

PY40	PY60	MG	EHL	-0.0013	0.002885	-0.456	PY69	PY32	MG	EHL	0.0036	0.003322	1.093
PY40	PY61	MG	EHL	-0.0013	0.002918	-0.456	PY69	PY33	MG	EHL	-0.001	0.002833	-0.361
PY40	PY62	MG	EHL	0.0017	0.003122	0.548	PY69	PY34	MG	EHL	0.0369	0.00286	12.912
PY40	PY63	MG	EHL	-0.0036	0.002694	-1.341	PY69	PY36	MG	EHL	0	0.003196	-0.009
PY40	PY67	MG	EHL	-0.0032	0.002688	-1.187	PY69	PY37	MG	EHL	0.0035	0.003175	1.092
PY40	PY68	MG	EHL	-0.0058	0.00282	-2.059	PY69	PY39	MG	EHL	-0.0191	0.002649	-7.212
PY40	PY69	MG	EHL	-0.0024	0.002553	-0.926	PY69	PY40	MG	EHL	0.0024	0.002553	0.926
PY40	PY70	MG	EHL	0.053	0.006056	8.76	PY69	PY42	MG	EHL	-0.0005	0.002905	-0.171
PY40	PY71	MG	EHL	-0.0102	0.002907	-3.503	PY69	PY43	MG	EHL	0.0006	0.002898	0.196
PY42	PY02	MG	EHL	-0.0041	0.002586	-1.574	PY69	PY44	MG	EHL	-0.0006	0.002649	-0.227
PY42	PY05	MG	EHL	0.0048	0.003164	1.502	PY69	PY45	MG	EHL	0.0003	0.003134	0.088
PY42	PY06	MG	EHL	0.0072	0.003364	2.133	PY69	PY46	MG	EHL	0.005	0.002876	1.753
PY42	PY10	MG	EHL	-0.0024	0.002836	-0.862	PY69	PY47	MG	EHL	-0.0008	0.002763	-0.272
PY42	PY17	MG	EHL	-0.0195	0.002321	-8.402	PY69	PY48	MG	EHL	-0.0007	0.002884	-0.253
PY42	PY22	MG	EHL	-0.0052	0.002727	-1.923	PY69	PY49	MG	EHL	0.0036	0.003111	1.165
PY42	PY23	MG	EHL	-0.0037	0.002665	-1.376	PY69	PY50	MG	EHL	-0.001	0.00271	-0.36
PY42	PY24	MG	EHL	0.003	0.00268	1.105	PY69	PY51	MG	EHL	-0.0028	0.003026	-0.923
PY42	PY25	MG	EHL	-0.0007	0.002715	-0.254	PY69	PY52	MG	EHL	0.0039	0.002859	1.37
PY42	PY26	MG	EHL	0.0008	0.002617	0.314	PY69	PY53	MG	EHL	0.0038	0.002979	1.266
PY42	PY27	MG	EHL	0.0116	0.002803	4.121	PY69	PY54	MG	EHL	0.0031	0.002916	1.052
PY42	PY28	MG	EHL	0.0016	0.003041	0.514	PY69	PY55	MG	EHL	0.0026	0.002755	0.952
PY42	PY29	MG	EHL	0.0016	0.00247	0.667	PY69	PY56	MG	EHL	0.0008	0.002831	0.27
PY42	PY30	MG	EHL	-0.0025	0.002405	-1.053	PY69	PY57	MG	EHL	0.0021	0.002751	0.747
PY42	PY31	MG	EHL	0.0033	0.002853	1.17	PY69	PY58	MG	EHL	0.0023	0.002747	0.829
PY42	PY32	MG	EHL	0.0044	0.00311	1.419	PY69	PY59	MG	EHL	-0.0008	0.002765	-0.289

PY42	PY33	MG	EHL	-0.0006	0.002551	-0.219	PY69	PY60	MG	EHL	0.0009	0.002717	0.316
PY42	PY34	MG	EHL	0.0367	0.002657	13.817	PY69	PY61	MG	EHL	0.0007	0.002828	0.233
PY42	PY36	MG	EHL	0.0002	0.002886	0.083	PY69	PY62	MG	EHL	0.0037	0.003217	1.155
PY42	PY37	MG	EHL	0.0048	0.00302	1.58	PY69	PY63	MG	EHL	-0.0013	0.002844	-0.458
PY42	PY39	MG	EHL	-0.0192	0.002387	-8.039	PY69	PY67	MG	EHL	-0.0008	0.002778	-0.296
PY42	PY40	MG	EHL	0.0029	0.002884	0.99	PY69	PY68	MG	EHL	-0.0045	0.002947	-1.512
PY42	PY43	MG	EHL	0.0011	0.002731	0.406	PY69	PY70	MG	EHL	0.0546	0.005895	9.266
PY42	PY44	MG	EHL	0	0.002391	-0.014	PY69	PY71	MG	EHL	-0.008	0.002897	-2.755
PY42	PY45	MG	EHL	0.0005	0.002951	0.165	PY70	PY02	MG	EHL	-0.0591	0.005756	-10.265
PY42	PY46	MG	EHL	0.006	0.002696	2.212	PY70	PY05	MG	EHL	-0.0585	0.007139	-8.195
PY42	PY47	MG	EHL	-0.0003	0.002573	-0.116	PY70	PY06	MG	EHL	-0.0569	0.007067	-8.047
PY42	PY48	MG	EHL	-0.0004	0.002632	-0.153	PY70	PY10	MG	EHL	-0.0652	0.006545	-9.963
PY42	PY49	MG	EHL	0.0046	0.002872	1.585	PY70	PY17	MG	EHL	-0.0713	0.005603	-12.717
PY42	PY50	MG	EHL	-0.0003	0.002379	-0.143	PY70	PY22	MG	EHL	-0.0599	0.005834	-10.276
PY42	PY51	MG	EHL	-0.0024	0.002681	-0.88	PY70	PY23	MG	EHL	-0.0585	0.005869	-9.974
PY42	PY52	MG	EHL	0.0046	0.002836	1.631	PY70	PY24	MG	EHL	-0.0529	0.00584	-9.064
PY42	PY53	MG	EHL	0.0043	0.002841	1.527	PY70	PY25	MG	EHL	-0.0561	0.00587	-9.565
PY42	PY54	MG	EHL	0.0036	0.002519	1.443	PY70	PY26	MG	EHL	-0.0553	0.006006	-9.209
PY42	PY55	MG	EHL	0.0029	0.00238	1.2	PY70	PY27	MG	EHL	-0.0444	0.006091	-7.291
PY42	PY56	MG	EHL	0.0013	0.002403	0.555	PY70	PY28	MG	EHL	-0.0543	0.006131	-8.859
PY42	PY57	MG	EHL	0.0023	0.002461	0.94	PY70	PY29	MG	EHL	-0.0538	0.005849	-9.203
PY42	PY58	MG	EHL	0.0031	0.00264	1.19	PY70	PY30	MG	EHL	-0.058	0.005842	-9.934
PY42	PY59	MG	EHL	-0.0004	0.002586	-0.138	PY70	PY31	MG	EHL	-0.0521	0.006038	-8.629
PY42	PY60	MG	EHL	0.0011	0.00247	0.457	PY70	PY32	MG	EHL	-0.0514	0.006336	-8.118
PY42	PY61	MG	EHL	0.0011	0.002456	0.434	PY70	PY33	MG	EHL	-0.0556	0.005974	-9.312

PY42	PY62	MG	EHL	0.0044	0.002975	1.495	PY70	PY34	MG	EHL	-0.017	0.005717	-2.968
PY42	PY63	MG	EHL	-0.0004	0.002465	-0.163	PY70	PY36	MG	EHL	-0.055	0.006119	-8.986
PY42	PY67	MG	EHL	-0.0002	0.002852	-0.059	PY70	PY37	MG	EHL	-0.0519	0.006316	-8.218
PY42	PY68	MG	EHL	-0.0032	0.002455	-1.314	PY70	PY39	MG	EHL	-0.0711	0.00549	-12.955
PY42	PY69	MG	EHL	0.0005	0.002905	0.171	PY70	PY40	MG	EHL	-0.053	0.006056	-8.76
PY42	PY70	MG	EHL	0.0553	0.005925	9.34	PY70	PY42	MG	EHL	-0.0553	0.005925	-9.34
PY42	PY71	MG	EHL	-0.0079	0.002707	-2.901	PY70	PY43	MG	EHL	-0.0542	0.005876	-9.233
PY43	PY02	MG	EHL	-0.0053	0.002592	-2.032	PY70	PY44	MG	EHL	-0.0553	0.005821	-9.507
PY43	PY05	MG	EHL	0.0039	0.0031	1.273	PY70	PY45	MG	EHL	-0.0546	0.005772	-9.465
PY43	PY06	MG	EHL	0.0062	0.003412	1.817	PY70	PY46	MG	EHL	-0.0505	0.00596	-8.469
PY43	PY10	MG	EHL	-0.0038	0.002765	-1.386	PY70	PY47	MG	EHL	-0.0557	0.005873	-9.475
PY43	PY17	MG	EHL	-0.0202	0.002236	-9.031	PY70	PY48	MG	EHL	-0.0554	0.005912	-9.363
PY43	PY22	MG	EHL	-0.0061	0.002704	-2.24	PY70	PY49	MG	EHL	-0.0514	0.006059	-8.487
PY43	PY23	MG	EHL	-0.0044	0.002569	-1.715	PY70	PY50	MG	EHL	-0.0562	0.005882	-9.554
PY43	PY24	MG	EHL	0.002	0.002744	0.725	PY70	PY51	MG	EHL	-0.0578	0.005786	-9.991
PY43	PY25	MG	EHL	-0.0021	0.002594	-0.804	PY70	PY52	MG	EHL	-0.051	0.005958	-8.567
PY43	PY26	MG	EHL	-0.0005	0.002513	-0.197	PY70	PY53	MG	EHL	-0.0517	0.005884	-8.785
PY43	PY27	MG	EHL	0.0107	0.002823	3.779	PY70	PY54	MG	EHL	-0.0519	0.005888	-8.819
PY43	PY28	MG	EHL	0.0002	0.003031	0.073	PY70	PY55	MG	EHL	-0.0525	0.00592	-8.863
PY43	PY29	MG	EHL	0.0004	0.002446	0.172	PY70	PY56	MG	EHL	-0.054	0.005881	-9.186
PY43	PY30	MG	EHL	-0.0042	0.002478	-1.686	PY70	PY57	MG	EHL	-0.053	0.005827	-9.089
PY43	PY31	MG	EHL	0.0023	0.002633	0.872	PY70	PY58	MG	EHL	-0.0529	0.005868	-9.024
PY43	PY32	MG	EHL	0.0033	0.003147	1.038	PY70	PY59	MG	EHL	-0.0561	0.005915	-9.48
PY43	PY33	MG	EHL	-0.0017	0.002557	-0.667	PY70	PY60	MG	EHL	-0.0539	0.005876	-9.177
PY43	PY34	MG	EHL	0.0363	0.002627	13.825	PY70	PY61	MG	EHL	-0.0544	0.005986	-9.096

PY43	PY36	MG	EHL	-0.0007	0.002867	-0.256	PY70	PY62	MG	EHL	-0.0513	0.00612	-8.375
PY43	PY37	MG	EHL	0.003	0.003043	0.989	PY70	PY63	MG	EHL	-0.0556	0.005885	-9.444
PY43	PY39	MG	EHL	-0.0198	0.002362	-8.375	PY70	PY67	MG	EHL	-0.0554	0.005951	-9.312
PY43	PY40	MG	EHL	0.0015	0.002904	0.52	PY70	PY68	MG	EHL	-0.0583	0.005766	-10.102
PY43	PY42	MG	EHL	-0.0011	0.002731	-0.406	PY70	PY69	MG	EHL	-0.0546	0.005895	-9.266
PY43	PY44	MG	EHL	-0.0012	0.002433	-0.513	PY70	PY71	MG	EHL	-0.0621	0.005931	-10.475
PY43	PY45	MG	EHL	-0.0007	0.002781	-0.244	PY71	PY02	MG	EHL	0.0035	0.002521	1.395
PY43	PY46	MG	EHL	0.0042	0.002808	1.497	PY71	PY05	MG	EHL	0.0129	0.003081	4.182
PY43	PY47	MG	EHL	-0.0014	0.002585	-0.541	PY71	PY06	MG	EHL	0.0148	0.003364	4.388
PY43	PY48	MG	EHL	-0.0014	0.002756	-0.493	PY71	PY10	MG	EHL	0.0048	0.002899	1.66
PY43	PY49	MG	EHL	0.0032	0.002988	1.075	PY71	PY17	MG	EHL	-0.0124	0.002318	-5.329
PY43	PY50	MG	EHL	-0.0017	0.002351	-0.704	PY71	PY22	MG	EHL	0.003	0.002659	1.13
PY43	PY51	MG	EHL	-0.0036	0.002633	-1.382	PY71	PY23	MG	EHL	0.0043	0.002697	1.597
PY43	PY52	MG	EHL	0.0031	0.002839	1.102	PY71	PY24	MG	EHL	0.0111	0.002669	4.162
PY43	PY53	MG	EHL	0.0028	0.002762	1.002	PY71	PY25	MG	EHL	0.0068	0.002619	2.579
PY43	PY54	MG	EHL	0.0024	0.002656	0.921	PY71	PY26	MG	EHL	0.0081	0.002644	3.076
PY43	PY55	MG	EHL	0.0021	0.002442	0.84	PY71	PY27	MG	EHL	0.0188	0.002839	6.62
PY43	PY56	MG	EHL	0	0.002388	0.018	PY71	PY28	MG	EHL	0.009	0.003105	2.893
PY43	PY57	MG	EHL	0.0012	0.002513	0.472	PY71	PY29	MG	EHL	0.0092	0.002566	3.604
PY43	PY58	MG	EHL	0.0016	0.002513	0.638	PY71	PY30	MG	EHL	0.0049	0.002435	2.002
PY43	PY59	MG	EHL	-0.0019	0.002515	-0.763	PY71	PY31	MG	EHL	0.011	0.002826	3.91
PY43	PY60	MG	EHL	0.0004	0.002468	0.174	PY71	PY32	MG	EHL	0.0118	0.003058	3.874
PY43	PY61	MG	EHL	0	0.002694	-0.011	PY71	PY33	MG	EHL	0.0072	0.002625	2.744
PY43	PY62	MG	EHL	0.0029	0.003018	0.964	PY71	PY34	MG	EHL	0.0538	0.002754	19.515
PY43	PY63	MG	EHL	-0.002	0.002653	-0.751	PY71	PY36	MG	EHL	0.0081	0.002933	2.754

PY43	PY67	MG	EHL	-0.0013	0.00277	-0.458	PY71	PY37	MG	EHL	0.0117	0.002853	4.101
PY43	PY68	MG	EHL	-0.0043	0.002509	-1.731	PY71	PY39	MG	EHL	-0.0118	0.0022	-5.366
PY43	PY69	MG	EHL	-0.0006	0.002898	-0.196	PY71	PY40	MG	EHL	0.0102	0.002907	3.503
PY43	PY70	MG	EHL	0.0542	0.005876	9.233	PY71	PY42	MG	EHL	0.0079	0.002707	2.901
PY43	PY71	MG	EHL	-0.0087	0.002704	-3.235	PY71	PY43	MG	EHL	0.0087	0.002704	3.235
PY44	PY02	MG	EHL	-0.0041	0.002512	-1.643	PY71	PY44	MG	EHL	0.0077	0.002482	3.1
PY44	PY05	MG	EHL	0.0051	0.002826	1.797	PY71	PY45	MG	EHL	0.0083	0.002816	2.931
PY44	PY06	MG	EHL	0.0073	0.003141	2.333	PY71	PY46	MG	EHL	0.0126	0.002668	4.708
PY44	PY10	MG	EHL	-0.0027	0.002561	-1.059	PY71	PY47	MG	EHL	0.0073	0.002611	2.792
PY44	PY17	MG	EHL	-0.0194	0.00219	-8.854	PY71	PY48	MG	EHL	0.0074	0.002821	2.608
PY44	PY22	MG	EHL	-0.0049	0.002575	-1.888	PY71	PY49	MG	EHL	0.0118	0.002818	4.197
PY44	PY23	MG	EHL	-0.0033	0.002615	-1.265	PY71	PY50	MG	EHL	0.0074	0.002513	2.955
PY44	PY24	MG	EHL	0.003	0.0025	1.185	PY71	PY51	MG	EHL	0.0052	0.002711	1.922
PY44	PY25	MG	EHL	-0.0009	0.002529	-0.35	PY71	PY52	MG	EHL	0.0115	0.002803	4.117
PY44	PY26	MG	EHL	0.0006	0.002306	0.241	PY71	PY53	MG	EHL	0.0115	0.00269	4.27
PY44	PY27	MG	EHL	0.0118	0.002587	4.568	PY71	PY54	MG	EHL	0.0109	0.002649	4.099
PY44	PY28	MG	EHL	0.0014	0.002921	0.484	PY71	PY55	MG	EHL	0.0104	0.002565	4.043
PY44	PY29	MG	EHL	0.0016	0.002331	0.694	PY71	PY56	MG	EHL	0.0085	0.002535	3.345
PY44	PY30	MG	EHL	-0.003	0.00221	-1.336	PY71	PY57	MG	EHL	0.0103	0.002544	4.04
PY44	PY31	MG	EHL	0.0035	0.002637	1.346	PY71	PY58	MG	EHL	0.0103	0.002482	4.141
PY44	PY32	MG	EHL	0.0044	0.002884	1.508	PY71	PY59	MG	EHL	0.007	0.002558	2.739
PY44	PY33	MG	EHL	-0.0005	0.002383	-0.204	PY71	PY60	MG	EHL	0.0089	0.002658	3.362
PY44	PY34	MG	EHL	0.0373	0.002488	14.998	PY71	PY61	MG	EHL	0.0082	0.002721	3.012
PY44	PY36	MG	EHL	0.0004	0.002642	0.16	PY71	PY62	MG	EHL	0.0117	0.003141	3.739
PY44	PY37	MG	EHL	0.0042	0.002819	1.5	PY71	PY63	MG	EHL	0.0069	0.002685	2.578

PY44	PY39	MG	EHL	-0.0187	0.00211	-8.876	PY71	PY67	MG	EHL	0.0075	0.00282	2.646
PY44	PY40	MG	EHL	0.0028	0.002778	1.009	PY71	PY68	MG	EHL	0.0045	0.002472	1.839
PY44	PY42	MG	EHL	0	0.002391	0.014	PY71	PY69	MG	EHL	0.008	0.002897	2.755
PY44	PY43	MG	EHL	0.0012	0.002433	0.513	PY71	PY70	MG	EHL	0.0621	0.005931	10.475

Table S18. Results of the apoh analysis. The Admixture index was *NA* for all individuals, consistent with no evidence for recent admixture under the tested pedigree models.

SampleID	InconsistencyIndex	Distance to independent pedigree	Distance to pedigree 1	Distance to pedigree 2	Admixture index
MY01	0.01	0	0.01	0.25	*NA*
MY02	0.01	0	0.01	0.25	*NA*
MY03	0.01	0	0.01	0.25	*NA*
MY04	0.02	0	0.01	0.26	*NA*
MY05	0.01	0	0.01	0.25	*NA*
MY06	0.01	0	0.01	0.26	*NA*
MY08	0.05	0	0.01	0.25	*NA*
MY10	0.02	0	0.01	0.26	*NA*
MY11	0.02	0	0.01	0.25	*NA*
MY16	0.02	0	0.01	0.26	*NA*
MY18	0.02	0	0.01	0.25	*NA*
MY19	0.06	0.01	0.01	0.25	*NA*
MY22	0.04	0	0.01	0.25	*NA*
MY24	0	0.07	0.1	0.18	*NA*
MY26	0.01	0.01	0.03	0.25	*NA*
MY28	0.02	0	0	0.26	*NA*
MY32	0.09	0.01	0.06	0.24	*NA*
MY35	0.04	0	0.01	0.25	*NA*
MY36	0.08	0	0.03	0.25	*NA*
MY37	0.06	0	0.01	0.25	*NA*
MY38	0.03	0	0.01	0.25	*NA*

MY39	0.03	0	0.01	0.25	*NA*
MY41	0.06	0	0.03	0.25	*NA*
MY42	0.05	0	0.01	0.25	*NA*
MY43	0.02	0	0.01	0.25	*NA*
MY44	0.06	0	0.01	0.25	*NA*
MY45	0.02	0	0.01	0.26	*NA*
MY46	0.06	0	0.01	0.25	*NA*
MY47	0.01	0	0.01	0.26	*NA*
MY48	0.07	0	0.02	0.25	*NA*
MY49	0.05	0	0.01	0.25	*NA*
MY50	0.07	0.01	0.02	0.25	*NA*
PY02	0.25	0	0.14	0.2	*NA*
PY05	0.16	0	0.2	0.23	*NA*
PY06	0.26	0	0.21	0.23	*NA*
PY10	0.14	0	0.16	0.21	*NA*
PY17	0.3	0	0.14	0.17	*NA*
PY22	0.07	0	0	0.26	*NA*
PY23	0.04	0	0	0.26	*NA*
PY24	0.02	0	0	0.26	*NA*
PY25	0.01	0	0	0.26	*NA*
PY26	0.01	0	0	0.26	*NA*
PY27	0.01	0	0	0.26	*NA*
PY28	0.01	0	0	0.26	*NA*
PY29	0.02	0	0	0.26	*NA*
PY30	0.02	0	0	0.26	*NA*

PY31	0.04	0	0	0.26	*NA*
PY32	0.01	0	0	0.26	*NA*
PY33	0.02	0	0	0.26	*NA*
PY34	0.2	0	0.19	0.28	*NA*
PY36	0.04	0	0	0.26	*NA*
PY37	0.01	0	0	0.26	*NA*
PY39	0.2	0	0.14	0.17	*NA*
PY40	0.01	0	0	0.26	*NA*
PY42	0.01	0	0	0.26	*NA*
PY43	0.02	0	0	0.26	*NA*
PY44	0.01	0	0	0.26	*NA*
PY45	0.01	0	0	0.26	*NA*
PY46	0.01	0	0	0.26	*NA*
PY47	0.02	0	0	0.26	*NA*
PY48	0.01	0	0	0.26	*NA*
PY49	0.02	0	0	0.26	*NA*
PY50	0.01	0	0	0.26	*NA*
PY51	0.01	0	0	0.26	*NA*
PY52	0.01	0	0	0.26	*NA*
PY53	0.01	0	0	0.26	*NA*
PY54	0.01	0	0	0.26	*NA*
PY55	0.01	0	0	0.26	*NA*
PY56	0.01	0	0	0.26	*NA*
PY57	0.01	0	0	0.26	*NA*
PY58	0.01	0	0	0.26	*NA*

PY59	0.02	0	0	0.26	*NA*
PY60	0.01	0	0	0.26	*NA*
PY61	0.01	0	0	0.26	*NA*
PY62	0.01	0	0	0.26	*NA*
PY63	0.02	0	0	0.26	*NA*
PY67	0.03	0	0	0.26	*NA*
PY68	0.02	0	0	0.26	*NA*
PY69	0.01	0	0	0.26	*NA*
PY70	0.23	0	0.19	0.21	*NA*
PY71	0.27	0	0.11	0.21	*NA*
ZY02	0.01	0	0	0	*NA*
ZY04	0.01	0	0.36	0	*NA*
ZY06	0.01	0	0.26	0.26	*NA*
ZY07	0.01	0	0.26	0.26	*NA*
ZY08	0.01	0	0.26	0.26	*NA*
ZY10	0.01	0	0.26	0.26	*NA*
ZY16	0	0	0.26	0.26	*NA*
ZY17	0	0	0.26	0.26	*NA*
ZY18	0.01	0	0.26	0.26	*NA*
ZY19	0.01	0	0.26	0.26	*NA*
ZY20	0	0	0.26	0.26	*NA*
ZY21	0	0	0.26	0.26	*NA*

Table S19. Correlations among selected bioclimatic variables within the study area.

Species	Bioclimatic variable	bio1	bio12	bio15	bio2
MG	bio1	1	-0.29	-0.09	0.05
	bio12	-0.29	1	0.26	-0.34
	bio15	-0.09	0.26	1	-0.01
	bio2	0.05	-0.34	-0.01	1
**Species**	**Bioclimatic variable**	**bio11**	**bio12**	**bio17**	
PG	bio11	1	0.27	0.31	
	bio12	0.27	1	0.78	
	bio17	0.31	0.78	1	
**Species**	**Bioclimatic variable**	**bio1**	**bio12**	**bio15**	**bio2**
TG	bio1	1	0.47	0.08	-0.02
	bio12	0.47	1	0.05	-0.66
	bio15	0.08	0.05	1	0.16
	bio2	-0.02	-0.66	0.16	1

Note: bio1: Annual Mean Temperature

bio2: Mean Diurnal Range (Mean of monthly (max temp – min temp))

bio11: Mean Temperature of Coldest Quarter

bio12: Annual Precipitation

bio15: Precipitation Seasonality (Coefficient of Variation)

bio17: Precipitation of Driest Quarter

Table S20. ENMeval tuning results for candidate species distribution models.

Species	Model number	fc	rm	tune.args	auc.train	cbi.train	auc.diff.avg	auc.diff.sd	auc.val.avg	auc.val.sd	cbi.val.avg	cbi.val.sd	or.10p.avg	or.10p.sd	or.mtp.avg	or.mtp.sd	AICc	delta.AICc	w.AIC	ncoef
MG	1	LQ	1	fc.LQ_rm.1	0.89	0.91	0.05	0.07	0.83	0.06	0.47	0.19	0.15	0.26	0.05	0.06	1358.89	1.01	0.37	9
MG	2	LQH	1	fc.LQH_rm.1	0.9	0.96	0.05	0.05	0.85	0.03	0.44	0.21	0.15	0.26	0.03	0.04	1357.88	0	0.62	15
MG	3	LQHP	1	fc.LQHP_rm.1	0.91	0.98	0.06	0.05	0.85	0.03	0.48	0.16	0.15	0.26	0.03	0.04	1475.82	117.95	0	33
MG	4	LQ	2	fc.LQ_rm.2	0.86	0.94	0.06	0.08	0.82	0.07	0.54	0.32	0.15	0.26	0.03	0.07	1375.07	17.19	0	7
MG	5	LQH	2	fc.LQH_rm.2	0.89	0.94	0.05	0.07	0.85	0.05	0.54	0.27	0.17	0.29	0.05	0.06	1382.35	24.47	0	17
MG	6	LQHP	2	fc.LQHP_rm.2	0.9	0.89	0.04	0.07	0.85	0.05	0.53	0.25	0.15	0.26	0.05	0.06	1366.25	8.37	0.01	13
MG	7	LQ	3	fc.LQ_rm.3	0.85	0.95	0.06	0.07	0.82	0.07	0.64	0.23	0.15	0.26	0.03	0.07	1383.31	25.43	0	6
MG	8	LQH	3	fc.LQH_rm.3	0.88	0.95	0.06	0.08	0.84	0.07	0.56	0.32	0.15	0.26	0.05	0.06	1378.71	20.83	0	13
MG	9	LQHP	3	fc.LQHP_rm.3	0.89	0.85	0.06	0.07	0.85	0.07	0.59	0.23	0.17	0.29	0.05	0.06	1374.08	16.2	0	12
PG	1	LQ	1	fc.LQ_rm.1	0.99	0.97	0.01	0.01	0.98	0.01	0.73	0.24	0.14	0.17	0.09	0.18	674.49	25.1	0	10
PG	2	LQH	1	fc.LQH_rm.1	1	0.92	0.01	0	0.99	0.01	0.81	0.17	0.21	0.19	0.14	0.12	837.81	188.41	0	32

PG	3	LQHP	1	fc.LQHP_rm.1	1	0.94	0.01	0	0.99	0.01	0.8	0.12	0.25	0.25	0.16	0.13	NA	NA	NA	45
PG	4	LQ	2	fc.LQ_rm.2	0.98	0.97	0.01	0	0.98	0.01	0.65	0.25	0.11	0.17	0.07	0.14	711.49	62.1	0	7
PG	5	LQH	2	fc.LQH_rm.2	1	0.86	0	0	0.99	0.01	0.87	0.09	0.16	0.13	0.09	0.1	660.26	10.86	0	21
PG	6	LQHP	2	fc.LQHP_rm.2	1	0.9	0.01	0	0.99	0.01	0.88	0.08	0.16	0.13	0.11	0.14	664.47	15.08	0	21
PG	7	LQ	3	fc.LQ_rm.3	0.98	0.94	0.01	0	0.98	0.01	0.58	0.26	0.14	0.16	0.05	0.09	714.9	65.5	0	6
PG	8	LQH	3	fc.LQH_rm.3	1	0.87	0	0	0.99	0.01	0.83	0.12	0.14	0.12	0.07	0.09	649.4	0	0.97	15
PG	9	LQHP	3	fc.LQHP_rm.3	1	0.91	0.01	0.01	0.99	0.01	0.82	0.18	0.11	0.14	0.11	0.14	656.96	7.56	0.02	15
TG	1	LQ	1	fc.LQ_rm.1	0.75	0.79	0.13	0.09	0.68	0.12	0.39	0.38	0.22	0.3	0.01	0.03	1670.81	27.95	0	7
TG	2	LQH	1	fc.LQH_rm.1	0.84	0.87	0.11	0.1	0.74	0.09	0.25	0.42	0.2	0.28	0.03	0.03	1681.39	38.54	0	24
TG	3	LQHP	1	fc.LQHP_rm.1	0.84	0.92	0.13	0.14	0.72	0.12	0.31	0.28	0.23	0.26	0.07	0.11	1702.59	59.73	0	28
TG	4	LQ	2	fc.LQ_rm.2	0.74	0.73	0.09	0.08	0.71	0.1	0.46	0.19	0.14	0.13	0.01	0.03	1671.67	28.81	0	6
TG	5	LQH	2	fc.LQH_rm.2	0.82	0.94	0.1	0.08	0.74	0.06	0.45	0.22	0.2	0.28	0.03	0.03	1656.95	14.09	0	14

TG	6	LQHP	2	fc.LQHP_rm.2	0.82	0.93	0.12	0.1	0.73	0.08	0.31	0.31	0.18	0.29	0.03	0.03	1658.71	15.86	0	15
TG	7	LQ	3	fc.LQ_rm.3	0.74	0.73	0.08	0.09	0.72	0.11	0.42	0.2	0.13	0.1	0.01	0.03	1669.83	26.97	0	5
TG	8	LQH	3	fc.LQH_rm.3	0.82	0.94	0.09	0.06	0.74	0.05	0.43	0.22	0.17	0.22	0.03	0.03	1642.86	0	0.75	7
TG	9	LQHP	3	fc.LQHP_rm.3	0.82	0.94	0.09	0.08	0.73	0.05	0.34	0.14	0.2	0.28	0.03	0.03	1645.09	2.23	0.25	8

Note:

Column definitions for model tuning results are provided below for reference.

fc: Feature class combination (e.g., L = linear, Q = quadratic, H = hinge, etc.)

rm: Regularization multiplier controlling model complexity

tune.args: Combined tuning parameters (feature class + regularization multiplier)

auc.train: Area Under the Curve (AUC) on training data

cbi.train: Continuous Boyce Index for the training dataset

auc.diff.avg: Mean difference between training and validation AUC values

auc.diff.sd: Standard deviation of AUC differences across replicates

auc.val.avg: Mean AUC on validation data (predictive performance)

auc.val.sd: Standard deviation of AUC on validation data

cbi.val.avg: Mean Continuous Boyce Index on validation data

cbi.val.sd: Standard deviation of validation CBI

or.10p.avg: Mean threshold at 10% training omission rate

or.10p.sd: Standard deviation of the 10% omission threshold

or.mtp.avg: Mean minimum training presence threshold

or.mtp.sd: Standard deviation of the minimum training threshold

AICc: Corrected Akaike Information Criterion (penalizes model complexity)

delta.AICc: AICc difference compared to the best-performing model

w.AIC: AICc weight (relative likelihood of each model)

ncoef: Number of coefficients used in the model

Table S21. AUC values and variable contributions of MaxEnt models.

**Species**	**Test AUC**	**bio1 contribution**	**bio2 contribution**	**bio12 contribution**	**bio15 contribution**
MG	0.8695	66.4833	1.3684	15.1588	16.9895
**Species**	**Test AUC**	**bio11 contribution**	**bio12 contribution**	**bio17 contribution**	
PG	0.9762	63.9088	15.3832	20.708	
**Species**	**Test AUC**	**bio1 contribution**	**bio2 contribution**	**bio12 contribution**	**bio15 contribution**
TG	0.816	37.9649	7.4683	53.2684	1.2984

Table S22. Average heterozygosity among the three *Procapra* species.

Group	Heterozygosity
MG	0.00226
PG	0.00143
TG	0.00114

Table S23. Genome-wide heterozygosity of *Procapra*.

**Group**	**Sample ID**	**Heterozygosity**
MG	MY01	0.00232
MG	MY02	0.00232
MG	MY03	0.00232
MG	MY04	0.00236
MG	MY05	0.00234
MG	MY06	0.00231
MG	MY08	0.00222
MG	MY10	0.00231
MG	MY11	0.00232
MG	MY16	0.00233
MG	MY18	0.00231
MG	MY19	0.00222
MG	MY22	0.00228
MG	MY24	0.0025
MG	MY26	0.00232
MG	MY28	0.0023
MG	MY32	0.00208
MG	MY35	0.00224
MG	MY36	0.00205
MG	MY37	0.0022
MG	MY38	0.00223
MG	MY39	0.00229

MG	MY41	0.00224
MG	MY42	0.00222
MG	MY43	0.00227
MG	MY44	0.00215
MG	MY45	0.0023
MG	MY46	0.00222
MG	MY47	0.0023
MG	MY48	0.00216
MG	MY49	0.0022
MG	MY50	0.00214
PG	PY02	0.0013
PG	PY05	0.00143
PG	PY06	0.00129
PG	PY10	0.00152
PG	PY17	0.00148
PG	PY23	0.00137
PG	PY24	0.00133
PG	PY25	0.00157
PG	PY26	0.00149
PG	PY27	0.00141
PG	PY29	0.00152
PG	PY31	0.00115
PG	PY32	0.0015
PG	PY33	0.00153
PG	PY34	0.00183

PG	PY36	0.0013
PG	PY37	0.00142
PG	PY39	0.00171
PG	PY40	0.00125
PG	PY42	0.00128
PG	PY43	0.00131
PG	PY44	0.00155
PG	PY45	0.00148
PG	PY46	0.00144
PG	PY47	0.00128
PG	PY48	0.00162
PG	PY49	0.00153
PG	PY50	0.00151
PG	PY51	0.00141
PG	PY52	0.00145
PG	PY53	0.00136
PG	PY54	0.00134
PG	PY55	0.00144
PG	PY56	0.00142
PG	PY57	0.00132
PG	PY58	0.00145
PG	PY59	0.00132
PG	PY60	0.0014
PG	PY61	0.00126
PG	PY62	0.00144

PG	PY68	0.00141
PG	PY70	0.00171
PG	PY71	0.00128
TG	ZY02	0.00072
TG	ZY04	0.0016
TG	ZY06	0.00096
TG	ZY07	0.00085
TG	ZY08	0.00074
TG	ZY10	0.00076
TG	ZY16	0.00143
TG	ZY17	0.00132
TG	ZY18	0.0011
TG	ZY19	0.00117
TG	ZY20	0.00134
TG	ZY21	0.00164

Table S24. Nucleotide diversity (π) estimates for three species of *Procapra*.

Group	Nucleotide diversity (π)
MG	0.00237
PG	0.0016
TG	0.0015

Table S25. Length of introgression region (bp) and genetic diversity across different PG individuals.

IND	Length of introgression region	Length of without introgression region	Introgression region_het	without introgression region_het
PY02	25,807,118	2,504,816,140	0.00207	0.00129
PY05	36,151,801	2,494,471,457	0.00335	0.0014
PY06	33,807,434	2,496,815,824	0.00302	0.00127
PY10	34,483,975	2,496,139,283	0.00312	0.0015
PY17	9,146,439	2,521,476,819	0.00248	0.00148
PY23	20,908,060	2,509,715,198	0.00198	0.00136
PY24	20,068,525	2,510,554,733	0.00256	0.00132
PY25	22,805,180	2,507,818,078	0.00278	0.00156
PY26	20,439,276	2,510,183,982	0.00292	0.00148
PY27	26,958,794	2,503,664,464	0.00289	0.0014
PY29	23,839,229	2,506,784,029	0.00297	0.0015
PY31	20,793,920	2,509,829,338	0.00203	0.00114
PY32	25,700,208	2,504,923,050	0.00302	0.00149
PY33	23,506,732	2,507,116,526	0.00313	0.00152
PY34	21,436,023	2,509,187,235	0.00304	0.00182
PY36	29,417,260	2,501,205,998	0.00288	0.00128
PY37	26,573,883	2,504,049,375	0.00316	0.0014
PY39	15,346,889	2,515,276,369	0.00307	0.0017
PY40	25,280,570	2,505,342,688	0.00248	0.00124
PY42	17,249,257	2,513,374,001	0.00254	0.00128
PY43	22,595,660	2,508,027,598	0.00281	0.00129
PY44	17,060,901	2,513,562,357	0.00246	0.00154

PY45	27,359,895	2,503,263,363	0.00228	0.00147
PY46	23,881,439	2,506,741,819	0.00306	0.00142
PY47	14,352,159	2,516,271,099	0.00161	0.00128
PY48	17,305,464	2,513,317,794	0.00343	0.00161
PY49	27,022,665	2,503,600,593	0.00324	0.00151
PY50	20,789,369	2,509,833,889	0.00281	0.0015
PY51	21,414,332	2,509,208,926	0.00291	0.0014
PY52	17,572,084	2,513,051,174	0.00292	0.00144
PY53	22,689,785	2,507,933,473	0.00309	0.00134
PY54	13,912,843	2,516,710,415	0.0023	0.00134
PY55	14,859,661	2,515,763,597	0.00252	0.00143
PY56	16,112,780	2,514,510,478	0.0022	0.00142
PY57	13,877,076	2,516,746,182	0.00288	0.00131
PY58	19,949,103	2,510,674,155	0.00315	0.00144
PY59	19,144,233	2,511,479,025	0.00258	0.00131
PY60	15,581,944	2,515,041,314	0.00212	0.00139
PY61	14,360,145	2,516,263,113	0.00199	0.00126
PY62	30,995,676	2,499,627,582	0.00283	0.00142
PY68	20,213,185	2,510,410,073	0.00258	0.0014
PY70	37,424,016	2,493,199,242	0.00329	0.00169
PY71	16,639,332	2,513,983,926	0.00159	0.00128

Table S26. The IUCN status and heterozygosity of the *Procapra* and other mammalian species.

Species	Scientific name	Heterozygosity	IUCN Status	Source
White-faced muskox	*Ovibos moschatus wardi*	0.000063	Least Concern	(Pečnerová et al. 2024)
Baiji	*Lipotes vexillifer*	0.000121	Critically Endangered	(Zhou et al. 2013)
Iberian lynx	*Lynx pardinus*	0.00018	Vulnerable	(Westbury et al. 2018)
Snow leopard	*Panthera uncia*	0.00023	Vulnerable	(Cho et al. 2013)
Golden Takin	*Budorcas tibetana*	0.00038	Vulnerable	(Li et al. 2023)
Amur tiger	*Panthera tigris altaica*	0.00049	Endangered	(Cho et al. 2013)
Milu	*Elaphurus davidianus*	0.00051	Extinct in the Wild	(Zhu et al. 2018)
Greater One-horned rhino	*Rhinoceros unicornis*	0.00059	Vulnerable	(Liu et al. 2021)
Moose	*Alces alces*	0.00067	Least Concern	(Dussex et al. 2020)
European bison	*Bison bonasus*	0.00079	Near Threatened	(Wang et al. 2017)
Ancient Siberian muskox	*Ovibos moschatus sp.*	0.00083	Least Concern	(Pečnerová et al. 2024)
Chinese pangolin	*Manis pentadactyla*	0.00085	Critically Endangered	(Heighton et al. 2023)
Snow sheep	*Ovis nivicola*	0.00091	Least Concern	(Upadhyay et al. 2021)
Black rhino	*Diceros bicornis*	0.00091	Critically Endangered	(Liu et al. 2021)
scimitar-horned oryx	*Oryx dammah*	0.00097	Endangered	(Humble et al. 2020)
Asian elephant	*Elephas maximus*	0.000993	Endangered	(Palkopoulou et al. 2018)
Bactrian camel	*Camelus ferus*	0.001	Critically Endangered	(Jirimutu et al. 2012)
Tibetan antelope	*Pantholops hodgsonii*	0.00103	Near Threatened	(Kuang 2021)
Yangtze finless porpoise	*Neophocaena asiaeorientalis*	0.00105	Critically Endangered	(Morin et al. 2021)
Tibetan gazelle	*Procapra picticaudata*	0.001137439	Near Threatened	This study
Sumatran rhinoceros	*Dicerorhinus sumatrensis*	0.00124	Critically Endangered	(von Seth et al. 2021)
Giant panda	*Ailuropoda melanoleuca*	0.00135	Vulnerable	(Li et al. 2010)

Przewalski's gazelle	*Procapra przewalskii*	0.001426565	Endangered	This study
Javan rhinoceros	*Rhinoceros sondaicus*	0.00192	Critically Endangered	(Liu et al. 2021)
Reindeer	*Rangifer tarandus*	0.00205	Vulnerable	(Weldenegodguad et al. 2020)
Mongolian gazelle	*Procapra gutturosa*	0.002260706	Least Concern	This study
African forest elephant	*Loxodonta africana cyclotis*	0.00325	Critically Endangered	(Palkopoulou et al. 2018)
Przewalski's horse	*Equus ferus przewalskii*	0.00363	Endangered	(Corbett-Detig et al. 2015)
Asiatic mouflon	*Ovis gmelini*	0.00367	Near Threatened	(Upadhyay et al. 2021)

Table S27. Inbreeding coefficients (F_{ROH}) estimated for *Procapra* species stratified by ROH length.

Samples	100–500 kb	500–1000 kb	1000–3000 kb	>3000 kb	ALL	F_{ROH} 100–500 kb	F_{ROH} 500–1000 kb	F_{ROH} 1000–3000 kb	F_{ROH} >3000 kb	F_{ROH}_All
MY01	39959.6	0	0	0	39959.6	0.015723572	0	0	0	0.015723572
MY02	45175.7	5437.78	0	0	50613.48	0.017776039	0.002139694	0	0	0.019915733
MY03	36948.5	5178.7	0	0	42127.2	0.014538745	0.00203775	0	0	0.016576494
MY04	31352.2	2586.84	3904.32	0	37843.36	0.012336675	0.001017887	0.001536298	0	0.01489086
MY05	38458.6	2666.34	1467.52	0	42592.46	0.015132949	0.001049169	0.00057745	0	0.016759568
MY06	34540	6146.29	0	0	40686.29	0.013591032	0.002418484	0	0	0.016009515
MY08	40150.7	1868.56	1239.45	0	43258.71	0.015798768	0.000735254	0.000487707	0	0.017021728
MY10	36357.3	2401.72	1924.74	0	40683.76	0.014306115	0.000945045	0.00075736	0	0.01600852
MY11	40532.5	3997.83	1146.23	0	45676.56	0.015949001	0.001573093	0.000451026	0	0.01797312
MY16	39981.7	13740.6	15043.8	0	68766.1	0.015732269	0.005406744	0.005919536	0	0.027058548
MY18	24873.7	1052.21	0	0	25925.91	0.009787471	0.000414031	0	0	0.010201502
MY19	30898.3	1816.37	0	0	32714.67	0.012158071	0.000714717	0	0	0.012872789
MY22	32863.4	2678.36	0	0	35541.76	0.012931312	0.001053899	0	0	0.013985211
MY24	8763.45	1606.25	0	0	10369.7	0.003448301	0.000632038	0	0	0.004080339
MY26	26226.7	654.233	0	0	26880.933	0.010319859	0.000257432	0	0	0.010577291
MY28	30401.9	0	0	0	30401.9	0.011962744	0	0	0	0.011962744
MY32	20394.8	0	0	0	20394.8	0.008025083	0	0	0	0.008025083
MY35	29780.3	637.198	0	0	30417.498	0.011718153	0.000250729	0	0	0.011968882
MY36	32767.8	2521.16	0	0	35288.96	0.012893695	0.000992043	0	0	0.013885738
MY37	35492.5	3218.66	0	0	38711.16	0.013965828	0.0012665	0	0	0.015232328
MY38	28225.5	3021.17	1054.11	0	32300.78	0.01110636	0.00118879	0.000414778	0	0.012709928
MY39	34582	5351.01	0	0	39933.01	0.013607558	0.002105551	0	0	0.01571311

MY41	40356.1	3179.95	1106.16	0	44642.21	0.01587959	0.001251268	0.000435259	0	0.017566117
MY42	32558	1632.54	0	0	34190.54	0.012811141	0.000642383	0	0	0.013453524
MY43	37328.4	531.227	0	0	37859.627	0.01468823	0.000209031	0	0	0.014897261
MY44	32456.5	2313.12	0	0	34769.62	0.012771202	0.000910182	0	0	0.013681384
MY45	28102.5	3016.47	0	0	31118.97	0.011057961	0.001186941	0	0	0.012244902
MY46	31167.2	515.171	0	0	31682.371	0.01226388	0.000202713	0	0	0.012466593
MY47	28661.8	1877.59	0	0	30539.39	0.011278038	0.000738807	0	0	0.012016845
MY48	43186.1	1150.68	0	0	44336.78	0.016993157	0.000452777	0	0	0.017445935
MY49	29923	1333.77	0	0	31256.77	0.011774304	0.000524821	0	0	0.012299124
MY50	39079.6	2297.65	0	0	41377.25	0.015377304	0.000904095	0	0	0.016281399
PY02	366451	211705	257457	30522.1	866135.1	0.14480662	0.083657257	0.101736598	0.0120611	0.342261574
PY05	373982	124306	82173.7	0	580461.7	0.147782567	0.049120706	0.032471724	0	0.229374996
PY06	444978	199209	179534	3305.2	827026.2	0.175837315	0.078719343	0.070944578	0.001306081	0.326807318
PY10	343354	119924	145628	30218.1	639124.1	0.135679619	0.047389116	0.057546298	0.011940971	0.252556005
PY17	312207	73834.6	73867.3	14028	473936.9	0.123371584	0.029176449	0.029189371	0.005543298	0.187280702
PY23	346272	146532	190378	54798.5	737980.5	0.136832695	0.057903522	0.075229689	0.021654152	0.291620057
PY24	368701	97214.6	127574	23625.3	617114.9	0.145695729	0.03841528	0.050412087	0.009335763	0.243858859
PY25	334503	89485.6	93864.9	50903.1	568756.6	0.132182062	0.035361091	0.037091614	0.020114847	0.224749614
PY26	348815	111139	82878.8	34586.1	577418.9	0.137837586	0.04391764	0.032750351	0.013667028	0.228172605
PY27	341913	83994.3	124384	96136	646427.3	0.135110194	0.033191152	0.049151528	0.03798906	0.255441934
PY29	351519	102367	121909	61521.2	637316.2	0.138906097	0.0404513	0.048173508	0.024310691	0.251841596
PY31	322046	162075	381241	171682	1037044	0.127259559	0.064045487	0.15065103	0.067841785	0.409797862
PY32	352780	91930.5	137277	27672.4	609659.9	0.139404393	0.036327217	0.05424632	0.010935014	0.240912944
PY33	373106	99652.7	108857	31179.1	612794.8	0.147436407	0.039378718	0.043015885	0.01232072	0.24215173
PY34	27627	0	0	0	27627	0.010917073	0	0	0	0.010917073

PY36	315240	138330	289439	161842	904851	0.124570103	0.054662423	0.114374591	0.063953415	0.357560533
PY37	349860	128900	136559	28009.6	643328.6	0.138250527	0.050936069	0.053962596	0.011068262	0.254217453
PY39	303996	44558.7	14732	3373.6	366660.3	0.120126929	0.017607797	0.005821491	0.00133311	0.144889327
PY40	397221	165594	171861	3657.82	738333.82	0.15696568	0.065436054	0.067912519	0.001445423	0.291759675
PY42	345850	133739	227602	74325.9	781516.9	0.136665937	0.052848246	0.089939109	0.029370591	0.308823883
PY43	307523	136845	315835	139407	899610	0.121520657	0.054075611	0.124805223	0.05508801	0.355489501
PY44	332292	108763	134710	19958.7	595723.7	0.131308364	0.04297874	0.053231946	0.007886871	0.235405921
PY45	352016	103893	93098.1	39867	588874.1	0.139102491	0.041054313	0.036788605	0.015753827	0.232699236
PY46	329255	103526	126544	77967.3	637292.3	0.130108264	0.04090929	0.050005073	0.030809525	0.251832152
PY47	336500	129375	285875	80016.8	831766.8	0.132971196	0.051123769	0.112966242	0.031619404	0.328680612
PY48	343416	91196.1	65659.9	11301.2	511573.2	0.135704119	0.036037012	0.025946138	0.004465777	0.202153046
PY49	322865	121519	137331	55228.9	636943.9	0.127583195	0.048019396	0.054267659	0.021824228	0.251694478
PY50	363572	94696.7	68723.5	3940.51	530932.71	0.143668955	0.037420307	0.027156749	0.00155713	0.209803142
PY51	354296	109760	128218	50737.6	643011.6	0.140003455	0.043372714	0.05066657	0.020049448	0.254092188
PY52	363146	112655	104000	20554.2	600355.2	0.143500617	0.044516701	0.041096595	0.008122189	0.237236103
PY53	365971	120330	113694	26622.2	626617.2	0.144616943	0.047549551	0.044927272	0.010520017	0.247613784
PY54	376205	133455	202445	10896.9	723001.9	0.148661006	0.05273602	0.079998079	0.004306014	0.28570112
PY55	380751	104226	91455.8	12841.5	589274.3	0.150457402	0.041185901	0.036139635	0.005074442	0.232857379
PY56	343745	134827	154833	18639.2	652044.2	0.135834127	0.053278179	0.061183742	0.007365458	0.257661506
PY57	388031	166237	157996	10076.6	722340.6	0.153334163	0.065690142	0.062433632	0.003981865	0.285439801
PY58	380201	124346	173588	28024.8	706159.8	0.150240064	0.049136512	0.06859496	0.011074268	0.279045803
PY59	331049	112904	211889	78776.4	734618.4	0.130817181	0.044615096	0.083729966	0.031129248	0.290291492
PY60	340855	128070	182769	42035	693729	0.134692115	0.050608086	0.07222292	0.016610533	0.274133654
PY61	368684	129642	182797	14558.6	695681.6	0.145689011	0.051229277	0.072233984	0.00575297	0.274905242
PY62	351991	127377	129857	31104.4	640329.4	0.139092612	0.050334241	0.051314236	0.012291201	0.253032291

PY68	329249	123204	225426	79958.9	757837.9	0.130105893	0.04868524	0.089079241	0.031596525	0.299466899
PY70	316779	135057	164483	7663.6	623982.6	0.125178254	0.053369066	0.064997032	0.003028345	0.246572696
PY71	298430	126003	249730	200838	875001	0.117927471	0.049791291	0.0986832	0.079363058	0.345765019
ZY02	637731	179851	251663	24295.4	1093540.4	0.231634039	0.065324743	0.091408003	0.008824476	0.397191261
ZY04	70650.2	31175.4	67294.3	6829.11	175949.01	0.025661276	0.011323401	0.02444236	0.002480441	0.063907478
ZY06	548277	55110.1	76392.1	10305.6	690084.8	0.199142924	0.020016865	0.027746825	0.003743158	0.250649772
ZY07	520271	35216.5	47863.8	11794.7	615146	0.1889707	0.012791193	0.017384893	0.004284023	0.223430808
ZY08	811983	142860	139532	27171.4	1121546.4	0.294925136	0.051889023	0.050680241	0.009869085	0.407363485
ZY10	796505	94081.8	91230.9	8046.61	989864.31	0.28930328	0.034172006	0.033136513	0.002922657	0.359534456
ZY16	78527.3	49977.4	119110	48804.5	296419.2	0.028522364	0.018152586	0.043262646	0.01772657	0.107664167
ZY17	331268	15474.5	28151.9	4437.34	379331.74	0.120321805	0.005620584	0.010225218	0.001611712	0.137779319
ZY18	214694	13866.1	15514.3	3295.19	247369.59	0.077980274	0.005036388	0.00563504	0.001196865	0.089848568
ZY19	264677	10230.8	7244.12	0	282151.92	0.096134895	0.003715989	0.00263118	0	0.102482064
ZY20	320039	21425.1	26857.5	10419.6	378741.2	0.116243253	0.007781937	0.009755071	0.003784564	0.137564826
ZY21	71552.3	29331.4	34364.2	0	135247.9	0.025988933	0.010653631	0.012481624	0	0.049124188

Table S28. Statistics of putatively derived functional variants in *Procapra* based on SnpEff and SIFT4G annotations.

Sample	heterozygous genotypes				homozygous derived genotypes			
	TOL	SYN	DEL	LOF	TOL	SYN	DEL	LOF
MY01	6140	15127	2438	152	2278	5020	576	40
MY02	6163	15303	2457	138	2354	5172	600	38
MY03	6143	15043	2514	150	2377	5279	639	51
MY04	6258	15476	2571	143	2258	5176	616	39
MY05	6204	15183	2509	143	2276	5228	603	48
MY06	6275	15186	2477	143	2320	5114	556	40
MY08	5927	14470	2447	135	2439	5318	586	50
MY10	6213	14960	2510	124	2324	5406	572	43
MY11	6216	15029	2474	139	2315	5192	593	43
MY16	6337	15317	2518	138	2342	5268	604	44
MY18	6094	14943	2597	131	2372	5198	625	44
MY19	6154	14655	2449	138	2306	5257	618	37
MY22	6026	14725	2472	137	2383	5311	620	53
MY24	6866	16500	2657	150	2046	4523	529	36
MY26	6260	15348	2516	145	2282	5039	610	40
MY28	6065	14660	2379	153	2371	5346	622	41
MY32	6197	15134	2542	137	2302	5091	608	40
MY35	6248	15115	2537	127	2356	5149	627	40
MY36	5834	14315	2336	100	2404	5392	640	47
MY37	5999	14752	2401	138	2403	5332	653	53
MY38	6293	15061	2481	145	2258	5198	537	37

MY39	6186	14887	2474	123	2403	5318	638	53
MY41	6082	14458	2439	142	2294	5300	588	52
MY42	5905	14606	2373	124	2481	5371	641	44
MY43	6275	14888	2485	124	2312	5242	600	53
MY44	5953	14077	2337	123	2402	5422	656	39
MY45	6026	14599	2491	150	2373	5308	615	42
MY46	5923	14147	2380	141	2326	5326	667	50
MY47	6154	15221	2531	141	2316	5149	573	48
MY48	6041	14575	2377	104	2409	5351	661	42
MY49	6114	14623	2423	150	2288	5317	611	36
MY50	5837	14173	2405	127	2462	5328	611	48
PY02	4300	9704	1614	87	2785	6101	776	40
PY05	4719	10958	1772	118	2616	5647	669	48
PY06	4143	9899	1641	95	2834	6135	733	45
PY10	4818	10984	1839	102	2557	5546	688	44
PY17	4260	10262	1662	97	2889	5983	711	48
PY23	4496	10092	1643	103	2501	5717	720	48
PY24	4477	10144	1683	92	2066	4383	572	37
PY25	5049	11665	1897	108	2026	4413	540	32
PY26	5033	11533	2020	108	2219	4842	565	28
PY27	4421	9741	1413	78	35	82	11	0
PY29	5057	11605	1891	125	2176	4842	595	32
PY31	3752	8424	1428	77	2824	6111	816	51
PY32	4874	11480	1839	111	2144	4740	598	37
PY33	4832	11247	1853	101	2192	4881	642	31

PY34	5287	11763	1902	102	2478	5522	634	44
PY36	4732	10564	1830	116	2466	5614	724	34
PY37	4790	10738	1894	109	2316	5371	657	39
PY39	5203	11994	1897	110	2505	5330	653	32
PY40	4578	10401	1789	115	2621	5853	693	38
PY42	4204	9569	1689	104	2680	5985	783	38
PY43	4439	9576	1686	95	2557	5887	717	37
PY44	4987	11322	1995	111	2343	5179	632	44
PY45	4925	11220	1913	108	2354	5032	609	38
PY46	4892	11212	1890	112	2392	5194	609	33
PY47	4476	10205	1801	101	2478	5554	708	44
PY48	5406	12042	2058	135	2156	4747	561	28
PY49	5140	11372	1954	107	2037	4713	546	31
PY50	4994	11429	1993	129	2145	4587	569	24
PY51	4634	10798	1862	107	2219	4747	573	35
PY52	4945	11205	1838	114	2313	5067	635	45
PY53	4653	10599	1760	106	2440	5426	684	43
PY54	4762	11008	1831	105	2495	5400	703	46
PY55	4813	11444	1956	116	2504	5319	675	39
PY56	4694	10681	1880	103	2518	5545	663	39
PY57	4608	10560	1874	107	2601	5800	735	37
PY58	4924	11085	1910	97	2416	5468	731	52
PY59	4483	10194	1802	94	2622	5762	725	44
PY60	4825	10968	1850	107	2397	5199	719	42
PY61	4582	10599	1830	107	2509	5465	669	46

PY62	4805	10857	1853	126	2349	5353	680	35
PY68	4710	10487	1718	103	2535	5907	768	39
PY70	5715	13197	2104	109	2610	5521	637	45
PY71	4124	9261	1578	91	2835	6175	776	46
ZY02	2765	5631	1324	67	4142	8760	1485	74
ZY04	4789	10119	1928	106	2217	4440	594	40
ZY06	3874	7684	1683	79	3470	7448	1161	70
ZY07	3438	6823	1519	74	3701	7950	1266	70
ZY08	2945	5854	1350	58	4048	8588	1356	78
ZY10	2990	6010	1387	65	3876	8475	1310	68
ZY16	4455	9368	1846	98	2452	4785	688	37
ZY17	4400	8824	1799	104	1622	3310	527	36
ZY18	3927	8214	1597	92	1638	3500	614	33
ZY19	4104	8456	1734	93	1602	3324	551	26
ZY20	4307	8984	1789	88	1551	3251	535	33
ZY21	4866	10482	1998	114	2247	4419	617	38

Table S29. Numbers of derived variants located within ROH and non-ROH regions across the three *Procapra* species.

Group	Sample ID	In ROH region						In non-ROH region					
		**heterozygous derived genotypes**			**homozygous derived genotypes (×2)**			**heterozygous derived genotypes**			**homozygous derived genotypes (×2)**		
		DEL	LOF	SYN	DEL	LOF	SYN	DEL	LOF	SYN	DEL	LOF	SYN
MY	MY01	1	1	3	34	2	150	2437	151	15124	1118	78	9890
MY	MY02	0	0	5	36	4	204	2457	138	15298	1164	72	10140
MY	MY03	0	1	1	26	2	154	2514	149	15042	1252	100	10404
MY	MY04	1	0	1	36	0	144	2570	143	15475	1196	78	10208
MY	MY05	2	0	3	40	2	154	2507	143	15180	1166	94	10302
MY	MY06	0	0	5	10	2	152	2477	143	15181	1102	78	10076
MY	MY08	1	1	4	18	2	138	2446	134	14466	1154	98	10498
MY	MY10	2	0	3	42	0	252	2508	124	14957	1102	86	10560
MY	MY11	0	0	3	34	10	242	2474	139	15026	1152	76	10142
MY	MY16	3	1	3	82	2	394	2515	137	15314	1126	86	10142
MY	MY18	2	0	5	24	2	142	2595	131	14938	1226	86	10254
MY	MY19	2	0	2	30	2	264	2447	138	14653	1206	72	10250
MY	MY22	0	0	6	58	2	182	2472	137	14719	1182	104	10440
MY	MY24	0	1	1	2	0	4	2657	149	16499	1056	72	9042
MY	MY26	0	1	9	26	4	82	2516	144	15339	1194	76	9996
MY	MY28	3	0	1	34	0	166	2376	153	14659	1210	82	10526
MY	MY32	0	1	3	14	0	100	2542	136	15131	1202	80	10082
MY	MY35	4	1	6	44	6	152	2533	126	15109	1210	74	10146
MY	MY36	2	1	0	22	2	108	2334	99	14315	1258	92	10676
MY	MY37	2	2	2	32	2	190	2399	136	14750	1274	104	10474

MY	MY38	1	1	2	30	2	110	2480	144	15059	1044	72	10286
MY	MY39	3	0	4	50	2	196	2471	123	14883	1226	104	10440
MY	MY41	1	0	0	12	2	166	2438	142	14458	1164	102	10434
MY	MY42	3	0	11	52	2	242	2370	124	14595	1230	86	10500
MY	MY43	1	1	8	26	6	236	2484	123	14880	1174	100	10248
MY	MY44	2	0	4	18	4	184	2335	123	14073	1294	74	10660
MY	MY45	1	0	7	36	0	154	2490	150	14592	1194	84	10462
MY	MY46	1	0	3	32	0	154	2379	141	14144	1302	100	10498
MY	MY47	1	1	1	14	0	142	2530	140	15220	1132	96	10156
MY	MY48	1	0	7	62	0	246	2376	104	14568	1260	84	10456
MY	MY49	0	0	3	24	0	162	2423	150	14620	1198	72	10472
MY	MY50	0	0	6	32	2	156	2405	127	14167	1190	94	10500
PY	PY02	34	2	80	900	36	6284	1580	85	9624	652	44	5918
PY	PY05	25	0	66	578	26	4450	1747	118	10892	760	70	6844
PY	PY06	25	1	84	836	40	6450	1616	94	9815	630	50	5820
PY	PY10	24	2	69	656	52	4894	1815	100	10915	720	36	6198
PY	PY17	15	1	53	536	42	4128	1647	96	10209	886	54	7838
PY	PY23	36	1	102	794	52	5522	1607	102	9990	646	44	5912
PY	PY24	24	0	78	588	30	3806	1659	92	10066	556	44	4960
PY	PY25	33	4	68	462	26	3506	1864	104	11597	618	38	5320
PY	PY26	24	2	89	480	14	3674	1996	106	11444	650	42	6010
PY	PY27	11	0	56	0	0	2	1402	78	9685	22	0	162
PY	PY29	23	3	89	594	26	4410	1868	122	11516	596	38	5274
PY	PY31	30	2	88	1204	64	8058	1398	75	8336	428	38	4164
PY	PY32	31	3	77	604	44	4254	1808	108	11403	592	30	5226

PY	PY33	29	1	94	638	14	4710	1824	100	11153	646	48	5052
PY	PY34	2	0	20	14	0	198	1900	102	11743	1254	88	10846
PY	PY36	26	0	64	866	30	5792	1804	116	10500	582	38	5436
PY	PY37	22	1	85	684	34	4748	1872	108	10653	630	44	5994
PY	PY39	13	0	48	338	16	2440	1884	110	11946	968	48	8220
PY	PY40	39	2	79	752	30	5750	1750	113	10322	634	46	5956
PY	PY42	38	2	111	944	54	6622	1651	102	9458	622	22	5348
PY	PY43	24	2	63	928	46	7042	1662	93	9513	506	28	4732
PY	PY44	21	2	68	648	34	4776	1974	109	11254	616	54	5582
PY	PY45	23	4	81	566	26	4212	1890	104	11139	652	50	5852
PY	PY46	17	1	72	564	22	4364	1873	111	11140	654	44	6024
PY	PY47	24	2	92	872	48	5928	1777	99	10113	544	40	5180
PY	PY48	27	0	73	462	14	3462	2031	135	11969	660	42	6032
PY	PY49	25	1	70	514	26	4034	1929	106	11302	578	36	5392
PY	PY50	27	3	84	494	16	3300	1966	126	11345	644	32	5874
PY	PY51	25	1	86	504	32	3936	1837	106	10712	642	38	5558
PY	PY52	20	1	84	592	38	4010	1818	113	11121	678	52	6124
PY	PY53	34	3	95	724	30	4946	1726	103	10504	644	56	5906
PY	PY54	25	0	103	708	46	4856	1806	105	10905	698	46	5944
PY	PY55	33	2	87	600	36	4488	1923	114	11357	750	42	6150
PY	PY56	24	0	89	670	44	5244	1856	103	10592	656	34	5846
PY	PY57	28	5	97	814	38	5732	1846	102	10463	656	36	5868
PY	PY58	23	1	98	826	54	5504	1887	96	10987	636	50	5432
PY	PY59	45	4	74	832	44	5760	1757	90	10120	618	44	5764
PY	PY60	23	0	84	720	52	4882	1827	107	10884	718	32	5516

PY	PY61	17	1	82	664	48	4840	1813	106	10517	674	44	6090
PY	PY62	36	2	89	710	38	4674	1817	124	10768	650	32	6032
PY	PY68	25	1	75	950	42	6096	1693	102	10412	586	36	5718
PY	PY70	20	1	70	666	38	4822	2084	108	13127	608	52	6220
PY	PY71	16	0	65	958	42	7062	1562	91	9196	594	50	5288
ZY	ZY02	32	0	96	1644	70	9700	1292	67	5535	1326	78	7820
ZY	ZY04	0	0	3	90	4	506	1928	106	10116	1098	76	8374
ZY	ZY06	24	1	81	808	44	4528	1659	78	7603	1514	96	10368
ZY	ZY07	15	1	59	788	30	4548	1504	73	6764	1744	110	11352
ZY	ZY08	29	1	85	1418	70	8030	1321	57	5769	1294	86	9146
ZY	ZY10	28	1	103	1168	48	7386	1359	64	5907	1452	88	9564
ZY	ZY16	1	0	9	222	12	1310	1845	98	9359	1154	62	8260
ZY	ZY17	13	0	41	254	10	1492	1786	104	8783	800	62	5128
ZY	ZY18	3	0	25	248	14	1364	1594	92	8189	980	52	5636
ZY	ZY19	15	0	31	206	8	1302	1719	93	8425	896	44	5346
ZY	ZY20	13	1	45	284	26	1460	1776	87	8939	786	40	5042
ZY	ZY21	0	0	6	98	4	486	1998	114	10476	1136	72	8352

Table S30. Genes carrying loss-of-function (LOF) variants retained after MAF ≥ 0.05 filtering in the three *Procapra* species. Gene symbols and functional descriptions were derived from species-specific eggNOG-mapper annotations. For genes carrying multiple LOF variants, variants and their corresponding ALT allele frequencies are separated by semicolons and listed in the same order. "Unknown" indicates that no corresponding annotation was available.

Species	Gene ID in the genome annotation	Gene symbol	Description	LOF variant(s)	ALT allele frequency(s)
MG	MY.1G.00003	SGOL1	Shugoshin-like 1	Chr1:129435 G>A	0.068965
MG	MY.1G.00192	PFDN4	Prefoldin subunit	Chr1:24724055 G>A	0.078125
MG	MY.1G.00229	TPT1	negative regulation of ectoderm development	Chr1:29910251 G>A	0.203125
MG	MY.1G.00320	hnrnpa3	heterogeneous nuclear ribonucleoprotein	Chr1:48475619 A>G; Chr1:48476240 G>A	0.09375; 0.375
MG	MY.1G.00429	HTR3D	Neurotransmitter-gated ion-channel ligand binding domain	Chr1:74561189 G>A	0.0625
MG	MY.1G.00430	HTR3C	Belongs to the ligand-gated ion channel (TC 1.A.9) family	Chr1:74586166 T>C	0.09375
MG	MY.1G.00432	OAZ1	Ornithine decarboxylase antizyme 1	Chr1:74611052 A>C	0.296875
MG	MY.1G.00467	MRPL50	ribosomal protein L50	Chr1:76971303 G>A	0.578125
MG	MY.1G.00523	NDUFA4	NADH-ubiquinone reductase complex 1 MLRQ subunit	Chr1:85699655 T>C	0.09375
MG	MY.1G.00533	PIGX	Phosphatidylinositol glycan anchor biosynthesis, class X	Chr1:86554148 A>T	0.145161
MG	MY.1G.00550	MUC4	Mucin 4, cell surface associated	Chr1:87275406 G>A; Chr1:87275407 G>A	0.09375; 0.109375
MG	MY.1G.00583	CCDC58	Coiled-coil domain-containing protein 58	Chr1:90987368 C>T	0.0625
MG	MY.1G.00663	Unknown	Unknown	Chr1:103031692 C>G	0.078125
MG	MY.1G.00742	CPSF6	cleavage and polyadenylation specificity factor subunit	Chr1:117661649 G>A	0.265625
MG	MY.1G.00745	Unknown	Unknown	Chr1:119374567 G>A	0.09375
MG	MY.1G.00842	SYNJ1	Synaptojanin 1	Chr1:156472796 G>A	0.125
MG	MY.2G.00008	CYFIP1	Cytoplasmic FMR1 interacting protein 1	Chr2:9012509 C>T	0.0625
MG	MY.2G.00041	INPP1	inositol	Chr2:14100842 C>T	0.09375
MG	MY.2G.00067	USP6NL	USP6 N-terminal-like protein	Chr2:19319143 A>T	0.109375
MG	MY.2G.00086	ZNF385B	zinc finger protein 385B	Chr2:25615252 T>C	0.0625
MG	MY.2G.00128	TMEM183A	transmembrane protein 183A	Chr2:30813066 C>G; Chr2:30813259 C>A	0.0625; 0.25

MG	MY.2G.00155	EIF3M	Eukaryotic translation initiation factor 3 subunit involved in translation initiation.	Chr2:33523667 G>T	0.1875
MG	MY.2G.00161	MYO3B	cochlea morphogenesis	Chr2:34264013 C>A; Chr2:34281256 G>T	0.0625; 0.0625
MG	MY.2G.00221	MAP3K7	Mitogen-activated protein kinase kinase kinase	Chr2:45382870 G>A	0.1875
MG	MY.2G.00226	PLAA	Phospholipase A2-activating protein	Chr2:46304375 T>C	0.078125
MG	MY.2G.00229	CIR1	Corepressor interacting with RBPJ 1	Chr2:46709680 T>C	0.216667
MG	MY.2G.00332	GLI2	zinc finger	Chr2:82064488 C>T	0.15625
MG	MY.2G.00340	Unknown	Thymosin	Chr2:82974862 C>A	0.0625
MG	MY.2G.00343	Unknown	Unknown	Chr2:84444983 C>T	0.140625
MG	MY.2G.00396	PPIL3	Peptidyl-prolyl cis-trans isomerase involved in protein folding.	Chr2:99163967 A>G	0.064516
MG	MY.2G.00422	CCT6A	chaperonin containing TCP1, subunit	Chr2:100968780 G>A	0.140625
MG	MY.2G.00431	NRP2	C-terminal domain of neuropilin glycoprotein	Chr2:103733472 A>G	0.359375
MG	MY.2G.00432	Unknown	Unknown	Chr2:103960342 T>A	0.1875
MG	MY.2G.00459	IDH1	Isocitrate dehydrogenase	Chr2:106296690 C>T	0.16129
MG	MY.2G.00463	MSL3	Male-specific lethal 3 homolog	Chr2:106391522 C>T	0.09375
MG	MY.2G.00531	SLC23A3	Solute carrier family 23 member 3	Chr2:117043611 T>A	0.140625
MG	MY.2G.00548	SPEG	Immunoglobulin C-2 Type	Chr2:117286565 G>A	0.078125
MG	MY.2G.00596	OSBPL9	Belongs to the OSBP family	Chr2:126629570 G>T	0.0625
MG	MY.2G.00688	PUM1	family) member 1	Chr2:132561045 G>A	0.421875
MG	MY.2G.00691	Unknown	Unknown	Chr2:134123152 C>G	0.53125
MG	MY.2G.00747	RPS6KA1	ribosomal protein S6 kinase	Chr2:136547113 G>A	0.53125
MG	MY.2G.00828	ALPL	alkaline phosphatase	Chr2:141140304 A>G	0.90625
MG	MY.3G.00015	Unknown	Unknown	Chr3:2534202 C>G	0.0625
MG	MY.3G.00031	PRKAG2	5'-AMP-activated protein kinase subunit gamma-2	Chr3:5947033 C>A	7.81E-02
MG	MY.3G.00102	Olfr13	Olfactory receptor	Chr3:12733244 A>G	0.209677

MG	MY.3G.00148	Unknown	Immunoglobulin V-set domain	Chr3:14117974 C>G	0.21875
MG	MY.3G.00151	Unknown	Immunoglobulin V-set domain	Chr3:14177518 C>A	0.189655
MG	MY.3G.00153	DYNLL1	dynein, light chain	Chr3:14205019 C>T	0.171875
MG	MY.3G.00316	TARP	Immunoglobulin C-Type	Chr3:36789794 A>T	0.129032
MG	MY.3G.00318	TARP	Immunoglobulin C-Type	Chr3:36920584 G>A	0.203125
MG	MY.3G.00362	Unknown	Unknown	Chr3:43365812 T>C	0.890625
MG	MY.3G.00430	INMT	substituted 1 base at 1 genomic stop codon	Chr3:53996016 G>T	0.0625
MG	MY.3G.00468	AIP	Aryl hydrocarbon receptor interacting protein	Chr3:60043032 G>T	0.078125
MG	MY.3G.00497	TARP	Immunoglobulin C-Type	Chr3:69617737 A>G	0.435484
MG	MY.3G.00568	EIF3M	Component of the eIF-3 complex involved in translation initiation and regulation of protein synthesis.	Chr3:83226837 A>C	0.15625
MG	MY.3G.00633	USP53	Ubiquitin specific peptidase 53	Chr3:97885452 G>A	0.21875
MG	MY.3G.00668	DYNC1I1	1, intermediate chain 1	Chr3:107287847 G>A	0.140625
MG	MY.3G.00708	ABCA13	cassette subfamily A	Chr3:112619127 G>A; Chr3:112685775 C>A; Chr3:112873767 T>A	0.125; 0.171875; 0.0625
MG	MY.3G.00709	UPP1	Uridine phosphorylase 1	Chr3:112779747 G>A	0.140625
MG	MY.4G.00123	CRP	C-reactive	Chr4:14431199 G>T	0.109375
MG	MY.4G.00163	CD1A	T-cell surface glycoprotein CD1a-like	Chr4:16286435 C>T	0.546875
MG	MY.4G.00175	MMGT1	Membrane magnesium transporter	Chr4:17282371 A>G	0.259259
MG	MY.4G.00180	Unknown	Immunoglobulin	Chr4:17679082 C>T; Chr4:17695189 A>G	0.078125; 0.1875
MG	MY.4G.00200	TTC24	Tetratricopeptide repeat protein 24	Chr4:18306243 T>A	0.40625
MG	MY.4G.00204	RHBG	Rh family B glycoprotein	Chr4:18512859 C>T	0.40625
MG	MY.4G.00232	ZSCAN21	Zinc finger and SCAN	Chr4:19246859 C>T; Chr4:19246883 G>T	0.078125; 0.0625
MG	MY.4G.00359	PI4KB	Phosphatidylinositol 4-kinase	Chr4:23491599 C>T	0.671875
MG	MY.4G.00463	NBPF3	Repeat of unknown function (DUF1220)	Chr4:27300608 T>C	0.051724

MG	MY.4G.00525	RPL22L1	cytoplasmic translation	Chr4:35168808 T>C	0.0625
MG	MY.4G.00596	AKNAD1	AT-hook-containing transcription factor	Chr4:39295554 T>A	0.125
MG	MY.4G.00602	Unknown	Calcium-binding mitochondrial carrier protein SCaMC-1-like	Chr4:39596615 T>A	0.0625
MG	MY.4G.00608	GAPDH	Glycolytic enzyme involved in energy metabolism, nitrosylation, and nuclear regulation.	Chr4:42773715 A>G	0.078125
MG	MY.4G.00646	Unknown	Unknown	Chr4:52182636 G>A	0.09375
MG	MY.4G.00678	Lpcat2b	Phosphate acyltransferases	Chr4:56121932 G>A	0.078125
MG	MY.4G.00683	HFM1	ATP-dependent DNA helicase	Chr4:56880130 G>A	0.09375
MG	MY.4G.00684	ATP5A1	proton-transporting ATP synthase activity, rotational mechanism	Chr4:57040295 A>G	0.078125
MG	MY.4G.00711	hnrnpa1	Heterogeneous nuclear ribonucleoprotein A1-like	Chr4:61510591 G>A	0.0625
MG	MY.4G.00761	ST6GALNAC3	Glycosyltransferase family 29 (sialyltransferase)	Chr4:72666503 T>C	0.390625
MG	MY.4G.00796	Unknown	Unknown	Chr4:81752020 T>C	0.1875
MG	MY.4G.00817	Unknown	Unknown	Chr4:84567909 G>A	0.078125
MG	MY.4G.00842	C1orf87	Chromosome 1 open reading frame 87	Chr4:90023628 T>C	0.328125
MG	MY.4G.00848	IDH1	Isocitrate dehydrogenase	Chr4:90432212 C>T	0.234375
MG	MY.4G.00878	ACOT11	in StAR and phosphatidylcholine transfer protein	Chr4:95958464 G>A	0.0625
MG	MY.4G.00903	ECHDC2	enoyl-CoA, hydratase	Chr4:97646253 G>C	0.21875
MG	MY.4G.00920	NRD1	Nardilysin	Chr4:98711731 G>A	0.109375
MG	MY.4G.00950	CYP4A11	16-hydroxypalmitate dehydrogenase activity	Chr4:103445114 G>T	0.140625
MG	MY.4G.01173	RALY	RNA-binding protein Raly isoform	Chr4:117736146 G>A	0.171875
MG	MY.4G.01174	MROH2A	family member	Chr4:117737193 C>T	0.15625
MG	MY.4G.01207	Unknown	Unknown	Chr4:122343060 C>T	0.25
MG	MY.4G.01249	RTP5	transporter protein 5	Chr4:124540263 A>T	0.1875
MG	MY.4G.01250	Unknown	Unknown	Chr4:124586058 C>T	0.0625
MG	MY.4G.01251	WBP11	WW domain-binding protein	Chr4:124634805 C>T	0.21875

MG	MY.5G.00032	POLN	DNA polymerase	Chr5:1209430 C>T	0.90625
MG	MY.5G.00042	SH3BP2	binding protein 2	Chr5:1857575 T>C	0.828125
MG	MY.5G.00046	HTT	huntingtin	Chr5:2177335 C>T	0.140625
MG	MY.5G.00074	MAN2B2	Alpha mannosidase, middle domain	Chr5:4769506 A>G	0.15625
MG	MY.5G.00075	MAN2B2	Alpha mannosidase, middle domain	Chr5:4792365 T>C; Chr5:4800734 C>T	0.419355; 0.15625
MG	MY.5G.00097	WBP11	WW domain-binding protein 11	Chr5:11382421 G>A	0.09375
MG	MY.5G.00128	AFF1	AF4 FMR2 family, member	Chr5:15688825 C>T	0.09375
MG	MY.5G.00157	NAP1L1	nucleosome assembly	Chr5:20416403 C>T	0.125
MG	MY.5G.00163	BRCC3	BRCA1 BRCA2-containing complex, subunit 3	Chr5:22368621 A>C	0.109375
MG	MY.5G.00173	FRAS1	extracellular matrix	Chr5:24410799 G>A	0.15625
MG	MY.5G.00245	Unknown	Unknown	Chr5:31902376 C>T; Chr5:31904273 C>A	0.064516; 0.1875
MG	MY.5G.00246	CSN1S2	Important role in the capacity of milk to transport calcium phosphate	Chr5:31902376 C>T; Chr5:31904273 C>A	0.064516; 0.1875
MG	MY.5G.00250	SULT1B1	Sulfotransferase family cytosolic 1B member 1	Chr5:32139531 G>T	0.203125
MG	MY.5G.00311	hnrnpa1	mRNA transport	Chr5:45511647 G>A	0.0625
MG	MY.5G.00361	Rps4x	ribosomal protein S4	Chr5:55160536 A>T	0.203125
MG	MY.5G.00362	RPS12	Ribosomal protein S12	Chr5:55229760 C>T	0.1875
MG	MY.5G.00368	UBE2F	Belongs to the ubiquitin-conjugating enzyme family	Chr5:56098188 C>T	0.109375
MG	MY.5G.00378	PDS5A	Sister chromatid cohesion protein PDS5 homolog A	Chr5:57140579 T>G; Chr5:57153979 C>T; Chr5:57169100 C>T	0.359375; 0.40625; 0.34375
MG	MY.5G.00443	HERC6	HECT and RLD domain containing E3 ubiquitin protein ligase family member 6	Chr5:79854236 G>A	0.203125
MG	MY.5G.00503	CENPE	Centromere-associated protein E	Chr5:94425630 G>A	0.0625
MG	MY.5G.00526	TCEB1	Transcription elongation factor B polypeptide	Chr5:100334999 A>G	0.0625
MG	MY.5G.00561	PRDX1	Peroxiredoxin-1	Chr5:109440860 C>T	0.0625

MG	MY.6G.00079	SUSD1	Sushi domain-containing protein	Chr6:11462170 C>A	0.140625
MG	MY.6G.00083	C9orf84	C9orf84 homolog	Chr6:11789984 G>A; Chr6:11795350 C>T	0.080645; 0.177419
MG	MY.6G.00159	SECISBP2	SECIS binding protein 2	Chr6:23551437 T>C	1.56E-01
MG	MY.6G.00170	SPATA31E1	Domain of unknown function (DUF4599)	Chr6:26607804 A>G; Chr6:26625414 C>T	0.265625; 0.1
MG	MY.6G.00171	ACBD5	acyl-CoA-binding domain-containing protein 5	Chr6:26641497 G>A; Chr6:26641542 G>A	0.25; 0.28125
MG	MY.6G.00235	FAM205A	Family with sequence similarity 205, member A	Chr6:35933726 C>T	0.125
MG	MY.6G.00272	R3HCC1	R3H and coiled-coil domain-containing protein 1	Chr6:37418214 T>C	0.796875
MG	MY.6G.00290	PPP2R2A	peptidyl-serine dephosphorylation	Chr6:40630941 G>A; Chr6:40638879 G>A	0.096774; 0.078125
MG	MY.6G.00302	HTD2	Hydroxyacyl-thioester dehydratase type 2	Chr6:41734764 C>T	0.109375
MG	MY.6G.00303	PPFIA1	PTPRF interacting protein alpha 1	Chr6:41770873 C>T	0.109375
MG	MY.6G.00349	STX17	Syntaxin-17	Chr6:46836051 G>A	0.071429
MG	MY.6G.00376	ALDH1B1	Aldehyde dehydrogenase	Chr6:49427229 T>A; Chr6:49428209 G>A	0.078125; 0.0625
MG	MY.6G.00392	GAPDH	Key glycolytic enzyme that also participates in cytoskeletal organization, nuclear regulation, and translation control.	Chr6:50548334 T>A	0.0625
MG	MY.6G.00513	CSNK1E	Casein kinase	Chr6:69809825 C>A	0.0625
MG	MY.6G.00531	SRSF3	Serine arginine-rich splicing factor	Chr6:72879217 C>T	0.0625
MG	MY.6G.00567	CISD1	CDGSH iron sulfur domain 1	Chr6:85624478 C>T	0.109375
MG	MY.6G.00656	hnrnpa1	Heterogeneous nuclear ribonucleoprotein A1-like	Chr6:97179245 G>C	0.140625
MG	MY.6G.00658	LIMS1	LIM and senescent cell antigen-like-containing domain protein	Chr6:99613987 A>G	0.125
MG	MY.6G.00676	FBXO16	F-box only protein 16	Chr6:101848236 G>A	0.5
MG	MY.6G.00691	TDH	L-threonine 3-dehydrogenase, mitochondrial-like	Chr6:104158286 A>G	0.109375
MG	MY.6G.00716	AADAT	Kynurenine alpha-aminoadipate aminotransferase	Chr6:110124378 G>T	0.390625
MG	MY.6G.00721	Unknown	Unknown	Chr6:110289488 G>A	0.109375
MG	MY.7G.00025	RPS12	structural constituent of ribosome	Chr7:547020 T>C	0.234375
MG	MY.7G.00030	TTLL8	Tubulin tyrosine	Chr7:689324 C>T	0.078125

MG	MY.7G.00038	Unknown	Unknown	Chr7:1577408 C>T	0.625
MG	MY.7G.00039	Unknown	Endonuclease/Exonuclease/phosphatase family	Chr7:1710494 C>T	0.84375
MG	MY.7G.00042	CERK	Ceramide kinase	Chr7:3167130 G>A	0.517857
MG	MY.7G.00052	PPARA	Peroxisome proliferator-activated receptor alpha	Chr7:3719264 G>A	0.125
MG	MY.7G.00093	Unknown	Unknown	Chr7:6786445 C>T	0.0625
MG	MY.7G.00126	CHADL	Chondroadherin-like	Chr7:7691845 C>T	0.15625
MG	MY.7G.00128	EP300	E1A binding protein p300	Chr7:7742372 C>T	0.140625
MG	MY.7G.00152	NPTXR	Neuronal pentraxin receptor	Chr7:9627400 G>A	0.0625
MG	MY.7G.00195	COQ10B	Coenzyme Q-binding protein COQ10 homolog B	Chr7:11104034 C>T; Chr7:11104281 G>T	0.171875; 0.265625
MG	MY.7G.00201	CACNA2D4	Voltage-dependent calcium channel subunit alpha-2 delta-4	Chr7:11867900 G>A	0.125
MG	MY.7G.00292	Unknown	Scavenger receptor Cys-rich	Chr7:16926363 C>T	0.125
MG	MY.7G.00294	Unknown	Scavenger receptor Cys-rich	Chr7:16965273 C>T	0.28125
MG	MY.7G.00325	KLRF1	Killer cell lectin-like receptor subfamily F member	Chr7:18756826 C>T	0.0625
MG	MY.7G.00347	TAS2R8	Taste receptor, type 2, member	Chr7:19858355 T>C	0.09375
MG	MY.7G.00354	TAS2R42	sensory perception of taste	Chr7:19970959 C>T	0.078125
MG	MY.7G.00361	CREBL2	cAMP responsive element binding protein-like 2	Chr7:21219475 C>T; Chr7:21239504 T>G	0.074074; 0.109375
MG	MY.7G.00388	EEF1A1	Elongation factor	Chr7:24497712 T>A; Chr7:24498174 G>A	0.0625; 0.0625
MG	MY.7G.00391	SLC15A5	solute carrier family 15 member 5	Chr7:24892134 C>G	0.0625
MG	MY.7G.00400	PSMC6	26S protease regulatory subunit	Chr7:27319593 G>A; Chr7:27319719 G>A	0.5; 0.515625
MG	MY.7G.00405	Unknown	Unknown	Chr7:28280268 C>T	0.078125
MG	MY.7G.00449	SMCO2	Single-pass membrane protein with coiled-coil domains 2	Chr7:36055794 G>T	0.203125
MG	MY.7G.00506	APOL3	Apolipoprotein	Chr7:43188714 A>G	0.546875
MG	MY.7G.00542	KIAA1033	WASH complex subunit	Chr7:48962079 A>G	0.78125
MG	MY.7G.00608	Unknown	Unknown	Chr7:57969446 T>A	0.1875
MG	MY.7G.00613	OR6C3	Olfactory receptor	Chr7:58098335 G>A	0.140625

MG	MY.7G.00688	RBMS2	RNA binding motif single stranded interacting protein 2	Chr7:59505365 G>A	0.0625
MG	MY.7G.00755	CENPBD1	Tigger transposable element-derived protein 1-like	Chr7:66075111 T>C	0.484375
MG	MY.7G.00771	C12orf56	Chromosome 12 open reading frame 56	Chr7:67638705 T>C	0.375
MG	MY.7G.00799	LYZ	lysozyme activity	Chr7:71980642 A>G	0.078125
MG	MY.7G.00841	OCIAD1	OCIA domain-containing protein 1 isoform	Chr7:81333391 C>T	0.234375
MG	MY.7G.00848	EIF2S3	Eukaryotic translation initiation factor 2, subunit	Chr7:82712237 G>T; Chr7:82713683 C>T	0.71875; 0.125
MG	MY.7G.00926	TMPRSS12	Transmembrane protease serine 12	Chr7:86711309 T>C	0.1875
MG	MY.7G.00958	KRT86	structural molecule activity	Chr7:88142875 A>G	0.09375
MG	MY.7G.00960	KRT85	Keratin type II head	Chr7:88190517 G>A	0.0625
MG	MY.7G.01010	RPL19	5.8S rRNA binding	Chr7:89310849 T>C	0.140625
MG	MY.7G.01041	C3orf20	protein C3orf20 homolog	Chr7:90646750 C>T	0.0625
MG	MY.7G.01090	WTAP	Wilms tumor 1 associated protein	Chr7:104475808 G>A	0.0625
MG	MY.7G.01109	OSBPL8	Oxysterol-binding protein-related protein 8	Chr7:109989045 G>T	0.140625
MG	MY.7G.01126	DUSP14	Belongs to the protein-tyrosine phosphatase family. Non-receptor class dual specificity subfamily	Chr7:114513173 A>G	0.0625
MG	MY.8G.00117	FDPS	Farnesyl pyrophosphate synthase	Chr8:38869136 T>C	0.203125
MG	MY.8G.00139	TIMD4	T-cell immunoglobulin and mucin	Chr8:41881843 C>T; Chr8:41881874 A>G; Chr8:41932866 G>A	0.125; 0.078125; 0.375
MG	MY.8G.00142	ELP4	elongator acetyltransferase complex subunit 4	Chr8:42585637 G>A	0.09375
MG	MY.8G.00252	DIAPH1	Formin 1	Chr8:57653500 C>T	0.09375
MG	MY.8G.00479	OR2T11	Olfactory receptor	Chr8:67832908 A>G	0.125
MG	MY.8G.00504	Unknown	Class I histocompatibility antigen	Chr8:68719239 G>A	0.640625
MG	MY.8G.00635	P4HA2	procollagen-proline 4-dioxygenase activity	Chr8:86984172 C>A	0.3125
MG	MY.8G.00832	HMGN1	pyrimidine dimer repair by nucleotide-excision repair	Chr8:93253266 T>C	0.796875
MG	MY.8G.00874	THTPA	thiamine triphosphatase	Chr8:94280407 G>A	0.15625

MG	MY.8G.00908	Unknown	Zinc finger protein	Chr8:95184300 C>T	0.140625
MG	MY.8G.00952	CCDC130	Coiled-coil domain-containing protein 130	Chr8:96757214 G>A	1.72E-01
MG	MY.8G.01021	HIST3H3	Core histone H2A/H2B/H3/H4	Chr8:99948790 T>C	0.0625
MG	MY.8G.01022	TRIM17	Tripartite motif containing 17	Chr8:99984012 G>A	0.09375
MG	MY.8G.01050	MAU2	Cohesin loading factor	Chr8:101344684 C>T	0.140625
MG	MY.8G.01104	COLGALT1	Procollagen galactosyltransferase 1	Chr8:102947122 C>T	0.15625
MG	MY.8G.01158	OR10H4	serotonin receptor activity	Chr8:104561649 C>T	6.25E-02
MG	MY.8G.01170	CYP4F22	Belongs to the cytochrome P450 family	Chr8:104951511 A>G	1.72E-01
MG	MY.9G.00003	ZC3H6	Zinc finger CCCH domain-containing protein 6	Chr9:2808631 A>G	9.38E-02
MG	MY.9G.00037	ARID5A	AT-rich interactive domain-containing protein 5A	Chr9:5216035 G>C	7.81E-02
MG	MY.9G.00169	NLRC4	NLR family, CARD	Chr9:17537042 T>A	7.81E-02
MG	MY.9G.00200	ATL2	Atlastin GTPase 2	Chr9:23525867 T>A; Chr9:23556341 G>A; Chr9:23558095 C>T	0.234375; 0.078125; 0.109375
MG	MY.9G.00215	ST13	suppression of tumorigenicity 13 (colon carcinoma) (Hsp70 interacting protein)	Chr9:25029675 C>T	7.81E-02
MG	MY.9G.00297	AFG3L2	AFG3-like protein	Chr9:43206579 T>C	2.34E-01
MG	MY.9G.00304	Unknown	Unknown	Chr9:45947082 C>T	2.97E-01
MG	MY.9G.00322	GCC2	GRIP and coiled-coil domain containing 2	Chr9:47293609 A>T	2.03E-01
MG	MY.9G.00351	SRD5A1	3-oxo-5-alpha-steroid 4-dehydrogenase 1	Chr9:49575623 C>A; Chr9:49575905 G>A	0.125; 0.703125
MG	MY.9G.00371	REEP1	receptor accessory protein 1	Chr9:50796922 T>C	1.09E-01
MG	MY.9G.00375	GCSH	Glycine cleavage system H protein	Chr9:51298479 G>A	1.77E-01
MG	MY.9G.00403	NOP58	NOP58 ribonucleoprotein	Chr9:58297417 G>A; Chr9:58297747 G>A; Chr9:58298361 T>C	0.09375; 0.59375; 0.0625
MG	MY.9G.00433	Unknown	Unknown	Chr9:64915292 C>T	5.63E-01
MG	MY.9G.00446	Unknown	Unknown	Chr9:67976971 C>T	2.19E-01

MG	MY.9G.00460	ANTXR1	Anthrax toxin receptor	Chr9:69143709 C>G	7.81E-02
MG	MY.9G.00468	NANOG	homeobox	Chr9:69883693 C>G	8.75E-01
MG	MY.9G.00537	GAREML	regulator of	Chr9:74986068 A>C	3.13E-01
MG	MY.9G.00602	COX7B	Cytochrome c oxidase subunit 7B	Chr9:85357387 C>T	6.09E-01
MG	MY.9G.00603	Unknown	Unknown	Chr9:85851313 C>T	1.56E-01
MG	MY.9G.00617	ODC1	ornithine	Chr9:88794393 T>C	6.45E-02
MG	MY.9G.00804	POMT1	Protein O-mannosyl-transferase 1 isoform X1	Chr9:103015430 G>A	6.25E-02
MG	MY.9G.00809	RPS4X	ribosomal protein S4	Chr9:103575439 G>A	1.72E-01
MG	MY.9G.00826	PAEP	Lipocalin / cytosolic fatty-acid binding protein family	Chr9:104576161 G>A	1.61E-01
MG	MY.9G.00830	SOHLH1	Spermatogenesis- and oogenesis-specific basic helix-loop-helix-containing protein	Chr9:104635487 G>A	5.16E-01
MG	MY.9G.00850	ABO	Histo-blood group ABO system transferase 1-like	Chr9:105472432 A>T	7.81E-02
MG	MY.9G.00876	Unknown	Unknown	Chr9:106883229 C>T	0.0625
MG	MY.10G.00023	PRPF38A	factor 38A	Chr10:6992898 C>T	0.171875
MG	MY.10G.00035	ANKRD31	Ankyrin repeat domain 31	Chr10:8735205 A>G	0.109375
MG	MY.10G.00086	ZC3H14	Zinc finger CCCH domain-containing protein 14	Chr10:14254706 T>A	0.0625
MG	MY.10G.00231	TRDV1	Immunoglobulin V-Type	Chr10:24945972 G>A	0.125
MG	MY.10G.00232	TRAV22	Immunoglobulin V-set domain	Chr10:24996890 C>G	0.09375
MG	MY.10G.00240	TRAV18	Immunoglobulin V-set domain	Chr10:25455812 C>A	0.1875
MG	MY.10G.00393	SPRED1	Sprouty-related, EVH1 domain-containing protein 1	Chr10:33893527 G>T	0.109375
MG	MY.10G.00470	MSH6	DNA mismatch repair	Chr10:42652881 C>T	0.107143
MG	MY.10G.00504	PIF1	DNA helicase involved in maintaining mitochondrial and nuclear genome stability, resolving G-quadruplex structures, and regulating telomere maintenance.	Chr10:45394896 T>C	0.109375

MG	MY.10G.00546	RPL7	60S ribosomal protein	Chr10:50885779 C>T; Chr10:50885911 C>T	0.078125; 0.125
MG	MY.10G.00603	MRPL42	ribosomal protein L42	Chr10:59670100 T>C	0.46875
MG	MY.10G.00620	CEP152	Centrosomal protein	Chr10:61364844 A>T	0.09375
MG	MY.10G.00641	NONO	non-POU domain-containing octamer-binding protein	Chr10:65226743 C>T	0.390625
MG	MY.10G.00701	EEF1G	Elongation factor	Chr10:73574105 G>T	0.265625
MG	MY.10G.00709	CBFA2T2	NHR2 domain like	Chr10:75326239 G>A	0.515625
MG	MY.10G.00828	CSDE1	Cold shock	Chr10:86503095 T>C; Chr10:86503182 A>G	0.064516; 0.140625
MG	MY.10G.00832	FLVCR2	Feline leukemia virus subgroup C receptor-related protein	Chr10:86623354 G>A	0.421875
MG	MY.11G.00107	UQCRB	Cytochrome b-c1 complex subunit 7-like	Chr11:18220039 C>T	0.328125
MG	MY.11G.00128	HSPA8	Molecular chaperone involved in protein folding, refolding, degradation of misfolded proteins, and cellular stress responses.	Chr11:21554371 A>C	0.051724
MG	MY.11G.00190	LGALS3	Galectin	Chr11:39577661 T>C	0.140625
MG	MY.11G.00199	FADS2	Fatty acid desaturase 2	Chr11:43489419 C>G	0.109375
MG	MY.11G.00256	YTHDF3	YTH domain family	Chr11:53133844 C>G	0.078125
MG	MY.11G.00263	Unknown	Unknown	Chr11:54961038 A>G	0.921875
MG	MY.11G.00273	FAM110B	Family with sequence similarity 110 member B	Chr11:56914179 G>A; Chr11:56961087 G>A	0.203125; 0.09375
MG	MY.11G.00334	TRMT12	tRNA wybutosine-synthesizing protein 2 homolog	Chr11:65631509 G>C	0.09375
MG	MY.11G.00348	HIST1H2BB	nucleosome assembly	Chr11:68884173 C>T	0.078125
MG	MY.11G.00351	FAM49B	Family with sequence similarity 49, member B	Chr11:70275144 A>G	0.703125
MG	MY.11G.00353	rps25	ribosomal protein	Chr11:70893965 T>C	0.28125
MG	MY.11G.00372	ANKRD26	Ankyrin repeat domain-containing protein 26-like	Chr11:76397371 G>C	0.0625
MG	MY.11G.00376	TRAPPC9	trafficking protein particle complex	Chr11:77675877 G>A	0.28125

MG	MY.11G.00409	TOP1MT	I, mitochondrial	Chr11:79842395 A>G; Chr11:79845259 C>A	0.28125; 0.09375
MG	MY.11G.00479	PPFIA1	Sterile alpha motif.	Chr11:81013118 G>T	0.0625
MG	MY.12G.00028	RPA3OS	RPA3 opposite strand	Chr12:10171881 T>A	0.9375
MG	MY.12G.00069	PKIB	cAMP-dependent protein kinase inhibitor beta	Chr12:19177126 C>A	0.125
MG	MY.12G.00077	Unknown	Unknown	Chr12:21456574 G>T	0.078125
MG	MY.12G.00119	Unknown	Unknown	Chr12:28952205 T>A	0.078125
MG	MY.12G.00214	UTP14A	U3 small nucleolar RNA-associated protein 14 homolog	Chr12:49853953 G>C; Chr12:49854858 C>T	0.0625; 0.21875
MG	MY.12G.00236	CFAP206	UPF0704 protein C6orf165 homolog	Chr12:52990162 G>A	0.109375
MG	MY.12G.00330	Unknown	Unknown	Chr12:69217232 T>C	0.125
MG	MY.12G.00344	ZC2HC1B	Zinc finger, C2HC-type containing 1B	Chr12:71277424 A>G	0.234375
MG	MY.12G.00361	RPL35A	Ribosomal protein L35Ae	Chr12:75449935 C>T	0.109375
MG	MY.12G.00429	ACAT2	acetyl-CoA acetyltransferase	Chr12:85622671 T>C	0.28125
MG	MY.12G.00439	PLG	Serine protease involved in fibrinolysis, extracellular matrix degradation, tissue remodeling, and inflammation.	Chr12:86165382 C>T	0.0625
MG	MY.12G.00452	RPS6KA2	Ribosomal protein S6 kinase	Chr12:91263825 G>A	0.9375
MG	MY.12G.00457	TCP10	T-complex protein 10 C-terminus	Chr12:91491223 A>G	0.145161
MG	MY.12G.00462	KIF25	Belongs to the TRAFAC class myosin-kinesin ATPase superfamily. Kinesin family	Chr12:91954981 G>A	0.428571
MG	MY.13G.00029	Unknown	Sperm acrosome associated 7	Chr13:1692491 C>T	0.0625
MG	MY.13G.00048	Unknown	Unknown	Chr13:4893394 G>A; Chr13:4893420 C>T	0.359375; 0.78125
MG	MY.13G.00111	Unknown	Unknown	Chr13:29136198 T>A	0.125
MG	MY.13G.00146	Unknown	attachment of spindle microtubules to kinetochore involved in meiotic chromosome segregation	Chr13:44813726 G>A; Chr13:44826766 T>G; Chr13:44837556 C>T	0.109375; 0.25; 0.078125

MG	MY.13G.00254	Unknown	Unknown	Chr13:59221987 G>A; Chr13:59221999 C>A	0.703125; 0.375
MG	MY.13G.00255	Unknown	Unknown	Chr13:59322860 G>A	0.125
MG	MY.13G.00258	Unknown	Unknown	Chr13:59466500 T>C	0.109375
MG	MY.13G.00268	NEK5	Serine threonine-protein kinase	Chr13:60027214 G>A	0.1875
MG	MY.13G.00278	Gucy1b2	substituted 2 bases at 2 genomic stop codons	Chr13:61008582 T>A	0.125
MG	MY.13G.00303	CIR1	Corepressor interacting with RBPJ 1	Chr13:63744508 G>A	0.109375
MG	MY.13G.00317	COG3	oligomeric golgi complex	Chr13:65917379 C>A; Chr13:65933577 G>T	0.078125; 0.09375
MG	MY.13G.00324	NUFIP1	nuclear fragile X mental	Chr13:66397504 C>T	0.140625
MG	MY.13G.00352	PCDH8	Cadherin repeats.	Chr13:71071808 C>A	0.125
MG	MY.14G.00040	Unknown	Unknown	Chr14:6733036 C>A; Chr14:7029562 G>A	0.796875; 0.0625
MG	MY.14G.00085	PTI	Pancreatic trypsin inhibitor-like	Chr14:9376467 A>G	0.078125
MG	MY.14G.00151	KIAA1755	protein KIAA1755 homolog	Chr14:16262966 A>G	0.171875
MG	MY.14G.00190	STMN1	stathmin	Chr14:18572997 G>A	0.09375
MG	MY.14G.00200	MMP24	Domain of unknown function (DUF3377)	Chr14:18838655 C>G	0.625
MG	MY.14G.00240	BPIFB6	family B member 6	Chr14:21052154 C>T	0.109375
MG	MY.14G.00389	ABHD16B	Abhydrolase domain containing 16B	Chr14:29395304 G>A	0.078125
MG	MY.14G.00495	AKR1C4	Dihydrodiol dehydrogenase 3-like	Chr14:38910047 C>T	0.34375
MG	MY.14G.00532	NAPB	Attachment Protein	Chr14:40460479 C>T	0.453125
MG	MY.14G.00677	Unknown	Unknown	Chr14:64257885 C>T	0.890625
MG	MY.14G.00690	ANKRD26	Domain of unknown function (DUF3496)	Chr14:64859213 G>A; Chr14:64862754 G>T	0.4375; 0.083333
MG	MY.14G.00717	NINL	ninein-like	Chr14:71296856 C>T; Chr14:71317464 C>T	0.09375; 0.21875

MG	MY.14G.00722	Unknown	Unknown	Chr14:71582449 A>G	0.234375
MG	MY.15G.00027	TIA1	TIA1 cytotoxic granule associated RNA binding protein	Chr15:9979800 C>G	0.078125
MG	MY.15G.00053	LDHB	dehydrogenase B	Chr15:14553350 A>G	0.15625
MG	MY.15G.00073	DLAT	acetyltransferase component of pyruvate dehydrogenase complex	Chr15:20306347 G>A	0.234375
MG	MY.15G.00096	Unknown	Unknown	Chr15:25234049 G>A	0.171875
MG	MY.15G.00099	Unknown	attachment of spindle microtubules to kinetochore involved in meiotic chromosome segregation	Chr15:25727294 G>A	0.1875
MG	MY.15G.00119	IL18	Augments natural killer cell activity in spleen cells and stimulates interferon gamma production in T-helper type I cells	Chr15:26449441 C>A	0.09375
MG	MY.15G.00129	TMPRSS5	Transmembrane protease, serine 5	Chr15:28216736 T>A; Chr15:28220315 C>G	0.078125; 0.609375
MG	MY.15G.00144	CADM1	Cell adhesion molecule 1	Chr15:29833441 T>G	0.109375
MG	MY.15G.00227	UBASH3B	Ubiquitin-associated and SH3 domain-containing protein	Chr15:37343725 C>T	0.375
MG	MY.15G.00229	C11orf63	protein C11orf63 homolog	Chr15:37556168 G>C	6.25E-02
MG	MY.15G.00401	Olfr639	Serpentine type 7TM GPCR chemoreceptor Srsx	Chr15:50996339 G>T	3.71E-01
MG	MY.15G.00531	OR2AT4	Olfactory receptor	Chr15:55329710 C>T	2.19E-01
MG	MY.15G.00554	GUCY2F	guanylate cyclase activity	Chr15:57069263 T>G; Chr15:57080887 C>T; Chr15:57082475 C>A	0.109375; 0.21875; 0.15625
MG	MY.15G.00626	ATMIN	ATM interactor	Chr15:68808234 C>G	1.56E-01
MG	MY.15G.00632	Unknown	Gag protein	Chr15:73354351 G>A	1.72E-01
MG	MY.15G.00633	Unknown	Unknown	Chr15:73552760 C>T; Chr15:73600759 G>A	0.0625; 0.125
MG	MY.15G.00651	SLC35C1	GDP-fucose transporter 1	Chr15:76350561 G>A	1.88E-01
MG	MY.15G.00668	LRP4	lipoprotein receptor-related protein 4	Chr15:77305385 C>G	6.25E-02

MG	MY.15G.00691	FNBP4	Formin-binding protein 4	Chr15:78017714 C>A	6.25E-02
MG	MY.15G.00716	OR5AP2	Olfactory receptor	Chr15:78850766 T>G	1.09E-01
MG	MY.16G.00010	RNPEP	Leukotriene A4 hydrolase, C-terminal	Chr16:421334 C>T	7.81E-02
MG	MY.16G.00015	PTPRB	Protein tyrosine phosphatase, catalytic domain	Chr16:532177 C>T	1.94E-01
MG	MY.16G.00027	IGFN1	Immunoglobulin-like and fibronectin type III	Chr16:1105726 A>G	3.75E-01
MG	MY.16G.00063	Unknown	Unknown	Chr16:6564435 C>T; Chr16:6589588 G>A	0.078125; 0.078125
MG	MY.16G.00201	PRAMEF12	Melanoma antigen preferentially expressed in tumors-like	Chr16:25871897 G>A	6.25E-02
MG	MY.16G.00206	TMEM51	transmembrane protein 51	Chr16:27765280 A>G	9.38E-02
MG	MY.16G.00317	PER3	Period circadian	Chr16:34409209 T>C; Chr16:34471557 C>T	0.09375; 0.09375
MG	MY.16G.00430	RABIF	Guanine nucleotide exchange factor	Chr16:47145023 C>A	1.13E-01
MG	MY.16G.00441	C1orf101	Cation channel sperm-associated protein subunit delta	Chr16:49272204 A>G	1.72E-01
MG	MY.16G.00468	WDR26	C-terminal to LisH motif.	Chr16:52334576 G>C	0.140625
MG	MY.16G.00521	TRA2B	Transformer-2 protein homolog	Chr16:70691818 G>C; Chr16:70691895 T>A	0.078125; 0.125
MG	MY.16G.00565	TMCC2	transmembrane and coiled-coil domain family 2	Chr16:75253173 T>C	0.15625
MG	MY.16G.00573	PTP4A1	protein tyrosine phosphatase type IVA	Chr16:76065438 C>T	0.09375
MG	MY.17G.00066	RPS3	ribosomal protein	Chr17:17418463 C>T	0.078125
MG	MY.17G.00073	Unknown	Unknown	Chr17:22143587 T>C	0.0625
MG	MY.17G.00076	CCT6A	chaperonin containing TCP1, subunit	Chr17:27279754 A>T	0.0625
MG	MY.17G.00083	Unknown	Unknown	Chr17:30324009 A>G	0.12069
MG	MY.17G.00094	SLC45A2	Solute carrier family 45 member 2	Chr17:32247688 A>C	0.078125
MG	MY.17G.00118	C5orf42	Chromosome 5 open reading frame 42	Chr17:34841064 G>A	0.1875
MG	MY.17G.00120	HMGN1	Non-histone chromosomal protein	Chr17:34958481 C>T	0.078125
MG	MY.17G.00220	IPO11	ribosomal protein import into nucleus	Chr17:54976610 A>G	0.203125

MG	MY.17G.00301	RPL26L1	ribosomal protein L26-like 1	Chr17:67176356 G>A	0.0625
MG	MY.18G.00005	SNRPN	Small nuclear	Chr18:6965853 T>C	0.1875
MG	MY.18G.00091	FES	Tyrosine-protein kinase Fes Fps	Chr18:26856476 C>T	0.171875
MG	MY.18G.00127	MYADM	myeloid-associated differentiation marker-like	Chr18:28417783 C>T	0.203125
MG	MY.18G.00134	PPFIA1	Protein tyrosine phosphatase, receptor type, f polypeptide (PTPRF), interacting protein (liprin), alpha 1	Chr18:28615287 C>T; Chr18:28620723 G>C	0.09375; 0.068965
MG	MY.18G.00137	MYADM	myeloid-associated differentiation marker-like	Chr18:28685452 A>G	0.09375
MG	MY.18G.00140	MYADM	myeloid-associated differentiation	Chr18:28718071 T>A	0.1875
MG	MY.18G.00212	HIGD1A	HIG1 domain family member	Chr18:36515036 A>G	0.0625
MG	MY.18G.00222	Unknown	Endonuclease/Exonuclease/phosphatase family	Chr18:37860147 G>A	0.0625
MG	MY.18G.00236	Unknown	SEA domain	Chr18:38612455 G>T	0.0625
MG	MY.18G.00317	PPP1CC	Serine threonine-protein phosphatase PP1-gamma catalytic	Chr18:52322890 C>T; Chr18:52323274 G>A	0.0625; 0.0625
MG	MY.18G.00410	TCL1B	T-cell leukemia lymphoma	Chr18:64465182 G>A	0.828125
MG	MY.18G.00421	Unknown	Unknown	Chr18:68093545 C>T; Chr18:68113311 C>T; Chr18:68122392 T>G	0.09375; 0.078125; 0.078125
MG	MY.18G.00462	Unknown	Unknown	Chr18:71866589 C>T	0.109375
MG	MY.19G.00026	Unknown	Unknown	Chr19:6782712 T>C	0.078125
MG	MY.19G.00072	EIF3M	Eukaryotic translation initiation factor 3, subunit	Chr19:17230309 A>G	0.140625
MG	MY.19G.00127	Unknown	Unknown	Chr19:35821747 A>G	0.203125
MG	MY.19G.00140	RPL19	ribosomal protein	Chr19:37454143 C>T	0.1875
MG	MY.19G.00206	Unknown	Unknown	Chr19:50701206 G>C; Chr19:50732747 C>T	0.0625; 0.359375
MG	MY.19G.00285	ACAD10	Acyl-CoA dehydrogenase family member 10	Chr19:55811649 C>T; Chr19:55834143 C>T	0.265625; 0.390625

MG	MY.19G.00286	ACAD10	Acyl-CoA dehydrogenase family member	Chr19:55893464 C>T	0.109375
MG	MY.19G.00299	KSR2	Kinase suppressor of Ras 2	Chr19:58026267 G>A	0.15625
MG	MY.19G.00406	MYO18B	unconventional	Chr19:65965949 T>C	0.83871
MG	MY.19G.00530	TMEM191B	TMEM191C family	Chr19:72017151 C>T	0.0625
MG	MY.19G.00532	SERPIND1	Heparin cofactor	Chr19:72044965 A>G	0.3125
MG	MY.20G.00024	Unknown	krueppel associated box	Chr20:640266 G>A	0.484375
MG	MY.20G.00083	OSCAR	immunoglobulin-like receptor	Chr20:2224605 C>T	0.71875
MG	MY.20G.00104	TPRX1	DNA-binding transcription factor activity, RNA polymerase II-specific	Chr20:2553346 C>T	0.359375
MG	MY.20G.00119	PTPRH	Protein tyrosine phosphatase, receptor type, H	Chr20:2824259 G>A	0.078125
MG	MY.20G.00162	UBQLN4	Heat shock chaperonin-binding motif.	Chr20:3627640 A>G	0.0625
MG	MY.20G.00169	AU018091	Cationic amino acid transporter 3-like	Chr20:3803218 C>A	0.0625
MG	MY.20G.00170	AU018091	Cationic amino acid transporter 3-like	Chr20:3813762 G>A	0.080645
MG	MY.20G.00188	ZNF160	Zinc finger protein	Chr20:4837228 G>A	0.109375
MG	MY.20G.00204	Unknown	C2H2-type zinc finger	Chr20:5593929 C>T	0.109375
MG	MY.20G.00224	CD33	Myeloid cell surface antigen	Chr20:6147491 C>T	0.0625
MG	MY.20G.00481	Unknown	Seminal plasma protein	Chr20:11631623 C>G	0.078125
MG	MY.20G.00544	HIPK4	kinase 4	Chr20:13294531 C>A	0.640625
MG	MY.20G.00581	FBXO27	F-box only protein	Chr20:14187643 T>C	0.09375
MG	MY.20G.00582	FBXO27	F-box only protein 27-like	Chr20:14220848 T>A; Chr20:14236775 G>A	0.0625; 0.09375
MG	MY.20G.00664	EI24	Etoposide-induced protein 2.4 homolog	Chr20:16553963 C>T	0.09375
MG	MY.20G.00747	HYDIN	HYDIN, axonemal central pair apparatus protein	Chr20:22504029 A>T	0.0625
MG	MY.20G.00748	TCEAL8	transcription elongation factor A	Chr20:22734226 A>G	0.171875

MG	MY.20G.00877	CNOT1	positive regulation of cytoplasmic mRNA processing body assembly	Chr20:36115628 G>A	0.05
MG	MY.20G.01004	Unknown	Unknown	Chr20:48193783 C>T	0.078125
MG	MY.20G.01030	SFR1	Swi5-dependent recombination DNA repair protein 1 homolog	Chr20:49533965 G>A	0.21875
MG	MY.20G.01046	RPL5	ribosomal protein	Chr20:51454502 C>T	0.0625
MG	MY.20G.01050	TLDC1	TLD domain-containing protein	Chr20:51672297 C>T	0.109375
MG	MY.21G.00093	FBF1	factor 1	Chr21:7509859 C>A; Chr21:7517139 C>T	0.171875; 0.1875
MG	MY.21G.00190	RPS12	Belongs to the eukaryotic ribosomal protein eS12 family	Chr21:12072269 T>C	0.59375
MG	MY.21G.00393	DHX58	DEXH (Asp-Glu-X-His) box polypeptide 58	Chr21:20064798 T>C	0.09375
MG	MY.21G.00601	SPAG9	Sperm associated antigen 9	Chr21:26298652 C>A	0.0625
MG	MY.21G.00652	Unknown	Unknown	Chr21:28295537 C>T	0.125
MG	MY.21G.00712	RNF222	RING finger protein 222	Chr21:34037430 A>T; Chr21:34037610 G>T	0.3125; 0.140625
MG	MY.21G.00933	NUFIP2	Nuclear fragile X mental retardation-interacting protein 2	Chr21:41270341 C>T	0.078125
MG	MY.21G.00976	WSB1	WD repeat and SOCS	Chr21:42866864 A>T	0.0625
MG	MY.21G.01048	MRM1	rRNA methyltransferase 1	Chr21:48899481 T>C	0.16129
MG	MY.21G.01088	RNF43	E3 ubiquitin-protein ligase	Chr21:52497386 T>C	0.21875
MG	MY.21G.01094	OR4D2	Olfactory receptor 4D2-like	Chr21:52752312 G>A	0.078125
MG	MY.21G.01106	OSTC	Oligosaccharyltransferase complex subunit	Chr21:53227586 C>A	0.203125
MG	MY.21G.01123	PCTP	Phosphatidylcholine transfer protein-like	Chr21:55464243 C>A	0.078125
MG	MY.21G.01135	BCL9	B-cell CLL lymphoma 9	Chr21:57071208 C>G	0.078125
MG	MY.22G.00001	CCDC37	Cilia and flagella associated protein 100	Chr22:94320 A>T	0.0625
MG	MY.22G.00029	COPG1	Component of the coatomer complex involved in Golgi vesicle formation and retrograde protein transport from Golgi to the ER.	Chr22:1597802 A>G	0.765625
MG	MY.22G.00036	KIAA1257	Uncharacterized protein FLJ43738-like	Chr22:1760590 C>T	0.064516

MG	MY.22G.00041	Unknown	Unknown	Chr22:1978910 G>A	0.109375
MG	MY.22G.00081	TRH	Regulates TSH biosynthesis and functions as a neurotransmitter and neuromodulator in the nervous system.	Chr22:4718921 C>A	0.516129
MG	MY.22G.00119	ALS2CL	ALS2 C-terminal-like protein	Chr22:7826294 C>T	0.0625
MG	MY.22G.00131	KIF9	Kinesin-like protein KIF9	Chr22:8254265 A>T	0.203125
MG	MY.22G.00163	WDR6	WD repeat-containing protein 6	Chr22:9592892 C>A	0.125
MG	MY.22G.00289	Unknown	Unknown	Chr22:17371110 C>T	0.09375
MG	MY.22G.00295	PDHB	Pyruvate dehydrogenase E1 component subunit	Chr22:17578358 A>G	0.0625
MG	MY.22G.00313	C3orf49	Chromosome 3 open reading frame 49	Chr22:22829746 C>G	0.0625
MG	MY.22G.00323	HMGB1	high mobility group	Chr22:25188556 C>T	0.109375
MG	MY.22G.00526	TNFRSF10A	Tumor necrosis factor receptor superfamily member	Chr22:60791075 A>G	0.078125
MG	MY.23G.00029	DNAJA1	homolog, subfamily A, member	Chr23:5363324 T>A; Chr23:5398597 C>T	0.0625; 0.578125
MG	MY.23G.00033	C18orf63	protein C18orf63 homolog	Chr23:5651826 G>A	0.0625
MG	MY.23G.00042	Unknown	Unknown	Chr23:8674754 G>A	0.265625
MG	MY.23G.00064	Unknown	Unknown	Chr23:20695037 G>A; Chr23:20723166 G>A	0.15625; 0.4375
MG	MY.23G.00068	Unknown	Unknown	Chr23:20833596 G>A	0.296875
MG	MY.23G.00127	C18orf8	protein C18orf8 homolog	Chr23:34456950 G>A	0.078125
MG	MY.23G.00181	MTCL1	Microtubule crosslinking factor 1	Chr23:42775884 G>T	0.0625
MG	MY.23G.00255	Unknown	Unknown	Chr23:54574961 T>C	0.140625
MG	MY.23G.00303	SERPINB13	Serpin B13	Chr23:62609099 C>T	0.296875
MG	MY.24G.00015	TOMM20	mitochondrial outer membrane translocase complex assembly	Chr24:10521793 C>T	0.140625
MG	MY.24G.00250	DCLRE1A	DNA cross-link repair 1A	Chr24:41605645 G>A	0.0625
MG	MY.24G.00257	ABLIM1	Actin binding LIM protein 1	Chr24:42366976 G>A	0.0625
MG	MY.24G.00264	PNLIPRP1	triglyceride lipase activity	Chr24:44257242 C>T	0.09375

MG	MY.24G.00267	PNLIPRP1	Pancreatic lipase-related protein 1	Chr24:44262459 G>A; Chr24:44264640 C>T	0.484375; 0.421875
MG	MY.24G.00299	WDR11	WD repeat domain 11	Chr24:48138477 G>A	0.203125
MG	MY.24G.00331	OVCA2	Ovarian tumor suppressor candidate 2	Chr24:51823223 C>T	0.0625
MG	MY.24G.00362	CFAP46	Cilia and flagella associated protein 46	Chr24:58391117 G>A	0.109375
MG	MY.25G.00059	Unknown	N-acetyllactosaminide beta-1,6-N-acetylglucosaminyl-transferase, isoform C-like	Chr25:6707800 G>A	0.140625
MG	MY.25G.00061	Unknown	Unknown	Chr25:6768109 C>T	0.09375
MG	MY.25G.00113	PRL	Prolactin-related protein	Chr25:16283420 C>T	0.109375
MG	MY.25G.00141	BLVRA	Biliverdin reductase A	Chr25:19324724 T>C	0.625
MG	MY.25G.00201	Olfr1361	Olfactory receptor	Chr25:21103586 C>T	4.22E-01
MG	MY.25G.00257	Unknown	Olfactory receptor	Chr25:22248577 G>A	7.81E-02
MG	MY.25G.00276	Unknown	BOLA class I histocompatibility antigen, alpha chain	Chr25:22693743 A>C	7.81E-02
MG	MY.25G.00277	Unknown	BOLA class I histocompatibility antigen, alpha chain	Chr25:22723574 T>C	2.03E-01
MG	MY.25G.00278	Unknown	BOLA class I histocompatibility antigen, alpha chain	Chr25:22769644 T>C	6.25E-02
MG	MY.25G.00311	Unknown	Belongs to the MHC class I family	Chr25:23428137 G>A	9.38E-02
MG	MY.25G.00357	C4A	complement	Chr25:23865392 G>A; Chr25:23867072 T>C	0.21875; 0.096774
MG	MY.25G.00372	Unknown	Butyrophilin subfamily 1 member A1-like	Chr25:24066332 C>G	2.34E-01
MG	MY.25G.00373	C6orf10	Chromosome 6 open reading frame 10	Chr25:24305940 C>A	9.38E-02
MG	MY.25G.00449	CAPN11	Calpain-11	Chr25:32250467 G>A; Chr25:32253218 A>G	0.359375; 0.34375
MG	MY.25G.00636	Unknown	Belongs to the MHC class II family	Chr25:42787031 C>T	9.38E-02
MG	MY.25G.00651	MYL12B	EF-hand domain	Chr25:46159847 G>A	6.25E-02

MG	MY.25G.00653	DST	Dystonin	Chr25:46368355 G>A; Chr25:46711846 A>G	0.125; 0.671875
MG	MY.26G.00015	IFITM1	Interferon-induced transmembrane protein	Chr26:208703 A>C	3.39E-01
MG	MY.26G.00051	PSME1	Proteasome activator complex subunit 1	Chr26:802081 T>C	1.09E-01
MG	MY.26G.00065	PRR33	Domain of unknown function (DUF4643)	Chr26:1559962 T>C	0.078125
MG	MY.26G.00069	Unknown	Unknown	Chr26:1673225 C>T	0.0625
MG	MY.26G.00085	CARS	cysteinyl-tRNA synthetase	Chr26:2552608 C>A	0.296875
MG	MY.26G.00109	MRGPRD	Mas-related G-protein coupled receptor member D-like	Chr26:4741973 T>C	0.0625
MG	MY.26G.00158	SPTBN2	Spectrin beta chain, non-erythrocytic 2	Chr26:6325826 C>T	0.109375
MG	MY.26G.00249	SLC22A11	solute carrier family 22	Chr26:8204330 C>T	0.53125
MG	MY.26G.00289	SLC22A10	solute carrier family 22, member	Chr26:9432423 C>T	0.109375
MG	MY.26G.00364	Pga5	Belongs to the peptidase A1 family	Chr26:11762108 T>G	0.125
MG	MY.26G.00370	Pga5	Belongs to the peptidase A1 family	Chr26:11979431 C>G	0.09375
MG	MY.26G.00386	Unknown	Unknown	Chr26:12770060 T>C	0.484375
MG	MY.26G.00523	EEF1A1	Elongation factor	Chr26:29339995 C>T	0.171875
MG	MY.26G.00542	GGTA1	N-acetyllactosaminide alpha-1,3-galactosyltransferase-like	Chr26:35062997 G>A	0.0625
MG	MY.26G.00587	PRAMEF12	PRAME family member	Chr26:42189404 C>T	0.09375
MG	MY.26G.00588	UBTFL1	upstream-binding factor 1-like protein	Chr26:42193939 C>T	0.0625
MG	MY.27G.00014	ZFAND4	Zinc finger AN1-type	Chr27:1541406 C>T	0.15625
MG	MY.27G.00035	PTPN20B	Tyrosine-protein phosphatase	Chr27:3124508 G>A; Chr27:3137567 C>A	0.0625; 0.09375
MG	MY.27G.00042	EIF1B	translation initiation factor activity	Chr27:3561793 C>T	0.112903
MG	MY.27G.00069	SH2D4B	SH2 domain containing 4B	Chr27:9412447 C>T	0.125
MG	MY.27G.00081	TMEM254	Transmembrane protein 254	Chr27:9822434 A>G	0.671875
MG	MY.27G.00103	RPS8	ribosomal protein	Chr27:14636570 A>T	0.078125
MG	MY.27G.00138	CHST3	sulfotransferase 3	Chr27:16859871 C>T	0.09375

MG	MY.27G.00232	RPS15A	ribosomal protein	Chr27:31204513 C>T	0.109375
MG	MY.27G.00261	GTF2I	General transcription factor II-I	Chr27:36958506 C>T	0.16129
MG	MY.28G.00009	Unknown	Unknown	Chr28:5704033 A>G	0.125
MG	MY.28G.00050	NCAPH2	complex subunit H2	Chr28:17037978 T>A; Chr28:17038086 T>C	0.1875; 0.21875
MG	MY.28G.00117	RPLP1	60S acidic ribosomal protein P1-like	Chr28:28197907 A>G	0.375
MG	MY.28G.00129	RPL19	ribosomal protein	Chr28:29413555 A>C	0.140625
MG	MY.28G.00130	GSR	Belongs to the class-I pyridine nucleotide-disulfide oxidoreductase family	Chr28:29464655 G>A	0.0625
MG	MY.28G.00135	WRN	Werner syndrome	Chr28:29763100 T>C	0.833333
MG	MY.28G.00144	RNF122	RING finger protein 122	Chr28:31793270 G>A	0.203125
MG	MY.28G.00181	ADAM18	Disintegrin and metalloproteinase domain-containing protein	Chr28:37522137 C>A	0.078125
MG	MY.28G.00203	KIAA1191	monooxygenase p33MONOX	Chr28:40155289 C>A	0.125
MG	MY.29G.00009	Unknown	Olfactory receptor 1013-like	Chr29:4669481 G>A	0.103448
MG	MY.29G.00014	GJC1	gap junction channel activity involved in AV node cell-bundle of His cell electrical coupling	Chr29:4799073 T>G; Chr29:4799153 G>A	0.0625; 0.125
MG	MY.29G.00054	METRN	Meteorin, glial cell differentiation regulator	Chr29:5970959 A>G	0.064516
MG	MY.29G.00156	IL32	Interleukin 32	Chr29:7830029 C>T; Chr29:7830039 G>A	0.109375; 0.09375
MG	MY.29G.00162	OR1F1	Olfactory receptor	Chr29:7963354 G>C	0.25
MG	MY.29G.00169	ZNF75A	Zinc finger protein	Chr29:8078799 G>A	0.109375
MG	MY.29G.00172	ZNF597	Zinc finger protein	Chr29:8158221 A>T; Chr29:8158393 C>T	0.15625; 0.078125
MG	MY.29G.00208	Unknown	Ectonucleotide pyrophosphatase phosphodiesterase family member 7-like	Chr29:9447609 G>A	0.109375
MG	MY.29G.00241	RPS3A	40S ribosomal protein	Chr29:15961324 G>A	0.09375
MG	MY.29G.00277	Unknown	Unknown	Chr29:22143402 G>A	0.140625

MG	MY.29G.00308	ABCA3	ABC-2 family transporter protein	Chr29:24792327 A>G	0.875
MG	MY.29G.00544	MOGAT3	2-acylglycerol O-acyltransferase	Chr29:40437451 A>G; Chr29:40438750 G>A	0.9375; 0.15625
MG	MY.29G.00548	MUC12	Mucin 12, cell surface associated	Chr29:40633799 C>A	0.62963
MG	MY.29G.00582	Unknown	Neurexophilin	Chr29:41234653 G>A	0.09375
MG	MY.29G.00583	PILRA	Paired immunoglobulin-like type 2 receptor	Chr29:41276768 G>A	0.09375
MG	MY.29G.00605	FAM200A	Family with sequence similarity 200, member A	Chr29:41835753 C>T	0.171875
MG	MY.29G.00616	ARPC1A	Component of the Arp2/3 complex involved in actin polymerization and formation of branched actin networks.	Chr29:42054373 G>A	0.125
MG	MY.29G.00672	KIF19	Belongs to the TRAFAC class myosin-kinesin ATPase superfamily. Kinesin family	Chr29:45785724 C>T; Chr29:45796086 A>T	0.171875; 0.0625
MG	MY.29G.00677	MAD1L1	Mitotic spindle assembly checkpoint protein	Chr29:46109159 G>A	0.1875
MG	MY.29G.00683	TMEM184A	transmembrane protein 184A	Chr29:46316895 T>C	0.109375
PG	PY.1G.00030	SYNJ1	Synaptojanin 1	Chr1:3366784 C>T	0.337209
PG	PY.1G.00133	CPSF6	cleavage and polyadenylation specificity factor subunit	Chr1:42236573 C>T	0.44186
PG	PY.1G.00196	FTH1	Iron-storage protein involved in maintaining cellular iron homeostasis and preventing iron toxicity.	Chr1:53127397 T>G	0.058139
PG	PY.1G.00235	CD200R1	Cell surface glycoprotein CD200 receptor	Chr1:59627605 G>A	0.44186
PG	PY.1G.00330	MUC4	Mucin 4, cell surface associated	Chr1:72587788 C>T	0.534884
PG	PY.1G.00387	CLDN16	Tight junction protein involved in calcium-independent cell adhesion and sealing of the intercellular space.	Chr1:79058733 A>T	0.116279
PG	PY.1G.00416	MRPL50	ribosomal protein L50	Chr1:82873745 C>T	0.104651
PG	PY.1G.00432	Unknown	Unknown	Chr1:84676447 G>C	0.139535
PG	PY.1G.00505	TNFSF10	Tumor necrosis factor (ligand) superfamily, member 10	Chr1:97269116 G>T	0.139535
PG	PY.1G.00569	HNRNPA3	Heterogeneous nuclear ribonucleoprotein	Chr1:111377144 C>T	0.234375

PG	PY.1G.00599	AADAC	Arylacetamide	Chr1:118434948 C>A	0.104651
PG	PY.1G.00664	TPT1	negative regulation of ectoderm development	Chr1:129918571 C>T	0.186047
PG	PY.1G.00680	Unknown	Unknown	Chr1:132720178 C>T	0.430233
PG	PY.2G.00035	Unknown	Unknown	Chr2:2800797 A>C	0.151163
PG	PY.2G.00074	ALPL	alkaline phosphatase	Chr2:5324735 C>T	0.127907
PG	PY.2G.00208	EPB41	erythrocyte membrane protein band 4.1	Chr2:12070920 G>T	0.116279
PG	PY.2G.00230	TMEM39B	transmembrane protein 39B	Chr2:14860688 T>C	0.360465
PG	PY.2G.00355	Unknown	Unknown	Chr2:27897629 C>T	0.116279
PG	PY.2G.00381	SLC23A3	Solute carrier family 23 member 3	Chr2:29392242 A>T	0.093023
PG	PY.2G.00447	MSL3	Male-specific lethal 3 homolog	Chr2:40066960 G>A	0.27907
PG	PY.2G.00488	CCT6A	chaperonin containing TCP1, subunit	Chr2:45482627 C>T	0.267442
PG	PY.2G.00538	ACTR3	negative regulation of bleb assembly	Chr2:51284716 G>A	0.337209
PG	PY.2G.00566	AS3MT	Arsenite methyltransferase	Chr2:63478209 A>G	1.40E-01
PG	PY.2G.00684	PLAA	Phospholipase A2-activating protein	Chr2:100135440 A>G	1.05E-01
PG	PY.2G.00689	MAP3K7	Mitogen-activated protein kinase kinase kinase	Chr2:101057016 C>T	0.255814
PG	PY.2G.00752	METTL8	Methyltransferase like 8	Chr2:112813054 A>G	0.127907
PG	PY.2G.00759	SLC25A12	Solute carrier family 25	Chr2:113268186 C>T	0.197674
PG	PY.3G.00049	DUSP12	dual specificity	Chr3:10997160 A>G	0.081395
PG	PY.3G.00080	CCM2	Cerebral cavernous malformation 2	Chr3:11742914 G>A	0.372093
PG	PY.3G.00084	LY9	T-lymphocyte surface antigen	Chr3:11890124 C>T	0.162791
PG	PY.3G.00114	CRP	C-reactive	Chr3:13121726 G>T	0.069767
PG	PY.3G.00140	OR10K1	Olfactory receptor	Chr3:14518826 A>G	0.348837
PG	PY.3G.00190	RHBG	Rh family B glycoprotein	Chr3:17259357 C>T	0.104651
PG	PY.3G.00212	GON4L	GON-4-like	Chr3:17710792 C>T; Chr3:17729841 C>T	0.290698; 0.27907
PG	PY.3G.00268	CRTC2	transcription coactivator 2	Chr3:19300315 T>A	1.34E-01

PG	PY.3G.00600	GAPDH	Key glycolytic enzyme with additional roles in cytoskeletal organization, nuclear regulation, and translational control.	Chr3:42778578 A>G	5.81E-02
PG	PY.3G.00623	ARID3A	AT-rich interactive domain-containing protein 3A	Chr3:47913048 A>G	0.488372
PG	PY.3G.00671	PLEKHA1	Pleckstrin homology domain-containing family A member 1 isoform	Chr3:56011753 G>A	0.325581
PG	PY.3G.00709	hnrnpa1	Heterogeneous nuclear ribonucleoprotein A1-like	Chr3:61415953 G>A	0.104651
PG	PY.3G.00729	AHSA1	AHA1,activator of heat shock 90kDa protein ATPase homolog 1	Chr3:63615344 G>A; Chr3:63615563 G>A	0.151163; 0.186047
PG	PY.3G.00753	FAM73A	Family with sequence similarity 73, member A	Chr3:71018488 G>C	0.337209
PG	PY.3G.00821	JAK1	Tyrosine-protein kinase JAK1	Chr3:84681770 A>G	0.267442
PG	PY.3G.00844	C1orf87	Chromosome 1 open reading frame 87	Chr3:89944693 T>C	0.714286
PG	PY.3G.00955	NACA	Nascent polypeptide-associated complex subunit alpha-like	Chr3:103550033 A>G	0.406977
PG	PY.3G.01018	ST3GAL3	ST3 beta-galactoside alpha-2,3-sialyltransferase 3	Chr3:106365015 G>A	1.63E-01
PG	PY.3G.01040	ERMAP	membrane-associated protein	Chr3:107439086 A>G	1.16E-01
PG	PY.3G.01119	Unknown	Unknown	Chr3:113152611 A>T	2.09E-01
PG	PY.3G.01120	Unknown	Unknown	Chr3:113196416 G>A	1.63E-01
PG	PY.3G.01201	ESPNL	Espin-like	Chr3:121614042 C>T	1.28E-01
PG	PY.4G.00031	Unknown	Unknown	Chr4:13720263 T>C	1.63E-01
PG	PY.4G.00130	FNDC9	fibronectin type III	Chr4:41275946 T>C	0.104651
PG	PY.4G.00139	TIMD4	T-cell immunoglobulin and mucin	Chr4:41944001 G>A	0.476744
PG	PY.4G.00210	MAPRE2	microtubule-associated protein, RP EB family, member 2	Chr4:50615481 G>T	0.430233
PG	PY.4G.00478	OR2T11	Olfactory receptor	Chr4:67907105 A>G	0.22093
PG	PY.4G.00500	OR2G2	olfactory receptor activity	Chr4:68594339 G>A	0.127907
PG	PY.4G.00510	Unknown	Class I histocompatibility antigen	Chr4:68993599 G>A	0.581395
PG	PY.4G.00790	CD97	EGF-like module-containing mucin-like hormone receptor-like	Chr4:92104885 G>T	0.186047
PG	PY.4G.00893	ICAM1	Intercellular adhesion	Chr4:94634612 C>T; Chr4:94636116 G>A	0.744186; 0.232558

PG	PY.4G.00967	CCDC130	Coiled-coil domain-containing protein 130	Chr4:97045436 G>A	0.058139
PG	PY.4G.00989	DDX39A	ATP-dependent RNA helicase DDX39A	Chr4:97588625 G>A	0.069767
PG	PY.4G.01037	TRIM17	Tripartite motif containing 17	Chr4:100308785 G>A	0.081395
PG	PY.4G.01084	CRTC1	transcription coactivator 1	Chr4:102194124 C>T	0.127907
PG	PY.5G.00139	TAS2R40	Taste receptor, type 2, member 40	Chr5:13435087 G>C	0.616279
PG	PY.5G.00157	Unknown	Immunoglobulin V-set domain	Chr5:14102395 G>A	0.290698
PG	PY.5G.00160	TRBV6-1	Immunoglobulin V-set domain	Chr5:14137444 C>T	0.651163
PG	PY.5G.00255	SMKR1	Small lysine-rich protein 1	Chr5:26194728 A>G	0.453488
PG	PY.5G.00317	TARP	Immunoglobulin C-Type	Chr5:36792937 A>T	0.44186
PG	PY.5G.00318	TARP	Immunoglobulin C-Type	Chr5:36792937 A>T	0.44186
PG	PY.5G.00320	TARP	Immunoglobulin C-Type	Chr5:36912776 C>T	0.058139
PG	PY.5G.00447	FKBP9	FK506 binding protein 9	Chr5:55740861 T>A	0.214286
PG	PY.5G.00452	CBX1	Chromo shadow domain	Chr5:56149261 G>A	0.095238
PG	PY.5G.00466	RPS7	Ribosomal protein S7e	Chr5:59064798 G>A	0.476744
PG	PY.5G.00473	AIP	Aryl hydrocarbon receptor interacting protein	Chr5:60065107 G>A	0.116279
PG	PY.5G.00497	WNT2	family), member 2	Chr5:68681107 C>T	0.139535
PG	PY.5G.00588	ABCB1	Multidrug resistance protein	Chr5:87010429 C>T	0.325581
PG	PY.5G.00593	ABCB1	Multidrug resistance protein	Chr5:87660924 G>A; Chr5:87675145 A>G	0.209302; 0.104651
PG	PY.5G.00719	ABCA13	cassette subfamily A	Chr5:112763293 T>A; Chr5:112844595 T>C	0.325581; 0.662791
PG	PY.6G.00014	SKP2	S-phase kinase-associated protein 2	Chr6:6065801 C>G	0.104651
PG	PY.6G.00189	DHX15	Oligonucleotide/oligosaccharide-binding (OB)-fold	Chr6:47729931 G>A; Chr6:47729991 G>A	0.372093; 0.348837
PG	PY.6G.00232	RFC1	Replication factor C	Chr6:62101592 C>T	0.127907
PG	PY.6G.00256	Rps4x	ribosomal protein S4	Chr6:64567909 T>A	0.151163
PG	PY.6G.00261	HNRNPK	ROKNT (NUC014) domain	Chr6:66160445 A>C	0.476744

PG	PY.6G.00323	RPS11	Ribosomal protein S11	Chr6:75603671 C>G	0.5
PG	PY.6G.00369	SULT1B1	Sulfotransferase family cytosolic 1B member 1	Chr6:87650921 C>A	0.476744
PG	PY.6G.00373	Unknown	Unknown	Chr6:87885348 G>A	0.406977
PG	PY.6G.00374	CSN1S2	Important role in the capacity of milk to transport calcium phosphate	Chr6:87885348 G>A	0.406977
PG	PY.6G.00391	HSPA8	Molecular chaperone involved in protein quality control, stress responses, transcriptional regulation, and pre-mRNA splicing.	Chr6:89233926 G>A	0.5
PG	PY.6G.00446	FRAS1	extracellular matrix	Chr6:95349340 C>T	0.081395
PG	PY.6G.00460	NAP1L1	nucleosome assembly	Chr6:99316073 G>A	0.058139
PG	PY.6G.00548	MAN2B2	Alpha mannosidase, middle domain	Chr6:115063674 G>A	0.255814
PG	PY.6G.00600	UVSSA	UV-stimulated scaffold protein A	Chr6:119023056 C>A	0.511628
PG	PY.7G.00038	Unknown	Endonuclease/Exonuclease/phosphatase family	Chr7:1720331 C>T	0.802326
PG	PY.7G.00128	EP300	E1A binding protein p300	Chr7:7768626 C>T	0.081395
PG	PY.7G.00195	COQ10B	Coenzyme Q-binding protein COQ10 homolog B	Chr7:11128674 G>T	0.290698
PG	PY.7G.00255	TAPBPL	Tapasin-related protein	Chr7:16217563 G>A	0.186047
PG	PY.7G.00295	Unknown	Scavenger receptor Cys-rich	Chr7:16976357 C>T	0.430233
PG	PY.7G.00297	Unknown	Scavenger receptor Cys-rich	Chr7:17164821 G>A	0.081395
PG	PY.7G.00327	KLRF1	Killer cell lectin-like receptor subfamily F member	Chr7:18743314 C>T	0.116279
PG	PY.7G.00349	TAS2R10	bitter taste receptor activity	Chr7:19847939 C>T	0.232558
PG	PY.7G.00401	PSMC6	26S protease regulatory subunit	Chr7:27279294 G>A	0.151163
PG	PY.7G.00542	TCP11L2	T-complex protein 11-like protein 2	Chr7:47880239 G>A	0.534884
PG	PY.7G.00617	OR6C3	Olfactory receptor	Chr7:58086648 G>A	0.104651
PG	PY.7G.00618	Unknown	Olfactory receptor	Chr7:58111994 G>A	0.104651
PG	PY.7G.00691	RPS23	maintenance of translational fidelity	Chr7:59495908 C>T	0.130952
PG	PY.7G.00806	CCT2	T-complex protein 1 subunit	Chr7:72172035 G>A	0.197674

PG	PY.7G.00842	OCIAD1	OCIA domain-containing protein 1 isoform	Chr7:81181485 C>T	0.686047
PG	PY.7G.00873	Olfr284	Olfactory receptor	Chr7:84230359 C>G	0.093023
PG	PY.7G.00985	KRT77	Keratin, type II cytoskeletal 1b	Chr7:88364392 A>G	0.348837
PG	PY.7G.00987	KRT3	keratin, type II cytoskeletal	Chr7:88420941 C>T	0.406977
PG	PY.7G.01017	RPL19	5.8S rRNA binding	Chr7:89170659 T>C	0.139535
PG	PY.7G.01049	C3orf20	protein C3orf20 homolog	Chr7:90506607 C>T	0.290698
PG	PY.7G.01095	TIPIN	TIMELESS interacting protein	Chr7:102427805 C>A	0.418605
PG	PY.7G.01114	ASNSD1	Asparagine synthetase	Chr7:108668180 C>T	0.162791
PG	PY.7G.01126	RPL7	60S ribosomal protein	Chr7:110100845 G>A	0.569767
PG	PY.7G.01149	TMED2	Protein involved in ER–Golgi vesicular trafficking, cargo selection, GPCR transport, and Golgi organization.	Chr7:114957287 T>C	0.069767
PG	PY.8G.00024	ACTR3	Actin-related protein	Chr8:7304292 A>C	0.232558
PG	PY.8G.00078	hnrnpa1	Heterogeneous nuclear ribonucleoprotein A1-like	Chr8:16146621 C>G	0.162791
PG	PY.8G.00137	IFNB1	interferon	Chr8:24686264 C>T	0.232558
PG	PY.8G.00383	CNTNAP3	Contactin-associated protein-like 3-like	Chr8:67088723 T>G	0.139535
PG	PY.8G.00390	ERH	enhancer of rudimentary homolog	Chr8:68902623 G>A	0.209302
PG	PY.8G.00421	HTD2	Hydroxyacyl-thioester dehydratase type 2	Chr8:71390367 G>A	0.104651
PG	PY.8G.00443	ADAM28	Disintegrin and metalloproteinase domain-containing protein 28	Chr8:74548169 C>A	0.093023
PG	PY.8G.00477	CNTFR	Ciliary neurotrophic factor receptor	Chr8:77027806 T>C	0.267442
PG	PY.8G.00494	PFN2	Profilin	Chr8:78070106 A>T	0.244186
PG	PY.8G.00554	ZNF169	Zinc finger protein	Chr8:86445025 T>C; Chr8:86461660 C>T	0.139535; 0.151163
PG	PY.8G.00711	CUTA	CutA1 divalent ion tolerance protein	Chr8:110492738 G>A; Chr8:110505860 A>G	0.255814; 0.095238
PG	PY.8G.00735	SH3YL1	SH3 domain-containing YSC84-like protein 1	Chr8:112878887 G>A	0.709302
PG	PY.9G.00093	Unknown	Unknown	Chr9:14146949 A>G	0.093023

PG	PY.9G.00097	Unknown	Unknown	Chr9:15252050 C>T	0.27907
PG	PY.9G.00179	NLRC4	NLR family, CARD	Chr9:21007567 C>T; Chr9:21010136 A>G	0.151163; 0.27907
PG	PY.9G.00184	RPL6	60S ribosomal protein	Chr9:21538373 T>A	0.081395
PG	PY.9G.00327	TEAD2	TEA domain	Chr9:50092847 G>A	0.162791
PG	PY.9G.00341	MKRN1	E3 ubiquitin-protein ligase	Chr9:51642670 C>A	0.197674
PG	PY.9G.00635	ODC1	ornithine	Chr9:92310932 G>T	0.197674
PG	PY.10G.00040	Unknown	Unknown	Chr10:13674359 C>T	0.174419
PG	PY.10G.00074	FLVCR2	Feline leukemia virus subgroup C receptor-related protein	Chr10:16076471 T>C	0.686047
PG	PY.10G.00204	EEF1G	Elongation factor	Chr10:29098026 G>A	0.430233
PG	PY.10G.00219	RTN1	Reticulon 1	Chr10:31064262 G>C	0.069767
PG	PY.10G.00313	CYP19A1	cytochrome P450	Chr10:43575887 A>T	0.360465
PG	PY.10G.00314	CYP19A1	cytochrome P450	Chr10:43575887 A>T	0.360465
PG	PY.10G.00407	PIF1	DNA helicase involved in genome stability, G-quadruplex resolution, and telomere maintenance.	Chr10:57331228 A>C	0.186047
PG	PY.10G.00604	GALNT1	polypeptide N-acetylgalactosaminyltransferase	Chr10:75440246 C>T	0.209302
PG	PY.10G.00663	TRAV8-6	Immunoglobulin V-set domain	Chr10:77030606 G>A; Chr10:77050788 G>T; Chr10:77069011 T>C	0.418605; 0.186047; 0.174419
PG	PY.10G.00664	PDE7A	phosphodiesterase 7A	Chr10:77193862 C>A	0.162791
PG	PY.10G.00897	Unknown	Unknown	Chr10:96531562 G>A	0.162791
PG	PY.10G.00902	GBAS	negative regulation of ATP citrate synthase activity	Chr10:98298652 G>A	0.348837
PG	PY.11G.00020	KIF25	negative regulation of mitotic centrosome separation	Chr11:1748570 A>G	0.186047
PG	PY.11G.00050	PNLDC1	poly(A)-specific ribonuclease PARN-like domain-containing protein 1	Chr11:8043591 A>G	0.232558
PG	PY.11G.00099	CCDC170	Coiled-coil domain containing 170	Chr11:15313715 G>A	0.151163
PG	PY.11G.00110	ULBP1	UL16 binding protein	Chr11:16714579 G>A	0.209302

PG	PY.11G.00151	Unknown	Unknown	Chr11:24403369 A>G	0.232558
PG	PY.11G.00158	ECT2L	Epithelial cell transforming 2 like	Chr11:26965581 T>A; Chr11:27041001 A>G	0.244186; 0.22093
PG	PY.11G.00200	TAAR2	trace-amine receptor activity	Chr11:32704452 G>A	0.232558
PG	PY.11G.00204	TAAR6	Belongs to the G-protein coupled receptor 1 family	Chr11:32741371 G>A	0.151163
PG	PY.11G.00223	MTFR1	mitochondrial fission regulator 1	Chr11:34855935 C>T	0.581395
PG	PY.11G.00249	CFAP206	UPF0704 protein C6orf165 homolog	Chr11:40666271 C>T	0.127907
PG	PY.11G.00271	UTP14A	U3 small nucleolar RNA-associated protein 14 homolog	Chr11:43804167 G>A	0.093023
PG	PY.11G.00451	RPA3OS	RPA3 opposite strand	Chr11:83443714 A>T	0.848837
PG	PY.12G.00080	Olfr132	Olfactory receptor	Chr12:13200243 G>C	0.162791
PG	PY.12G.00144	EIF3E	Component of the eIF-3 complex involved in translation initiation, translational regulation, and nonsense-mediated mRNA decay.	Chr12:25382993 A>G	0.081395
PG	PY.12G.00400	Unknown	Unknown	Chr12:78966056 C>T	0.360465
PG	PY.12G.00494	RPS9	positive regulation of translational fidelity	Chr12:80931087 A>G	0.162791
PG	PY.12G.00508	COL9A1	collagen	Chr12:90014891 G>T	0.104651
PG	PY.13G.00045	Unknown	Unknown	Chr13:7108340 A>G	0.906977
PG	PY.13G.00089	PTI	Pancreatic trypsin inhibitor-like	Chr13:9358706 A>G	0.081395
PG	PY.13G.00167	CMPK1	Enzyme involved in pyrimidine nucleotide biosynthesis by phosphorylating UMP and CMP, with additional nucleoside diphosphate kinase activity.	Chr13:17224571 A>G	0.139535
PG	PY.13G.00193	FER1L4	genomic stop codons	Chr13:18561573 G>A; Chr13:18561711 G>T	0.22093; 0.232558
PG	PY.13G.00203	MMP24	Domain of unknown function (DUF3377)	Chr13:18822190 C>G	0.627907
PG	PY.13G.00328	VAPB	Vesicle-associated membrane protein-associated protein B	Chr13:25469431 C>T	0.197674

PG	PY.13G.00357	RBBP8NL	RBBP8 N-terminal like	Chr13:28571570 C>A	0.151163
PG	PY.13G.00490	SURF1	Surfeit locus protein	Chr13:37637285 A>G	0.581395
PG	PY.13G.00498	AKR1C4	Dihydrodiol dehydrogenase 3-like	Chr13:38967642 C>T	0.52381
PG	PY.13G.00505	AKR1C4	Dihydrodiol dehydrogenase 3-like	Chr13:39218261 G>A	0.232558
PG	PY.13G.00688	Unknown	Unknown	Chr13:64397132 A>G; Chr13:64397462 A>T	0.465116; 0.406977
PG	PY.13G.00715	ATP5C1	ATP synthase subunit gamma, mitochondrial	Chr13:69731155 C>A	0.104651
PG	PY.13G.00727	FBXO18	F-box only protein 18	Chr13:71301671 C>T	0.406977
PG	PY.13G.00728	NINL	ninein-like	Chr13:71364127 C>T; Chr13:71384746 C>T	0.162791; 0.139535
PG	PY.13G.00763	CAP1	cyclase-associated protein 1	Chr13:81415040 C>T	0.488372
PG	PY.14G.00027	TIA1	TIA1 cytotoxic granule associated RNA binding protein	Chr14:12299042 C>G	0.209302
PG	PY.14G.00040	ATP5L	ATP synthase subunit g, mitochondrial-like	Chr14:13146693 C>T	0.069767
PG	PY.14G.00053	LDHB	dehydrogenase B	Chr14:16896237 A>G; Chr14:16896800 G>A	0.186047; 0.186047
PG	PY.14G.00118	BCO2	Beta,beta-carotene 9',10'-oxygenase	Chr14:28863080 T>G	0.255814
PG	PY.14G.00125	TMPRSS5	Transmembrane protease, serine 5	Chr14:30572128 T>A; Chr14:30575719 C>T	0.232558; 0.174419
PG	PY.14G.00224	C11orf63	protein C11orf63 homolog	Chr14:39876723 G>C	0.360465
PG	PY.14G.00392	Olfr639	Serpentine type 7TM GPCR chemoreceptor Srsx	Chr14:52963437 G>T	0.244186
PG	PY.14G.00450	OR52B4	Olfactory receptor	Chr14:53893280 T>C	0.627907
PG	PY.14G.00455	EEF1A1	Elongation factor	Chr14:53949976 T>C	0.453488
PG	PY.14G.00565	Unknown	Unknown	Chr14:64239078 G>C	0.488372
PG	PY.14G.00614	ATMIN	ATM interactor	Chr14:70733960 C>G	0.151163
PG	PY.14G.00637	SLC35C1	GDP-fucose transporter 1	Chr14:78263364 G>A	0.081395

PG	PY.14G.00717	OR8K1	Olfactory receptor	Chr14:80948704 A>G	0.465116
PG	PY.14G.00731	Olfr1009	Olfactory receptor	Chr14:81161212 A>T	0.197674
PG	PY.15G.00010	PCDH8	Cadherin repeats.	Chr15:11706937 G>T	0.069767
PG	PY.15G.00034	EPSTI1	Epithelial stromal interaction 1	Chr15:14355389 G>A	0.127907
PG	PY.15G.00045	NUFIP1	nuclear fragile X mental	Chr15:16382131 G>A	0.302326
PG	PY.15G.00098	NEK5	Serine threonine-protein kinase	Chr15:22697933 C>T	0.081395
PG	PY.15G.00143	AIDA	Cytoskeletal adhesion	Chr15:30816119 G>A	0.22619
PG	PY.15G.00185	SPATA13	Guanine nucleotide exchange factor for Rho/Rac/Cdc42-like GTPases	Chr15:35435353 G>T	0.081395
PG	PY.15G.00222	Unknown	Unknown	Chr15:45997578 C>T	0.058139
PG	PY.15G.00325	Unknown	Unknown	Chr15:80415847 C>A	0.593023
PG	PY.15G.00333	Unknown	Sperm acrosome associated 7	Chr15:81015652 G>A	0.325581
PG	PY.15G.00336	Unknown	Unknown	Chr15:81289692 A>G	0.130952
PG	PY.15G.00338	Unknown	Unknown	Chr15:81542017 T>C; Chr15:81548991 A>G; Chr15:81549049 T>C	0.255814; 0.058139; 0.058139
PG	PY.16G.00010	RNPEP	Leukotriene A4 hydrolase, C-terminal	Chr16:420374 C>T	0.081395
PG	PY.16G.00015	PTPRB	Protein tyrosine phosphatase, catalytic domain	Chr16:531398 C>T	0.453488
PG	PY.16G.00044	Unknown	Unknown	Chr16:3314225 G>C	0.627907
PG	PY.16G.00118	FAM129A	family with sequence similarity 129, member A	Chr16:14665560 C>T	0.52381
PG	PY.16G.00143	Unknown	Unknown	Chr16:18289785 G>A	0.209302
PG	PY.16G.00325	PER3	Period circadian	Chr16:34444319 C>T; Chr16:34456564 C>T	0.058139; 0.139535
PG	PY.16G.00502	Unknown	Unknown	Chr16:55775846 G>C	0.081395
PG	PY.16G.00537	TRA2B	Transformer-2 protein homolog	Chr16:70760606 G>C	0.139535
PG	PY.16G.00546	C4BPA	Complement component 4 binding protein alpha	Chr16:73359421 A>G	0.093023

PG	PY.16G.00580	Unknown	Unknown	Chr16:75132484 T>C	0.546512
PG	PY.16G.00584	TMCC2	transmembrane and coiled-coil domain family 2	Chr16:75273310 T>C	0.255814
PG	PY.17G.00014	C22orf29	substituted 1 base at 1 genomic stop codon	Chr17:323878 G>A; Chr17:325299 A>G	0.325581; 0.244186
PG	PY.17G.00098	Unknown	Immunoglobulin V-set domain	Chr17:2102658 T>A	0.116279
PG	PY.17G.00169	TPST2	sulfotransferase 2	Chr17:6300774 G>T; Chr17:6300979 C>A	0.209302; 0.209302
PG	PY.17G.00235	rpsa	40S ribosomal protein SA-like	Chr17:9895575 C>T	0.302326
PG	PY.17G.00383	Unknown	Unknown	Chr17:25665298 C>G	0.27907
PG	PY.17G.00405	PXMP2	peroxisomal membrane protein 2	Chr17:27975919 G>A	0.27907
PG	PY.17G.00431	RXFP1	family peptide receptor 1	Chr17:31433044 A>G	0.255814
PG	PY.17G.00445	RPL19	ribosomal protein	Chr17:35530773 G>A	0.55814
PG	PY.17G.00463	Unknown	Unknown	Chr17:40202911 G>A	0.267442
PG	PY.17G.00478	GLRX3	Glutaredoxin	Chr17:41467935 T>C	0.372093
PG	PY.18G.00011	BRD9	Bromodomain-containing protein 9	Chr18:518703 C>T	0.404762
PG	PY.18G.00041	MTRR	5-methyltetrahydrofolate-homocysteine methyltransferase reductase	Chr18:6521275 T>C	0.102564
PG	PY.18G.00095	NPR3	Natriuretic peptide receptor 3	Chr18:29043100 C>A	0.174419
PG	PY.18G.00288	HEXB	Beta-hexosaminidase subunit	Chr18:63021088 G>T	0.127907
PG	PY.19G.00161	BCL2A1	Bcl-2-related protein A1	Chr19:28407491 A>G	0.186047
PG	PY.19G.00327	PPP1CC	Serine threonine-protein phosphatase PP1-gamma catalytic	Chr19:49161489 G>A	0.27907
PG	PY.19G.00340	SKP2	S-phase kinase-associated protein 2	Chr19:51606999 G>C	0.186047
PG	PY.19G.00477	Unknown	Unknown	Chr19:68714327 C>T; Chr19:68722962 A>G	0.360465; 0.069767
PG	PY.20G.00024	Unknown	krueppel associated box	Chr20:638004 G>A	0.430233
PG	PY.20G.00089	LILRB3	Leukocyte immunoglobulin-like receptor subfamily	Chr20:2382649 C>T; Chr20:2384601 G>C; Chr20:2389615 T>C	0.406977; 0.345238; 0.822581

PG	PY.20G.00162	AU018091	Cationic amino acid transporter 3-like	Chr20:3733442 G>T	0.151163
PG	PY.20G.00186	Unknown	Unknown	Chr20:5133528 G>C	0.395349
PG	PY.20G.00193	PPP2R1A	Serine threonine-protein phosphatase 2A 65 kDa regulatory subunit A alpha	Chr20:5368223 C>T	0.476744
PG	PY.20G.00399	BHMG1	high mobility group	Chr20:9913831 C>G	0.337209
PG	PY.20G.00473	BSP5	metalloendopeptidase activity	Chr20:11554495 C>T	0.627907
PG	PY.20G.00554	LEUTX	homeobox	Chr20:13660526 G>A	0.174419
PG	PY.20G.00577	FBXO27	F-box only protein	Chr20:14165018 A>T	0.313953
PG	PY.20G.00663	EI24	Etoposide-induced protein 2.4 homolog	Chr20:16549179 C>T	0.360465
PG	PY.20G.00707	C1orf43	NICE-3 protein	Chr20:18128289 C>G	0.139535
PG	PY.20G.00741	HYDIN	HYDIN, axonemal central pair apparatus protein	Chr20:22546645 A>T	0.190476
PG	PY.20G.00742	TCEAL8	transcription elongation factor A	Chr20:22775605 C>A; Chr20:22775789 A>G	0.069767; 0.360465
PG	PY.20G.00930	YWHAQ	14-3-3 protein	Chr20:39327817 C>G	0.209302
PG	PY.20G.01021	KLHDC4	Kelch domain-containing protein 4	Chr20:49141293 A>C	0.186047
PG	PY.20G.01115	PDPR	Pyruvate dehydrogenase phosphatase regulatory subunit	Chr20:60566794 A>G	0.081395
PG	PY.21G.00017	PCTP	Phosphatidylcholine transfer protein-like	Chr21:10293777 C>T	0.130952
PG	PY.21G.00020	PCTP	Phosphatidylcholine transfer protein-like	Chr21:10469429 G>A	0.72093
PG	PY.21G.00094	MRM1	rRNA methyltransferase 1	Chr21:17018627 A>G	0.837209
PG	PY.21G.00130	SLFN11	Schlafen family member	Chr21:18871207 C>A	0.093023
PG	PY.21G.00171	WSB1	WD repeat and SOCS	Chr21:23051716 T>A	0.406977
PG	PY.21G.00282	OR3A2	Olfactory receptor	Chr21:27999844 T>A	0.686047
PG	PY.21G.00722	Unknown	Unknown	Chr21:45070841 G>A	0.151163
PG	PY.21G.00770	STAT3	Signal transducer and activator of transcription	Chr21:46050901 G>A	0.116279
PG	PY.21G.00982	FSCN2	Fascin actin-bundling protein 2, retinal	Chr21:54056933 C>T	0.151163

PG	PY.22G.00037	KIAA1257	Uncharacterized protein FLJ43738-like	Chr22:1732350 C>T	0.232558
PG	PY.22G.00288	ASB14	Ankyrin repeat and SOCS box	Chr22:16641651 G>A	0.116279
PG	PY.22G.00295	Unknown	Unknown	Chr22:17333741 C>T	0.093023
PG	PY.22G.00318	Unknown	cadherin-related family member	Chr22:22757705 C>A	0.197674
PG	PY.23G.00005	SERPINB13	Serpin B13	Chr23:202470 A>G	0.395349
PG	PY.23G.00103	CEP192	positive regulation of TORC1 signaling	Chr23:18192107 C>T	0.104651
PG	PY.23G.00183	LAMA3	Laminin subunit	Chr23:28634342 A>T	0.104651
PG	PY.23G.00266	Unknown	Unknown	Chr23:56544495 C>T	0.186047
PG	PY.23G.00268	Unknown	Unknown	Chr23:56665675 A>G	0.744186
PG	PY.24G.00023	CFAP46	Cilia and flagella associated protein 46	Chr24:791295 C>T	0.313953
PG	PY.24G.00061	OVCA2	Ovarian tumor suppressor candidate 2	Chr24:7358635 T>C	0.813953
PG	PY.24G.00132	ABLIM1	Actin binding LIM protein 1	Chr24:16807336 C>T	0.104651
PG	PY.24G.00243	SEC31B	SEC31 homolog B, COPII coat complex component	Chr24:30074042 G>C	0.302326
PG	PY.25G.00012	DST	Dystonin	Chr25:7404032 C>T	0.337209
PG	PY.25G.00388	Unknown	PRY	Chr25:31273751 T>A	0.058139
PG	PY.25G.00424	Unknown	Olfactory receptor	Chr25:31818536 C>T	0.174419
PG	PY.25G.00559	Unknown	Unknown	Chr25:38206651 C>T	0.151163
PG	PY.25G.00650	RIPK1	Receptor-interacting serine threonine-protein kinase	Chr25:52011160 C>T	0.406977
PG	PY.26G.00049	MUC5B	C8	Chr26:918065 A>G	0.651163
PG	PY.26G.00103	Unknown	Unknown	Chr26:4326909 T>C	0.5
PG	PY.26G.00124	TCIRG1	V-type proton ATPase 116 kDa subunit a isoform 3	Chr26:5502645 A>G	0.732558
PG	PY.26G.00250	SLC22A11	solute carrier family 22	Chr26:8229653 C>T	0.290698
PG	PY.26G.00387	Unknown	Unknown	Chr26:12793166 T>C	0.383721
PG	PY.26G.00408	FLI1	megakaryocyte development	Chr26:17229875 T>C	0.302326
PG	PY.26G.00458	Unknown	Olfactory receptor	Chr26:21408572 A>G	0.267442

PG	PY.26G.00613	TBC1D22B	TBC1 domain family, member 22B	Chr26:43612803 A>C	0.255814
PG	PY.27G.00007	OR4C6	Olfactory receptor	Chr27:4225931 C>T	0.081395
PG	PY.27G.00009	OR4C15	Olfactory receptor	Chr27:4346954 C>T	0.44186
PG	PY.27G.00052	RHBDL1	Rhomboid-related protein 1	Chr27:5986605 T>C	0.732558
PG	PY.27G.00058	METRN	Meteorin, glial cell differentiation regulator	Chr27:6021389 A>G; Chr27:6022147 C>T	0.139535; 0.127907
PG	PY.27G.00174	ZNF75A	Zinc finger protein	Chr27:8129342 G>A	0.22093
PG	PY.27G.00182	GTSE1	G2 and S phase-expressed protein 1	Chr27:8331859 G>A	0.627907
PG	PY.27G.00348	Unknown	Endonuclease/Exonuclease/phosphatase family	Chr27:30195585 G>A; Chr27:30195804 C>T	0.186047; 0.197674
PG	PY.27G.00410	ITGAL	integrin	Chr27:31770436 A>G	0.209302
PG	PY.27G.00551	MUC12	Mucin 12, cell surface associated	Chr27:40702849 C>G	0.488372
PG	PY.27G.00607	FAM200A	Family with sequence similarity 200, member A	Chr27:41834076 C>T; Chr27:41834750 G>A; Chr27:41834871 C>T	0.232558; 0.255814; 0.255814
PG	PY.27G.00681	Unknown	Unknown	Chr27:46189424 G>A	0.232558
PG	PY.27G.00684	TMEM184A	transmembrane protein 184A	Chr27:46311680 T>C	0.267442
PG	PY.28G.00012	Unknown	Unknown	Chr28:4005016 T>C	0.209302
PG	PY.28G.00066	PPAPDC1B	phosphatase	Chr28:12088306 C>T	0.104651
PG	PY.28G.00102	GSR	Belongs to the class-I pyridine nucleotide-disulfide oxidoreductase family	Chr28:18534564 C>T	0.313953
PG	PY.28G.00103	RPL19	ribosomal protein	Chr28:18586617 T>G	0.232558
PG	PY.28G.00117	LRP5L	lipoprotein receptor-related protein	Chr28:20668840 G>A	0.511628
PG	PY.28G.00233	CSMD1	CUB and sushi	Chr28:43162050 C>G	0.523256
PG	PY.29G.00071	LIN28A	Lin-28 homolog A	Chr29:14152725 C>T	0.069767
PG	PY.29G.00112	RPS15A	ribosomal protein	Chr29:24909535 T>C	0.267442
PG	PY.29G.00156	CHST3	sulfotransferase 3	Chr29:28228028 G>A	0.127907

PG	PY.29G.00255	EIF1B	translation initiation factor activity	Chr29:41477067 G>A	0.116279
PG	PY.29G.00289	Unknown	Unknown	Chr29:44516098 T>G	0.093023
TG	ZY.1G.00077	Unknown	Unknown	Chr1:12209746 C>A	0.291667
TG	ZY.1G.00086	TSPEAR	Thrombospondin-type laminin G domain and EAR	Chr1:12801398 A>C	0.083333
TG	ZY.1G.00105	DNMT3L	DNA (cytosine-5)-methyltransferase 3-like	Chr1:13075202 C>T	0.791667
TG	ZY.1G.00256	CPA3	Carboxypeptidase	Chr1:38044690 A>C	0.083333
TG	ZY.1G.00292	AADAC	Arylacetamide	Chr1:41234430 G>A	0.083333
TG	ZY.1G.00391	NAALADL2	Inactive N-acetylated-alpha-linked acidic dipeptidase-like protein 2-like	Chr1:64909261 G>A	0.083333
TG	ZY.1G.00433	HTR3E	serotonin-gated cation-selective channel activity	Chr1:74479406 C>A	0.083333
TG	ZY.1G.00455	RPL7	ribosomal protein	Chr1:75447031 G>T	0.083333
TG	ZY.1G.00610	ADPRH	ADP-ribosylarginine hydrolase	Chr1:93251113 C>T	0.125
TG	ZY.1G.00652	ABHD10	Mycophenolic acid acyl-glucuronide esterase	Chr1:100853663 C>T	0.541667
TG	ZY.1G.00692	ABI3BP	ABI family, member 3	Chr1:112476517 G>A	0.083333
TG	ZY.1G.00737	EEF1A1	Elongation factor 1-alpha	Chr1:118827601 C>A	0.333333
TG	ZY.1G.00777	MOB4	MOB-like protein phocein	Chr1:143530782 G>A	0.125
TG	ZY.1G.00810	Krtap13	Keratin-associated protein 13-1-like	Chr1:153581192 G>A	0.333333
TG	ZY.2G.00124	SYF2	pre-mRNA-splicing factor	Chr2:8515220 C>T	0.083333
TG	ZY.2G.00198	PHACTR4	phosphatase and actin regulator 4	Chr2:11263452 T>C	0.5
TG	ZY.2G.00212	PTPRU	phosphatase	Chr2:12547228 C>T	0.208333
TG	ZY.2G.00253	RPL6	5.8S rRNA binding	Chr2:15107369 C>G	0.25
TG	ZY.2G.00299	SP100	SP100 nuclear antigen	Chr2:17611788 C>T; Chr2:17714740 T>C	0.458333; 0.75
TG	ZY.2G.00366	RPS8	Belongs to the eukaryotic ribosomal protein eS8 family	Chr2:29046355 C>A	0.666667
TG	ZY.2G.00477	Unknown	Unknown	Chr2:44173269 C>A	0.125
TG	ZY.2G.00590	MARCO	Macrophage receptor with collagenous structure	Chr2:66145251 C>A	0.083333

TG	ZY.2G.00624	DARS	aspartate--tRNA ligase, cytoplasmic	Chr2:75616454 C>T	0.083333
TG	ZY.2G.00653	MAN1A2	Mannosidase, alpha, class 1A, member 2	Chr2:90358954 C>T	0.083333
TG	ZY.2G.00763	HAT1	Histone acetyl transferase HAT1 N-terminus	Chr2:113063080 C>A	0.5
TG	ZY.2G.00784	TMEM183A	transmembrane protein 183A	Chr2:115390494 T>C	0.291667
TG	ZY.2G.00848	MTIF3	Initiation factor	Chr2:127863012 C>A; Chr2:128004626 C>A	0.166667; 0.25
TG	ZY.2G.00869	RPL37	Ribosomal protein L37e	Chr2:131945240 A>G	0.166667
TG	ZY.2G.00872	INPP1	inositol	Chr2:132136579 G>A	0.875
TG	ZY.3G.00007	snrpe	LSM domain	Chr3:17492247 G>A	0.208333
TG	ZY.3G.00079	AP1S2	Clathrin adaptor complex small chain	Chr3:32821928 A>T	0.227273
TG	ZY.3G.00097	AGMO	Alkylglycerol monooxygenase	Chr3:38274330 T>A	0.166667
TG	ZY.3G.00134	SLC40A1	Ferroportin1 (FPN1)	Chr3:46179083 A>G	0.083333
TG	ZY.3G.00164	HNRNPH1	heterogeneous nuclear ribonucleoprotein	Chr3:50614691 C>T	0.25
TG	ZY.3G.00225	LAMB4	genomic stop codons	Chr3:64107481 G>A	0.083333
TG	ZY.3G.00234	TARP	Immunoglobulin C-Type	Chr3:65098816 A>G	0.333333
TG	ZY.3G.00240	PGAM1	phosphoglycerate mutase	Chr3:66055639 G>C	0.791667
TG	ZY.3G.00299	AVL9	Late secretory pathway protein AVL9 homolog	Chr3:79147590 A>C	0.625
TG	ZY.3G.00420	TARP	Immunoglobulin C-Type	Chr3:97891021 G>T	0.083333
TG	ZY.3G.00453	SUMO1	Small ubiquitin-related modifier 1-like	Chr3:106883682 A>G	0.291667
TG	ZY.3G.00508	AKR1B10	aldo-keto reductase family 1, member B10	Chr3:113413903 C>A	0.208333
TG	ZY.3G.00582	Unknown	Immunoglobulin V-set domain	Chr3:120577629 C>T	0.291667
TG	ZY.3G.00636	Olfr13	Olfactory receptor	Chr3:122108191 T>C	0.166667
TG	ZY.3G.00680	GIMAP8	GTPase IMAP family member 8	Chr3:128001272 G>C	0.166667
TG	ZY.3G.00711	GALNT11	polypeptide N-acetylgalactosaminyltransferase	Chr3:129342955 G>A	0.083333
TG	ZY.4G.00026	RAN	snRNA import into nucleus	Chr4:14704230 T>C	0.25

TG	ZY.4G.00123	SLAMF8	SLAM family member 8	Chr4:21779454 C>T	0.125
TG	ZY.4G.00162	CD1B	adaptive immune response	Chr4:23654905 A>G	0.166667
TG	ZY.4G.00170	FCRL2	Fc receptor-like	Chr4:24184336 G>A	0.583333
TG	ZY.4G.00201	IQGAP3	IQ motif containing GTPase activating protein 3	Chr4:25818085 T>A	0.125
TG	ZY.4G.00228	GON4L	GON-4-like	Chr4:26553934 C>T	0.083333
TG	ZY.4G.00284	CRTC2	transcription coactivator 2	Chr4:28017104 T>A	0.25
TG	ZY.4G.00302	S100A6	S100 calcium binding protein A6	Chr4:28367508 C>T	0.083333
TG	ZY.4G.00391	ERH	Enhancer of rudimentary	Chr4:31598446 C>T	0.125
TG	ZY.4G.00400	METTL9	Methyltransferase-like protein	Chr4:31831284 C>A	0.208333
TG	ZY.4G.00498	CD58	Lymphocyte function-associated antigen 3	Chr4:38992647 T>A	0.208333
TG	ZY.4G.00554	OVGP1	Binds to oocyte zona pellucida in vivo. May play a role in the fertilization process and or early embryonic development	Chr4:44096345 T>C; Chr4:44104307 G>A	0.272727; 0.090909
TG	ZY.4G.00671	Unknown	Unknown	Chr4:61198400 G>A	0.458333
TG	ZY.4G.00673	Unknown	Unknown	Chr4:61244237 C>T	0.583333
TG	ZY.4G.00704	ATP5A1	proton-transporting ATP synthase activity, rotational mechanism	Chr4:64481289 A>G	0.416667
TG	ZY.4G.00713	BCDIN3D	pre-miRNA 5'-monophosphate methyltransferase	Chr4:66125347 G>A	0.583333
TG	ZY.4G.00716	GBP1	Interferon-induced guanylate-binding protein	Chr4:66278834 C>T	0.333333
TG	ZY.4G.00836	JAK1	Tyrosine-protein kinase JAK1	Chr4:92148731 A>G	0.291667
TG	ZY.4G.00893	ACOT11	Acyl-coenzyme A thioesterase 11	Chr4:103642084 T>C	0.125
TG	ZY.4G.00938	Calr4	Calreticulin-like	Chr4:106710846 G>A	0.083333
TG	ZY.4G.01034	ST3GAL3	ST3 beta-galactoside alpha-2,3-sialyltransferase 3	Chr4:114116519 C>T	0.125
TG	ZY.4G.01086	GTF2F2	general transcription factor IIF subunit 2-like	Chr4:117376155 C>T	0.208333
TG	ZY.4G.01135	Unknown	Unknown	Chr4:120968079 A>T	0.625
TG	ZY.4G.01236	Unknown	Unknown	Chr4:131100340 G>A	0.333333
TG	ZY.4G.01244	AQP12B	passive transmembrane transporter activity	Chr4:131392479 A>G	0.833333

TG	ZY.5G.00038	TNIP2	TNFAIP3 interacting protein 2	Chr5:1788266 A>G	0.208333
TG	ZY.5G.00074	MAN2B2	Alpha mannosidase, middle domain	Chr5:4790761 G>C; Chr5:4799261 C>T	0.708333; 0.458333
TG	ZY.5G.00077	FTL	Iron-storage protein involved in iron homeostasis and protection against iron toxicity.	Chr5:5314201 G>A	0.083333
TG	ZY.5G.00165	BRCC3	BRCA1 BRCA2-containing complex, subunit 3	Chr5:22392018 G>A; Chr5:22392146 A>G	0.333333; 0.5
TG	ZY.5G.00246	CSN2	Important role in determination of the surface properties of the casein micelles	Chr5:31939289 A>C	0.125
TG	ZY.5G.00260	UGT2B4	glucuronosyltransferase activity	Chr5:32731810 G>A	0.333333
TG	ZY.5G.00267	TMPRSS11B	transmembrane protease serine	Chr5:33149759 G>A	0.083333
TG	ZY.5G.00285	IL4R	receptor	Chr5:41701911 T>G	0.416667
TG	ZY.5G.00318	Unknown	Unknown	Chr5:46279865 A>G	0.125
TG	ZY.5G.00379	RPS23	maintenance of translational fidelity	Chr5:56990899 C>T	0.291667
TG	ZY.5G.00429	FTL	Iron-storage protein involved in iron homeostasis and protection against iron toxicity.	Chr5:72184724 C>T	0.25
TG	ZY.5G.00452	HERC6	HECT and RLD domain containing E3 ubiquitin protein ligase family member 6	Chr5:79831786 G>T; Chr5:79833437 C>T	0.291667; 0.375
TG	ZY.5G.00499	Unknown	Unknown	Chr5:91600177 T>C	0.083333
TG	ZY.5G.00502	GLUL	glutamine	Chr5:92390388 G>C	0.625
TG	ZY.5G.00522	GSTCD	Glutathione S-transferase C-terminal	Chr5:96800187 T>A	0.090909
TG	ZY.5G.00538	COL25A1	Collagen alpha-1(XXV) chain	Chr5:100040945 C>T	0.208333
TG	ZY.5G.00619	Olfr1178	Olfactory receptor	Chr5:118681791 G>A	0.5
TG	ZY.6G.00049	WTAP	Wilms tumor 1 associated protein	Chr6:22791255 C>T	0.125
TG	ZY.6G.00318	ARPC3	protein 2 3 complex subunit 3	Chr6:49163166 G>T	0.416667
TG	ZY.6G.00630	Unknown	protein HP-25 homolog	Chr6:81574270 C>T	0.125
TG	ZY.6G.00749	PSMC6	26S protease regulatory subunit	Chr6:100112893 C>T	0.125

TG	ZY.6G.00755	NAP1L1	nucleosome assembly	Chr6:101169602 G>A	0.125
TG	ZY.6G.00759	SLC15A5	solute carrier family 15 member 5	Chr6:102485667 C>A	0.75
TG	ZY.6G.00809	KLRC1	NKG2-A NKG2-B type II integral membrane	Chr6:107922246 C>A	0.083333
TG	ZY.6G.00825	KLRF1	Killer cell lectin-like receptor subfamily F member	Chr6:108652181 C>T	0.125
TG	ZY.6G.00851	CD163	Scavenger receptor Cys-rich	Chr6:110065695 G>A	0.166667
TG	ZY.6G.00856	DHX15	Oligonucleotide/oligosaccharide-binding (OB)-fold	Chr6:110398089 C>T	0.272727
TG	ZY.6G.00957	COQ10B	Coenzyme Q-binding protein COQ10 homolog B	Chr6:116277353 C>T	0.125
TG	ZY.6G.01038	NHP2L1	U4atac snRNA binding	Chr6:120044155 T>C	0.583333
TG	ZY.6G.01072	EFCAB6	EF-hand calcium-binding domain-containing protein 6	Chr6:121736289 G>A	0.166667
TG	ZY.7G.00009	Gm355	Cell cycle regulatory protein	Chr7:21734862 C>A	0.166667
TG	ZY.7G.00035	NGFRAP1	Protein BEX3 isoform	Chr7:23452793 C>T	0.125
TG	ZY.7G.00059	FBXL14	ubiquitin-protein transferase activity	Chr7:24963321 A>G	0.416667
TG	ZY.7G.00069	AFF3	AF4 FMR2 family, member	Chr7:25857899 C>T	0.208333
TG	ZY.7G.00163	FIGLA	Folliculogenesis specific bHLH transcription factor	Chr7:34709015 G>A	0.5
TG	ZY.7G.00176	NLRC4	NLR family, CARD	Chr7:35923448 A>G	0.125
TG	ZY.7G.00181	RPL6	60S ribosomal protein	Chr7:36453315 T>A	0.916667
TG	ZY.7G.00196	SULT6B1	sulfotransferase family, cytosolic, 6B, member 1	Chr7:40717983 G>A	0.083333
TG	ZY.7G.00320	Unknown	Unknown	Chr7:64375000 T>A	0.208333
TG	ZY.7G.00331	TEAD2	TEA domain	Chr7:65025664 G>A	0.083333
TG	ZY.7G.00336	CCDC138	Coiled-coil domain containing 138	Chr7:65541189 C>T	0.083333
TG	ZY.7G.00358	NT5DC4	5'-nucleotidase domain containing 4	Chr7:67302350 A>G	0.458333
TG	ZY.7G.00560	OTOF	Otoferlin	Chr7:93140019 G>T	0.083333
TG	ZY.7G.00705	Unknown	Olfactory receptor	Chr7:113750698 C>T	0.166667
TG	ZY.7G.00836	RPS4X	ribosomal protein S4	Chr7:121978327 T>C	0.375
TG	ZY.7G.00853	PAEP	Lipocalin / cytosolic fatty-acid binding protein family	Chr7:122979020 T>C	0.333333

TG	ZY.7G.00908	MRPS2	ribosomal protein S2	Chr7:125477126 C>A	0.318182
TG	ZY.8G.00063	Unknown	Unknown	Chr8:21715127 T>C	0.291667
TG	ZY.8G.00077	TMEM167A	Protein of unknown function (DUF1242)	Chr8:27325650 G>A	0.125
TG	ZY.8G.00100	HMMR	Hyaluronan mediated motility receptor	Chr8:35849355 G>A	0.291667
TG	ZY.8G.00171	Unknown	Unknown	Chr8:47705155 C>T	0.083333
TG	ZY.8G.00413	RPS15	ribosomal protein	Chr8:66473793 G>A	0.333333
TG	ZY.8G.00524	BTNL9	Butyrophilin-like protein	Chr8:69265227 G>A	0.125
TG	ZY.8G.00539	RMND5B	Required for meiotic nuclear division 5 homolog B	Chr8:70144822 C>T	0.125
TG	ZY.8G.00608	ZNF608	Zinc finger protein	Chr8:80391381 C>T	0.208333
TG	ZY.8G.00644	ST13	suppression of tumorigenicity 13 (colon carcinoma) (Hsp70 interacting protein)	Chr8:87293236 G>A	0.25
TG	ZY.8G.00836	IFT57	Intraflagellar transport protein 57 homolog	Chr8:93456006 C>A	0.083333
TG	ZY.8G.00840	HMGN1	pyrimidine dimer repair by nucleotide-excision repair	Chr8:93559405 T>C	0.625
TG	ZY.8G.00862	TBC1D26	Rab-GTPase-TBC domain	Chr8:94068031 A>G	0.125
TG	ZY.8G.01129	ANKLE1	Ankyrin repeat and LEM	Chr8:103552557 C>T	0.208333
TG	ZY.8G.01229	Unknown	Olfactory receptor	Chr8:106821587 A>G	0.208333
TG	ZY.9G.00019	EXOC6B	Exocyst complex component 6B	Chr9:13094109 C>T; Chr9:13094333 C>T	0.416667; 0.25
TG	ZY.9G.00131	Unknown	Unknown	Chr9:26139479 G>A	0.125
TG	ZY.9G.00233	GLUL	glutamine	Chr9:31225101 C>T; Chr9:31225336 G>A	0.083333; 0.083333
TG	ZY.9G.00235	TRDV1	Immunoglobulin V-set domain	Chr9:31612538 A>G; Chr9:31647190 C>G	0.083333; 0.083333
TG	ZY.9G.00242	TRAV18	Immunoglobulin V-set domain	Chr9:32027788 C>A	0.083333
TG	ZY.9G.00243	TRAV4	T cell receptor alpha variable 4	Chr9:32113648 C>T	0.083333
TG	ZY.9G.00245	Unknown	Immunoglobulin V-set domain	Chr9:32236392 A>G	0.090909
TG	ZY.9G.00288	RNASE9	Inactive ribonuclease-like protein 9	Chr9:33261465 C>T	0.083333
TG	ZY.9G.00473	MDGA2	MAM domain containing glycosylphosphatidylinositol anchor 2	Chr9:47422480 G>T	0.291667

TG	ZY.9G.00583	FAM114A2	Protein of unknown function (DUF719)	Chr9:64716481 G>A	0.083333
TG	ZY.9G.00718	RPL8	ribosomal protein	Chr9:82011580 A>G	0.333333
TG	ZY.9G.00755	RAD51B	DNA repair protein RAD51 homolog	Chr9:85868642 G>C	0.083333
TG	ZY.9G.00804	COQ6	ubiquinone biosynthesis monooxygenase	Chr9:91520657 T>A	0.375
TG	ZY.9G.00838	FLVCR2	Feline leukemia virus subgroup C receptor-related protein	Chr9:93107557 A>G	0.166667
TG	ZY.9G.00880	Unknown	Unknown	Chr9:105555655 C>T	0.125
TG	ZY.9G.00885	Unknown	Unknown	Chr9:105750794 T>A	0.375
TG	ZY.9G.00898	KCNK13	stabilization of membrane potential	Chr9:107825875 T>G	0.375
TG	ZY.10G.00065	RPL7	60S ribosomal protein	Chr10:16945372 C>T	0.125
TG	ZY.10G.00081	Unknown	Unknown	Chr10:22642369 G>A	0.291667
TG	ZY.10G.00108	IFNA5	Interferon	Chr10:28998128 T>C	0.5
TG	ZY.10G.00111	IFNA5	Interferon	Chr10:29035783 T>C	0.208333
TG	ZY.10G.00113	IFNA5	Interferon	Chr10:29057202 C>T	0.291667
TG	ZY.10G.00268	FAM205C	transmembrane protein ENSP00000340100 homolog	Chr10:65268015 G>A	0.083333
TG	ZY.10G.00372	SPATA31E1	spermatogenesis	Chr10:71638967 G>A	0.416667
TG	ZY.10G.00440	Unknown	Unknown	Chr10:80281579 G>A	0.5
TG	ZY.10G.00452	BAG1	BAG family molecular chaperone regulator 1	Chr10:80906769 A>G	0.333333
TG	ZY.10G.00482	PPFIA1	Protein tyrosine phosphatase, receptor type, f polypeptide (PTPRF), interacting protein (liprin), alpha 1	Chr10:82237554 C>T	0.083333
TG	ZY.10G.00720	SNTG2	Gamma-2-syntrophin	Chr10:117306790 C>T	0.458333
TG	ZY.10G.00728	PRDM9	Histone-lysine N-methyltransferase	Chr10:117847449 A>C	0.208333
TG	ZY.11G.00039	LYPLA1	Acyl-protein thioesterase 1	Chr11:28126433 C>T	0.25
TG	ZY.11G.00121	Unknown	Unknown	Chr11:43683769 C>A	0.125
TG	ZY.11G.00153	CCDC162P	Domain of unknown function (DUF4549)	Chr11:46224583 G>A	0.125
TG	ZY.11G.00224	GJA10	Gap junction protein, alpha 10	Chr11:65560192 T>C	0.125

TG	ZY.11G.00249	RPL10	embryonic brain development	Chr11:67946841 T>C	0.083333
TG	ZY.11G.00269	MTFR1	mitochondrial fission regulator 1	Chr11:73526601 G>A	0.375
TG	ZY.11G.00302	EYA4	EYA transcriptional coactivator and phosphatase 4	Chr11:76432311 G>T	0.083333
TG	ZY.11G.00309	CBX1	Chromobox protein homolog 1	Chr11:77710413 A>T	0.25
TG	ZY.11G.00334	CCDC28A	Coiled-coil domain containing 28A	Chr11:81425073 A>T	0.083333
TG	ZY.11G.00385	ULBP3	UL16 binding protein	Chr11:91733298 T>A	0.083333
TG	ZY.11G.00403	NCL	nucleolin	Chr11:94628548 C>A	0.083333
TG	ZY.11G.00429	SKP1	SCF-dependent proteasomal ubiquitin-dependent protein catabolic process	Chr11:99288971 C>T	0.583333
TG	ZY.11G.00441	MRPL18	ribosomal protein L18	Chr11:100335905 T>G	0.083333
TG	ZY.11G.00468	TCP10	T-complex protein 10 C-terminus	Chr11:106188484 G>A	0.166667
TG	ZY.11G.00472	Unknown	Unknown	Chr11:106373051 C>T	0.25
TG	ZY.12G.00012	ZFAND1	AN1-type zinc finger protein 1	Chr12:1400585 G>A	0.083333
TG	ZY.12G.00058	RPS23	maintenance of translational fidelity	Chr12:11339657 G>T	0.125
TG	ZY.12G.00157	SLC30A8	Solute carrier family 30	Chr12:33780358 G>A	0.166667
TG	ZY.12G.00349	ATF4	Transcription factor	Chr12:67131839 C>A	0.166667
TG	ZY.12G.00403	CYP11B1	cytochrome P450	Chr12:79790663 C>T	0.125
TG	ZY.12G.00404	CYP11B1	cytochrome P450	Chr12:79837279 G>A	0.458333
TG	ZY.12G.00411	Unknown	Unknown	Chr12:80138014 T>C	0.166667
TG	ZY.12G.00422	ZNF623	Zinc finger protein 623	Chr12:80302313 G>A	0.208333
TG	ZY.12G.00480	PPFIA1	Protein tyrosine phosphatase, receptor type, f polypeptide (PTPRF), interacting protein (liprin), alpha 1	Chr12:81261908 C>T	0.208333
TG	ZY.13G.00001	OR10AG1	Olfactory receptor	Chr13:16056448 G>A	0.25
TG	ZY.13G.00035	NUAK2	NUAK family, SNF1-like kinase, 2	Chr13:18562292 T>C	0.083333
TG	ZY.13G.00037	LEMD1	LEM domain-containing protein 1	Chr13:18666690 C>T	0.083333

TG	ZY.13G.00182	TMEM150A	Transmembrane protein 150A	Chr13:48125616 C>A	0.083333
TG	ZY.13G.00232	AADACL4	substituted 1 base at 1 genomic stop codon	Chr13:55179026 G>A	0.083333
TG	ZY.13G.00298	TNFRSF25	Tumor necrosis factor receptor superfamily member	Chr13:61028447 A>C	0.125
TG	ZY.14G.00264	AKR1C4	Dihydrodiol dehydrogenase 3-like	Chr14:52134311 T>C	0.208333
TG	ZY.14G.00334	PTPRA	Protein tyrosine phosphatase, receptor type A	Chr14:60056502 A>T	0.25
TG	ZY.14G.00352	SIRPA	Tyrosine-protein phosphatase non-receptor type substrate	Chr14:61226252 C>T	0.25
TG	ZY.14G.00428	PIEZO2	Piezo-type mechanosensitive ion channel component 2-like	Chr14:65328452 G>A; Chr14:65338050 G>A; Chr14:65345963 C>T	0.166667; 0.083333; 0.083333
TG	ZY.15G.00016	DCUN1D2	Defective in cullin neddylation 1 domain containing	Chr15:474319 G>A	0.125
TG	ZY.15G.00035	ARHGEF7	lamellipodium assembly	Chr15:1611641 A>G	0.083333
TG	ZY.15G.00099	ABCC4	Multidrug resistance-associated protein 4-like	Chr15:14923410 G>A	0.208333
TG	ZY.15G.00223	Unknown	WD domain, G-beta repeat	Chr15:51962087 C>T	0.25
TG	ZY.15G.00233	MDH1	Malate dehydrogenase	Chr15:53313689 T>C; Chr15:53314509 T>C	0.5; 0.25
TG	ZY.15G.00242	SOHLH2	Spermatogenesis- and oogenesis-specific basic helix-loop-helix-containing protein 2	Chr15:56547633 G>A	0.833333
TG	ZY.15G.00279	FAM124A	family with sequence similarity	Chr15:61106765 C>A	0.083333
TG	ZY.15G.00360	RPL30	selenocysteine insertion sequence binding	Chr15:79493782 T>C	0.166667
TG	ZY.16G.00039	RPS17	40S ribosomal protein S17-like	Chr16:10406772 T>C	0.416667
TG	ZY.16G.00051	LDHB	dehydrogenase B	Chr16:14154794 C>T	0.208333
TG	ZY.16G.00327	OR5P2	Olfactory receptor	Chr16:48165175 C>T	0.125
TG	ZY.16G.00331	Unknown	Olfactory receptor	Chr16:48220137 C>T	0.125
TG	ZY.16G.00423	OR52A5	Olfactory receptor	Chr16:50546774 A>G	0.125
TG	ZY.16G.00476	Unknown	Olfactory receptor	Chr16:51301832 T>C	0.166667
TG	ZY.16G.00548	MOGAT2	2-acylglycerol O-acyltransferase 2-like	Chr16:55202617 G>A	0.208333

TG	ZY.16G.00557	GUCY2F	guanylate cyclase activity	Chr16:56293638 A>G; Chr16:56303291 C>A	0.791667; 0.125
TG	ZY.16G.00588	Unknown	Unknown	Chr16:61519824 G>C	0.166667
TG	ZY.16G.00624	SLC1A2	solute carrier family 1	Chr16:65777764 C>T	0.083333
TG	ZY.16G.00637	ATP5H	Component of mitochondrial ATP synthase (Complex V) involved in proton-driven ATP production and structural stabilization of the enzyme complex.	Chr16:68319603 T>C	0.083333
TG	ZY.16G.00756	Unknown	Olfactory receptor	Chr16:78472257 A>G	0.625
TG	ZY.16G.00802	DNAJA1	homolog, subfamily A, member	Chr16:80252213 C>T	0.375
TG	ZY.16G.00810	OR4D9	Olfactory receptor	Chr16:80444138 C>T	0.125
TG	ZY.16G.00812	OSBP	Oxysterol-binding protein 1	Chr16:80496219 T>A	0.125
TG	ZY.16G.00819	OOSP1	oocyte-secreted protein	Chr16:80746999 G>A	0.083333
TG	ZY.17G.00166	TPST2	sulfotransferase 2	Chr17:6286079 G>T	0.416667
TG	ZY.17G.00297	BRAP	BRCA1 associated protein	Chr17:17182714 A>T	0.083333
TG	ZY.17G.00549	DFFA	DNA fragmentation factor	Chr17:64635241 T>A	0.125
TG	ZY.17G.00559	TLR2	toll-like receptor	Chr17:66584607 C>A	0.583333
TG	ZY.17G.00562	DCHS2	Dachsous cadherin-related 2	Chr17:67609848 G>A	0.083333
TG	ZY.18G.00019	MLKL	Mixed lineage kinase	Chr18:16245829 C>T	0.5
TG	ZY.18G.00152	CENPBD1	Tigger transposable element-derived protein 1-like	Chr18:28919005 C>T	0.5
TG	ZY.18G.00468	EI24	Etoposide-induced protein 2.4 homolog	Chr18:60020149 G>A	0.083333
TG	ZY.18G.00506	ZNF260	DNA-binding transcription factor activity	Chr18:60744641 G>A	0.208333
TG	ZY.18G.00577	Unknown	Galactoside-binding soluble	Chr18:62885952 G>A	0.375
TG	ZY.18G.00657	BSP5	metalloendopeptidase activity	Chr18:64925344 G>T	0.791667
TG	ZY.18G.00665	PINLYP	Phospholipase A2 inhibitor and Ly6 PLAUR domain-containing	Chr18:65096149 T>A	0.166667
TG	ZY.18G.00688	TAOK3	TAO kinase 3	Chr18:65631208 G>A	0.083333

TG	ZY.18G.00901	KLK6	Kallikrein-6	Chr18:70224411 A>T	0.125
TG	ZY.18G.00912	CD33	Myeloid cell surface antigen	Chr18:70373934 T>A	0.875
TG	ZY.18G.00927	CTU1	tRNA-modifying enzyme required for 2-thiolation of wobble uridines in specific tRNAs.	Chr18:70770591 C>A	0.541667
TG	ZY.18G.00942	Unknown	DNA-binding transcription factor activity	Chr18:71272092 C>A	0.125
TG	ZY.18G.00972	AU018091	Cationic amino acid transporter 3-like	Chr18:72876643 G>A	0.125
TG	ZY.18G.00973	UBQLN4	Ubiquilin 4	Chr18:72907750 C>T; Chr18:72907772 G>A; Chr18:72908372 T>C	0.333333; 0.25; 0.291667
TG	ZY.18G.01025	NLRP2	NACHT, LRR and PYD domains-containing protein	Chr18:73850734 C>A	0.416667
TG	ZY.18G.01030	KIR3DX1	Killer cell immunoglobulin-like receptor	Chr18:73963638 C>T	0.708333
TG	ZY.18G.01032	EIF4B	eukaryotic translation initiation factor	Chr18:74000823 C>T	0.083333
TG	ZY.18G.01050	OSCAR	immunoglobulin-like receptor	Chr18:74302075 G>A	0.166667
TG	ZY.18G.01056	ZNF444	Zinc finger protein 444	Chr18:74465026 G>C	0.125
TG	ZY.18G.01114	ZNF606	Zinc finger protein	Chr18:76121483 C>T	0.083333
TG	ZY.19G.00003	SDHA	Succinate dehydrogenase ubiquinone flavoprotein subunit	Chr19:115684 C>T	0.25
TG	ZY.19G.00079	WTAP	Wilms tumor 1 associated protein	Chr19:25899240 C>T	0.166667
TG	ZY.19G.00120	C5orf42	Chromosome 5 open reading frame 42	Chr19:32809437 C>G	0.208333
TG	ZY.19G.00208	RSRC2	Arginine serine-rich coiled-coil	Chr19:50846640 G>T	0.25
TG	ZY.19G.00247	CCDC125	Coiled-coil domain-containing protein 125	Chr19:59480953 G>C	0.083333
TG	ZY.19G.00278	ERH	enhancer of rudimentary homolog	Chr19:61908670 A>G	0.125
TG	ZY.20G.00018	Unknown	Unknown	Chr20:13407426 A>G	0.083333
TG	ZY.20G.00054	BTBD2	PHR domain	Chr20:24086406 C>G	0.166667
TG	ZY.20G.00160	Unknown	Unknown	Chr20:50311243 T>C	0.541667
TG	ZY.20G.00244	STARD6	stAR-related lipid transfer	Chr20:64594860 G>C; Chr20:64594877 G>A	0.833333; 0.833333

TG	ZY.21G.00054	TAMM41	Mitochondrial translocator assembly and maintenance protein 41 homolog	Chr21:3553752 T>A	0.5
TG	ZY.21G.00072	TRH	Regulates TSH biosynthesis and functions as a neurotransmitter and neuromodulator in the nervous system.	Chr21:4752886 G>A	0.25
TG	ZY.21G.00121	SETD2	Histone-lysine N-methyltransferase SETD2	Chr21:8162824 T>C	0.166667
TG	ZY.21G.00122	KIF9	Kinesin-like protein KIF9	Chr21:8253914 C>T	0.208333
TG	ZY.21G.00270	Unknown	Unknown	Chr21:14640612 T>C	0.083333
TG	ZY.21G.00520	Unknown	Unknown	Chr21:58152249 G>T	0.083333
TG	ZY.22G.00017	PCTP	Phosphatidylcholine transfer protein-like	Chr22:15391016 G>A	0.375
TG	ZY.22G.00033	OSTC	Oligosaccharyltransferase complex subunit	Chr22:17743053 C>T	0.125
TG	ZY.22G.00103	Wfdc18	Four-disulfide core domains	Chr22:23312548 G>A	0.083333
TG	ZY.22G.00106	Wfdc17	Four-disulfide core domains	Chr22:23406831 A>C	0.208333
TG	ZY.22G.00642	CACNB1	Voltage gated calcium channel subunit beta domain 4Aa N terminal	Chr22:48464349 T>A	0.083333
TG	ZY.22G.00643	CACNB1	Voltage gated calcium channel subunit beta domain 4Aa N terminal	Chr22:48464349 T>A	0.083333
TG	ZY.22G.00713	Unknown	Unknown	Chr22:50096280 C>G	0.083333
TG	ZY.22G.00735	KRT16	keratinocyte migration	Chr22:50508309 C>T	0.25
TG	ZY.22G.00896	Tcam1	Intercellular adhesion molecule (ICAM), N-terminal domain	Chr22:56245136 G>A	0.125
TG	ZY.22G.00901	ERN1	Endoplasmic reticulum to nucleus signaling 1	Chr22:56427692 A>G	0.833333
TG	ZY.22G.00979	ENTHD2	TEPSIN, adaptor related protein complex 4 accessory protein	Chr22:59345637 C>T	0.083333
TG	ZY.22G.01044	ATP5L	ATP synthase subunit g, mitochondrial-like	Chr22:63191141 A>G	0.666667
TG	ZY.22G.01071	KIAA0195	protein KIAA0195 homolog	Chr22:63764642 T>C	0.625
TG	ZY.22G.01122	Unknown	Unknown	Chr22:68092346 T>C	0.166667
TG	ZY.22G.01151	Unknown	Unknown	Chr22:70668728 A>G	0.916667

TG	ZY.23G.00011	Unknown	antigen binding	Chr23:316712 T>C	0.083333
TG	ZY.23G.00055	Unknown	Unknown	Chr23:2233359 C>T	0.166667
TG	ZY.23G.00066	CINP	kinase 2 interacting protein	Chr23:2974992 T>C	0.5
TG	ZY.23G.00077	Unknown	Unknown	Chr23:4362798 T>C	0.625
TG	ZY.23G.00132	CCDC197	Unknown	Chr23:11297462 T>G	0.083333
TG	ZY.23G.00188	EEF1A1	Elongation factor	Chr23:19290968 A>T	0.791667
TG	ZY.23G.00204	PPP1CC	Serine threonine-protein phosphatase PP1-gamma catalytic	Chr23:21786858 T>A	0.083333
TG	ZY.23G.00372	TNFRSF10A	TRAIL binding	Chr23:43452538 G>A	0.083333
TG	ZY.23G.00444	CKAP2	Cytoskeleton associated protein 2	Chr23:48084080 G>T	0.125
TG	ZY.23G.00484	FUS	FUS RNA binding protein	Chr23:58479594 C>T	0.166667
TG	ZY.23G.00486	FAM169B	Family with sequence similarity 169, member B	Chr23:60665674 C>A	0.083333
TG	ZY.24G.00012	EEF1A1	This protein promotes the GTP-dependent binding of aminoacyl-tRNA to the A-site of ribosomes during protein biosynthesis	Chr24:16668526 G>A	0.083333
TG	ZY.24G.00017	TBC1D22B	TBC1 domain family, member 22B	Chr24:18897787 T>G	0.458333
TG	ZY.24G.00026	TRIM43	Tripartite motif-containing protein 43-like	Chr24:20076151 C>T	0.791667
TG	ZY.24G.00033	TRIM43	Tripartite motif-containing protein 43-like	Chr24:20377073 T>A	0.166667
TG	ZY.24G.00278	Pga5	Belongs to the peptidase A1 family	Chr24:51273554 C>T	0.125
TG	ZY.24G.00346	SLC22A10	solute carrier family 22, member	Chr24:53159523 C>T	0.75
TG	ZY.24G.00351	SLC22A10	solute carrier family 22, member	Chr24:53422209 C>A	0.125
TG	ZY.24G.00518	Unknown	protein C11orf24 homolog	Chr24:57350161 A>G	0.75
TG	ZY.24G.00571	SYT8	Synaptotagmin VIII	Chr24:61149076 C>T	0.083333
TG	ZY.24G.00579	MUC5AC	stimulatory C-type lectin receptor signaling pathway	Chr24:61750403 C>T	0.333333
TG	ZY.25G.00021	CTAGE5	cargo loading into vesicle	Chr25:17321781 G>A	0.083333
TG	ZY.25G.00059	Unknown	Unknown	Chr25:23714128 T>C	0.083333
TG	ZY.25G.00104	OPALIN	Oligodendrocytic myelin paranodal and inner loop protein	Chr25:28197014 C>T	0.583333

TG	ZY.25G.00232	Unknown	Unknown	Chr25:41074081 C>A	0.166667
TG	ZY.25G.00295	HSPE1	10 kDa heat shock protein	Chr25:49277205 T>C	0.541667
TG	ZY.25G.00304	MCMBP	minichromosome maintenance	Chr25:49957985 C>T	0.125
TG	ZY.25G.00317	DMBT1	Deleted in malignant brain tumors 1	Chr25:52359681 G>A	0.583333
TG	ZY.25G.00369	Unknown	Unknown	Chr25:60531138 G>A	0.083333
TG	ZY.26G.00091	FANCE	Ring finger	Chr26:19807585 G>T	0.083333
TG	ZY.26G.00173	Unknown	Unknown	Chr26:26431926 C>T	0.125
TG	ZY.26G.00249	TNFRSF21	Tumor necrosis factor receptor superfamily member	Chr26:30735453 C>T	0.25
TG	ZY.26G.00270	PKHD1	Polycystic kidney and hepatic disease 1 (autosomal recessive)	Chr26:34100767 C>A	0.083333
TG	ZY.26G.00297	BTNL2	Butyrophilin-like	Chr26:35826777 C>T	0.125
TG	ZY.26G.00300	C6orf10	Chromosome 6 open reading frame 10	Chr26:35882303 T>A	0.625
TG	ZY.26G.00318	SKIV2L	helicase	Chr26:36609265 G>A; Chr26:36609771 G>A	0.5; 0.208333
TG	ZY.26G.00410	Unknown	olfactory	Chr26:38355018 C>T	0.083333
TG	ZY.26G.00459	Unknown	Zinc finger protein	Chr26:39328902 A>G	0.208333
TG	ZY.26G.00537	BLVRA	Biliverdin reductase A	Chr26:41347094 A>G	0.916667
TG	ZY.26G.00554	PRL	Prolactin-related protein	Chr26:43087970 A>T	0.125
TG	ZY.26G.00556	PRL	Prolactin-related protein	Chr26:43268040 C>T	0.25
TG	ZY.26G.00636	RPS15A	ribosomal protein	Chr26:56349842 G>C	0.083333
TG	ZY.27G.00027	Unknown	Unknown	Chr27:17285226 C>T	0.708333
TG	ZY.27G.00060	Unknown	Unknown	Chr27:23285396 A>G	0.375
TG	ZY.27G.00063	ZNF33B	Zinc finger protein	Chr27:24909652 G>A	0.166667
TG	ZY.27G.00065	RALGDS	small GTPase mediated signal transduction	Chr27:25020223 C>A; Chr27:25020304 G>A	0.772727; 0.166667
TG	ZY.27G.00101	RPS3A	40S ribosomal protein	Chr27:35560316 G>T	0.25

TG	ZY.27G.00278	MSMB	Microseminoprotein beta	Chr27:54501583 A>G	0.625
TG	ZY.28G.00023	Unknown	Unknown	Chr28:1052076 A>G	0.291667
TG	ZY.28G.00025	MAD1L1	Mitotic spindle assembly checkpoint protein	Chr28:1126420 A>G	0.916667
TG	ZY.28G.00335	APOBR	Apolipoprotein B receptor	Chr28:16148836 G>A	0.125
TG	ZY.28G.00352	Unknown	Endonuclease/Exonuclease/phosphatase family	Chr28:17132889 G>A	0.166667
TG	ZY.28G.00431	SMG1	Region of unknown function (DUF5303)	Chr28:25461627 T>A	0.083333
TG	ZY.28G.00651	Unknown	Zgc 112496	Chr28:41305330 G>T	0.25
TG	ZY.29G.00017	ZNF705D	nucleic acid-templated transcription	Chr29:11034663 G>A; Chr29:11077173 C>A	0.083333; 0.208333
TG	ZY.29G.00138	WRN	Werner syndrome	Chr29:31230027 T>C	0.291667
TG	ZY.29G.00157	Unknown	Unknown	Chr29:36495835 G>A	0.083333
TG	ZY.29G.00165	ADRB3	Beta-adrenergic receptor involved in catecholamine signaling, lipolysis, and thermogenesis.	Chr29:37185544 G>A	0.708333

Table S31. GO and KEGG enrichment of genes carrying LOF and predicted-deleterious variants in the three *Procapra* species.

**Species**	**Variant type**	**Category**	**GO/KEGG ID**	**Term names**	***P*-value**	***P*-adjust**	Gene number
MG	LOF	GO	GO:0006518	peptide metabolic process	0.00024654	0.375400392	33
MG	LOF	GO	GO:0001578	microtubule bundle formation	0.000337452	0.375400392	10
MG	LOF	GO	GO:0000226	microtubule cytoskeleton organization	0.0003668	0.375400392	29
MG	LOF	GO	GO:0006412	translation	0.000389243	0.375400392	28
MG	LOF	GO	GO:0007017	microtubule-based process	0.000457047	0.375400392	35
MG	LOF	GO	GO:0006417	regulation of translation	0.000552042	0.375400392	22
MG	LOF	GO	GO:0043043	peptide biosynthetic process	0.000632291	0.375400392	28
MG	LOF	GO	GO:1900119	positive regulation of execution phase of apoptosis	0.000857125	0.445276485	4
MG	LOF	GO	GO:0052547	regulation of peptidase activity	0.001298833	0.52279348	20
MG	LOF	GO	GO:0007018	microtubule-based movement	0.001470206	0.52279348	16
MG	LOF	GO	GO:0052548	regulation of endopeptidase activity	0.001520454	0.52279348	19
MG	LOF	GO	GO:0002181	cytoplasmic translation	0.001560655	0.52279348	16
MG	LOF	GO	GO:0010225	response to UV-C	0.001645113	0.52279348	4
MG	LOF	GO	GO:0061983	meiosis II cell cycle process	0.001761094	0.52279348	5
MG	LOF	GO	GO:0035082	axoneme assembly	0.002144476	0.561995365	7
MG	LOF	GO	GO:0002711	positive regulation of T cell mediated immunity	0.002369371	0.561995365	5
MG	LOF	GO	GO:0007111	meiosis II cytokinesis	0.002434051	0.561995365	3
MG	LOF	GO	GO:1903936	cellular response to sodium arsenite	0.002434051	0.561995365	3
MG	LOF	GO	GO:1902903	regulation of supramolecular fiber organization	0.003018595	0.630874494	19
MG	LOF	GO	GO:0007030	Golgi organization	0.003105197	0.630874494	10
MG	LOF	GO	GO:0045727	positive regulation of translation	0.004629291	0.699872823	11
MG	LOF	GO	GO:0006090	pyruvate metabolic process	0.004817236	0.699872823	7
MG	LOF	GO	GO:0044837	actomyosin contractile ring organization	0.005005636	0.699872823	4

MG	LOF	GO	GO:1903054	negative regulation of extracellular matrix organization	0.005143705	0.699872823	3
MG	LOF	GO	GO:0006891	intra-Golgi vesicle-mediated transport	0.005544308	0.699872823	4
MG	LOF	GO	GO:0002708	positive regulation of lymphocyte mediated immunity	0.005622712	0.699872823	7
MG	LOF	GO	GO:1903935	response to sodium arsenite	0.006018877	0.699872823	3
MG	LOF	GO	GO:0010608	post-transcriptional regulation of gene expression	0.006273157	0.699872823	24
MG	LOF	GO	GO:1902904	negative regulation of supramolecular fiber organization	0.006317722	0.699872823	10
MG	LOF	GO	GO:0002183	cytoplasmic translational initiation	0.006734813	0.699872823	4
MG	LOF	GO	GO:0007099	centriole replication	0.006734813	0.699872823	4
MG	LOF	GO	GO:0051497	negative regulation of stress fiber assembly	0.006734813	0.699872823	4
MG	LOF	GO	GO:0033119	negative regulation of RNA splicing	0.007049263	0.699872823	6
MG	LOF	GO	GO:0007110	meiosis I cytokinesis	0.008021853	0.699872823	3
MG	LOF	GO	GO:0007112	male meiosis cytokinesis	0.008083228	0.699872823	4
MG	LOF	GO	GO:0050913	sensory perception of bitter taste	0.008083228	0.699872823	4
MG	LOF	GO	GO:0002709	regulation of T cell mediated immunity	0.008769666	0.728934647	6
MG	LOF	GO	GO:1901224	positive regulation of non-canonical NF-kappaB signal transduction	0.008769666	0.728934647	6
MG	LOF	GO	GO:0002726	positive regulation of T cell cytokine production	0.009153126	0.730673095	3
MG	LOF	GO	GO:0010951	negative regulation of endopeptidase activity	0.009264645	0.730673095	11
MG	LOF	GO	GO:1900117	regulation of execution phase of apoptosis	0.009597488	0.730673095	4
MG	LOF	GO	GO:0032787	monocarboxylic acid metabolic process	0.009808663	0.730673095	20
MG	LOF	GO	GO:0009062	fatty acid catabolic process	0.009880409	0.730673095	7
PG	LOF	GO	GO:0061370	testosterone biosynthetic process	6.07E-05	1.73E-01	4
PG	LOF	GO	GO:0033119	negative regulation of RNA splicing	0.00010127	0.172643987	7
PG	LOF	GO	GO:0048024	regulation of mRNA splicing, via spliceosome	0.000241054	0.172643987	10
PG	LOF	GO	GO:0009142	nucleoside triphosphate biosynthetic process	0.000276936	0.172643987	8
PG	LOF	GO	GO:0018963	phthalate metabolic process	0.000285999	0.172643987	3

PG	LOF	GO	GO:0050684	regulation of mRNA processing	0.000321011	0.172643987	11
PG	LOF	GO	GO:0009156	ribonucleoside monophosphate biosynthetic process	0.000448253	0.172643987	8
PG	LOF	GO	GO:0006754	ATP biosynthetic process	0.000510896	0.172643987	7
PG	LOF	GO	GO:0009124	nucleoside monophosphate biosynthetic process	0.000612995	0.172643987	8
PG	LOF	GO	GO:0042447	hormone catabolic process	0.000623179	0.172643987	4
PG	LOF	GO	GO:0002220	innate immune response activating cell surface receptor signaling pathway	0.000631329	0.172643987	5
PG	LOF	GO	GO:0008209	androgen metabolic process	0.000685161	0.172643987	5
PG	LOF	GO	GO:0009206	purine ribonucleoside triphosphate biosynthetic process	0.000696366	0.172643987	7
PG	LOF	GO	GO:0009145	purine nucleoside triphosphate biosynthetic process	0.000731884	0.172643987	7
PG	LOF	GO	GO:0042181	ketone biosynthetic process	0.000803041	0.172643987	5
PG	LOF	GO	GO:0009201	ribonucleoside triphosphate biosynthetic process	0.000807219	0.172643987	7
PG	LOF	GO	GO:0009635	response to herbicide	0.00120729	0.226846279	4
PG	LOF	GO	GO:0001553	luteinization	0.001326849	0.226846279	3
PG	LOF	GO	GO:0071372	cellular response to follicle-stimulating hormone stimulus	0.001332374	0.226846279	4
PG	LOF	GO	GO:0002286	T cell activation involved in immune response	0.001436928	0.226846279	6
PG	LOF	GO	GO:0009127	purine nucleoside monophosphate biosynthetic process	0.001458392	0.226846279	7
PG	LOF	GO	GO:0009168	purine ribonucleoside monophosphate biosynthetic process	0.001458392	0.226846279	7
PG	LOF	GO	GO:0008300	isoprenoid catabolic process	0.00156135	0.232301762	3
PG	LOF	GO	GO:0043484	regulation of RNA splicing	0.001897337	0.235491422	10
PG	LOF	GO	GO:0046685	response to arsenic-containing substance	0.001971861	0.235491422	5
PG	LOF	GO	GO:0032354	response to follicle-stimulating hormone	0.002278245	0.235491422	4
PG	LOF	GO	GO:0071333	cellular response to glucose stimulus	0.002345924	0.235491422	6
PG	LOF	GO	GO:0031581	hemidesmosome assembly	0.002471376	0.235491422	4
PG	LOF	GO	GO:0071331	cellular response to hexose stimulus	0.002570355	0.235491422	6
PG	LOF	GO	GO:0008207	C21-steroid hormone metabolic process	0.002890823	0.235491422	4

PG	LOF	GO	GO:0071371	cellular response to gonadotropin stimulus	0.002890823	0.235491422	4
PG	LOF	GO	GO:0071326	cellular response to monosaccharide stimulus	0.003067161	0.235491422	6
PG	LOF	GO	GO:0030237	female sex determination	0.003116367	0.235491422	3
PG	LOF	GO	GO:0042593	glucose homeostasis	0.003190892	0.235491422	10
PG	LOF	GO	GO:0009260	ribonucleotide biosynthetic process	0.003324091	0.235491422	9
PG	LOF	GO	GO:0002223	stimulatory C-type lectin receptor signaling pathway	0.003356517	0.235491422	4
PG	LOF	GO	GO:0002218	activation of innate immune response	0.003491282	0.235491422	8
PG	LOF	GO	GO:0042574	retinal metabolic process	0.003509339	0.235491422	3
PG	LOF	GO	GO:0016071	mRNA metabolic process	0.003589046	0.235491422	21
PG	LOF	GO	GO:0046390	ribose phosphate biosynthetic process	0.003603713	0.235491422	9
PG	LOF	GO	GO:0006706	steroid catabolic process	0.003607444	0.235491422	4
PG	LOF	GO	GO:2000104	negative regulation of DNA-templated DNA replication	0.003931268	0.235491422	3
PG	LOF	GO	GO:0033500	carbohydrate homeostasis	0.004060197	0.235491422	10
PG	LOF	GO	GO:0007530	sex determination	0.004271996	0.243646154	5
PG	LOF	GO	GO:0006417	regulation of translation	0.004398581	0.244838142	14
PG	LOF	GO	GO:0048025	negative regulation of mRNA splicing, via spliceosome	0.004435992	0.244838142	4
PG	LOF	GO	GO:0008206	bile acid metabolic process	0.004738366	0.246349002	4
PG	LOF	GO	GO:0050892	intestinal absorption	0.004738366	0.246349002	4
PG	LOF	GO	GO:0015914	phospholipid transport	0.005181993	0.246349002	5
PG	LOF	GO	GO:0001678	intracellular glucose homeostasis	0.005183264	0.246349002	6
PG	LOF	GO	GO:0002253	activation of immune response	0.005430425	0.254560482	12
PG	LOF	GO	GO:0045727	positive regulation of translation	0.005530148	0.255731999	8
PG	LOF	GO	GO:0008585	female gonad development	0.006164162	0.258473373	7
PG	LOF	GO	GO:0071322	cellular response to carbohydrate stimulus	0.006230053	0.258473373	6
PG	LOF	GO	GO:1901654	response to ketone	0.006412237	0.258473373	12

PG	LOF	GO	GO:1904321	response to forskolin	0.006495824	0.258473373	3
PG	LOF	GO	GO:1904322	cellular response to forskolin	0.006495824	0.258473373	3
PG	LOF	GO	GO:0071236	cellular response to antibiotic	0.007019485	0.266243048	8
PG	LOF	GO	GO:0060856	establishment of blood-brain barrier	0.007103339	0.266243048	3
PG	LOF	GO	GO:0046545	development of primary female sexual characteristics	0.007369159	0.266243048	7
PG	LOF	GO	GO:2001025	positive regulation of response to drug	0.007667108	0.266243048	4
PG	LOF	GO	GO:0009152	purine ribonucleotide biosynthetic process	0.007968139	0.266307207	8
PG	LOF	GO	GO:0000381	regulation of alternative mRNA splicing, via spliceosome	0.008213314	0.266307207	6
PG	LOF	GO	GO:1904035	regulation of epithelial cell apoptotic process	0.008378917	0.266307207	5
PG	LOF	GO	GO:0061478	response to platelet aggregation inhibitor	0.008416517	0.266307207	3
PG	LOF	GO	GO:1902914	regulation of protein polyubiquitination	0.008416517	0.266307207	3
PG	LOF	GO	GO:0000184	nuclear-transcribed mRNA catabolic process, nonsense-mediated decay	0.008581804	0.266307207	8
PG	LOF	GO	GO:0033574	response to testosterone	0.008724486	0.266307207	5
PG	LOF	GO	GO:0006401	RNA catabolic process	0.008763978	0.266307207	11
PG	LOF	GO	GO:0097327	response to antineoplastic agent	0.009236738	0.266307207	7
PG	LOF	GO	GO:0002429	immune response-activating cell surface receptor signaling pathway	0.00968241	0.273159042	8
PG	LOF	GO	GO:0022600	digestive system process	0.009820128	0.273159042	5
PG	LOF	GO	GO:0002285	lymphocyte activation involved in immune response	0.009972694	0.273159042	6
TG	LOF	GO	GO:0032696	negative regulation of interleukin-13 production	8.75E-06	1.34E-02	4
TG	LOF	GO	GO:0032714	negative regulation of interleukin-5 production	8.75E-06	1.34E-02	4
TG	LOF	GO	GO:1902644	tertiary alcohol metabolic process	0.000207486	0.134552537	4
TG	LOF	GO	GO:0045087	innate immune response	0.000242884	0.134552537	20
TG	LOF	GO	GO:0006412	translation	0.000259428	0.134552537	22
TG	LOF	GO	GO:0032674	regulation of interleukin-5 production	0.000339696	0.134552537	4
TG	LOF	GO	GO:0000184	nuclear-transcribed mRNA catabolic process, nonsense-mediated decay	0.000342184	0.134552537	11

TG	LOF	GO	GO:0043043	peptide biosynthetic process	0.000389049	0.134552537	22
TG	LOF	GO	GO:0032656	regulation of interleukin-13 production	0.000394711	0.134552537	4
TG	LOF	GO	GO:0060337	type I interferon-mediated signaling pathway	0.000747877	0.195804241	5
TG	LOF	GO	GO:0071357	cellular response to type I interferon	0.000747877	0.195804241	5
TG	LOF	GO	GO:0042181	ketone biosynthetic process	0.000812569	0.195804241	5
TG	LOF	GO	GO:0006612	protein targeting to membrane	0.000829679	0.195804241	11
TG	LOF	GO	GO:2000552	negative regulation of T-helper 2 cell cytokine production	0.00089433	0.195986102	3
TG	LOF	GO	GO:0034340	response to type I interferon	0.001031777	0.211032788	5
TG	LOF	GO	GO:0045581	negative regulation of T cell differentiation	0.001388749	0.231434614	5
TG	LOF	GO	GO:0006518	peptide metabolic process	0.001527349	0.231434614	23
TG	LOF	GO	GO:0006413	translational initiation	0.001565443	0.231434614	11
TG	LOF	GO	GO:0006614	SRP-dependent cotranslational protein targeting to membrane	0.001758422	0.234558175	9
TG	LOF	GO	GO:0006613	cotranslational protein targeting to membrane	0.002001153	0.252384912	9
TG	LOF	GO	GO:0002725	negative regulation of T cell cytokine production	0.002135283	0.252384912	3
TG	LOF	GO	GO:0002828	regulation of type 2 immune response	0.002357508	0.252384912	4
TG	LOF	GO	GO:0001991	regulation of systemic arterial blood pressure by circulatory renin-angiotensin	0.002467306	0.252384912	3
TG	LOF	GO	GO:2000551	regulation of T-helper 2 cell cytokine production	0.002829538	0.269007932	3
TG	LOF	GO	GO:0006402	mRNA catabolic process	0.002856001	0.269007932	12
TG	LOF	GO	GO:0045047	protein targeting to ER	0.002893501	0.269007932	9
TG	LOF	GO	GO:0045620	negative regulation of lymphocyte differentiation	0.003005629	0.271213793	5
TG	LOF	GO	GO:0001504	neurotransmitter uptake	0.003222846	0.2736352	3
TG	LOF	GO	GO:0072599	establishment of protein localization to endoplasmic reticulum	0.00325235	0.2736352	9
TG	LOF	GO	GO:0050868	negative regulation of T cell activation	0.003358874	0.2736352	7
TG	LOF	GO	GO:0042180	ketone metabolic process	0.003389223	0.2736352	6
TG	LOF	GO	GO:0002829	negative regulation of type 2 immune response	0.003648046	0.284966856	3

TG	LOF	GO	GO:1901661	quinone metabolic process	0.003808227	0.284966856	4
TG	LOF	GO	GO:1903077	negative regulation of protein localization to plasma membrane	0.003808227	0.284966856	4
TG	LOF	GO	GO:0010225	response to UV-C	0.004105908	0.299926802	3
TG	LOF	GO	GO:0000956	nuclear-transcribed mRNA catabolic process	0.004323715	0.306905681	11
TG	LOF	GO	GO:1904376	negative regulation of protein localization to cell periphery	0.004401516	0.306905681	4
TG	LOF	GO	GO:1903038	negative regulation of leukocyte cell-cell adhesion	0.004804729	0.320454536	7
TG	LOF	GO	GO:0070972	protein localization to endoplasmic reticulum	0.005185732	0.338506925	9
TG	LOF	GO	GO:0034341	response to type II interferon	0.005334289	0.34095	8
TG	LOF	GO	GO:0046597	host-mediated suppression of symbiont invasion	0.005682457	0.35579136	3
TG	LOF	GO	GO:1904894	positive regulation of receptor signaling pathway via STAT	0.006043321	0.362842953	6
TG	LOF	GO	GO:0006401	RNA catabolic process	0.006065848	0.362842953	12
TG	LOF	GO	GO:0002823	negative regulation of adaptive immune response based on somatic recombination of immune receptors built from immunoglobulin superfamily domains	0.006149881	0.362842953	4
TG	LOF	GO	GO:1905476	negative regulation of protein localization to membrane	0.006961813	0.387401583	4
TG	LOF	GO	GO:0002820	negative regulation of adaptive immune response	0.007393089	0.387401583	4
TG	LOF	GO	GO:0003044	regulation of systemic arterial blood pressure mediated by a chemical signal	0.007393089	0.387401583	4
TG	LOF	GO	GO:0006094	gluconeogenesis	0.007393089	0.387401583	4
TG	LOF	GO	GO:0002710	negative regulation of T cell mediated immunity	0.007576302	0.387401583	3
TG	LOF	GO	GO:0033141	positive regulation of peptidyl-serine phosphorylation of STAT protein	0.007576302	0.387401583	3
TG	LOF	GO	GO:0045943	positive regulation of transcription by RNA polymerase I	0.008280563	0.414114372	3
TG	LOF	GO	GO:1904950	negative regulation of establishment of protein localization	0.008488813	0.414114372	9
TG	LOF	GO	GO:0033139	regulation of peptidyl-serine phosphorylation of STAT protein	0.009022064	0.414114372	3
TG	LOF	GO	GO:0051084	'de novo' post-translational protein folding	0.009022064	0.414114372	3
TG	LOF	GO	GO:0071346	cellular response to type II interferon	0.009043567	0.414114372	7
TG	LOF	GO	GO:0002791	regulation of peptide secretion	0.00948073	0.414416618	16

TG	LOF	GO	GO:0090150	establishment of protein localization to membrane	0.009485176	0.414416618	11
TG	LOF	GO	GO:0002323	natural killer cell activation involved in immune response	0.009801199	0.414416618	3
TG	LOF	GO	GO:0003081	regulation of systemic arterial blood pressure by renin-angiotensin	0.009801199	0.414416618	3
TG	LOF	GO	GO:0016137	glycoside metabolic process	0.009801199	0.414416618	3
TG	LOF	GO	GO:0051250	negative regulation of lymphocyte activation	0.00986063	0.414416618	7
MG	LOF	KEGG	ko05332	Graft-versus-host disease	0.000189122	0.030183625	7
MG	LOF	KEGG	ko04612	Antigen processing and presentation	0.00023581	0.030183625	9
MG	LOF	KEGG	ko05330	Allograft rejection	0.000363643	0.031030884	7
MG	LOF	KEGG	ko04940	Type I diabetes mellitus	0.000845448	0.054108693	7
MG	LOF	KEGG	ko03040	Spliceosome	0.001916486	0.085760229	12
MG	LOF	KEGG	ko05168	Herpes simplex virus 1 infection	0.002010005	0.085760229	16
MG	LOF	KEGG	ko00620	Pyruvate metabolism	0.007616251	0.233626375	5
MG	LOF	KEGG	ko03010	Ribosome	0.007745264	0.233626375	19
MG	LOF	KEGG	ko05416	Viral myocarditis	0.008849726	0.233626375	7
MG	LOF	KEGG	ko00720	Other carbon fixation pathways	0.00912603	0.233626375	3
PG	LOF	KEGG	ko05169	Epstein-Barr virus infection	4.78E-05	1.05E-02	14
PG	LOF	KEGG	ko05144	Malaria	0.001225824	0.134840615	6
PG	LOF	KEGG	ko03015	mRNA surveillance pathway	0.003333718	0.244472632	7
TG	LOF	KEGG	ko03010	Ribosome	0.001391615	0.228398423	18
TG	LOF	KEGG	ko04217	Necroptosis	0.001805521	0.228398423	12
MG	DEL	GO	GO:0050907	detection of chemical stimulus involved in sensory perception	2.38E-46	2.00E-42	207
MG	DEL	GO	GO:0009593	detection of chemical stimulus	2.60E-42	1.09E-38	221
MG	DEL	GO	GO:0050911	detection of chemical stimulus involved in sensory perception of smell	1.92E-38	5.40E-35	156
MG	DEL	GO	GO:0007606	sensory perception of chemical stimulus	5.30E-37	1.12E-33	232
MG	DEL	GO	GO:0050906	detection of stimulus involved in sensory perception	2.51E-35	4.23E-32	224

MG	DEL	GO	GO:0007608	sensory perception of smell	1.27E-32	1.78E-29	169
MG	DEL	GO	GO:0051606	detection of stimulus	8.36E-27	1.01E-23	283
MG	DEL	GO	GO:0002181	cytoplasmic translation	1.37E-13	1.28E-10	139
MG	DEL	GO	GO:0043043	peptide biosynthetic process	2.00E-13	1.68E-10	249
MG	DEL	GO	GO:0006412	translation	3.31E-13	2.53E-10	242
MG	DEL	GO	GO:0042273	ribosomal large subunit biogenesis	5.53E-09	3.33E-06	72
MG	DEL	GO	GO:0000027	ribosomal large subunit assembly	2.90E-08	1.63E-05	42
MG	DEL	GO	GO:0042255	ribosome assembly	7.38E-08	3.88E-05	60
MG	DEL	GO	GO:0070972	protein localization to endoplasmic reticulum	1.11E-06	5.20E-04	88
MG	DEL	GO	GO:0072599	establishment of protein localization to endoplasmic reticulum	2.30E-06	9.68E-04	82
MG	DEL	GO	GO:0006413	translational initiation	2.93E-06	1.16E-03	98
MG	DEL	GO	GO:0036100	leukotriene catabolic process	3.31E-06	1.16E-03	10
MG	DEL	GO	GO:0036101	leukotriene B4 catabolic process	3.31E-06	1.16E-03	10
MG	DEL	GO	GO:0036102	leukotriene B4 metabolic process	3.31E-06	1.16E-03	10
MG	DEL	GO	GO:0045047	protein targeting to ER	4.09E-06	1.38E-03	80
MG	DEL	GO	GO:0002093	auditory receptor cell morphogenesis	7.42E-06	2.40E-03	15
MG	DEL	GO	GO:0006614	SRP-dependent cotranslational protein targeting to membrane	8.49E-06	2.65E-03	74
MG	DEL	GO	GO:0060088	auditory receptor cell stereocilium organization	9.47E-06	2.85E-03	14
MG	DEL	GO	GO:0006612	protein targeting to membrane	1.23E-05	3.58E-03	90
MG	DEL	GO	GO:0006613	cotranslational protein targeting to membrane	2.23E-05	6.25E-03	74
MG	DEL	GO	GO:1901569	fatty acid derivative catabolic process	3.54E-05	9.60E-03	15
MG	DEL	GO	GO:1901523	icosanoid catabolic process	4.64E-05	1.22E-02	11
MG	DEL	GO	GO:1904714	regulation of chaperone-mediated autophagy	0.000117616	0.028278294	11
MG	DEL	GO	GO:0032305	positive regulation of icosanoid secretion	0.000159544	0.037293388	16
MG	DEL	GO	GO:0002449	lymphocyte mediated immunity	0.000187763	0.042703305	48

MG	DEL	GO	GO:0042758	long-chain fatty acid catabolic process	0.000264363	0.057973213	11
MG	DEL	GO	GO:0060117	auditory receptor cell development	0.000439874	0.092538429	16
MG	DEL	GO	GO:0060122	inner ear receptor cell stereocilium organization	0.000513922	0.105479455	19
MG	DEL	GO	GO:0000056	ribosomal small subunit export from nucleus	0.000539578	0.108108255	11
MG	DEL	GO	GO:1905258	regulation of nitrosative stress-induced intrinsic apoptotic signaling pathway	0.000613181	0.110544934	8
MG	DEL	GO	GO:1905259	negative regulation of nitrosative stress-induced intrinsic apoptotic signaling pathway	0.000613181	0.110544934	8
MG	DEL	GO	GO:0003095	pressure natriuresis	0.000617423	0.110544934	5
MG	DEL	GO	GO:0048252	lauric acid metabolic process	0.000617423	0.110544934	5
MG	DEL	GO	GO:2001189	negative regulation of T cell activation via T cell receptor contact with antigen bound to MHC molecule on antigen presenting cell	0.000617423	0.110544934	5
MG	DEL	GO	GO:0033119	negative regulation of RNA splicing	0.000652459	0.114384164	33
MG	DEL	GO	GO:2000193	positive regulation of fatty acid transport	0.000694518	0.116466501	16
MG	DEL	GO	GO:0002455	humoral immune response mediated by circulating immunoglobulin	0.000702828	0.116466501	12
MG	DEL	GO	GO:0061015	snRNA import into nucleus	0.000740926	0.117649413	7
MG	DEL	GO	GO:0016052	carbohydrate catabolic process	0.000740989	0.117649413	50
MG	DEL	GO	GO:0018119	peptidyl-cysteine S-nitrosylation	0.000761788	0.118711936	10
MG	DEL	GO	GO:0032310	prostaglandin secretion	0.000792755	0.121291488	6
MG	DEL	GO	GO:0002228	natural killer cell mediated immunity	0.001017047	0.145058502	15
MG	DEL	GO	GO:1903936	cellular response to sodium arsenite	0.001065963	0.149501381	9
MG	DEL	GO	GO:0006691	leukotriene metabolic process	0.001089486	0.150295542	17
MG	DEL	GO	GO:0045494	photoreceptor cell maintenance	0.001413344	0.191827203	26
MG	DEL	GO	GO:0051030	snRNA transport	0.001470656	0.193596887	8
MG	DEL	GO	GO:0017014	protein nitrosylation	0.001472395	0.193596887	10
MG	DEL	GO	GO:0032303	regulation of icosanoid secretion	0.0015889	0.205701498	16
MG	DEL	GO	GO:0007095	mitotic G2 DNA damage checkpoint signaling	0.001830337	0.229884887	22

MG	DEL	GO	GO:0002309	T cell proliferation involved in immune response	0.001981253	0.231558974	7
MG	DEL	GO	GO:0002933	lipid hydroxylation	0.001981253	0.231558974	7
MG	DEL	GO	GO:0006404	RNA import into nucleus	0.001981253	0.231558974	7
MG	DEL	GO	GO:2000586	regulation of platelet-derived growth factor receptor-beta signaling pathway	0.001981253	0.231558974	7
MG	DEL	GO	GO:0007210	serotonin receptor signaling pathway	0.002209938	0.244692491	18
MG	DEL	GO	GO:0000463	maturation of LSU-rRNA from tricistronic rRNA transcript (SSU-rRNA, 5.8S rRNA, LSU-rRNA)	0.002313328	0.250310965	16
MG	DEL	GO	GO:0046112	nucleobase biosynthetic process	0.002320173	0.250310965	15
MG	DEL	GO	GO:1905515	non-motile cilium assembly	0.00254718	0.262163914	25
MG	DEL	GO	GO:0070493	thrombin-activated receptor signaling pathway	0.002554657	0.262163914	6
MG	DEL	GO	GO:2000587	negative regulation of platelet-derived growth factor receptor-beta signaling pathway	0.002554657	0.262163914	6
MG	DEL	GO	GO:1903748	negative regulation of protein localization to mitochondrion	0.002638416	0.266142294	10
MG	DEL	GO	GO:1903793	positive regulation of monoatomic anion transport	0.002656679	0.266142294	30
MG	DEL	GO	GO:0000724	double-strand break repair via homologous recombination	0.002743188	0.271575579	40
MG	DEL	GO	GO:0032892	positive regulation of organic acid transport	0.002967167	0.280575568	23
MG	DEL	GO	GO:0042361	menaquinone catabolic process	0.003000808	0.280575568	5
MG	DEL	GO	GO:0042377	vitamin K catabolic process	0.003000808	0.280575568	5
MG	DEL	GO	GO:0050912	detection of chemical stimulus involved in sensory perception of taste	0.003052958	0.282314706	18
MG	DEL	GO	GO:0016233	telomere capping	0.003354843	0.291041239	13
MG	DEL	GO	GO:0098581	detection of external biotic stimulus	0.00339281	0.291331617	14
MG	DEL	GO	GO:0001895	retina homeostasis	0.003604579	0.306389177	29
MG	DEL	GO	GO:0044818	mitotic G2/M transition checkpoint	0.003665565	0.308457329	20
MG	DEL	GO	GO:2000182	regulation of progesterone biosynthetic process	0.003880302	0.32012494	9
MG	DEL	GO	GO:0031122	cytoplasmic microtubule organization	0.004323354	0.349817509	27
MG	DEL	GO	GO:0006409	tRNA export from nucleus	0.00437538	0.350655421	7

MG	DEL	GO	GO:1903935	response to sodium arsenite	0.004439138	0.352408959	10
MG	DEL	GO	GO:0001580	detection of chemical stimulus involved in sensory perception of bitter taste	0.004769658	0.365369367	15
MG	DEL	GO	GO:0032309	icosanoid secretion	0.00477607	0.365369367	11
MG	DEL	GO	GO:2000781	positive regulation of double-strand break repair	0.00477607	0.365369367	11
MG	DEL	GO	GO:0001539	cilium or flagellum-dependent cell motility	0.004969274	0.373361071	13
MG	DEL	GO	GO:0060285	cilium-dependent cell motility	0.004969274	0.373361071	13
MG	DEL	GO	GO:1902229	regulation of intrinsic apoptotic signaling pathway in response to DNA damage	0.005176934	0.382139493	25
MG	DEL	GO	GO:0000740	nuclear membrane fusion	0.00618212	0.429938349	6
MG	DEL	GO	GO:0035281	pre-miRNA export from nucleus	0.00618212	0.429938349	6
MG	DEL	GO	GO:0085020	protein K6-linked ubiquitination	0.00618212	0.429938349	6
MG	DEL	GO	GO:0097267	omega-hydroxylase P450 pathway	0.00618212	0.429938349	6
MG	DEL	GO	GO:1990428	miRNA transport	0.00618212	0.429938349	6
MG	DEL	GO	GO:2001034	positive regulation of double-strand break repair via nonhomologous end joining	0.00618212	0.429938349	6
MG	DEL	GO	GO:0051497	negative regulation of stress fiber assembly	0.006272125	0.432622423	16
MG	DEL	GO	GO:0046456	icosanoid biosynthetic process	0.006752773	0.455574594	22
MG	DEL	GO	GO:1901661	quinone metabolic process	0.006767299	0.455574594	19
MG	DEL	GO	GO:0071364	cellular response to epidermal growth factor stimulus	0.007236142	0.472675557	26
MG	DEL	GO	GO:0006011	UDP-alpha-D-glucose metabolic process	0.007288577	0.472675557	11
MG	DEL	GO	GO:0042182	ketone catabolic process	0.007291448	0.472675557	12
MG	DEL	GO	GO:0032232	negative regulation of actin filament bundle assembly	0.007302177	0.472675557	18
MG	DEL	GO	GO:0002526	acute inflammatory response	0.007877089	0.505997756	31
MG	DEL	GO	GO:0032635	interleukin-6 production	0.008441992	0.515854133	7
MG	DEL	GO	GO:1904076	regulation of estrogen biosynthetic process	0.008441992	0.515854133	7
MG	DEL	GO	GO:1904077	negative regulation of estrogen biosynthetic process	0.008441992	0.515854133	7
MG	DEL	GO	GO:2000183	negative regulation of progesterone biosynthetic process	0.008441992	0.515854133	7

MG	DEL	GO	GO:0009112	nucleobase metabolic process	0.008491232	0.515854133	22
MG	DEL	GO	GO:0009233	menaquinone metabolic process	0.008520942	0.515854133	5
MG	DEL	GO	GO:0009589	detection of UV	0.008520942	0.515854133	5
MG	DEL	GO	GO:0035608	protein deglutamylation	0.008520942	0.515854133	5
MG	DEL	GO	GO:0009595	detection of biotic stimulus	0.009526517	0.560817921	14
MG	DEL	GO	GO:0071421	manganese ion transmembrane transport	0.009822765	0.560817921	8
PG	DEL	GO	GO:0050907	detection of chemical stimulus involved in sensory perception	5.95E-34	4.75E-30	159
PG	DEL	GO	GO:0009593	detection of chemical stimulus	2.73E-32	1.09E-28	171
PG	DEL	GO	GO:0050911	detection of chemical stimulus involved in sensory perception of smell	1.51E-29	4.02E-26	122
PG	DEL	GO	GO:0050906	detection of stimulus involved in sensory perception	3.60E-29	7.18E-26	177
PG	DEL	GO	GO:0007606	sensory perception of chemical stimulus	9.45E-29	1.51E-25	179
PG	DEL	GO	GO:0007608	sensory perception of smell	3.08E-26	4.10E-23	133
PG	DEL	GO	GO:0051606	detection of stimulus	1.47E-23	1.68E-20	224
PG	DEL	GO	GO:0007600	sensory perception	5.65E-17	5.63E-14	271
PG	DEL	GO	GO:0042254	ribosome biogenesis	6.08E-09	5.38E-06	119
PG	DEL	GO	GO:0006364	rRNA processing	3.79E-07	2.32E-04	81
PG	DEL	GO	GO:0002181	cytoplasmic translation	4.94E-07	2.63E-04	94
PG	DEL	GO	GO:1902644	tertiary alcohol metabolic process	2.11E-06	9.90E-04	14
PG	DEL	GO	GO:0016072	rRNA metabolic process	2.46E-06	1.09E-03	86
PG	DEL	GO	GO:0042273	ribosomal large subunit biogenesis	3.79E-06	1.51E-03	53
PG	DEL	GO	GO:0030638	polyketide metabolic process	6.74E-06	2.34E-03	12
PG	DEL	GO	GO:0044597	daunorubicin metabolic process	6.74E-06	2.34E-03	12
PG	DEL	GO	GO:0044598	doxorubicin metabolic process	6.74E-06	2.34E-03	12
PG	DEL	GO	GO:0002309	T cell proliferation involved in immune response	5.82E-05	1.86E-02	8
PG	DEL	GO	GO:0016137	glycoside metabolic process	8.07E-05	2.38E-02	16

PG	DEL	GO	GO:1900454	positive regulation of long-term synaptic depression	9.03E-05	2.57E-02	7
PG	DEL	GO	GO:0042274	ribosomal small subunit biogenesis	0.000115108	0.031658734	37
PG	DEL	GO	GO:0042255	ribosome assembly	0.000131397	0.034933951	42
PG	DEL	GO	GO:0030490	maturation of SSU-rRNA	0.000137129	0.035281961	26
PG	DEL	GO	GO:1901661	quinone metabolic process	0.000308658	0.072407544	19
PG	DEL	GO	GO:0002449	lymphocyte mediated immunity	0.000335024	0.074328768	37
PG	DEL	GO	GO:0070972	protein localization to endoplasmic reticulum	0.000335486	0.074328768	61
PG	DEL	GO	GO:0001578	microtubule bundle formation	0.000426051	0.084954485	38
PG	DEL	GO	GO:0002455	humoral immune response mediated by circulating immunoglobulin	0.000490749	0.091312531	11
PG	DEL	GO	GO:0016045	detection of bacterium	0.000490749	0.091312531	11
PG	DEL	GO	GO:0000027	ribosomal large subunit assembly	0.0005032	0.091312531	27
PG	DEL	GO	GO:0072599	establishment of protein localization to endoplasmic reticulum	0.00050373	0.091312531	57
PG	DEL	GO	GO:0045047	protein targeting to ER	0.000557688	0.098847042	56
PG	DEL	GO	GO:1904714	regulation of chaperone-mediated autophagy	0.000634251	0.109973622	9
PG	DEL	GO	GO:0000462	maturation of SSU-rRNA from tricistronic rRNA transcript (SSU-rRNA, 5.8S rRNA, LSU-rRNA)	0.000708901	0.120301967	19
PG	DEL	GO	GO:0006614	SRP-dependent cotranslational protein targeting to membrane	0.000743383	0.123525491	52
PG	DEL	GO	GO:0098543	detection of other organism	0.000770973	0.125495519	11
PG	DEL	GO	GO:0009595	detection of biotic stimulus	0.000826461	0.129252015	14
PG	DEL	GO	GO:0003341	cilium movement	0.001052755	0.155056951	25
PG	DEL	GO	GO:0098581	detection of external biotic stimulus	0.001061802	0.155056951	13
PG	DEL	GO	GO:0009313	oligosaccharide catabolic process	0.001082518	0.155056951	9
PG	DEL	GO	GO:0016204	determination of muscle attachment site	0.001082518	0.155056951	9
PG	DEL	GO	GO:0000740	nuclear membrane fusion	0.00110125	0.155056951	6
PG	DEL	GO	GO:0008207	C21-steroid hormone metabolic process	0.001223905	0.163176796	17

PG	DEL	GO	GO:0009608	response to symbiont	0.001247967	0.163176796	7
PG	DEL	GO	GO:0016487	farnesol metabolic process	0.001247967	0.163176796	7
PG	DEL	GO	GO:0016488	farnesol catabolic process	0.001247967	0.163176796	7
PG	DEL	GO	GO:0006613	cotranslational protein targeting to membrane	0.001335183	0.171764833	52
PG	DEL	GO	GO:0060122	inner ear receptor cell stereocilium organization	0.001605936	0.20331656	15
PG	DEL	GO	GO:0050764	regulation of phagocytosis	0.001858269	0.231586757	38
PG	DEL	GO	GO:0006013	mannose metabolic process	0.002317842	0.265855309	7
PG	DEL	GO	GO:0016095	polyprenol catabolic process	0.002317842	0.265855309	7
PG	DEL	GO	GO:0016107	sesquiterpenoid catabolic process	0.002317842	0.265855309	7
PG	DEL	GO	GO:0035606	peptidyl-cysteine S-trans-nitrosylation	0.002366566	0.265855309	6
PG	DEL	GO	GO:0048003	antigen processing and presentation of lipid antigen via MHC class Ib	0.002366566	0.265855309	6
PG	DEL	GO	GO:0048007	antigen processing and presentation, exogenous lipid antigen via MHC class Ib	0.002366566	0.265855309	6
PG	DEL	GO	GO:0050892	intestinal absorption	0.002477166	0.274414964	18
PG	DEL	GO	GO:0040010	positive regulation of growth rate	0.002674583	0.289948957	12
PG	DEL	GO	GO:0032611	interleukin-1 beta production	0.002726451	0.289948957	9
PG	DEL	GO	GO:0097211	cellular response to gonadotropin-releasing hormone	0.002726451	0.289948957	9
PG	DEL	GO	GO:0022600	digestive system process	0.002974263	0.312141026	29
PG	DEL	GO	GO:0050766	positive regulation of phagocytosis	0.00308421	0.319476135	27
PG	DEL	GO	GO:1904592	positive regulation of protein refolding	0.003275303	0.322516233	4
PG	DEL	GO	GO:1904764	chaperone-mediated autophagy translocation complex disassembly	0.003275303	0.322516233	4
PG	DEL	GO	GO:1905875	negative regulation of postsynaptic density organization	0.003275303	0.322516233	4
PG	DEL	GO	GO:0051315	attachment of mitotic spindle microtubules to kinetochore	0.003342225	0.325092563	8
PG	DEL	GO	GO:0042448	progesterone metabolic process	0.00348818	0.331210956	11
PG	DEL	GO	GO:0060856	establishment of blood-brain barrier	0.003642251	0.338113705	12
PG	DEL	GO	GO:0006699	bile acid biosynthetic process	0.003653415	0.338113705	14

PG	DEL	GO	GO:0051084	'de novo' post-translational protein folding	0.003688051	0.338113705	13
PG	DEL	GO	GO:0071421	manganese ion transmembrane transport	0.003975808	0.353402503	7
PG	DEL	GO	GO:0045494	photoreceptor cell maintenance	0.004049952	0.353402503	20
PG	DEL	GO	GO:0002577	regulation of antigen processing and presentation	0.004076358	0.353402503	9
PG	DEL	GO	GO:0032612	interleukin-1 production	0.004076358	0.353402503	9
PG	DEL	GO	GO:0097210	response to gonadotropin-releasing hormone	0.004076358	0.353402503	9
PG	DEL	GO	GO:0006709	progesterone catabolic process	0.004478159	0.358635404	6
PG	DEL	GO	GO:0008208	C21-steroid hormone catabolic process	0.004478159	0.358635404	6
PG	DEL	GO	GO:0009753	response to jasmonic acid	0.004478159	0.358635404	6
PG	DEL	GO	GO:0045827	negative regulation of isoprenoid metabolic process	0.004478159	0.358635404	6
PG	DEL	GO	GO:0071395	cellular response to jasmonic acid stimulus	0.004478159	0.358635404	6
PG	DEL	GO	GO:0072318	clathrin coat disassembly	0.004478159	0.358635404	6
PG	DEL	GO	GO:1900053	negative regulation of retinoic acid biosynthetic process	0.004478159	0.358635404	6
PG	DEL	GO	GO:1903416	response to glycoside	0.004496432	0.358635404	5
PG	DEL	GO	GO:0007094	mitotic spindle assembly checkpoint signaling	0.004789438	0.363906359	11
PG	DEL	GO	GO:0031577	spindle checkpoint signaling	0.004789438	0.363906359	11
PG	DEL	GO	GO:0071173	spindle assembly checkpoint signaling	0.004789438	0.363906359	11
PG	DEL	GO	GO:0071174	mitotic spindle checkpoint signaling	0.004789438	0.363906359	11
PG	DEL	GO	GO:0008608	attachment of spindle microtubules to kinetochore	0.004836268	0.363906359	13
PG	DEL	GO	GO:0031116	positive regulation of microtubule polymerization	0.004836268	0.363906359	13
PG	DEL	GO	GO:0035563	positive regulation of chromatin binding	0.005149992	0.383890974	8
PG	DEL	GO	GO:0019724	B cell mediated immunity	0.005373013	0.396806953	21
PG	DEL	GO	GO:0042181	ketone biosynthetic process	0.005751355	0.419883022	18
PG	DEL	GO	GO:0002093	auditory receptor cell morphogenesis	0.00589605	0.419883022	9
PG	DEL	GO	GO:0006828	manganese ion transport	0.00589605	0.419883022	9

PG	DEL	GO	GO:0060857	establishment of glial blood-brain barrier	0.00589605	0.419883022	9
PG	DEL	GO	GO:0035082	axoneme assembly	0.005980637	0.422137733	25
PG	DEL	GO	GO:0006458	'de novo' protein folding	0.006207731	0.433790072	17
PG	DEL	GO	GO:0006829	zinc ion transport	0.006254496	0.433790072	13
PG	DEL	GO	GO:2000224	regulation of testosterone biosynthetic process	0.00639687	0.439492228	7
PG	DEL	GO	GO:0045841	negative regulation of mitotic metaphase/anaphase transition	0.006446915	0.439492228	11
PG	DEL	GO	GO:0019369	arachidonate metabolic process	0.007297533	0.480326675	23
PG	DEL	GO	GO:0006910	phagocytosis, recognition	0.007618444	0.480326675	8
PG	DEL	GO	GO:0032483	regulation of Rab protein signal transduction	0.007618444	0.480326675	8
PG	DEL	GO	GO:0060119	inner ear receptor cell development	0.007671225	0.480326675	19
PG	DEL	GO	GO:0002579	positive regulation of antigen processing and presentation	0.007708352	0.480326675	6
PG	DEL	GO	GO:0052042	symbiont-mediated activation of host programmed cell death	0.007708352	0.480326675	6
PG	DEL	GO	GO:0061083	regulation of protein refolding	0.007708352	0.480326675	6
PG	DEL	GO	GO:0072319	vesicle uncoating	0.007708352	0.480326675	6
PG	DEL	GO	GO:1900052	regulation of retinoic acid biosynthetic process	0.007708352	0.480326675	6
PG	DEL	GO	GO:0032351	negative regulation of hormone metabolic process	0.007984357	0.489871003	13
PG	DEL	GO	GO:0072499	photoreceptor cell axon guidance	0.007984357	0.489871003	13
PG	DEL	GO	GO:0042180	ketone metabolic process	0.008164446	0.490940131	30
PG	DEL	GO	GO:0016064	immunoglobulin mediated immune response	0.008312573	0.490940131	20
PG	DEL	GO	GO:0071577	zinc ion transmembrane transport	0.008313722	0.490940131	12
PG	DEL	GO	GO:1902100	negative regulation of metaphase/anaphase transition of cell cycle	0.008521697	0.490940131	11
PG	DEL	GO	GO:0033212	iron import into cell	0.008532039	0.490940131	4
PG	DEL	GO	GO:0097214	positive regulation of lysosomal membrane permeability	0.008532039	0.490940131	4
PG	DEL	GO	GO:0002933	lipid hydroxylation	0.008740409	0.490940131	5
PG	DEL	GO	GO:0046462	monoacylglycerol metabolic process	0.008740409	0.490940131	5

PG	DEL	GO	GO:0071211	protein targeting to vacuole involved in autophagy	0.008740409	0.490940131	5
PG	DEL	GO	GO:1902946	protein localization to early endosome	0.008740409	0.490940131	5
PG	DEL	GO	GO:0030449	regulation of complement activation	0.008820994	0.492001717	15
PG	DEL	GO	GO:0010566	regulation of ketone biosynthetic process	0.009456552	0.519176933	14
PG	DEL	GO	GO:0006714	sesquiterpenoid metabolic process	0.009763859	0.519176933	7
PG	DEL	GO	GO:0016115	terpenoid catabolic process	0.009763859	0.519176933	7
PG	DEL	GO	GO:0021682	nerve maturation	0.009763859	0.519176933	7
PG	DEL	GO	GO:0034982	mitochondrial protein processing	0.009763859	0.519176933	7
PG	DEL	GO	GO:0046137	negative regulation of vitamin metabolic process	0.009763859	0.519176933	7
TG	DEL	GO	GO:0050907	detection of chemical stimulus involved in sensory perception	1.42E-38	1.17E-34	181
TG	DEL	GO	GO:0009593	detection of chemical stimulus	3.21E-35	1.32E-31	192
TG	DEL	GO	GO:0007606	sensory perception of chemical stimulus	5.81E-30	1.59E-26	199
TG	DEL	GO	GO:0050906	detection of stimulus involved in sensory perception	1.05E-29	2.15E-26	195
TG	DEL	GO	GO:0050911	detection of chemical stimulus involved in sensory perception of smell	4.92E-28	8.09E-25	131
TG	DEL	GO	GO:0007608	sensory perception of smell	1.11E-23	1.52E-20	141
TG	DEL	GO	GO:0051606	detection of stimulus	5.85E-22	6.87E-19	242
TG	DEL	GO	GO:0060271	cilium assembly	5.92E-09	5.41E-06	125
TG	DEL	GO	GO:0044782	cilium organization	2.06E-08	1.70E-05	127
TG	DEL	GO	GO:0001539	cilium or flagellum-dependent cell motility	1.50E-06	1.03E-03	17
TG	DEL	GO	GO:0060285	cilium-dependent cell motility	1.50E-06	1.03E-03	17
TG	DEL	GO	GO:0001578	microtubule bundle formation	2.20E-06	1.29E-03	49
TG	DEL	GO	GO:0003341	cilium movement	2.67E-06	1.37E-03	34
TG	DEL	GO	GO:0035082	axoneme assembly	3.22E-06	1.56E-03	37
TG	DEL	GO	GO:2000359	regulation of binding of sperm to zona pellucida	9.48E-06	3.78E-03	7
TG	DEL	GO	GO:0034341	response to type II interferon	0.000120693	0.034235284	61

TG	DEL	GO	GO:2000360	negative regulation of binding of sperm to zona pellucida	0.000258558	0.070896722	5
TG	DEL	GO	GO:0070286	axonemal dynein complex assembly	0.000282976	0.074273165	17
TG	DEL	GO	GO:0042255	ribosome assembly	0.00028893	0.074273165	46
TG	DEL	GO	GO:0000027	ribosomal large subunit assembly	0.000340018	0.084757224	30
TG	DEL	GO	GO:0060088	auditory receptor cell stereocilium organization	0.000381122	0.092209063	11
TG	DEL	GO	GO:0071346	cellular response to type II interferon	0.000415491	0.097652275	53
TG	DEL	GO	GO:0019835	cytolysis	0.000618544	0.137517293	13
TG	DEL	GO	GO:0070587	regulation of cell-cell adhesion involved in gastrulation	0.00103577	0.22421693	12
TG	DEL	GO	GO:0002093	auditory receptor cell morphogenesis	0.00109587	0.231144286	11
TG	DEL	GO	GO:0016339	calcium-dependent cell-cell adhesion	0.001148504	0.236189787	17
TG	DEL	GO	GO:0045943	positive regulation of transcription by RNA polymerase I	0.001262839	0.253368614	14
TG	DEL	GO	GO:0080154	regulation of fertilization	0.001624506	0.310771712	20
TG	DEL	GO	GO:0071378	cellular response to growth hormone stimulus	0.001844517	0.337177777	16
TG	DEL	GO	GO:0061028	establishment of endothelial barrier	0.002099243	0.367412261	20
TG	DEL	GO	GO:0000740	nuclear membrane fusion	0.00243511	0.417317029	6
TG	DEL	GO	GO:0034115	negative regulation of heterotypic cell-cell adhesion	0.002659005	0.446387266	11
TG	DEL	GO	GO:0044774	mitotic DNA integrity checkpoint signaling	0.00294335	0.476855108	40
TG	DEL	GO	GO:1903859	regulation of dendrite extension	0.002956432	0.476855108	15
TG	DEL	GO	GO:0051307	meiotic chromosome separation	0.003429152	0.525743041	12
TG	DEL	GO	GO:0001885	endothelial cell development	0.003802769	0.535230786	25
TG	DEL	GO	GO:0002399	MHC class II protein complex assembly	0.003838879	0.535230786	5
TG	DEL	GO	GO:0002503	peptide antigen assembly with MHC class II protein complex	0.003838879	0.535230786	5
TG	DEL	GO	GO:1904170	regulation of bleb assembly	0.003838879	0.535230786	5
TG	DEL	GO	GO:0031573	mitotic intra-S DNA damage checkpoint signaling	0.003934404	0.539406814	11
TG	DEL	GO	GO:0044773	mitotic DNA damage checkpoint signaling	0.004159434	0.560909949	38

TG	DEL	GO	GO:0000724	double-strand break repair via homologous recombination	0.004680225	0.571695115	34
TG	DEL	GO	GO:0060467	negative regulation of fertilization	0.004949903	0.571695115	9
TG	DEL	GO	GO:0035606	peptidyl-cysteine S-trans-nitrosylation	0.005101266	0.571695115	6
TG	DEL	GO	GO:0070269	pyroptotic inflammatory response	0.005101266	0.571695115	6
TG	DEL	GO	GO:2000258	negative regulation of protein activation cascade	0.005101266	0.571695115	6
TG	DEL	GO	GO:0000725	recombinational repair	0.005425986	0.571695115	34
TG	DEL	GO	GO:0006013	mannose metabolic process	0.005470089	0.571695115	7
TG	DEL	GO	GO:0097374	sensory neuron axon guidance	0.005470089	0.571695115	7
TG	DEL	GO	GO:1904076	regulation of estrogen biosynthetic process	0.005470089	0.571695115	7
TG	DEL	GO	GO:1904077	negative regulation of estrogen biosynthetic process	0.005470089	0.571695115	7
TG	DEL	GO	GO:1905489	regulation of sensory neuron axon guidance	0.005470089	0.571695115	7
TG	DEL	GO	GO:2000183	negative regulation of progesterone biosynthetic process	0.005470089	0.571695115	7
TG	DEL	GO	GO:0001580	detection of chemical stimulus involved in sensory perception of bitter taste	0.00562757	0.571695115	13
TG	DEL	GO	GO:0009595	detection of biotic stimulus	0.00562757	0.571695115	13
TG	DEL	GO	GO:0002505	antigen processing and presentation of polysaccharide antigen via MHC class II	0.005714695	0.571695115	4
TG	DEL	GO	GO:0071823	protein-carbohydrate complex subunit organization	0.005714695	0.571695115	4
TG	DEL	GO	GO:0080144	intracellular amino acid homeostasis	0.005714695	0.571695115	4
TG	DEL	GO	GO:0086046	membrane depolarization during SA node cell action potential	0.005714695	0.571695115	4
TG	DEL	GO	GO:0034114	regulation of heterotypic cell-cell adhesion	0.006284943	0.615475456	14
TG	DEL	GO	GO:0090038	negative regulation of protein kinase C signaling	0.006683567	0.639290981	12
TG	DEL	GO	GO:0098581	detection of external biotic stimulus	0.006683567	0.639290981	12
TG	DEL	GO	GO:0060122	inner ear receptor cell stereocilium organization	0.006819864	0.644829886	15
TG	DEL	GO	GO:0086010	membrane depolarization during action potential	0.007242576	0.675407984	16
TG	DEL	GO	GO:0006356	regulation of transcription by RNA polymerase I	0.007565188	0.675407984	17
TG	DEL	GO	GO:0008594	photoreceptor cell morphogenesis	0.007911833	0.675407984	11

TG	DEL	GO	GO:0071539	protein localization to centrosome	0.007911833	0.675407984	11
TG	DEL	GO	GO:0072378	blood coagulation, fibrin clot formation	0.007911833	0.675407984	11
TG	DEL	GO	GO:1905508	protein localization to microtubule organizing center	0.007911833	0.675407984	11
TG	DEL	GO	GO:0060333	type II interferon-mediated signaling pathway	0.008052694	0.675407984	20
TG	DEL	GO	GO:0072376	protein activation cascade	0.008052694	0.675407984	20
TG	DEL	GO	GO:0045005	DNA-templated DNA replication maintenance of fidelity	0.008226574	0.675407984	14
TG	DEL	GO	GO:0050913	sensory perception of bitter taste	0.008226574	0.675407984	14
TG	DEL	GO	GO:0033685	negative regulation of luteinizing hormone secretion	0.008403732	0.675407984	8
TG	DEL	GO	GO:0036159	inner dynein arm assembly	0.008403732	0.675407984	8
TG	DEL	GO	GO:1901838	positive regulation of transcription of nucleolar large rRNA by RNA polymerase I	0.008403732	0.675407984	8
TG	DEL	GO	GO:0002396	MHC protein complex assembly	0.008621181	0.675407984	5
TG	DEL	GO	GO:0002501	peptide antigen assembly with MHC protein complex	0.008621181	0.675407984	5
TG	DEL	GO	GO:0035385	Roundabout signaling pathway	0.008621181	0.675407984	5
TG	DEL	GO	GO:2001188	regulation of T cell activation via T cell receptor contact with antigen bound to MHC molecule on antigen presenting cell	0.008621181	0.675407984	5
TG	DEL	GO	GO:0060117	auditory receptor cell development	0.009034334	0.685555932	12
TG	DEL	GO	GO:0006651	diacylglycerol biosynthetic process	0.009145478	0.685555932	7
TG	DEL	GO	GO:0060294	cilium movement involved in cell motility	0.009145478	0.685555932	7
TG	DEL	GO	GO:2000257	regulation of protein activation cascade	0.009167415	0.685555932	16
TG	DEL	GO	GO:0060623	regulation of chromosome condensation	0.009412417	0.687600395	6
TG	DEL	GO	GO:2000344	positive regulation of acrosome reaction	0.009412417	0.687600395	6
TG	DEL	GO	GO:0009988	cell-cell recognition	0.00944552	0.687600395	24
TG	DEL	GO	GO:0050892	intestinal absorption	0.00975361	0.692753805	19
TG	DEL	GO	GO:0007595	lactation	0.009768957	0.692753805	21
MG	DEL	KEGG	ko04740	Olfactory transduction	6.33E-94	2.27E-91	448

MG	DEL	KEGG	ko03010	Ribosome	7.12E-12	1.27E-09	186
MG	DEL	KEGG	ko05134	Legionellosis	0.000108372	0.012932397	48
MG	DEL	KEGG	ko05332	Graft-versus-host disease	0.000419147	0.037513657	26
MG	DEL	KEGG	ko05330	Allograft rejection	0.001169698	0.072437363	27
MG	DEL	KEGG	ko04940	Type I diabetes mellitus	0.001214034	0.072437363	30
MG	DEL	KEGG	ko05150	Staphylococcus aureus infection	0.001558842	0.072798097	33
MG	DEL	KEGG	ko04512	ECM-receptor interaction	0.001636119	0.072798097	41
MG	DEL	KEGG	ko00830	Retinol metabolism	0.00183012	0.072798097	37
MG	DEL	KEGG	ko00511	Other glycan degradation	0.003064139	0.10969617	18
MG	DEL	KEGG	ko00040	Pentose and glucuronate interconversions	0.004346814	0.140794149	18
MG	DEL	KEGG	ko02010	ABC transporters	0.004719357	0.140794149	25
PG	DEL	KEGG	ko04740	Olfactory transduction	8.77E-63	3.08E-60	327
PG	DEL	KEGG	ko05320	Autoimmune thyroid disease	5.59E-05	6.54E-03	35
PG	DEL	KEGG	ko05150	Staphylococcus aureus infection	0.00030828	0.027051602	29
PG	DEL	KEGG	ko05330	Allograft rejection	0.000392498	0.027553374	23
PG	DEL	KEGG	ko05134	Legionellosis	0.000551625	0.032270086	36
PG	DEL	KEGG	ko04612	Antigen processing and presentation	0.000886007	0.044426914	31
PG	DEL	KEGG	ko04512	ECM-receptor interaction	0.001023837	0.04492086	33
PG	DEL	KEGG	ko05332	Graft-versus-host disease	0.00158956	0.060286589	20
PG	DEL	KEGG	ko04940	Type I diabetes mellitus	0.001717567	0.060286589	24
PG	DEL	KEGG	ko05310	Asthma	0.007669847	0.244737847	16
TG	DEL	KEGG	ko04740	Olfactory transduction	3.73E-69	1.33E-66	380
TG	DEL	KEGG	ko05320	Autoimmune thyroid disease	1.67E-07	2.99E-05	43
TG	DEL	KEGG	ko05332	Graft-versus-host disease	4.28E-06	3.89E-04	28
TG	DEL	KEGG	ko04512	ECM-receptor interaction	4.36E-06	3.89E-04	44

TG	DEL	KEGG	ko05330	Allograft rejection	6.60E-06	4.71E-04	30
TG	DEL	KEGG	ko04940	Type I diabetes mellitus	3.84E-05	2.28E-03	31
TG	DEL	KEGG	ko05416	Viral myocarditis	0.000575625	0.029356868	38
TG	DEL	KEGG	ko05134	Legionellosis	0.000685711	0.029399257	40
TG	DEL	KEGG	ko04610	Complement and coagulation cascades	0.000741158	0.029399257	41
TG	DEL	KEGG	ko05150	Staphylococcus aureus infection	0.001155658	0.037506353	30
TG	DEL	KEGG	ko02010	ABC transporters	0.002367778	0.069128904	21
TG	DEL	KEGG	ko04612	Antigen processing and presentation	0.002517299	0.069128904	34
TG	DEL	KEGG	ko03460	Fanconi anemia pathway	0.003206197	0.081758022	23
TG	DEL	KEGG	ko05310	Asthma	0.007167249	0.170580532	18

Table S32. Potential historical distribution regions of *Procapra* species.

Species	Potential distribution region	Source
MG	Chuisk (Chuya) steppe (southeastern Altai)	(Zhang et al. 1995; Sokolov and Lushchekina 1997; Jin and Gu 2005)
	Tuva	
	Baikal	
	Sanjiang plain	
	Harbin	
	Liaohe River	
	Hebei province	
	The Yellow River Basin	
	Ningxia Province (Helan, Yinchuan, Yongning, Zhongwei, Tongxin, Yanchi, Lingwu)	
	Gansu Province (Huanxian, Shandan, Subei)	
	Jilin Province (Zhenlai, Tongyu, Taonan)	
PG	Inner Mongolia (Ordos and Alashan plateaus)	(Jiang et al. 1995; Leslie et al. 2010)
	Gansu Province (Hexi Corridor)	
	Ningxia Province (Helan Mountains)	
	eastern Qinghai Province	
TG	Sichuan Province (Shiqu, Aba, Ruoergai, Rangtang, Hongyuan, Seda, Batang, Litang, Ganzi, Dege, Luhuo, Daofu, Danba, Qingchuan, Pingwu, Wanyuan, Nanjiang, Tongjiang, Qionglai, Dayi, Pengzhou, Shifang, Chongzhou, Dujiangyan)	(Lu and Wang 2003; Lu and Wang 2004; Leslie 2010)
	Chongqing Municipality (Chengkou)	
	Gansu Province (Sunan, Aksay, Subei Yanchiwan, Gannan)	

	Qinghai Province (Kunlun-Altyn Tagh Basin, Headwaters of Yangtze River, Menyuan, Qilian, Tianjun, Haiyan, Southern Mountains of Golmud, Banma, Qumalai, Yushu, Golog, Huangnan, Gonghe, Yeniugou Nature Reserve, Kunlun Mountains)	
	Tibet Autonomous Region (Dingqing, Mangkang, Basu, Nyainrong, Amdo, Changtang, Nagqu, Ngari, Dingri Grassland, Shishapangma)	
	Ladakh Region	
	Sikkim State	

References:

Cho YS, Hu L, Hou H, Lee H, Xu J, Kwon S, Oh S, Kim HM, Jho S, Kim S, et al. 2013. The tiger genome and comparative analysis with lion and snow leopard genomes. *Nat Commun.* 4:2433.

Corbett-Detig RB, Hartl DL, Sackton TB. 2015. Natural selection constrains neutral diversity across a wide range of species. *PLoS Biol.* 13:e1002112.

Dussex N, Alberti F, Heino MT, Olsen RA, van der Valk T, Ryman N, Laikre L, Ahlgren H, Askeyev IV, Askeyev OV, et al. 2020. Moose genomes reveal past glacial demography and the origin of modern lineages. *BMC Genomics.* 21:854.

Heighton SP, Allio R, Murienne J, Salmona J, Meng H, Scornavacca C, Bastos ADS, Njiokou F, Pietersen DW, Tilak MK, et al. 2023. Pangolin genomes offer key insights and resources for the world's most trafficked wild mammals. *Mol Biol Evol.* 40:msad190.

Humble E, Dobrynin P, Senn H, Chuven J, Scott AF, Mohr DW, Dudchenko O, Omer AD, Colaric Z, Lieberman Aiden E, et al. 2020. Chromosomal-level genome assembly of the scimitar-horned oryx: insights into diversity and demography of a species extinct in the wild. *Mol Ecol Resour.* 20:1668-1681.

Jiang Z, Feng Z, Wang Z, Chen L, Cai P, Li Y. 1995. Historical and current distributions of Przewalski's gazelles. *Acta Theriol Sin.* 15:241-245.

Jin K, Gu Z. 2005. Distribution of population for Mongolian gazelle in China and its status. *J Northeast For Univ.* 33:155-157.

Jirimutu, Wang Z, Ding G, Chen G, Sun Y, Sun Z, Zhang H, Wang L, Hasi S, Zhang Y, et al. 2012. Genome sequences of wild and domestic Bactrian camels. *Nat Commun.* 3:1202.

Kuang W. 2021. Conservation genomics of the snub-nosed monkey and Tibetan antelope [dissertation]. Kunming: Yunnan University.

Leslie DM Jr. 2010. *Procapra picticaudata* (Artiodactyla: Bovidae). *Mamm Species.* 42:138-148.

Leslie DM Jr, Groves CP, Abramov AV. 2010. *Procapra przewalskii* (Artiodactyla: Bovidae). *Mamm Species.* 42:124-137.

Li A, Yang Q, Li R, Dai X, Cai K, Lei Y, Jia K, Jiang Y, Zan L. 2023. Chromosome-level genome assembly for takin (*Budorcas taxicolor*) provides insights into its taxonomic status and genetic diversity. *Mol Ecol.* 32:1323-1334.

Li R, Fan W, Tian G, Zhu H, He L, Cai J, Huang Q, Cai Q, Li B, Bai Y, et al. 2010. The sequence and de novo assembly of the giant panda genome. *Nature.* 463:311-317.

Liu S, Westbury MV, Dussex N, Mitchell KJ, Sinding MHS, Heintzman PD, Duchêne DA, Kapp JD, von Seth J, Heiniger H, et al. 2021. Ancient and modern genomes unravel the evolutionary history of the rhinoceros family. *Cell.* 184:4874-4885.e16.

Lu Q, Wang X. 2003. General situation of biology study to Tibetan gazelle. *Bull Biol.* 38:24-25.

Lu Q, Wang X. 2004. Status and progress of Tibetan gazelle study. *J Neijiang Norm Univ.* 19:42-48.

Morin PA, Archer FI, Avila CD, Balacco JR, Bukhman YV, Chow W, Fedrigo O, Formenti G, Fronczek JA, Fungtammasan A, et al. 2021. Reference genome and demographic history of the most endangered marine mammal, the vaquita. *Mol Ecol Resour.* 21:1008-1020.

Palkopoulou E, Lipson M, Mallick S, Nielsen S, Rohland N, Baleka S, Karpinski E, Ivancevic AM, To TH, Kortschak RD, et al. 2018. A comprehensive genomic history of extinct and living elephants. *Proc Natl Acad Sci U S A.* 115:E2566-E2574.

Pečnerová P, Lord E, Garcia-Erill G, Hanghøj K, Rasmussen MS, Meisner J, Liu X, van der Valk T, Santander CG, Quinn L, et al. 2024. Population genomics of the muskox's resilience in the near absence of genetic variation. *Mol Ecol.* 33:e17205.

Sokolov VE, Lushchekina AA. 1997. *Procapra gutturosa*. *Mamm Species.* 571:1-5.

Upadhyay M, Kunz E, Sandoval-Castellanos E, Hauser A, Krebs S, Graf A, Blum H, Dotsev A, Okhlopkov I, Shakhin A, et al. 2021. Whole genome sequencing reveals a complex introgression history and the basis of adaptation to subarctic climate in wild sheep. *Mol Ecol.* 30:6701-6717.

von Seth J, Dussex N, Díez-del-Molino D, van der Valk T, Kutschera VE, Kierczak M, Steiner CC, Liu S, Gilbert MTP, Sinding MHS, et al. 2021. Genomic insights into the conservation status of the world's last remaining Sumatran rhinoceros populations. *Nat Commun.* 12:2393.

Wang K, Wang L, Lenstra JA, Jian J, Yang Y, Hu Q, Lai D, Qiu Q, Ma T, Du Z, et al. 2017. The genome sequence of the wisent (*Bison bonasus*). *Gigascience.* 6:gix016.

Weldenegodguad M, Pokharel K, Ming Y, Honkatukia M, Peippo J, Reilas T, Røed KH, Kantanen J. 2020. Genome sequence and comparative analysis of reindeer (*Rangifer tarandus*) in northern Eurasia. *Sci Rep.* 10:8980.

Westbury MV, Hartmann S, Barlow A, Wiesel I, Leo V, Welch R, Parker DM, Sicks F, Ludwig A, Dalén L, et al. 2018. Extended and continuous decline in effective population size results in low genomic diversity in the world's rarest hyena species, the brown hyena. *Mol Biol Evol.* 35:1225-1237.

Zhang Z, Sun J, Bai S, Wang Z. 1995. The vicissitudes of gazelle (*Procapra gutturosa*) distribution in China and the sustainable use of the resources. *Biodivers Sci.* 3:95-98.

Zhou X, Sun F, Xu S, Fan G, Zhu K, Liu X, Chen Y, Shi C, Yang Y, Huang Z, et al. 2013. Baiji genomes reveal low genetic variability and new insights into secondary aquatic adaptations. *Nat Commun.* 4:2708.

Zhu L, Deng C, Zhao X, Ding J, Huang H, Zhu S, Wang Z, Qin S, Ding Y, Lu G, et al. 2018. Endangered Père David's deer genome provides insights into population recovering. *Evol Appl.* 11:2040-2053.
